# Ferroptosis in Cancer and Inflammatory Diseases: Mechanisms and Therapeutic Implications

**DOI:** 10.1002/mco2.70349

**Published:** 2025-09-03

**Authors:** Guangyi Shen, Jiachen Liu, Yinhuai Wang, Zebin Deng, Fei Deng

**Affiliations:** ^1^ Department of Urology The Second Xiangya Hospital Central South University Changsha Hunan China; ^2^ The Center of Systems Biology and Data Science School of Basic Medical Science Central South University Changsha Hunan China; ^3^ Xiangya Hospital Central South University Changsha Hunan China; ^4^ National Clinical Research Center for Metabolic Disease Key Laboratory of Diabetes Immunology (Central South University) Ministry of Education Changsha China; ^5^ Department of Nephrology The Second Xiangya Hospital Central South University Changsha Hunan China

**Keywords:** ferroptosis, lipid peroxidation, cancer immunotherapy, inflammatory signaling, tumor microenvironment

## Abstract

Ferroptosis, an iron‐dependent cell death pathway driven by lipid peroxidation, has emerged as a critical pathophysiological mechanism linking cancer and inflammatory diseases. The seemingly distinct pathologies exhibit shared microenvironmental hallmarks—oxidative stress, immune dysregulation, and metabolic reprogramming—that converge on ferroptosis regulation. This review synthesizes how ferroptosis operates at the intersection of these diseases, acting as both a tumor‐suppressive mechanism and a driver of inflammatory tissue damage. In cancer, ferroptosis eliminates therapy‐resistant cells but paradoxically facilitates metastasis through lipid peroxidation byproducts that remodel the tumor microenvironment and suppress antitumor immunity. In chronic inflammatory diseases—from atherosclerosis to rheumatoid arthritis—ferroptosis amplifies neuroinflammatory cascades while simultaneously exposing vulnerabilities for therapeutic targeting. Central to this duality are shared regulatory nodes, including nuclear factor kappa B‐driven inflammation, NOD‐like receptor family pyrin domain‐containing 3 inflammasome activation, and GPX4 dysfunction. Therapeutically, ferroptosis induction shows promise against therapy‐resistant cancers but risks exacerbating inflammatory damage, underscoring the need for precision modulation. Emerging strategies—nanoparticle‐based inducers, immunotherapy combinations, and biomarker‐guided patient stratification—aim to balance prodeath efficacy against off‐target toxicity. By dissecting the ferroptosis–inflammation–cancer axis, this review provides a unified framework for understanding disease pathogenesis and advancing therapies for conditions resistant to conventional treatments. Future research must prioritize spatial mapping of ferroptosis dynamics, mechanistic crosstalk with immune checkpoints, and combinatorial regimens that exploit ferroptosis vulnerabilities while mitigating its inflammatory consequences.

## Graphical abstract

Ferroptosis, a form of cell death driven by iron and lipid peroxidation, serves as a bridge between cancer and inflammatory diseases. Microenvironmental factors such as oxidative stress collectively regulate this process. In cancer, ferroptosis exhibits dual roles: while it eliminates drug‐resistant cells, it may also promote tumor metastasis. During inflammatory processes, it exacerbates tissue damage yet simultaneously presents therapeutic targets. Inducing ferroptosis shows promise in combating cancer, though it risks amplifying inflammation. Emerging strategies focus on balancing these dual effects while optimizing therapeutic outcomes.

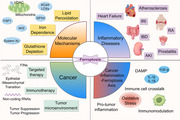

## 1 Introduction

The discovery of ferroptosis—an iron (Fe)‐dependent cell death pathway driven by lipid peroxidation (LPO)—has reshaped our understanding of disease pathogenesis across oncology and immunology [1, 2]. Unlike classical apoptotic or necrotic cell death, ferroptosis is defined by its unique biochemical signature: glutathione (GSH) peroxidase 4 (GPX4) inactivation, Fe‐mediated Fenton reactions, and phospholipid peroxidation. The core features position ferroptosis at the nexus of oxidative stress, metabolic reprogramming, and immune dysregulation, making it a critical but underappreciated link between seemingly disparate diseases.

**FIGURE 1:**
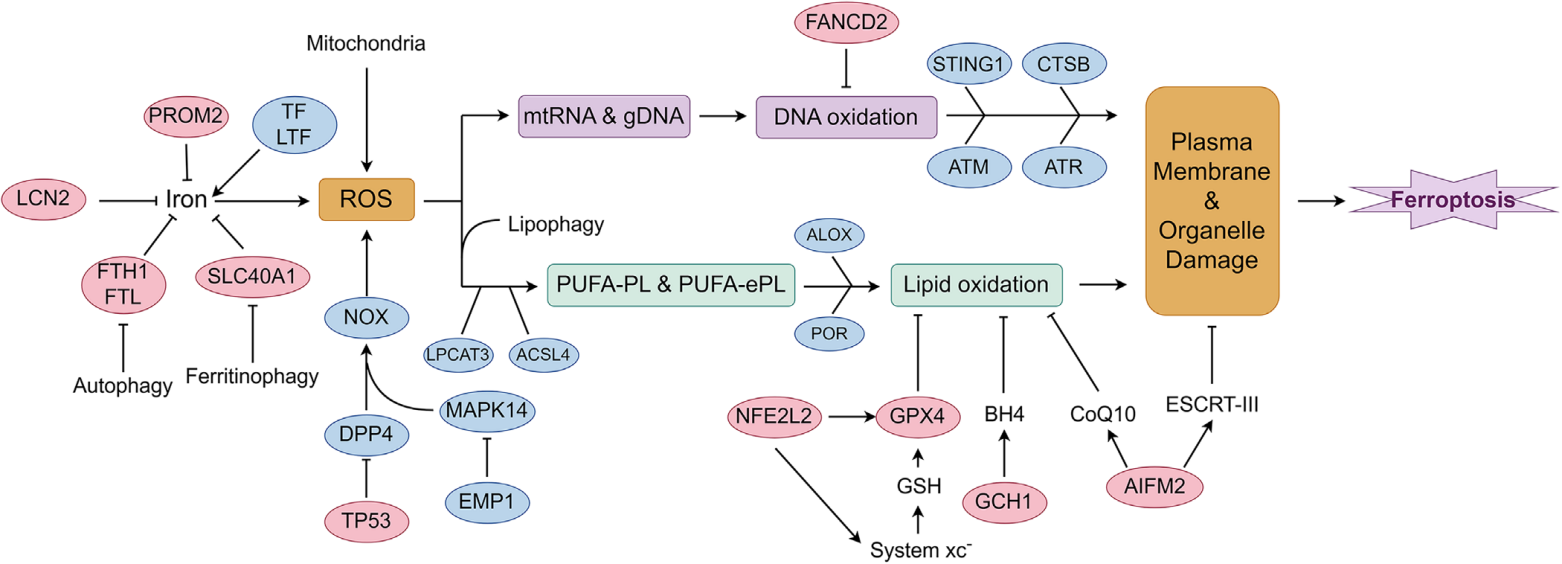
Signaling pathways of ferroptosis. Ferroptosis is initiated by excessive lipid peroxidation and organelle damage resulting from disrupted redox and iron homeostasis. Iron uptake and metabolism are mediated by transferrin (TF), lactotransferrin (LTF), and divalent metal transporter SLC40A1, while storage involves ferritin heavy chain (FTH1), light chain (FTL), and autophagic ferritin degradation (ferritinophagy). Lipocalin 2 (LCN2) and prominin 2 (PROM2) contribute to extracellular iron modulation. Intracellular iron overload catalyzes reactive oxygen species (ROS) generation via Fenton chemistry, amplifying oxidative damage and mitochondrial stress. NADPH oxidase (NOX) and ROS accumulation activate downstream signaling including mitogen‐activated protein kinase 14 (MAPK14) and epithelial membrane protein 1 (EMP1), modulated by tumor suppressor p53 (TP53) and dipeptidyl peptidase 4 (DPP4). ROS also induces mitochondrial lipophagy, releasing polyunsaturated fatty acyl phospholipids (PUFA‐PLs) and ether phospholipids (PUFA‐ePLs), which are converted by ACSL4 and LPCAT3 into substrates for lipid peroxidation by lipoxygenases (ALOX) and cytochrome P450 oxidoreductase (POR). Unchecked lipid peroxidation disrupts plasma and organelle membranes, culminating in ferroptosis. Nuclear factor erythroid 2–related factor 2 (NFE2L2) transcriptionally regulates system Xc^−^ (SLC7A11 and SLC3A2), which imports cystine for glutathione (GSH) synthesis. GSH, along with GPX4, prevents lipid peroxide accumulation; its depletion enhances ferroptotic sensitivity. GCH1 and tetrahydrobiopterin (BH4), along with coenzyme Q10 (CoQ10) and the ESCRT‐III complex, act as parallel ferroptosis defense systems. DNA oxidative damage, triggered by ROS and mediated by FANCD2, activates the ATM–ATR–STING1–CTSB axis, reinforcing membrane breakdown. AIFM2 may promote mitochondrial injury and ferroptosis under CoQ10‐deficient conditions. Collectively, ferroptosis emerges from a convergence of iron dysregulation, lipid oxidation, and compromised antioxidant defenses.

Emerging evidence reveals that cancer and inflammatory disorders, though clinically distinct, share ferroptosis‐driven pathophysiological convergence [3, 4]. Both disease states exhibit microenvironmental Fe overload, chronic oxidative stress, and lipid peroxide accumulation—conditions that prime cells for ferroptosis. In cancer, ferroptosis acts as an evolutionary double agent: it eliminates therapy‐resistant tumor cells through lethal LPO while paradoxically promoting metastasis via proinflammatory byproducts that reshape the tumor microenvironment (TME) [5]. Similarly, in chronic inflammatory diseases, ferroptosis amplifies tissue damage through neuroinflammatory cascades but simultaneously exposes druggable vulnerabilities in dysregulated Fe metabolism (Figure 1). This duality highlights ferroptosis as a molecular fulcrum balancing cellular survival and inflammatory pathology.

The shared ferroptosis–inflammation axis is further reinforced by conserved regulatory nodes. Nuclear factor kappa B (NF‐κB) activation, NOD‐like receptor family pyrin domain‐containing 3 (NLRP3) inflammasome signaling, and GPX4/GSH depletion emerge as common denominators across cancer progression and inflammatory tissue injury. For instance, tumor‐associated macrophages (TAMs) in the TME and synovial fibroblasts in rheumatoid arthritis (RA) both undergo ferroptosis in response to analogous oxidative triggers, releasing damage‐associated molecular patterns (DAMPs) that perpetuate disease‐specific inflammation [6, 7]. Such parallels suggest that therapeutic strategies targeting ferroptosis regulators—such as LPO repair enzymes or Fe chelation—could transcend traditional disease classifications.

This review bridges the cancer–inflammation divide by dissecting ferroptosis as a unified pathomechanism. We first establish the biochemical principles of ferroptosis and its immunometabolic crosstalk, then explore its context‐dependent roles in tumor evolution versus inflammatory tissue destruction. By mapping shared vulnerabilities and divergent consequences, we provide a framework for developing ferroptosis‐modulating therapies. Finally, we discuss emerging strategies to exploit ferroptosis in drug‐resistant cancers while mitigating its inflammatory collateral damage—a critical balance for precision medicine.

## 2 The Concept of Ferroptosis

The cellular response to oxidative stress is an inevitable and crucial aspect of life processes in aerobic organisms, which rely on oxygen as the primary oxidant in oxidative metabolism [8]. Oxidative stress is typically defined as the disruption of the balance between the accumulation of reactive oxygen/nitrogen species (ROS/RNS) and the capacity of antioxidant defense mechanisms in the organism to counteract their effects [9]. This imbalance can result from either an overload of ROS/RNS or a reduction in antioxidant capacity, characterized by a diminished ability of cells to resist oxidative attacks by electron‐deficient molecules [9]. Oxidative stress has been closely associated with the development of conditions such as cancer [10, 11], neurodegenerative diseases [12], and ischemic organ injury [13]. Lipid oxidation modifications within cell membranes, a condition of cellular oxidative stress [14], serve as a nexus for receiving environmental stimuli and genetic inputs. These modifications influence redox homeostasis [15], immune surveillance [16, 17], and signal transduction [18], guiding cells in making decisions regarding ferroptosis [18].

**FIGURE 2:**
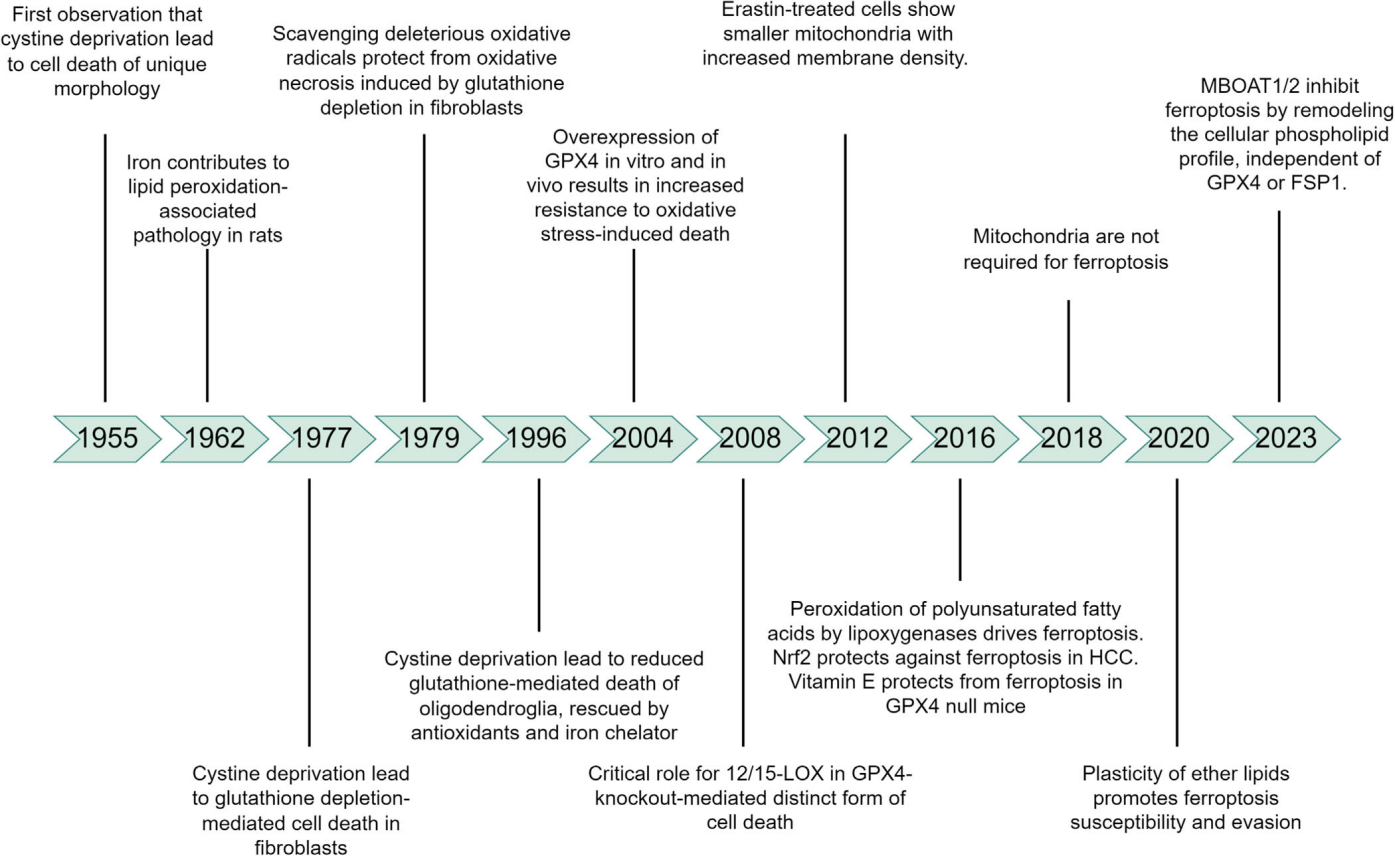
Development of the concept of ferroptosis. Starting in 1955 with the discovery of cell death due to cystine deprivation, key milestones follow. In 1962, iron's link to lipid peroxidation pathology emerged. Glutathione's antioxidant role was identified in 1977. Later, research uncovered the importance of enzymes like 12/15‐LOX (2008) and proteins such as GPX4 in ferroptosis. Emodin‐treated cell mitochondrial changes were noted in 2012, and polyunsaturated fatty acid peroxidation's role in 2016. By 2020, MBOAT1/2 were found to inhibit ferroptosis independently of GPX4 or FSP1. In 2023, the impact of ether lipid plasticity on ferroptosis was revealed. Each step deepens our understanding of the molecular and cellular mechanisms of ferroptosis.

The concept of ferroptosis, a form of regulated cell death (RCD) characterized by Fe‐dependent LPO, has evolved over several decades of research on Fe metabolism and oxidative stress. Early studies in the mid‐20th century laid the groundwork for understanding the toxic effects of Fe and LPO. For instance, as early as 1955, researchers observed that cystine deprivation induced a unique form of cell death that could not be solely attributed to impaired protein synthesis, suggesting additional metabolic roles for cystine, particularly in maintaining cellular redox balance through GSH synthesis [19, 20]. Further insights were gained in the 1970s when liver necrosis in mice, now recognized as ferroptosis, was linked to GSH depletion, which could be ameliorated by supplementation with GSH or its precursor, cysteine [21]. Bannai and colleagues [22] demonstrated that cysteine starvation led to decreased GSH levels and subsequent cell death, which could be rescued by the lipophilic antioxidant α‐tocopherol without restoring GSH, highlighting the role of lipid ROS in this death process. By 1996, Yonezawa et al. [23] induced oxidative death in oligodendrocytes through cysteine deprivation, which was preventable by free radical scavengers, including antioxidants and Fe chelators. These early findings, initially termed “oxidative glutamate toxicity” or “oxytosis,” were precursors to the formal identification of ferroptosis [24]. Subsequent research solidified the understanding that cellular cysteine deprivation and GSH depletion are critical for inducing this form of cell death, which can be inhibited by Fe chelators or lipophilic antioxidants [25]. These characteristics are now hallmarks of ferroptosis. In 2012, Dixon et al. [2] formally defined ferroptosis as an Fe‐dependent form of RCD driven by LPO, distinct from other cell death modalities. Their work, using RSL3 to induce ferroptosis in NRAS‐mutant fibrosarcoma cells, demonstrated that Fe chelation with deferoxamine (DFO) could prevent ROS accumulation and cell death, establishing the core features of ferroptosis. Figure 2 illustrates the timeline of key discoveries leading to the conceptualization of ferroptosis. This historical context provides a foundation for understanding the molecular mechanisms of ferroptosis.

### 2.1 Distinctiveness of RCD

RCD refers to the autonomous and orderly cellular demise controlled by genetic mechanisms to maintain internal environmental stability. The induction and execution of RCD are primarily regulated by the formation of signal amplification complexes, which play a crucial evolutionary role in development and immune responses [26]. When RCD occurs under physiological conditions, it is also referred to as programmed cell death (PCD) [27].

Unlike other forms of RCD, ferroptosis resembles cellular “destruction” more than an active “suicidal” mechanism [28]. While “suicidal” pathways like apoptosis, necroptosis, and pyroptosis are triggered by specialized molecular mechanisms dedicated to cell death, ferroptosis involves a “destruction” mechanism. This occurs when physiological processes become inactivated or excessively activated, resulting in lethal metabolic imbalances without the involvement of dedicated death proteins [29]. In cases of ferroptosis, cells experience ongoing metabolic “destruction” [30]. In Table 1, we compare the characteristics of all forms of RCDs.

**TABLE 1:** Classification of regulated cell death.

Types of cell death	Definition	Morphological characteristics	Biochemical characteristics	Regulatory pathways	Key genes	References
Ferroptosis	The nonapoptotic cell death characterized by iron‐dependent lipid peroxidation.	Mitochondria show reduced size, increased membrane density, decreased or absent cristae, mitochondrial outer membrane rupture, while the cell nucleus remains intact.	Iron accumulation and lipid peroxidation	System Xc^−^ –GPX4, MVA, HSPB1–TFR1, p62–Keap1–Nrf2, p53–SLC7A11	GPX4, TFR1, SLC7A11, Nrf2, p53, ACSL4, FSP1	[8, 31, 32]
Apoptosis	Genes regulate cell‐autonomous orderly death, maintaining internal stability.	Cell and nuclear volumes decrease, chromatin condenses, nuclei fragment, apoptotic bodies form, and cytoskeleton disintegrates, while mitochondrial structure remains unchanged.	DNA fragmentation reduces mitochondrial membrane potential.	Caspase, p53, Bcl‐2, Mitochondria‐ER, death receptor	Caspase, Bcl‐2, Bax, p53, Fas	[33, 34]
Necroptosis	The cell death mode initiated with a necrotic phenotype in the form of apoptosis.	Plasma membrane rupture, widespread cytoplasmic and organelle swelling, moderate chromatin condensation, leakage of cellular components into the microenvironment.	Enrichment of kinases and reduction in ATP levels	TNF, RIP1/RIP3–MLKL, TLR, PKC–MAPK–AP‐1	ATG5, ATG7, LC3, Beclin‐1	[35, 36, 37]
Pyroptosis	Pyroptosis mediated by pyroptin, dependent on inflammatory caspase activation.	Loss of membrane integrity, organelle disintegration, DNA condensation, and fragmentation.	Formation of inflammasomes, activation of caspase‐1, release of proinflammatory cytokines	Caspase‐1, NLRP3	Caspase‐1, IL‐1β, IL‐18	[38, 39, 40]
Autophagy	The process of lysosomal degradation of damaged organelles and macromolecules within cells is regulated by relevant genes.	Formation of double‐membrane autophagosomes, including macroautophagy, microautophagy, and chaperone‐mediated autophagy.	Enhanced lysosomal activity	mTOR, Beclin‐1, ATG, ULK1, PI3K, p53	RIP1, RIP3	[41, 42, 43, 44]

### 2.2 Definition and Core Characteristics

#### 2.2.1 Morphological Features

Morphologically, ferroptotic cells do not exhibit the typical features shown by other forms of RCD, such as chromatin condensation, apoptotic bodies, or autophagosome formation. However, they do contain mitochondria with increased membrane density and reduced cristae [2]. Initial studies found that ferroptosis morphologically differs from apoptosis, necrosis, or autophagy [2]. Still, it is generally believed that ferroptotic cells undergo morphological changes similar to necrosis, such as cytoplasmic granulation, organelle, and cell swelling [45]. Mitochondria play crucial roles in energy and lipid metabolism and maintain Fe homeostasis [46]. In ferroptosis, mitochondria exhibit unique features: loss of structural integrity, including reduced volume, increased membrane density, outer membrane rupture, and decreased or absent cristae [47]. Notably, unlike other forms of RCD, ferroptosis does not induce changes in nuclear structure.

#### 2.2.2 Biochemical Markers

Ferroptosis is a form of RCD characterized by two main biochemical features: Fe accumulation and LPO. Fe accumulation, particularly of intracellular redox‐active ferrous Fe, promotes the peroxidation of polyunsaturated fatty acids (PUFAs) in membrane phospholipids, leading to membrane damage [48]. This LPO is critically regulated by GPX4, which plays a central role in preventing ferroptosis by reducing lipid hydroperoxides to lipid alcohols [49]. The activity of GPX4 is supported by GSH, an antioxidant synthesized from cysteine and glutamate, with cysteine derived from extracellular cystine imported via the system Xc^−^ antiporter. System Xc^−^, a heterodimer of the 4F2 heavy subunit (SLC3A2) and the xCT light subunit (SLC7A11), facilitates the exchange of extracellular cystine for intracellular glutamate, thereby maintaining GSH levels essential for GPX4 function. For a comprehensive understanding of the GSH/System Xc^−^/GPX4 pathway and its role in ferroptosis, see section 3.4.1.

#### 2.2.3 Immunomodulatory Effects

Recently, the immunological characteristics of ferroptosis have been characterized, and its immunological consequences involve two aspects. First, ferroptosis can lead to the death of leukocyte subsets and the corresponding loss of immune function. For instance, LPO‐induced ferroptosis in T cells can promote viral or parasitic infections [50]. Second, and more importantly, when ferroptosis affects nonleukocytes, it determines how the immune system handles dying cells or the corpses generated. Different types of cell death can lead to various immune and inflammatory responses by releasing and activating different DAMP signals. Generally, ferroptosis is an inflammatory form of cell death associated with the release of DAMPs, such as high‐mobility group box 1 (HMGB1) and DNA or lipid oxidation products, such as 4‐hydroxynonenal (4‐HNE), oxPLs, LTB4, LTC4, LTD4, and PGE2 during tissue damage or tumor treatment. For example, the LPO product 4‐HNE is a proinflammatory mediator in ageing and chronic diseases that can activate the NF‐κB pathway [51]. HMGB1, a typical DAMP involved in various types of cell death [52], is released by Fe‐deposited cells and subsequently triggers an inflammatory response in peripheral macrophages by activating the receptor for advanced glycation end products (AGER/RAGE), a pattern recognition receptor (PRR) that activates the NF‐κB pathway in innate immunity [53]. Targeting lipid metabolism‐related DAMP signal transduction may be a promising strategy for treating inflammatory diseases associated with Fe‐related damage.

## 3 Molecular Mechanisms of Ferroptosis

The molecular execution of ferroptosis involves a dynamic interplay of metabolic pathways, organellar functions, and signaling networks. This section systematically dissects the core machinery driving ferroptosis, progressing from primary biochemical triggers (LPO dynamics and Fe dysregulation) to organelle‐specific contributions, and culminating in cross‐regulatory signaling axes that fine‐tune cell fate.

### 3.1 Dynamics of LPO

Unrestricted LPO is a hallmark of ferroptosis, where fatty acids (FAs) constitute a pivotal topic in cellular lipid metabolism. FAs are precursors to numerous biomolecules and play crucial roles in energy metabolism, cell membrane synthesis, signal transduction, and other vital cellular processes [54]. Ferroptosis is primarily propelled by the oxidation of PUFAs, notably catalyzed by bis‐allylic methylene groups within PUFAs [55]. One pathway involves the esterification of PUFAs (including arachidonic acid [AA] and adrenic acid or their acyl‐CoA forms) into phosphatidylethanolamine (PE), followed by PE oxidation within compartments associated with the endoplasmic reticulum, thereby transmitting ferroptotic signals [56, 57, 58].

**FIGURE 3:**
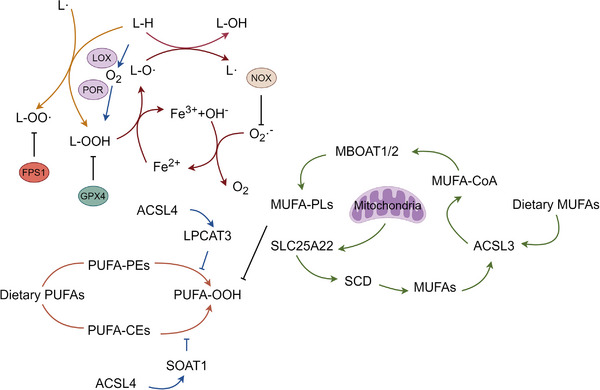
Lipid metabolism and lipid peroxidation pathways. Polyunsaturated fatty acids (PUFAs) derived from the diet are esterified by acyl‐CoA synthetase long‐chain family member 4 (ACSL4) into PUFA‐CoAs and incorporated into membrane phospholipids such as phosphatidylethanolamines (PUFA‐PEs) and cholesteryl esters (PUFA‐CEs) by lysophosphatidylcholine acyltransferase 3 (LPCAT3) and sterol O‐acyltransferase 1 (SOAT1), respectively. PUFA‐PEs undergo enzymatic oxidation mediated by lipoxygenases (LOX) and cytochrome P450 oxidoreductase (POR), producing lipid hydroperoxides (PUFA‐OOH), which are key death signals in ferroptosis. Lipid radicals (L•) and peroxyl radicals (LOO•) propagate peroxidation via hydrogen abstraction from adjacent lipids (L‐H), yielding hydroxylated lipids (L‐OH) and new radicals. Iron (Fe^2^⁺) participates in the Fenton reaction, converting hydrogen peroxide (H_2_O_2_) into highly reactive hydroxyl radicals (•OH), which further drive lipid peroxidation. NADPH oxidases (NOX) also contribute to ROS generation via superoxide (O^−^•^−^), amplifying oxidative stress. The cytosolic antioxidant glutathione peroxidase 4 (GPX4) detoxifies PUFA‐OOH to PUFA‐OH using glutathione, while the aquaporin channel FPS1 regulates lipid peroxide export. In contrast, monounsaturated fatty acids (MUFAs), either exogenously supplied or endogenously synthesized by stearoyl‐CoA desaturase (SCD), are activated by ACSL3 and incorporated into phospholipids by MBOAT1/2. These MUFA‐containing phospholipids (MUFA‐PLs) are transported via SLC25A22 into mitochondria, where they exhibit resistance to peroxidation, thereby antagonizing ferroptosis. This lipid selectivity highlights the balance between PUFA‐ and MUFA‐derived lipid pools in dictating ferroptosis sensitivity.

The initiation of LPO typically begins with binding the acyl chain of PUFAs to phospholipids within the lipid bilayer (PUFA‐PL). Subsequently, hydrogen atoms from bis‐allylic positions in the PUFA‐PL carbon–carbon double bonds are transferred, forming carbon‐cantered radicals (PL•). These radicals react with molecular oxygen to generate peroxyl radicals (PLOO•). These peroxyl radicals then facilitate dehydrogenating another PUFA, forming phospholipid hydroperoxides (PLOOH). When GPX4 fails to catalyze the conversion of PLOOH molecules into their corresponding alcohols (PLOH), PLOOH and lipid radicals, particularly PLOO• and alkoxyl phospholipid radicals (PLO•), continue to drive the dehydrogenation of PUFA‐PLs. This process results in generating a large number of PLOOH molecules through reactions with oxygen via the Fenton reaction [17, 59]. Ultimately, this cascade of responses generates many secondary products, including decomposition products of lipid peroxides such as 4‐HNE and malondialdehyde (MDA) and various oxidized and modified proteins. These reactions lead to the disruption of membrane integrity and ultimately accelerate the rupture of organelles and cell membranes [57]. Figure 3 demonstrates the lipid metabolism and LPO pathways.

The abundance and localization of PUFA within cells largely dictate the occurrence and extent of ferroptosis. Interestingly, in contrast to PUFA, MUFA have been demonstrated to serve as effective ferroptosis inhibitors [55, 60].

Multiple enzymes have been demonstrated to participate in driving LPO, all of which are Fe dependent. Among them, the lipoxygenase family (LOX) plays a crucial role in catalyzing the synthesis of lipid radicals directly, making it essential in the signaling cascade of ferroptosis [61]. LOXs are a class of dioxygenases that directly oxidize PUFAs and PUFA‐containing lipids in biological membranes. Free PUFAs are the preferred substrates for LOX, and knocking out LOX genes can effectively prevent erasin‐induced ferroptosis. Furthermore, specific known ferroptosis inhibitors, such as the vitamin E family (including various tocopherols and tocotrienols), are believed to regulate ferroptosis by inhibiting LOX activity [57, 62].

### 3.2 Fe Metabolism

Fe overload is a prerequisite for ferroptosis, and Fe metabolism plays a crucial role in the mechanism of ferroptosis [2]. Both the accumulation of LPO and the initiation of ferroptosis require Fe. It is evident from the process of LPO that Fe‐dependent enzymes catalyze it, and the Fenton chain reaction involving Fe is crucial for the accumulation of ROS [63]. An imbalance in Fe uptake, transport, and storage can affect the sensitivity to ferroptosis [64]. During ferroptosis, genes related to Fe metabolism are typically upregulated, and silencing of the critical regulator of Fe metabolism, iron‐responsive element‐binding protein 2 (IREB2), leads to reduced sensitivity to ferroptosis [2]. Conversely, induction/resistance of ferroptosis can be achieved through modulation of Fe input/output [65, 66, 67].

**FIGURE 4:**
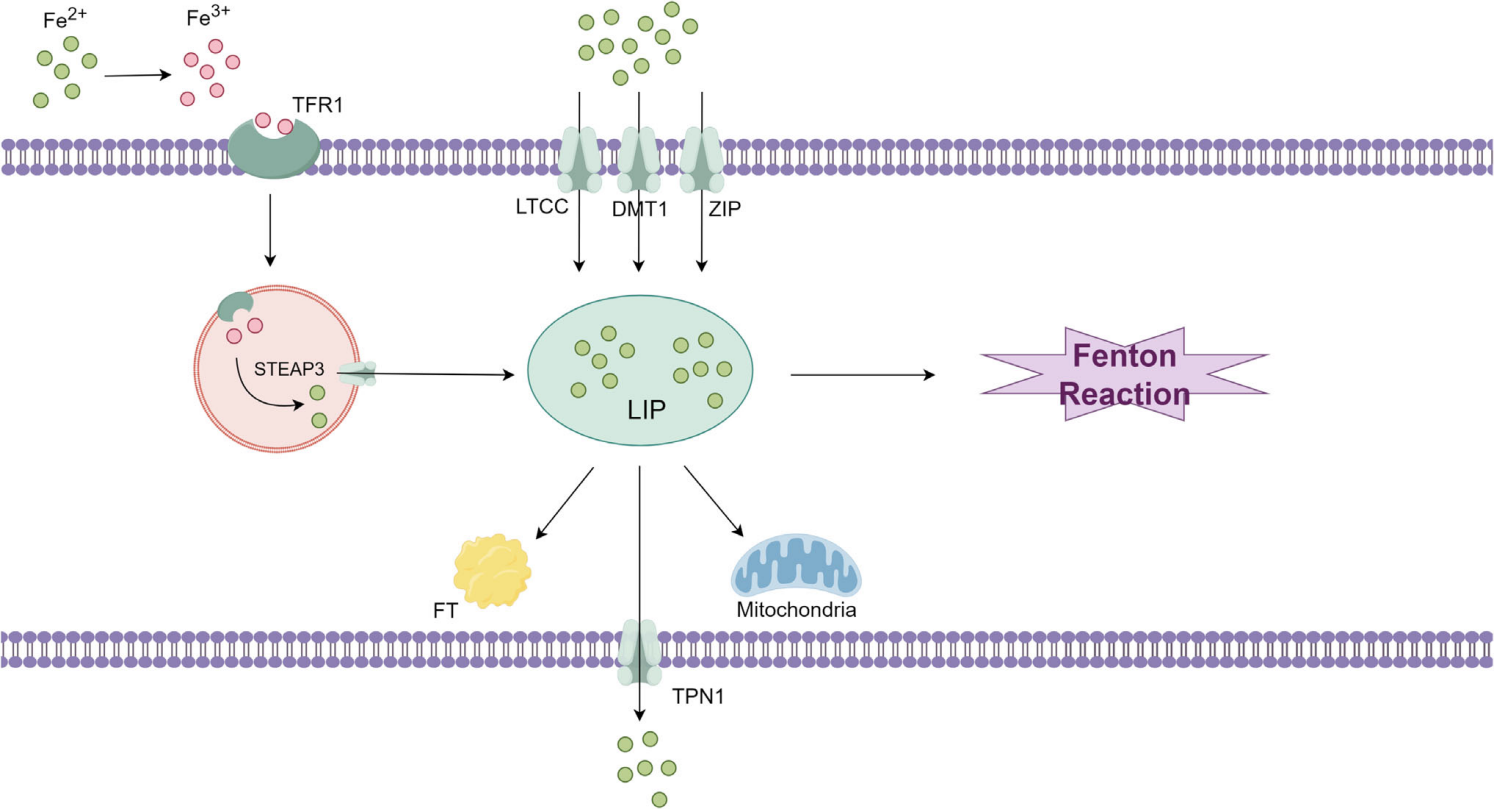
Pathways of iron metabolism. Extracellular Fe^3^⁺ is taken up by transferrin receptor 1 (TFR1) through binding of transferrin‐bound iron at the plasma membrane. Following endocytosis, six‐transmembrane epithelial antigen of prostate 3 (STEAP3) catalyzes the reduction of Fe^3^⁺ to Fe^2^⁺ within endosomes, facilitating its release into the cytoplasm and incorporation into the labile iron pool (LIP). Additional Fe^2^⁺ influx occurs via multiple transporters, including L‐type calcium channels (LTCC), divalent metal transporter 1 (DMT1), and ZRT/IRT‐like protein (ZIP) family channels. The LIP serves as a central hub for intracellular iron trafficking and distribution. Labile Fe^2^⁺ can be sequestered by ferritin (FT) for storage, transported into mitochondria for metabolic and biosynthetic processes or exported via transport protein 1 (TPN1) to maintain iron homeostasis. Excess Fe^2^⁺ in the LIP participates in the Fenton reaction, generating reactive oxygen species (ROS) that contribute to oxidative damage and ferroptosis. This compartmentalized system of iron acquisition, storage, utilization, and efflux tightly regulates cellular redox balance, while dysregulation leads to iron overload and ferroptotic cell death.

Under normal circumstances, Fe homeostasis is tightly regulated by Fe regulatory proteins IRP1 and IRP2 [68]. Extracellular Fe^3^⁺ ions initially bind to transferrin (TFR) and enter the cell through the membrane transferrin receptor 1 (TFR1) [69], primarily stored in ferritin. Fe^3^⁺ is then reduced to Fe^2^⁺ by the STEAP3 metalloreductase upon entering the cytoplasm, where it plays crucial physiological roles, including regulating energy metabolism and synthesizing iron–sulfur (Fe/S) proteins (Figure 4).

Genes associated with Fe metabolism are typically upregulated during the process of ferroptosis. IREB2 is a critical regulator of Fe metabolism, and its silencing affects the expression of transferrin receptor(TFRC), Iron–sulfur cluster scaffold homolog(ISCU), FTH1, and FTL during ferroptosis [2].

NCOA4 serves as the principal cargo receptor mediating ferritinophagy. Knockdown of NCOA4 diminishes intracellular free Fe levels and oxidative stress while concurrently elevating GSH levels [70], conferring resistance against ferroptosis [71]. Besides ferritinophagy, there is autophagic degradation of lipid droplets (LDs). In the context of RSL3‐induced ferroptosis in hepatocellular carcinoma cell lines, an initial increase followed by subsequent decrease in LD levels has been observed [72]. Autophagy‐mediated LD degradation facilitates LPO‐induced ferroptosis, which can be reversed through the depletion of LD cargo receptor RAB7A [72, 73]. It can be argued that a close relationship exists between selective autophagy and ferroptosis [65, 70, 74]. Nevertheless, the molecular crosstalk between ferroptosis and autophagic pathways remains incompletely understood, warranting further exploration into the molecular mechanisms through which autophagy influences ferroptosis [71].

### 3.3 Mitochondrial Involvement in Ferroptosis

**FIGURE 5:**
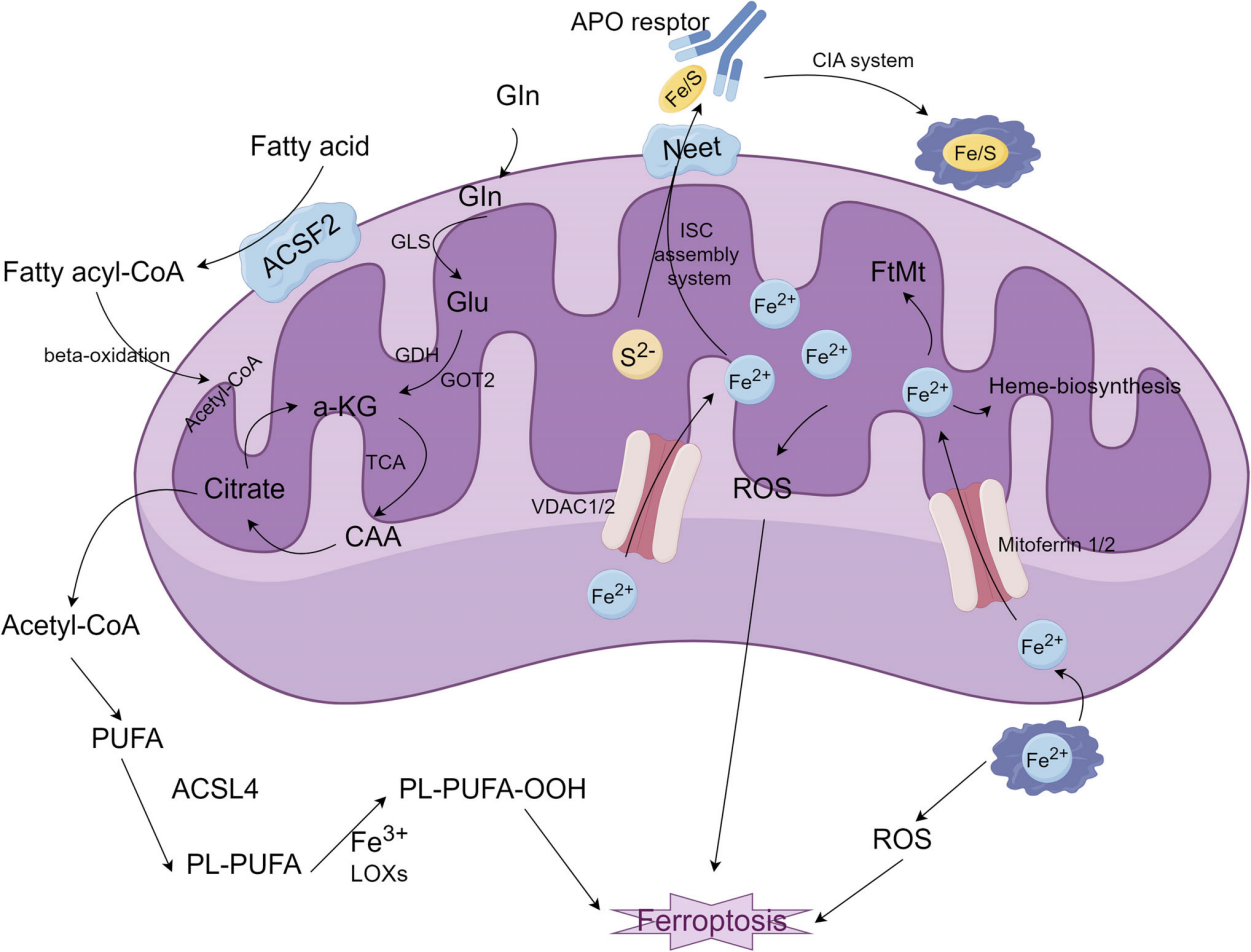
Mitochondrial regulatory pathways of ferroptosis. Mitochondria serve as a central hub for metabolic and redox processes that modulate ferroptosis susceptibility. Glutamine (Gln) is metabolized to glutamate (Glu) via glutaminase (GLS), which is further converted to α‐ketoglutarate (α‐KG) through glutamate dehydrogenase (GDH) or transaminated by glutamic‐oxaloacetic transaminase 2 (GOT2). Entry of α‐KG into the tricarboxylic acid (TCA) cycle generates citrate and downstream intermediates, collectively supporting mitochondrial respiration and carbon/amino acid anabolism (CAA). Concurrently, fatty acids undergo activation by acyl‐CoA synthetase family member 2 (ACSF2) and are imported into mitochondria for β‐oxidation, generating acetyl‐CoA that feeds into PUFA biosynthesis. These polyunsaturated fatty acids (PUFAs), when esterified into phospholipids by ACSL4, can undergo lipoxygenase (LOX)‐mediated peroxidation to generate lipid hydroperoxides (PL‐PUFA‐OOH), potent triggers of ferroptosis. Mitochondrial iron metabolism also contributes to ferroptosis. Ferrous iron (Fe^2^⁺) enters mitochondria via mitoferrin 1/2 and is utilized for heme biosynthesis or stored in mitochondrial ferritin (FtMt). Fe^2^⁺ also participates in the biogenesis of iron–sulfur clusters (Fe/S) via the ISC assembly system, which supports extramitochondrial CIA system function through iron export mediated by NEET proteins. Surplus Fe^2^⁺ can accumulate at voltage‐dependent anion channels (VDAC1/2), where it promotes ROS generation via the Fenton reaction. Excessive mitochondrial ROS drives membrane damage and contributes to ferroptosis induction. Together, these pathways establish mitochondria as both metabolic amplifiers and redox mediators of ferroptotic cell death.

Mitochondria play a pivotal role in supplying energy through ATP in cellular metabolism, maintaining Fe homeostasis, and regulating both catabolic and anabolic pathways (Figure 5). Given their critical involvement in oxidative stress and metabolic regulation, mitochondria exert regulatory effects on numerous forms of RCD [12]. The morphological alterations of mitochondria, including mitochondrial membrane condensation, reduced volume, decreased quantity, loss of cristae, and outer membrane rupture, constitute distinctive features of ferroptosis that set it apart from other forms of RCD [46]. It is generally believed that mitochondria play a pivotal role in cysteine deprivation‐induced ferroptosis, as the mitochondrial tricarboxylic acid (TCA) cycle and electron transport chain serve as primary sources of ROS, thereby promoting ferroptosis. However, this may not be the case in ferroptosis induced by GPX4 inhibition, as pharmacological inhibition or genetic ablation of GPX4 can still induce ferroptosis without intact mitochondrial function [75]. Mitochondrial dysfunction stands out as a prominent feature of acute kidney injury (AKI) and neurodegenerative diseases. Moreover, mitochondrial dysfunction accelerates the progression of those as mentioned above diseases [12, 76].

Mitochondria harbor an unstable Fe pool characterized by potent redox activity [77]. Under physiological conditions, the homeostasis of free Fe is tightly regulated by FtMt. Structurally akin to cytosolic FtH [78], FtMt possesses Fe/S enzyme and Fe‐binding activities akin to cytosolic ferritin [79]. Mitochondrial ferritin prevents the accumulation of mitochondrial ROS [80]; otherwise, there is a risk of detrimental effects on mitochondrial proteins, lipids, and DNA, impairing ATP production and triggering energy stress [81]. Downregulation of FtMt enhances the accumulation of free Fe within mitochondria, inevitably leading to mitochondrial ROS accumulation and ferroptosis [82].

The cytosolic free Fe influx into mitochondria primarily serves as substrate for synthesizing heme and Fe/S clusters. Fe/S clusters, acting as cofactors, participate in various biological processes including Fe metabolism, mitochondrial respiration, and redox balance [83]. The synthesis and export of Fe/S clusters involve multiple enzymatic steps, with one such synthesis protein, frataxin, being crucial for delivering Fe into the mitochondrial Fe/S synthesis pathway [84]. Interestingly, mitochondrial fragmentation is observed in fruit flies with frataxin mutations without alterations in mitochondrial fission and fusion proteins [85]. This may be attributed to Fe toxicity induced by frataxin loss leading to dysregulated sphingolipid synthesis [86].

The understanding of mitochondrial involvement in LPO is limited compared with Fe metabolism. Studies investigating mitochondrial events in ferroptosis show that ρ0 cells lacking mitochondrial DNA exhibit resistance to cell death, attributed to enhanced antioxidant enzyme expression. Additionally, accumulation of cellular ROS and oxidized lipids has been observed in SK‐Hep1 ρ+ cells with intact mitochondrial DNA upon treatment with erastin or RSL3, whereas this phenomenon is not observed in SK‐Hep1 ρ0 cells [87]. This underscores the critical role of mitochondrial LPO in ferroptosis. Lyamzaev et al. [88] demonstrated that using mitochondria‐targeted antioxidants SkQ1 and MitoTEMPO to prevent mitochondrial LPO can inhibit ferroptosis induced by erastin or GSH depletion. This suggests that mitochondrial oxidation may be an early feature of ferroptosis. It can be argued that mitochondria‐targeted antioxidants are proposed as a promising therapeutic approach alongside traditional Fe chelation therapy [46, 88].

The voltage‐dependent anion channels (VDACs), located on the outer mitochondrial membrane, regulate the passage of metabolites into and out of mitochondria, thus controlling crosstalk between mitochondria and the rest of the cell during oxidative stress. The three isoforms of VDAC—VDAC1, VDAC2, and VDAC3—govern the transport of ions and metabolites between the cytoplasm and mitochondria [89]. Consequently, the absence of VDAC2/3 disrupts mitochondrial membrane potential (ΔΨm) homeostasis, affecting mitochondrial activity. In this context, Yagoda et al. [90] demonstrated that targeting VDAC2/3 leads to alterations in ΔΨm, ultimately resulting in ferroptosis in cancer cells harboring RAS mutations. It is widely accepted that siRNA‐mediated knockdown of VDAC2/3 can attenuate erastin‐induced ferroptosis [91].

Initially, erastin was believed to activate VDAC2 and VDAC3 directly, increasing mitochondrial transmembrane potential [90]. Subsequently, VDAC2 was identified as a direct target of carbonylation (a lipid‐derived electrophilic modification of proteins) during RSL3‐induced ferroptosis [92]. Recently, it has been discovered that erastin induces the degradation of VDAC2 and VDAC3 in melanoma cells in a NEDD4 E3 ubiquitin ligase‐dependent manner [93]. These findings support the crucial role of VDAC in mediating mitochondrial damage, including mitochondrial ROS generation, during ferroptosis.

### 3.4 Cross‐Regulation of Signaling Pathways

**FIGURE 6:**
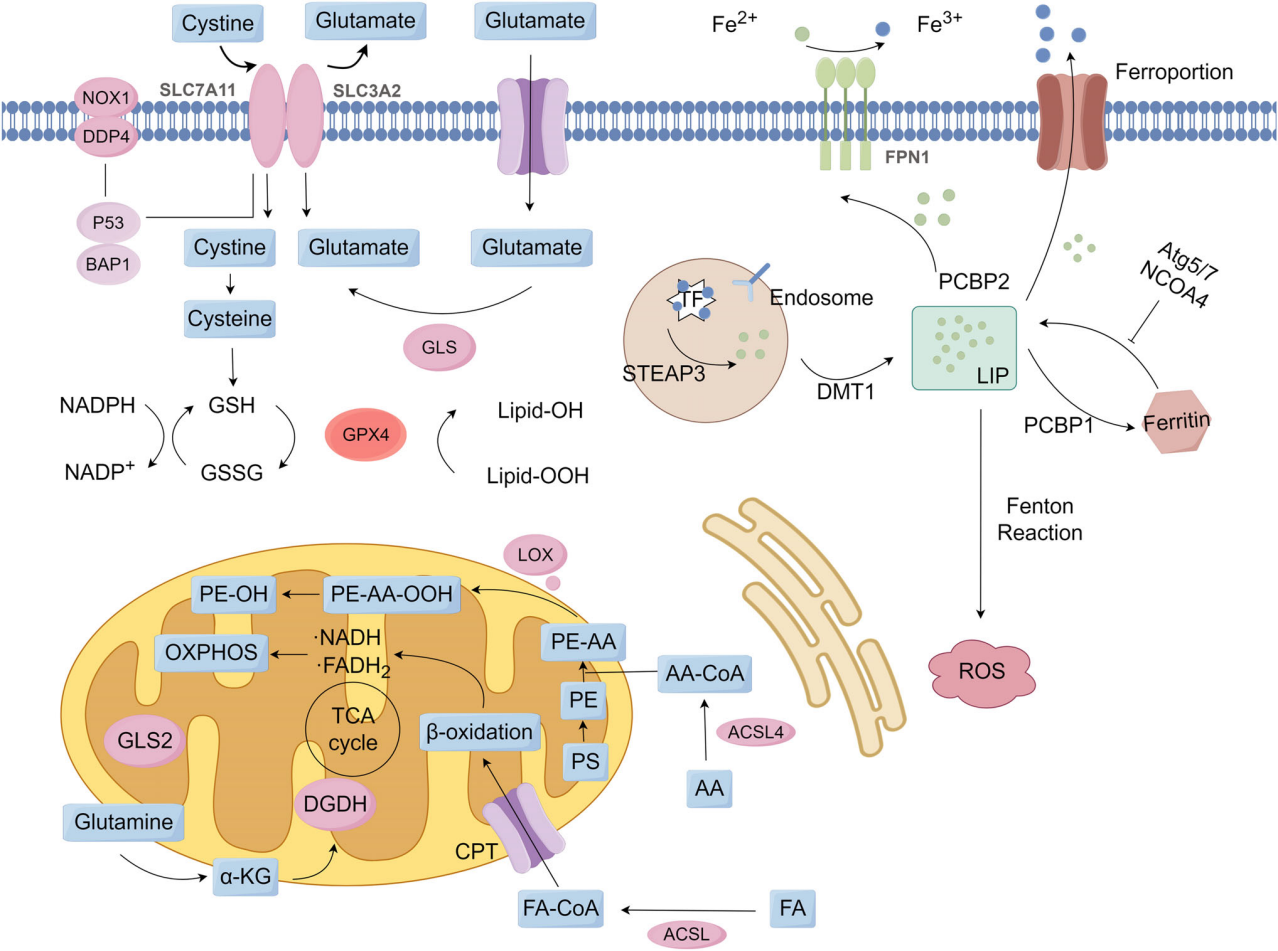
Regulatory mechanisms of ferroptosis. Ferroptosis is governed by a network of metabolic and redox‐regulatory pathways that converge on lipid peroxidation and iron homeostasis. Cystine is imported via the cystine/glutamate antiporter system Xc^−^ (composed of SLC7A11 and SLC3A2), where it is reduced to cysteine and used for glutathione (GSH) synthesis. GSH functions as a cofactor for glutathione peroxidase 4 (GPX4), which converts lipid hydroperoxides (Lipid‐OOH) to lipid alcohols (Lipid‐OH) and prevents ferroptotic cell death. This pathway is negatively regulated by p53, BAP1, and NOX1, while NADPH maintains GSH levels via glutathione reductase activity. Iron metabolism plays a central role in ferroptosis regulation. Transferrin (TF)‐bound Fe^3^⁺ is endocytosed and reduced by STEAP3, with Fe^2^⁺ released through DMT1 into the labile iron pool (LIP), stabilized by poly(rC)‐binding proteins PCBP1 and PCBP2. Ferritin sequesters excess iron and is degraded via NCOA4‐ or Atg5/7‐mediated ferritinophagy, promoting Fenton reaction‐mediated ROS generation. Mitochondrial and lipid metabolism contribute essential substrates for ferroptosis. Glutamine, via GLS2 and DGDH, supports the TCA cycle and oxidative phosphorylation (OXPHOS), enhancing ROS production. β‐Oxidation of fatty acids (FA), catalyzed by carnitine palmitoyltransferase (CPT), provides acetyl‐CoA and further augments electron transport. Arachidonic acid (AA) and other polyunsaturated fatty acids (PUFAs) are esterified into membrane phospholipids by ACSL4 and LPCAT3, forming PUFA‐phosphatidylethanolamines (PE‐AA), which are subsequently oxidized by lipoxygenases (LOX) into PE‐AA‐OOH—critical death signals in ferroptosis.

Beyond metabolic substrates and organelle dysfunction, ferroptosis is orchestrated by a network of signaling pathways that finely regulate redox homeostasis and cellular susceptibility to ferroptotic death. These pathways serve as molecular switches that integrate environmental and intracellular cues, modulating the execution or inhibition of ferroptosis. In this section, we focus on the major regulatory axes—including GSH/System Xc^−^/GPX4, ferroptosis suppressor protein 1 (FSP1)/CoQ/NAD(P)H, and acyl‐CoA synthetase long‐chain family member 4 (ACSL4)—that constitute the core defense and execution programs of ferroptosis (Figure 6).

#### 3.4.1 GSH/System Xc^−^/GPX4

The GSH/System Xc^−^/GPX4 pathway is a central regulator of ferroptosis, playing a crucial role in maintaining cellular redox balance and lipid homeostasis. System Xc^−^ is a widely expressed amino acid antiporter protein on the membrane [94], with its active subunit being the amino acid transporter protein SLC7A11 [95]. SLC7A11 mediates the ATP‐dependent exchange of extracellular cystine for intracellular glutamate, facilitating cystine uptake for GSH synthesis. Intracellular cysteine, derived from cystine reduction, dynamically regulates GSH synthesis. GSH, an endogenous antioxidant with a thiol group structure, exhibits significant antioxidant and free radical scavenging abilities.

Early studies on oxidative neuronal death highlighted the importance of this pathway. For instance, Himi et al. [96] discovered that inhibition of glutamate uptake in hippocampal neurons led to oxidative neuronal death. Subsequently, Lewerenz et al. [91] reported on the synergistic action of the glutamate transporter and System Xc^−^ in preventing glutamate oxidative toxicity.

Erastin, a classic inducer of ferroptosis, inhibits System Xc^−^ activity by binding to SLC7A11, thereby depleting GSH and inhibiting GPX4 activity, ultimately inducing ferroptosis [2]. Wang et al. [97] reported that activating transcription factor 3 (ATF3) promotes erastin‐induced ferroptosis by suppressing SLC7A11 expression in a p53‐independent manner through binding to the SLC7A11 promoter. Similarly, ethylmalonic encephalopathy (EC) triggers ferroptosis by depleting GSH, which inhibits SLC7A11 and GSH synthesis‐related protein GCLC, with nuclear factor erythroid 2‐related factor 2 (Nrf2) acting as an upstream determinant gene in this process. Ferroptosis inhibitors such as Fer‐1 and tBHQ can alleviate EC‐induced cellular damage and inflammation [98].

GPX4, a member of the GPX family, is a crucial regulator of ferroptosis [99]. Initially discovered as a selenoprotein through biochemical purification by Ursini and colleagues [100], GPX4 is the only known intracellular enzyme capable of reducing lipid hydroperoxides (PLOOH) to alcohols (PLOH) [49, 101]. This reduction, mediated by selenocysteine, interferes with the Fenton reaction, thereby reducing the accumulation of oxidative hydrogen peroxide, resisting oxidative damage, and inhibiting ferroptosis. The downregulation of GPX4 renders cells sensitive to ferroptosis, and the inhibition of GPX4 activity is a hallmark of this process. GSH acts as a cofactor for GPX4, maintaining redox homeostasis within cells; thus, GSH levels directly affect the reduction capacity of GPX4 [102].

GPX4 inhibitors such as RSL3, DPI7, and DPI10 block its activity pharmacologically [103, 104]. Autophagy is an essential regulatory mechanism for GPX4 [105], where creatine kinase B (CKB) is activated by the insulin‐like growth factor 1 receptor signaling pathway. As a protein kinase, CKB phosphorylates GPX4 to prevent its binding to HSC70, thereby inhibiting chaperone‐mediated autophagic degradation of GPX4, enhancing its stability, and resisting ferroptosis [106]. Copper also plays an unexpected role in GPX4 autophagy; exogenous copper binds directly to the selenocysteine of GPX4, leading to increased ubiquitination and aggregation of GPX4, followed by subsequent autophagic degradation. Copper chelators can mitigate ferroptosis induced by System Xc^−^/GPX4 dysfunction [107].

It is worth noting that RSL3 and ML162, though classically recognized as GPX4 inhibitors, have recently been shown to lack the ability to directly affect GPX4 enzyme activity [108]. Instead, they act as potent inhibitors of another selenoprotein, TXNRD1, indirectly inhibiting thioredoxin‐dependent enzymes and regulatory redox pathways. Despite this, their cytotoxic characteristics are markedly different from those of specific TXNRD1 inhibitors [109], suggesting that previous studies on RSL3 and other ferroptosis inducers (FINs) may require reassessment.

The GSH/System Xc^−^/GPX4 pathway is considered the main route of ferroptosis [49], underscoring the close connection between amino acid metabolism and ferroptosis. Research on specific regulatory pathways related to GSH/System Xc^−^/GPX4 could significantly advance our understanding of ferroptosis.

#### 3.4.2 FSP1/CoQ/NAD(P)H

The FSP1/CoQ/NAD(P)H pathway represents another GSH‐independent ferroptosis monitoring pathway parallel to the GSH/System Xc^−^/GPX4 pathway [110]. It acts in synergy with the GSH/GPX4 system to counteract LPO, with its core component being FSP1.

FSP1 functions as an endogenous ferroptosis inhibitor by interrupting cellular ferroptosis through its impact on ubiquinol metabolism [111]. FSP1 can provide protection against ferroptosis induced by GPX4 gene knockout. Conversely, depletion of FSP1 abolishes the resistance of tumor cells to FINs like RSL3, leading to a significant increase in cellular ferroptosis [112].

From a mechanistic standpoint, the FSP1/CoQ/NAD(P)H pathway is understood to initiate with the acetylation of the N‐terminus of the FSP1 protein at lysine [112]. This modification facilitates the recruitment of FSP1 to the mitochondrial membrane, where it utilizes NADH/NADPH as cofactors for CoQ10 redox cycling, thereby reducing CoQ10 to its active form. Subsequently, FSP1 shuttles the reducing equivalents across the membrane, generating lipophilic radical‐trapping antioxidants that inhibit LPO reactions. This process elucidates the function of coenzyme Q10 in suppressing ferroptosis [113].

Additionally, FSP1 serves as a vitamin K reductase, participating in the noncanonical vitamin K oxidation‐reduction cycle to inhibit ferroptosis. Vitamin K, a naphthoquinone with redox activity, shares similarities with ubiquinone [114].

Ferroptosis is considered a significant mechanism of radiation‐induced cell death, with radioresistant cells exhibiting tolerance to GPX4 inhibitors but increased sensitivity to FSP1‐mediated ferroptosis. This suggests a shift in the dependency mechanism toward ferroptosis in acquired radioresistant cells. The driving force behind this functional alteration is attributed to differences in upstream metabolite synthesis, specifically the upregulation of CoQ synthesis and downregulation of GSH synthesis [115]. This discovery provides potential strategies for reversing radioresistance and reveals possible regulatory mechanisms for monitoring two types of ferroptosis.

The methylated regulatory factor Uhrf1 has been identified as one of the targets of ferroptosis. Elevated Uhrf1 levels simultaneously increase the methylation levels of CpG sites in the promoters of both GPX4 and FSP1 genes, leading to their epigenetic suppression. This collaborative action accelerates cellular ferroptosis. Uhrf1 is thus identified as a common upstream regulator of GPX4 and FSP1 [116], providing insights into the epigenetic regulation of ferroptosis and potential therapeutic targets for overcoming radioresistance.

#### 3.4.3 Acyl‐CoA Synthetase Long‐Chain Family Member 4

PUFAs such as AAs are primary substrates for LPO in ferroptosis [101]. In contrast, MUFAs can counteract PUFAs. The synthesis and degradation of PUFAs and MUFAs are regulated by various enzymes involved in multiple metabolic processes [117], with ACSL4 and Lys phosphatidylcholine acyltransferase 3 (LPCAT3) playing pivotal roles in integrating PUFAs into cellular phospholipids, crucial for inducing ferroptosis [118]. Specifically, Gpx4/ACSL4 double‐knockout cells show unprecedented resistance to ferroptosis [56], and a decrease in ACSL4 expression is observed in cells depleted of GPX4 [119], suggesting a potential collaborative relationship between the two. Overactivation of ACSL4, LPCAT3, and LOXs, coupled with excessive accumulation of Fe 2+, leads to the excessive consumption of cell membrane phospholipids and accumulation of lipid peroxides, ultimately triggering intracellular ferroptosis [101].

LPO protein kinase C‐β II (PKCβII) has recently been identified as a crucial contributor to ferroptosis. PKCβII mediates the phosphorylation of ACSL4, activating it to trigger the generation and integration of PUFAs. Subsequently, the LPO level is amplified through a positive feedback signaling pathway, further inducing ferroptosis [120]. The PKCβII–ACSL4 axis plays a pivotal role in rapidly amplifying LPO signals to lethal levels, presenting targeted interventions in this pathway as potential strategies for therapeutics related to ferroptosis.

Liao et al. [121] reported how T cell‐derived interferon (IFN)γ and AA collectively activate ACSL4. This activation occurs through altering tumor cell lipid metabolism, enhancing the integration of AA with phospholipids containing C16 and C18 acyl chains, thereby promoting ACSL4‐dependent ferroptosis [121]. This is believed to represent the initiating mechanism of ferroptosis in tumor cells, with ACSL4 as a critical node in lipid metabolism pathways.

Interestingly, another subtype of the long‐chain ACSL family, ACSL3, is associated with sensitivity to ferroptosis. ACSL3 activation, dependent on MUFAs, substitutes and reduces the oxidative toxicity of PUFAs on the plasma membrane, thereby inhibiting cellular ferroptosis [60]. Moreover, ACSL3 plays a crucial role in lipid metabolism, particularly in forming and regulating neutral lipid LDs. Treatment with baicalein significantly reduces oxidative stress levels in HT22 cells and improves Fe storage, attenuating RSL3‐induced ferroptosis [122]. This is attributed to the activation of GPX4, inhibition of ACSL4 expression, and upregulation of ACSL3 expression [123].

### 3.5 Key Inducers and Inhibitors of Ferroptosis

Ferroptosis is regulated by various inducers and inhibitors that influence key cellular processes, particularly oxidative stress and Fe metabolism. Inducers such as Erastin and sulfasalazine inhibit the System Xc^−^ antiporter, leading to cysteine depletion and GSH deficiency, critical for ferroptosis induction [2, 124, 125, 126, 127, 128]. RSL3, ML162, and FIN56 inactivate GPX4, further depleting GSH and promoting LPO, a hallmark of ferroptosis [113, 129, 130]. Chemotherapeutic agents like Cisplatin also induce ferroptosis by reducing GSH levels and inactivating GPX4, while IFN‐γ triggers LPO through targeting SLC7A11 [131, 132].

Conversely, ferroptosis can be inhibited by targeting oxidative stress and Fe homeostasis pathways. HO‐1 enhances cellular Fe availability, mitigating ferroptosis [133]. Sulfasalazine blocks System Xc^−^, preventing cysteine deprivation and ferroptosis [134]. Inhibitors like ferrostatin‐1 and liproxstatin‐1 act by inhibiting LPO [47, 135], while Fe chelators such as DFO reduce intracellular Fe, limiting the Fe‐driven LPO that triggers ferroptosis [2]. Additionally, antioxidants like Vitamin E and Trolox help restrain LPO, protecting cells from ferroptosis [57, 136, 137]. These compounds underscore potential therapeutic strategies for modulating ferroptosis in both cancer and inflammatory diseases.

## 4 Ferroptosis and Inflammatory Diseases

Ferroptosis critically exacerbates tissue damage across diverse inflammatory pathologies by engaging with ROS and inflammatory signaling cascades. This nexus contributes to the pathophysiology of conditions including RA, cardiovascular disorders, and inflammatory bowel disease (IBD). We delineate key mechanistic interactions: the crosstalk between ferroptosis and inflammatory responses, emphasizing central signaling axes such as NF‐κB and the NLRP3 inflammasome; disease‐specific mechanisms wherein dysregulated Fe homeostasis and LPO propagate tissue damage; and therapeutic strategies targeting ferroptosis inhibition, with focus on emerging approaches combining immune modulation and antioxidant systems.

### 4.1 Ferroptosis in Inflammation

Inflammation, as a crucial defense mechanism against tissue damage and pathogen invasion, has been extensively demonstrated to correlate with tumorigenesis and progression [138]. Under physiological conditions, an intact immune surveillance system should possess the capability to distinguish sterile injury, microbial infection, and abnormal cellular proliferation, initiating precise immune responses through activation of specific cell death modalities [139]. Programmed apoptosis, as a classical form of cell death, achieves cellular clearance without inducing significant inflammatory responses. In contrast, necrosis‐induced membrane integrity disruption releases substantial intracellular components, which exacerbate inflammatory responses through activation of PRR signaling pathways [139]. Notably, ferroptosis may induce sterile inflammation through unique molecular mechanisms [139].

Emerging evidence indicates that DAMPs and alarmins released during ferroptosis can bind to specific receptors and activate proinflammatory signaling cascades. This bidirectional regulatory relationship between ferroptosis and inflammatory responses has been supported by multiple studies [140, 141]. At the molecular level, peroxidation of PUFAs constitutes the core feature of ferroptosis, a process precisely regulated by lipid metabolism networks [142, 143, 144]. Studies reveal that in erastin‐induced ferroptosis, cyclooxygenase (COX), cytochrome P450 (CYP450), and LOX are identified as key enzyme systems mediating LPO [142, 143]. When intracellular Fe homeostasis imbalance leads to ROS accumulation, abnormal LOX activation triggers chain peroxidation of PUFAs, ultimately resulting in membrane system collapse [142, 143]. Importantly, this pathological mechanism has been explicitly confirmed in Pseudomonas aeruginosa and Mycobacterium tuberculosis infection models [145].

The interaction between AA metabolic pathways and ferroptosis exhibits remarkable complexity. Experimental data demonstrate that ferroptosis significantly upregulates prostaglandin‐endoperoxide synthase 2 (PTGS2/COX2) expression, thereby accelerating AA metabolism and synthesis/release of proinflammatory mediators [146]. The GSH metabolic system effectively inhibits LOX activity by regulating cellular redox status, with GPX4 playing a critical cytoprotective role through elimination of lipid hydroperoxides [147]. Recent studies reveal that active AA intermediates released from ferroptotic cells promote transcellular eicosanoid biosynthesis, providing novel insights into spatiotemporal correlations between cell death and inflammation [148]. Furthermore, metabolites including HETEs, oxidized eicosanoids, and leukotriene family members (LTB4–LTE4) generated through LOX catalysis exhibit dual functions in modifying immune cell phenotypes and indirectly inducing ferroptosis [149]. Research by Dar's team [150] in P. aeruginosa infection models further confirms that LOX‐mediated phospholipid oxidation in pulmonary epithelial cells significantly promotes ferroptosis progression.

Regarding inflammatory regulation mechanisms, HMGB1, as a crucial DAMP molecule, amplifies inflammatory signaling cascades through PRR binding postrelease. Animal studies confirm that HMGB1 neutralization via specific antibodies effectively alleviates ferroptosis‐associated inflammation [151, 152]. Currently identified ferroptosis inhibitors include Fe chelators (ferrostatin‐1, DFO, deferiprone) and antioxidants (liproxstatin‐1, sulfasalazine), all demonstrating significant anti‐inflammatory properties across diverse disease models [153, 154, 155]. Notably, the neuroprotective effects of Fer‐1 in intracerebral hemorrhage and traumatic brain injury have been validated by multiple preclinical studies [156, 157]. The therapeutic potential of DFP in neurodegenerative diseases such as motor neuron disease and Parkinson's disease (PD) is gradually emerging [158].

Mechanistic analyses indicate these inhibitors primarily function through: (1) ROS generation inhibition; (2) upregulation of GSH/GPX4/xCT antioxidant systems; and (3) downregulation of proinflammatory cytokines including interleukin‐6 (IL‐6) and tumor necrosis factor‐α (TNF‐α). Intriguingly, certain inflammatory mediators like TNF‐α have been found to directly inhibit GPX4 activity, establishing a positive feedback loop between inflammation and ferroptosis [158]. In‐depth elucidation of ferroptosis–inflammation interaction networks will provide crucial theoretical foundations for developing novel anti‐inflammatory therapeutic strategies.

#### 4.1.1 Crosstalk among Ferroptosis, ROS, and Inflammation

Inflammatory regulation exhibits close associations with multiple RCD mechanisms. The novel concept of “necrotic inflammation response” proposed by recent studies specifically refers to pathological inflammatory responses triggered by necrotic cell death [159, 160]. The core mechanism involves the release of DAMPs, including ATP, HMGB1, histones, and nucleotides following plasma membrane integrity disruption. These molecules synergize with proinflammatory factors such as IL‐1α and IL‐33 to activate PRR‐mediated innate immune responses. Notably, persistent DAMP accumulation can establish a self‐reinforcing loop between inflammation and cell death, a positive feedback mechanism that plays critical roles in pathological processes of tissue damage and organ failure [159, 160]. Compared with the well‐characterized regulatory networks of necroptosis and pyroptosis, the inflammatory mechanisms associated with ferroptosis remain largely enigmatic. Emerging evidence demonstrates that ferroptotic cells release DAMPs through signaling axes such as RAGE–NF‐κB, participating in sterile inflammation and progression of various inflammatory diseases [160, 161].

Oxidative stress, as a crucial driver of inflammation, regulates expression profiles of inflammatory mediators including TNF‐α through activation of transcription factors such as Nrf2 and NF‐κB1 [162, 163]. Excessive ROS accumulation not only depletes intracellular antioxidant reserves but also perpetuates inflammatory cascades through LPO products. Experimental studies reveal that LPO derivatives induce low‐density lipoprotein modification and exacerbate inflammatory responses via macrophage phenotype polarization pathways. This molecular mechanism demonstrates universality across pathological processes of multiple diseases.

The GSH–GPX4 antioxidant system occupies a pivotal position in ferroptosis–inflammation crosstalk. As the primary redox buffering system, GSH maintains cellular homeostasis by neutralizing ROS, with its depletion being closely associated with inflammation‐related injuries [164]. In tumor models, functional inhibition of GPX4 induces upregulation of 12‐LOX and COX1 expression, thereby triggering characteristic inflammatory responses [165, 166]. Conversely, GPX4 activation exerts dual protective mechanisms: suppressing synthesis of AA metabolism‐related inflammatory mediators while blocking NF‐κB pathway activation and reducing ROS production [167]. AA, as a crucial signaling molecule, is released from membrane phospholipids under cytokine stimulation and metabolized by COX, LOX, and CYP450 enzyme systems to generate prostaglandins and leukotrienes with broad biological effects [168, 169]. Particularly noteworthy is the confirmed critical regulatory role of LOX catalytic product accumulation in ferroptosis progression [166].

Significant progress has been achieved in understanding Fe metabolism dysregulation and immune modulation. Intracellular Fe overload has been shown to promote M1 macrophage polarization, a phenomenon particularly prominent in liver fibrosis and steatohepatitis models [170, 171]. The novel Fe chelator DIBI demonstrates unique advantages in sepsis treatment by modulating inflammatory mediator levels to improve microcirculatory disorders [172, 173]. Animal studies confirm that Fe homeostasis imbalance disrupts M1/M2 macrophage equilibrium, driving inflammation‐fibrosis cascades in diseases such as nonalcoholic fatty liver disease [171]. These findings provide fresh perspectives for understanding dynamic interactions between ferroptosis and immune microenvironments.

#### 4.1.2 Inflammatory Signaling Pathways in Ferroptosis

##### 4.1.2.1 Janus Kinase–Signal Transducer and Activator of Transcription

The Janus kinase (JAK)–signal transducer and activator of transcription (STAT) signaling transduction pathway, as a crucial regulatory hub for cellular responses, plays pivotal roles in diverse biological processes [174, 175]. This pathway mediates dimerization of cytokine receptors such as ILs and IFNs, triggering phosphorylation cascades of JAK family members, subsequently activating STAT proteins to regulate transcriptional activity of downstream target genes [176, 177]. Notably, a suppressor of cytokine signaling (SOCS) protein‐mediated negative feedback mechanism exists in this pathway, effectively maintaining dynamic equilibrium of signal transduction [176].

In tumor immunoregulation, IFN‐γ significantly enhances tumor cell sensitivity to FINs through the JAK–STAT–IRF1 signaling axis. Mechanistic studies demonstrate that this cytokine specifically inhibits expression of the SLC3A2/SLC7A11 complex while regulating LPO processes through direct binding of STAT1 to the SLC7A11 promoter [178, 179]. Intriguingly, this pathway exhibits bidirectional regulatory effects in retinopathy, where hyperactivation of JAK1/2–STAT1 signaling induces GSH synthesis impairment, consequently triggering ferroptosis events associated with macular degeneration [179].

The STAT family, as effector molecules of the JAK–STAT pathway, demonstrates significant tissue specificity in ferroptosis regulation. The clinical anesthetic propofol exerts dual regulation on GPX4 and SLC7A11 expression via the miR‐125b‐5p/STAT3 axis, exhibiting proferroptotic effects in gastric cancer models while demonstrating Fe homeostasis protection in neuroblastoma [180, 181]. This cell‐type‐dependent effect may be closely associated with tumor metabolic reprogramming features [182]. Furthermore, natural products such as artesunate enhance diffuse large B‐cell lymphoma (DLBCL) cell sensitivity to ferroptosis through STAT3 signaling inhibition, providing novel therapeutic strategies [183].

Fe metabolism homeostasis networks show close interactions with the JAK–STAT pathway. The IL‐6–JAK2–STAT3 signaling axis has been identified as the core regulatory mechanism for hepcidin expression, providing molecular basis for auranofin and other drugs to improve Fe overload conditions [184, 185]. Notably, SOCS1 enhances cellular susceptibility to ferroptosis through regulation of the p53/SLC7A11 axis, while SOCS1 mimetic peptides demonstrate significant antioxidant stress effects in renal disease models [186, 187].

Although several JAK inhibitors have been approved for clinical use, their effects on ferroptosis progression require systematic evaluation [188]. Current research suggests that tissue specificity and spatiotemporal activation patterns of the JAK–STAT pathway may explain differential regulation of ferroptosis by various stimuli. Drug development targeting this pathway should prioritize investigating its crosstalk with redox homeostasis and Fe metabolism networks. Future studies should focus on resolving key clinical translation challenges, including drug selectivity, tissue targeting, and development of ferroptosis‐related biomarkers.

##### 4.1.2.2 Nuclear Factor‐κB

NF‐κB, as a central regulator of innate immune responses, has been extensively implicated in inflammatory regulation and cell fate determination since its discovery over three decades ago [189, 190, 191]. This signaling system comprises canonical and noncanonical activation pathways: the former relies on nuclear translocation of p50/p65 heterodimers to mediate gene transcription, while the latter operates through p52/RELB complexes for signal transduction [192]. Recent studies reveal complex bidirectional regulatory roles of this pathway in ferroptosis control.

In hepatocellular carcinoma models, leukemia inhibitory factor receptor deficiency enhances tumor cell resistance to ferroptosis by promoting SHP1–TRAF6 complex formation, augmenting K63‐linked ubiquitination modification, and activating NF‐κB signaling, ultimately upregulating lipocalin‐2 (LCN2) expression to significantly reduce extracellular Fe uptake capacity [193]. This regulatory mechanism provides novel therapeutic targets. Notably, the neuroprotective agent dimethyl fumarate demonstrates potent antiferroptotic effects in rat models of chronic cerebral ischemia by activating the NRF2 signaling axis to upregulate IκBα expression, concurrently suppressing NF‐κB activation and promoting antioxidant protein expression including heme oxygenase‐1 (HMOX1), NAD(P)H quinone dehydrogenase 1 (NQO1), and GPX4 [194]. The regulatory role of the noncanonical pathway has been validated in hepatotoxicity models, where NIK or IKKα knockout effectively alleviates acetaminophen‐induced LPO and hepatocyte death, exhibiting protection comparable to ferrostatin‐1 [192].

Mechanistic studies of FINs provide crucial insights into NF‐κB pathway regulation. In glioblastoma (GBM) models, RSL3 activates NF‐κB signaling through GPX4 inhibition, subsequently suppressing ATF4/xCT system expression to exacerbate LPO [195].The application of NF‐κB inhibitor BAY 11‐7082 significantly attenuates the antitumor efficacy of RSL3 in vivo, highlighting the critical role of this pathway in ferroptosis. Paradoxically, the FIN piericidin A exhibits NF‐κB inhibitory effects in macrophages, suggesting cell‐type‐specific regulatory mechanisms [196].

HMOX1, as a key ferroptosis regulator, forms intricate interaction networks with NF‐κB signaling. In TNF‐α‐stimulated endothelial cells, NF‐κB upregulates miR‐155 to suppress BTB and CNC homology 1 expression, thereby promoting NRF2‐mediated HMOX1 transcription [197]. Conversely, HMOX1 overexpression inhibits RelA phosphorylation at serine 276, blocking proinflammatory factor VCAM‐1 expression to establish a negative feedback loop [198]. This bidirectional regulation is corroborated in studies of galangin‐treated RA, where enhanced HMOX1 expression suppresses IKKβ/NF‐κB signaling to alleviate synovial cell inflammation [199]. Furthermore, ferritin components FTH and FTL have been found to participate in hepatocellular carcinoma apoptosis regulation and macrophage inflammatory response suppression, respectively, expanding the scope of the NF‐κB–Fe metabolism regulatory axis [200, 201].

As a central inflammatory mediator, NF‐κB pathway activation triggers complex cascade networks. Current evidence confirms its involvement not only in classical inflammatory responses but also in profound modulation of ferroptosis through oxidative stress and Fe metabolism regulation. Although pathway‐targeting agents demonstrate promising efficacy in experimental models, clinically available specific NF‐κB inhibitors remain scarce compared with JAK inhibitors. Future research should prioritize deciphering tissue‐specific regulatory mechanisms, developing spatiotemporally selective intervention strategies, and establishing ferroptosis‐related biomarker systems to advance clinical translation.

##### 4.1.2.3 Inflammasome

The inflammasome, as a crucial component of PRRs, can respond to pathogen‐associated molecular patterns and DAMPs to form multiprotein complexes [202]. Among these, the NLRP3 inflammasome holds central importance in innate immune regulation, with its activation process involving recruitment of the apoptosis‐associated speck‐like protein containing a CARD (ASC) adaptor protein and cleavage‐mediated activation of procaspase‐1 [203, 204]. Activated caspase‐1 not only mediates maturation and secretion of IL‐1β and IL‐18, but also cleaves gasdermin D (GSDMD) to form plasma membrane pores, triggering pyroptosis—a distinct form of PCD [204].

Recent studies have revealed close connections between inflammasome activation and ferroptosis. In age‐related macular degeneration models, abnormally elevated LCN2 promotes ferroptosis in retinal pigment epithelial cells by blocking autophagy‐related 4B (ATG4B)‐mediated autophagosome maturation, leading to ferrous Fe accumulation and activation of the cyclic GMP–AMP synthase (cGAS)‐stimulator of IFN genes 1 (STING1) signaling axis, ultimately driving NLRP3 inflammasome‐dependent ferroptosis [205]. Pulmonary hypertension research demonstrates that monocrotaline treatment enhances LPO through upregulation of Toll‐like receptor 4 (TLR4)/NLRP3 expression, while ferrostatin‐1 intervention effectively maintains GPX4 and ferritin heavy chain 1 (FTH1) levels, alleviating pulmonary vascular endothelial cell loss and right ventricular remodeling [206]. Notably, the NLRP1 inflammasome also regulates ferroptosis in trophoblasts, as its silencing restores GPX4 activity and reduces oxidative damage markers [207].

The interaction between LPO and inflammasome activation exhibits bidirectional regulatory characteristics. Octanal promotes NLRP3 activation through olfactory receptor 2‐mediated sensing mechanisms, exacerbating atherosclerosis progression, while coenzyme Q10 supplementation inhibits this process [208, 209]. Paradoxically, the LPO product 4‐HNE demonstrates protective effects by binding to NLRP3 structural domains to block NEK7 interaction, suppressing inflammasome activation in acute lung injury models [210]. This double‐edged sword effect suggests that LPO microenvironments may regulate inflammasome activity through distinct molecular mechanisms.

Cross‐regulation of cell death modalities has emerged as a research hotspot. Clinical pathological analyses reveal significant positive correlations between NLRP3 expression and ferroptosis markers PTGS2 and ACSL4 in atherosclerotic plaques [211]. GPX4 has been confirmed to possess dual regulatory functions: its deficiency not only exacerbates LPO but also promotes GSDMD‐dependent pyroptosis through caspase‐11 activation, significantly increasing mortality in sepsis models [212]. The natural compound wedelolactone provides novel therapeutic strategies by upregulating GPX4 expression to simultaneously inhibit ferroptosis and pyroptosis in acute pancreatitis models [213]. These findings indicate complex signaling crosstalk networks among different PCD pathways, and elucidation of their interactive mechanisms will provide theoretical foundations for precise regulation of cellular fate.

##### 4.1.2.4 Mitogen‐Activated Protein Kinase

The mitogen‐activated protein kinase (MAPK) family, as a central regulator of cellular signal transduction, participates in the precise modulation of inflammatory responses and oxidative stress through cascade phosphorylation reactions [214, 215]. Family members orchestrate complex signaling networks by phosphorylating downstream effectors or regulating transcription factor activity, playing pivotal roles in maintaining cellular homeostasis [215, 216]. For instance, p38α‐mediated activation of mitogen‐ and stress‐activated protein kinase 1/2 induces dual specificity phosphatase 1 expression, which subsequently exerts negative feedback inhibition on JNK–c‐Jun pathway activity, exemplifying the self‐regulatory nature of MAPK signaling networks.

Studies confirm significant interactions between MAPK pathways and ferroptosis. In neonatal rat hypoxic–ischemic models, activation of the TLR4–p38 axis not only promotes inflammatory cytokine release but also exacerbates neuronal ferroptosis by suppressing SLC7A11/GPX4 expression, with p38‐specific inhibitors demonstrating therapeutic reversibility [217]. Oxygen‐glucose deprivation experiments further reveal that TLR4–p38 activation induces neuronal ferroptosis through elevated MDA levels, a mechanism closely associated with enhanced mitochondrial oxidative stress [218]. The extracellular signal‐regulated kinase (ERK) signaling branch also critically regulates ferroptosis: cadmium telluride quantum dot exposure enhances ferritinophagy via the NRF2–ERK axis, leading to intracellular labile Fe accumulation and triggering macrophage ferroptosis [217]. Notably, loganin significantly alleviates cisplatin‐induced renal tubular cell ferroptosis through ERK phosphorylation inhibition, providing potential therapeutic targets for AKI [219].

Characteristic ferroptosis‐associated metabolic disturbances exhibit bidirectional regulation of MAPK pathways. In cyclophosphamide (CYP)‐induced muscle injury models, Fe overload aggravates oxidative stress by suppressing ERK1/2 and p38 phosphorylation, while taxifolin improves hepatocyte redox homeostasis through MAPK activity restoration [220, 221]. Antioxidants α‐lipoic acid and resveratrol demonstrate potent LPO inhibition in Fe overload‐induced renal injury and spinal cord ischemia–reperfusion models via p38 axis modulation [222, 223]. Coenzyme Q10 metabolism studies reveal that the Braf/MAPK inhibitor GDC‐0879 effectively mitigates coenzyme Q deficiency‐induced renal cell death by maintaining PUFA homeostasis [224, 225].

Current evidence indicates that MAPK‐ferroptosis interactions exhibit tissue specificity and pathological context dependency. Comprehensive elucidation of spatiotemporal regulatory mechanisms governing MAPK subtypes (p38, ERK, JNK) in ferroptosis, combined with single‐cell transcriptomics and phosphoproteomics approaches, will provide critical theoretical foundations for developing precision intervention strategies.

### 4.2 Disease‐Specific Mechanisms

Ferroptosis, through its unique mechanisms of Fe accumulation and LPO, plays a crucial role in the pathogenesis of various inflammatory diseases. Its involvement extends beyond mere cellular death to influence the broader inflammatory response, contributing to tissue damage and disease progression. In cardiovascular diseases, ferroptosis exacerbates endothelial dysfunction and myocardial injury, while in IBD, it promotes intestinal inflammation and epithelial cell death. The dysregulated Fe homeostasis and accumulation of ROS that characterize ferroptosis fuel the pathological cycle of inflammation and tissue degeneration. In this section, we will explore disease‐specific mechanisms of ferroptosis in conditions such as cardiovascular diseases, IBD, sepsis, and RA, highlighting the physiological and pathological significance of ferroptosis in these diseases and its potential as a therapeutic target.

#### 4.2.1 Ferroptosis in IBD

The characteristic biological markers of ferroptosis—including lipid peroxide accumulation, GSH depletion, GPX4 activity suppression, and Fe overload—have been conclusively validated in intestinal lesion tissues and disease models of IBD patients [226, 227]. This form of cell death plays a pivotal role in IBD pathogenesis by regulating intestinal epithelial cell homeostasis: abnormally elevated epithelial cell mortality leads to mucosal barrier disruption, impairing tissue repair processes. Such pathological alterations are closely associated with persistent progression of chronic intestinal inflammation, fibrosis, and carcinogenesis [228, 229]. Mechanistic studies reveal that ferroptosis drives IBD progression through a triple‐axis mechanism: toxic ROS accumulation, Fe metabolism disorder‐induced oxidative stress, and dysregulated LPO collectively trigger programmed epithelial cell death [230, 231]. Notably, aberrant expression of ferroptosis‐related genes in experimental colitis models significantly modifies disease progression, highlighting their potential therapeutic targeting value [232, 233].

Clinical specimen analyses demonstrate markedly elevated MDA and ferrous ion levels in colonic mucosa of colitis patients compared with controls. This metabolic dysregulation induces ferritin upregulation, creating a proinflammatory vicious cycle [234, 235, 236]. In ulcerative colitis (UC) pathogenesis, abnormal activation of endoplasmic reticulum stress signaling pathways has been identified as a crucial mediator of ferroptosis. Research indicates that phosphorylation of NF‐κB p65 in intestinal epithelial cells suppresses endoplasmic reticulum stress by regulating eukaryotic translation initiation factor 2α, thereby inhibiting ferroptosis and alleviating colitis symptoms [237]. Furthermore, aryl hydrocarbon receptor repressor deficiency increases colitis susceptibility by activating CYP1A1‐mediated oxidative stress, inducing ferroptosis in intestinal intraepithelial lymphocytes [238].

Integrated multiomics analyses unveil complex regulatory patterns of ferroptosis‐related gene networks in IBD. Key lipid/Fe metabolism regulators including ACSL4, GPX4, and LPCAT3 exhibit significant expression dysregulation in UC patients [238, 239, 240]. Systematic analysis of UC patient transcriptomic data identified 26 differentially expressed genes predominantly enriched in energy metabolism pathways. The protein–protein interaction network formed by hub genes such as IL6, PTGS2, and HIF1A provides novel insights into ferroptosis–inflammation crosstalk [240]. Clinical cohort studies confirm persistent overexpression of CD44 and MUC1 as ferroptosis‐associated biomarkers in UC patients, with expression levels positively correlating with disease severity, establishing molecular foundations for novel diagnostic tools [240].

Emerging evidence highlights the regulatory role of noncoding RNAs in ferroptosis networks. Large‐scale RNA sequencing in IBD patients identified six differentially expressed long noncoding RNAs associated with ferroptosis and immune regulation. Construction of lncRNA–miRNA–mRNA networks confirmed their involvement in IBD‐related abnormal immune responses through competitive endogenous RNA mechanisms [241]. These discoveries open new avenues for deciphering epigenetic regulatory mechanisms of ferroptosis in IBD.

#### 4.2.2 Ferroptosis in Sepsis

As a life‐threatening systemic inflammatory response syndrome triggered by infection, the pathological features of sepsis involve complex mechanisms including immune dysregulation, metabolic disturbances, and multiorgan dysfunction [242]. Recent studies have revealed the dual regulatory role of ferroptosis (an Fe‐dependent form of RCD) in sepsis progression. During pathogen invasion, ferroptosis in intestinal epithelial cells compromises mucosal barrier integrity and promotes gut microbiota translocation, while macrophages enhance intracellular pathogen clearance through ferroptosis pathways [243, 244]. Notably, T cell homeostasis maintenance depends on GPX4‐mediated inhibition of membrane LPO, with their ferroptosis significantly impairing adaptive immune responses [245]. This cell death modality exhibits differential effects across immune cell subsets, highlighting the critical importance of spatiotemporal regulation of ferroptosis for sepsis outcomes.

Myocardial dysfunction, a common sepsis complication, demonstrates close association with cardiomyocyte ferroptosis. Animal studies confirm that LPS stimulation significantly upregulates Fe metabolism‐related gene expression in myocardial tissue, while ferroptosis inhibitors effectively improve cardiac function parameters and prolong survival [246, 247]. Preclinical research indicates that dexmedetomidine (Dex) reduces sepsis‐associated atrial fibrillation incidence by modulating ferroportin activity to inhibit LPO accumulation in atrial tissue [248, 249]. Regarding respiratory complications, alveolar epithelial ferroptosis has been identified as a key pathological component of acute lung injury. Electroacupuncture at ST36 acupoint activates the cholinergic anti‐inflammatory pathway to regulate α7nAchR‐mediated ferroptosis suppression, offering a novel nonpharmacological intervention strategy for ARDS treatment [249].

Investigations of renal and central nervous system injuries reveal that the sepsis microenvironment induces ferroptosis through dual mechanisms: direct Fe overload‐induced LPO in renal tubular epithelium, and mitochondrial ROS burst‐amplified cell death signals via oxidative stress [250, 251]. Pharmacological interventions targeting the Nrf2 signaling pathway, such as Marin conjugated protein‐1 (which enhances antioxidant defense systems to alleviate renal injury) and ferrostatin‐1 (which blocks glutamate receptor overactivation‐induced neurotoxicity), show therapeutic potential [252, 253]. Hepatic injury studies demonstrate that irisin effectively mitigates sepsis‐associated hepatocyte ferroptosis by regulating GPX4 expression levels [254].

Current research not only clarifies the central role of ferroptosis in sepsis‐induced organ damage but also identifies multiple potential clinical intervention targets. Beyond classical ferroptosis regulators (GPX4, SLC7A11), emerging therapeutic directions include the FSP1‐mediated coenzyme Q10 metabolic pathway and mechanistic target of rapamycin (mTOR) signaling network. Notably, exosome‐derived noncoding RNAs epigenetically regulate ferroptosis progression, providing theoretical foundations for nucleic acid‐based drug development [255]. Future studies should integrate single‐cell sequencing and metabolomics technologies to decipher dynamic ferroptosis patterns across different sepsis stages, ultimately advancing personalized treatment strategies.

#### 4.2.3 Inflammatory Urinary System Disorders and Ferroptosis

##### 4.2.3.1 Acute Kidney Injury

AKI is characterized by a rapid decline in kidney function, often indicated by elevated serum creatinine (CRE) levels and reduced urine output [256]. It is a complex syndrome associated with a variety of physiological and pathological factors, encompassing loose collections of syndromes such as sepsis, cardiorenal syndrome, and urinary tract obstruction. The primary causes of AKI include inadequate renal perfusion, exposure to nephrotoxins, sepsis, or surgical trauma.

In response to harmful stimuli, renal tubular cells initiate an inflammatory cascade that leads to cell death. AKI is not only associated with numerous complications such as cardiovascular diseases and hepatorenal disorders but also has a high mortality rate exceeding 50%. Incomplete repair following AKI can result in renal fibrosis, which accelerates the progression to chronic kidney disease (CKD) and end‐stage renal disease (ESRD) [91, 257]. Additionally, there is a significantly increased risk of subsequent development of kidney cancer. The coexistence of multiple syndromes and the complexity of prognosis present challenges in the diagnosis and treatment of AKI.

AKI is intricately linked to various cellular processes such as apoptosis [258], necrosis [259], autophagy [260], and notably, inflammatory reactions. The inflammatory response is a pivotal component in the pathophysiology of AKI. Primary injury to renal tubular cells can trigger a secondary inflammatory response, which further amplifies the synchronous death of tubular cells and accelerates the deterioration of AKI [261, 262]. Various RCD pathways, including ferroptosis, necroptosis, and pyroptosis—which are known for releasing proinflammatory factors—are considered promising therapeutic targets for AKI. In particular, ferroptosis has been identified as an initiator in well‐defined nephrotoxic AKI models, leading to an inflammatory response and subsequent acceleration of other forms of cell death like necroptosis, which contribute to sustained renal dysfunction [263]. This underscores the critical role of ferroptosis in the progression of AKI.

Multiple lines of functional evidence and pathological models have demonstrated the efficacy of inhibiting ferroptosis in combating AKI [264, 265]. However, the exact mechanistic pathways through which ferroptosis influences AKI remain unclear, particularly in cases with varying etiologies.

##### 4.2.3.2 Ischemia–Reperfusion Injury‐AKI

Ischemia–reperfusion injury (IRI), induced by intermittent blood flow, typically involves severe cellular damage and death, accompanied by a robust oxidative stress response. Renal ischemia–reperfusion, a significant cause of AKI, exhibits pathophysiology characterized by mitochondrial dysfunction, cascading inflammatory reactions, and metabolic disruptions [256]. IRI is divided into two stages: the ischemic phase, marked by energy depletion and cellular apoptosis [266], and the reperfusion phase, where the accumulation of ROS induces Fe‐dependent cell death. This type of cell death is considered a novel factor driving IRI‐induced AKI.

Extensive research is underway to identify therapeutic targets for Fe‐dependent cell death in IRI‐induced AKI (IRI‐AKI).

Among these targets, ACSL4 is a prominent intervention site in Fe‐dependent cell death. Upregulation of ACSL4 is evident in the IRI‐AKI mouse model, while other vital pathways such as GPX4 and FSP1 show no significant responses. Notably, the knockout of ACSL4 results in a substantial alleviation of Fe‐dependent cell death and inflammatory reactions. This protective effect is further evidenced by the reduction in renal tissue lipid oxidation product MDA and blood CRE levels, highlighting the crucial role of ACSL4 in mitigating kidney pathological damage [267].

The anti‐inflammatory drug Dex has been shown to reverse the elevation of GSH levels in renal tissue. Moreover, Dex downregulates inflammatory responses and mitigates tissue damage caused by Fe‐dependent cell death, primarily by inhibiting ACSL4 expression [268].

The α2‐adrenergic receptor (α2‐AR) is a pivotal receptor mediating the signal for inhibiting Fe‐dependent cell death in this process. Consequently, inhibitors of α2‐AR counteract the protective effects of Dex, underscoring the significance of this receptor in the observed therapeutic outcomes.

In regenerative medicine, stem cell therapy utilizing human urine‐derived stem cell exosomes (USC‐Exo) provides additional supporting evidence. USC‐Exo is found to regulate ACSL4‐mediated Fe‐dependent cell death through interactions with RNA‐binding proteins [269]. Particularly noteworthy is the protective role of the long noncoding RNA TUG1 abundantly present in USC‐Exo.

In a study by Huang et al. [270], the growth‐promoting factor enhanced liver regenerator (ALR) has been explored for its role in inhibiting Fe‐dependent cell death, primarily through its interaction with ACSL4. Utilizing a mouse model of renal IRI, the study demonstrated overexpression of ALR and efficient clearance of ROS. The protective effect against Fe‐dependent cell death is attributed to the reduced accumulation of PUFAs in proximal tubular cells (HK‐2).

The multifaceted protective role of ALR in IRI‐AKI is emphasized. Previous research has shown the involvement of ALR in various pathways that influence Fe‐dependent cell death [271], including restoring mitochondrial dysfunction, promoting mitochondrial autophagy [272], and mediating Fe‐dependent cell death through the GSH–GPX4 system. ALR actively regulates GSH and Fe levels, thereby enhancing the antioxidant capacity of HK‐2 cells.

Further insights into the role of ALR come from experiments involving the inhibition of the system Xc^−^ with erastin, which exacerbates Fe‐dependent cell death. Concurrent silencing of ALR expression accentuates this effect, suggesting the participation of a novel signaling pathway in Fe‐dependent cell death orchestrated by ALR [273]. These findings contribute valuable information to our understanding of the intricate molecular mechanisms underlying the protective effects of ALR in the context of IRI‐AKI.

In a mouse model of IRI, the depletion of Legumain, an asparagine endopeptidase, has been linked to resistance against renal Fe‐dependent cell death. Chen et al. [105] postulate that Legumain contributes to the pathogenesis of AKI by regulating the lysosomal degradation of GPX4. Inhibiting Legumain proves effective in reducing lysosomal autophagy of GPX4, thereby providing relief from Fe‐dependent cell death. Notably, the absence of the E3 ubiquitin ligase TRIM21 mitigates kidney damage in the IRI mouse model.

The mechanistic insights reveal that TRIM21 exerts a negative regulatory role on GPX4 at the protein level through ubiquitination‐mediated degradation, involving the ubiquitin‐proteasome system [274]. The administration of the JAK2 inhibitor Fedratinib demonstrates the potential to reverse the expression levels of both TRIM21 and GPX4, thereby alleviating the damage caused by Fe‐dependent cell death.

Feng et al. [275] have confirmed that lysine‐specific demethylase 1 (LSD1) exacerbates renal IRI by activating the TLR4/NOX4 pathway. Notably, the inhibition of LSD1 has demonstrated effectiveness in alleviating Fe‐dependent cell death and oxidative stress induced by renal IRI [275]. This implies that LSD1 holds promise as a potential therapeutic target for mitigating complications associated with renal IRI, shedding light on a novel avenue for intervention in Fe‐dependent cell death and oxidative stress‐related renal injuries.

##### 4.2.3.3 Nephrotoxic Drugs

Nephrotoxic drugs, such as cisplatin, folate, and aristolochic acid, are significant factors in inducing AKI [99]. Cisplatin, widely utilized in cancer treatment, is associated with severe renal toxicity, which often results in complications of AKI, thereby limiting its clinical utility and garnering continuous attention. The pathophysiology of cisplatin‐induced AKI involves damage to proximal tubular epithelial cells (PTECs), oxidative stress, inflammation, and vascular injury [276].

Recent studies have explored the role of Fe‐dependent cell death in PTEC injury induced by cisplatin. Cisplatin disrupts lipid metabolism and the respiratory chain, intricately linking it to the accumulation of ROS and mitochondrial dysfunction [277]. Consequently, Fe‐dependent cell death emerges as a crucial target for mitigating cisplatin toxicity.

As early as 1998, it was discovered that DFO significantly alleviates cisplatin‐induced renal dysfunction and tissue damage in vivo [278]. Recent reports highlight arctigenin as a potential therapeutic agent, demonstrating its efficacy in modulating the System Xc^−^/GSH/GPX4 pathway and inhibiting disruptions in Fe metabolism, thereby mitigating cisplatin‐induced cell death in AKI. PD exhibits superior efficacy compared with traditional ferrostatin‐1 and DFO [279].

CD36, a scavenger receptor involved in countering lipid accumulation and oxidation in chronic kidney disease, has recently been implicated in a new mechanism during cisplatin‐induced AKI. Ma et al. [280] uncovered that CD36 directly interacts with FSP1, leading to its degradation through ubiquitination. This discovery introduces a potential novel therapeutic option for AKI.

Elevated concentrations of FAs can crystallize and accumulate in renal tubules, disrupting Fe homeostasis in the kidney. This disruption leads to Fe accumulation, oxidative stress, and ultimately triggers AKI [281, 282]. The use of ferroptosis inhibitors, such as Fer‐1 [264] and rutin [283], has shown efficacy in alleviating FA‐induced declines in renal function and reducing kidney damage. Xue et al. [284] reported that Krüppel‐like factor 15 (KLF15) mitigates folic acid‐induced AKI by activating the NRF2/GPX4 pathway, whereas the knockdown of KLF15 has the opposite effect. Additionally, Zhang et al. [285] demonstrated that distortion of histone deacetylase 3 (HDAC3) contributes to FA‐induced AKI in mice by suppressing GPX4, a suppression mediated by the joint regulation of GPX4 transcription by HDAC3 and KLF5.

##### 4.2.3.4 Rhabdomyolysis‐AKI

Rhabdomyolysis (RM) is a potentially life‐threatening clinical syndrome that encompasses a spectrum of conditions, with AKI being a prevalent complication. Approximately 10% of AKI cases are attributable to RM. Hemoglobin (Hb) and myoglobin (Mb), endogenous nephrotoxic substances released from cells during intravascular hemolysis or RM, play critical roles [279]. Mb‐mediated LPO in renal tubular epithelial cells (TECs) is closely associated with glutamate metabolism and can induce ferroptosis in proximal renal tubules [286], thereby triggering RM‐AKI. Research indicates that the accumulation of Mb in the kidneys is a significant cause of renal damage [287]. Excessive Mb enters the lysosomes of renal tubular cells, where it is broken down into globin and high levels of heme, which are further metabolized to produce substantial amounts of free Fe [288]. This accumulated Fe induces LPO through the Fenton reaction, leading to acute tubular injury [289].

Observations in murine RM‐AKI models include changes in renal MDA and 4‐HNE levels, along with increased Fe deposition [290]. Despite the lack of anticipated preventive effects from existing small molecule Fe chelators on RM‐AKI, Zu et al. [290] engineered and synthesized a novel hydroxyquinoline‐based Fe chelator targeting unstable Fe‐mediated ferroptosis in RM‐AKI. This new chelator exhibited notable efficacy and safety, balancing lipophilicity and hydrophilicity, demonstrating antioxidant properties, and upregulating hypoxia‐inducible factor 1α (HIF‐1α), providing auxiliary characteristics against Fe‐mediated cell death.

Fe deficiency has been identified as a factor that exacerbates RM‐induced AKI by amplifying nonheme Fe‐dependent Nox4 mechanisms. This regulation affects LPO and DNA oxidation, leading to upregulated p53/p21 activity and accelerating cellular aging [291]. These findings elucidate the synergistic impact of nutritional Fe deficiency on RM‐AKI, highlighting Hb as a potential primary catalytic Fe species in the kidneys during RM‐AKI.

##### 4.2.3.5 Sepsis‐AKI

Sepsis, a potentially lethal condition, arises from a dysregulated response to infection, leading to multiorgan damage or failure. Perturbations in innate immune responses, cascading inflammatory reactions, procoagulant and antifibrinolytic pathways, cellular metabolism, alterations in signal transduction, and acquired immune dysfunction are primary pathophysiological changes in the pathogenesis of sepsis [292]. Among critically ill patients, sepsis‐associated AKI (SA‐AKI) stands out as one of the most prevalent and severe complications [293, 294]. Current investigations into the Fe‐death nexus within SA‐AKI are still in their early stages.

Klotho, a protein family closely associated with aging, is primarily found in α‐, β‐, and γ‐isoforms [295]. α‐Klotho, often referred to as the “soluble” Klotho protein, serves as the main functional form circulating within the body, with notable expression in the kidneys (particularly in the distal and proximal renal tubules), as well as in blood, urine, and cerebrospinal fluid [296]. Zhou et al. [297] investigated the protective role of α‐Klotho in the kidneys afflicted with SA‐AKI. Their study revealed that Klotho mitigates the release of renal injury markers and inflammatory cytokines, diminishes oxidative stress, facilitates renal tissue histopathological changes, attenuates mitochondrial damage in SA‐AKI mouse renal TECs, enhances HK‐2 cell viability, and reduces the accumulation of ROS [297]. Importantly, emerging evidence suggests these protective effects are mechanistically linked to ferroptosis regulation. Klotho activates GPX4 through Nrf2 signaling pathway. It downregulates ACSL4 expression thereby limiting PUFA incorporation into membrane phospholipids. Klotho modulates Fe metabolism via upregulating FTH and reducing TFR1 expression. These coordinated actions establish α‐Klotho as a multifunctional regulator of ferroptosis in renal pathologies [297].

##### 4.2.3.6 Chronic Kidney Disease

CKD is characterized by progressive renal damage and a decline in renal function [298]. Early investigations have observed Fe accumulation within lysosomes of proximal renal tubules [299]. Fe deposition in the kidneys has been reported in both CKD patients and models of fibrosis‐induced cell injury, associated with features of cellular aging and various fibrotic diseases [300, 301]. Disruptions in Fe and lipid metabolism are noted across different forms of CKD.

Emerging evidence suggests that maladaptive renal responses to AKI contribute to the progression of CKD [91]. The kidneys play a crucial role in responding to oxidative stress stimuli, such as ischemia–reperfusion or nephrotoxicity, which influence cell death and repair processes. Persistent adverse conditions can lead to tubular atrophy, interstitial fibrosis, and glomerulosclerosis in renal cells [91], fostering the development of CKD and eventually leading to ESRD.

Fe‐dependent cell death, specifically ferroptosis, emerges as a significant factor in the transition from AKI to CKD, impacting various cellular physiological processes, including inflammation [302, 303], mitochondrial dysfunction [304], and the metabolism of renal TECs [305].

##### 4.2.3.7 Fibrosis

Fibrosis is the transformative process where damaged areas, responding to challenging repair stimuli, convert into fibrotic tissues characterized by fibroblasts and extracellular matrix accumulation. This process fundamentally signifies the persistence of unrepaired damage [306]. Renal fibrosis involves complex cellular processes including renal cell injury, inflammatory cell infiltration, activation of myofibroblasts, tubular atrophy, and microvascular rarefaction [307], making it a common pathological occurrence in various CKD [298].

Ferroptosis and fibrosis share metabolic pathways characterized by heightened glycolysis, excessive glutaminolysis, and increased fatty acid oxidation (FAO) [91]. A growing body of evidence underscores the pivotal role of ferroptosis in renal fibrosis [306, 308]. Studies have reported a reduction in GPX4 expression and an elevation of 4‐HNE in the renal TECs of CKD patients and in unilateral ureteral obstruction (UUO)/IRI mouse models. Inhibiting ferroptosis has shown significant efficacy in alleviating kidney injury, interstitial fibrosis, and the accumulation of inflammatory cells [309]. Treatment of UUO mice with the ferroptosis inhibitor liproxstatin‐1 led to suppressed downregulated GPX4 expression in renal tissues, resulting in reduced collagen deposition, attenuation of profibrotic factor secretion, and alleviation of myofibroblast activation [310]. Additionally, the Fe chelator DFO has been verified to mitigate ferroptosis and fibrosis in CKD rats [305].

Dai et al. [311], through the specific inhibition of ACSL4 using rosiglitazone, observed a significant reduction in ferroptosis and alleviation of interstitial fibrotic responses in renal TECs in mouse models subjected to TGF‐β, UUO, and FA exposure. This highlights the therapeutic potential of targeting ACSL4 in fibrosis treatment [312]. Building on the insights into the ACSL4 signaling pathway, Wang et al. [313] successfully ameliorated tubular injury, inflammation, and tubulointerstitial fibrosis in two CKD mouse models using the natural antioxidant quercetin, a flavonol. This intervention notably reduced ACSL4, COX2, and HMGB1 levels, while increasing GPX4, demonstrating the effectiveness of modulating this pathway in managing CKD‐associated fibrosis [313].

Furthermore, transcriptomics has provided profound molecular and cellular insights into the kidney fibrosis repair process. The adaptive/maladaptive kidney regeneration model suggests that pharmacological interventions targeting ferroptosis can guide cells toward adaptive repair, thereby improving fibrosis outcomes. This process involves the influx and activation of immune cells, and druggability screening has identified ferroptosis as a critical driver pathway for maladaptive repair, indicating potential therapeutic targets for enhancing kidney repair and function [314].

##### 4.2.3.8 Diabetic Nephropathy

Diabetic nephropathy (DN) is a significant cause of mortality and morbidity in patients with CKD, primarily involving factors such as hyperglycemia, oxidative stress, and inflammatory responses [315]. Studies have observed reductions in GPX4 and SLC7A11 in both streptozotocin‐induced DN mouse models and kidney biopsies from DN patients [316, 317], confirming the involvement of ferroptosis in DN.

Research has observed decreased levels of Nrf2 in DN models. Specific knockout of Nrf2 increases cellular sensitivity to ferroptosis under high glucose conditions, while upregulating Nrf2 effectively inhibits ferroptosis, thereby slowing down the progression of DN [318]. Pretreatment of db/db diabetic mice with Fer‐1 resulted in a significant decrease in HIF‐1α and HO‐1 [319], suggesting that ferroptosis may exacerbate tubular damage and worsen DN through the HIF‐1α/HO‐1 pathway.

##### 4.2.3.9 IgA Nephropathy

IgA nephropathy (IgAN) is an immune‐mediated chronic kidney disease and represents the most common form of primary glomerulonephritis [320]. Previous research has highlighted a complementary and reciprocal relationship between ferroptosis and Fe metabolism. In IgAN, disruptions in Fe metabolism exacerbate the progression of the disease [321, 322]. Recent studies have revealed that approximately 40% of IgAN patients exhibit Fe deposition in renal tissues [320]. Intriguingly, serum IgA levels are higher in IgAN patients with positive Fe deposition than in those without. Additionally, in patients with high levels of Fe deposition, levels of urinary protein excretion, serum CRE, blood urea nitrogen, and N‐acetyl‐β‐d‐glucosaminidase are elevated [321]. These findings suggest a close association between the amount of Fe deposited in renal tissues and the progression of IgAN, potentially serving as an early predictive factor for patients with this condition.

##### 4.2.3.10 Urolithiasis

Urolithiasis, characterized by the prevalent occurrence of stones in the urinary system, frequently involves calcium oxalate (CaOx) stones, which are among the most common types of kidney stones. These stones are associated with various kidney conditions such as AKI, renal fibrosis, and chronic kidney disease [323, 324]. High urinary oxalate levels are known to induce injury to renal TECs, playing a significant role in the formation of kidney stones through mechanisms including urine supersaturation, crystal nucleation, and adhesion [325].

**FIGURE 7:**
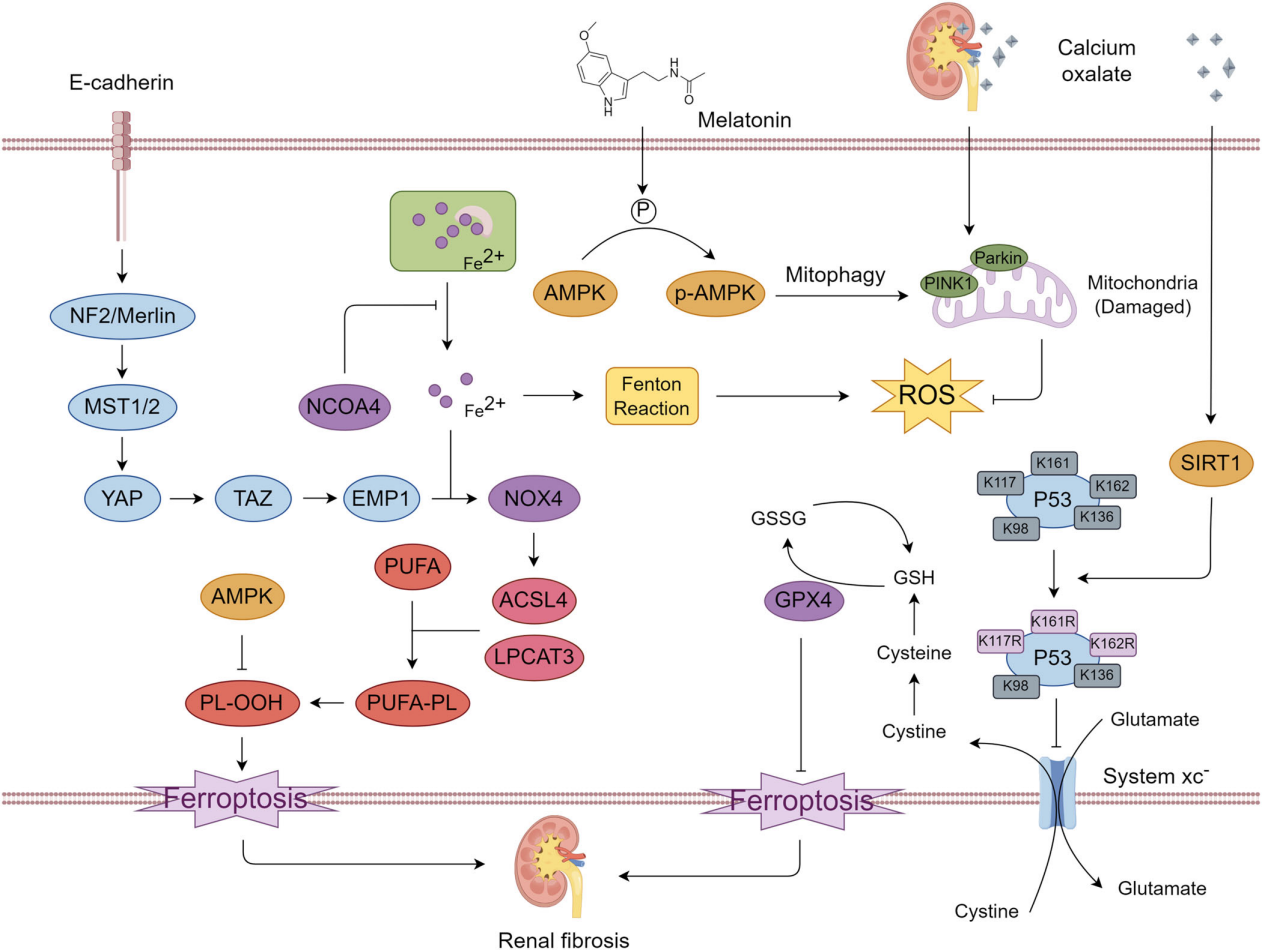
The mechanism of kidney fibrosis induced by ferroptosis in urolithiasis. Ferroptosis contributes to kidney fibrosis in urolithiasis through iron overload, lipid peroxidation, and disrupted antioxidant defense. Calcium oxalate crystals induce mitochondrial damage and mitophagy via the PINK1–Parkin axis, leading to reactive oxygen species (ROS) accumulation. ROS production is further amplified by Fe^2^⁺‐driven Fenton chemistry and nicotinamide adenine dinucleotide phosphate oxidase 4 (NOX4) activation. Labile Fe^2^⁺ is released through NCOA4‐mediated ferritin degradation, while arachidonic acid‐enriched phospholipids (PUFA‐PLs) are generated via acyl‐CoA synthetase long‐chain family member 4 (ACSL4) and lysophosphatidylcholine acyltransferase 3 (LPCAT3), forming lipid peroxides (PL‐OOH) under ROS exposure. Adenosine monophosphate‐activated protein kinase (AMPK) regulates this process by modulating polyunsaturated fatty acid (PUFA) metabolism and lipid oxidation. The Hippo signaling pathway, activated downstream of E‐cadherin–NF2–Merlin and MST1/2, suppresses ferroptosis via the YAP–TAZ–EMP1 axis. The cystine/glutamate antiporter system Xc^−^ imports cystine for glutathione (GSH) synthesis, which serves as a substrate for glutathione peroxidase 4 (GPX4) to detoxify lipid peroxides. Acetylation at multiple lysine residues (K98, K117, K136, K161, K162) modulates the ferroptosis‐sensitizing function of p53, while SIRT1 deacetylates p53 and promotes system Xc^−^ activity. Melatonin enhances AMPK phosphorylation, reduces Fe^2^⁺ levels and inhibits ferroptosis. Collectively, these pathways converge to promote ferroptosis‐associated tubular injury and renal fibrotic remodeling.

Early studies have identified that high concentrations of CaOx lead to oxidative stress and inflammatory responses in renal TECs [326]. An overload of CaOx disrupts mitochondrial homeostasis, as evidenced by impaired mitochondrial autophagy (mitophagy), diminished ATP production, and increased release of ROS. Melatonin has been shown to enhance mitochondrial autophagy through AMP‐activated protein kinase (AMPK) phosphorylation, regulate the PINK1–Parkin axis, limit ROS release, and reduce damage from ferroptosis, thus mitigating the deleterious effects associated with CaOx stone formation [327] (Figure 7).

He et al. [328] first examined the role of ferroptosis in the development of CaOx‐induced urolithiasis and observed that the expression of TF and TFRC in renal TECs increased under high concentrations of CaOx. Concurrently, the expression of SLC7A11 and GPX4 decreases, leading to cellular Fe^2+^ accumulation and LPO, which induces cellular ferroptosis. This damage is CaOx concentration dependent [328]. Activating p53 deacetylation to inhibit ferroptosis can effectively alleviate the degree of renal fibrosis in patients with kidney stones [324]. Additionally, Yes‐associated protein (YAP), a transcriptional coactivator of the Hippo pathway, translocate to the nucleus and activates ACSL4 in the tissues of patients with kidney stones. Silencing YAP inhibits ferroptosis by downregulating the expression of ACSL4, offering a beneficial approach to alleviating renal fibrosis caused by CaOx stones [329].

##### 4.2.3.11 Cystitis

Interstitial cystitis (IC), also known as chronic bladder pain syndrome, is a chronic nonbacterial cystitis often associated with immune reactions in bladder tissues, neurological abnormalities, or other contributing factors. Oxidative stress and inflammatory responses, attributed to factors such as aging or damage, are considered crucial in the pathogenesis of IC, leading to urothelial cell dysfunction and pain [282].

Mao et al.’s [330] study offers a detailed characterization of the involvement of ferroptosis in cystitis, particularly emphasizing its critical role in CYP‐induced hemorrhagic cystitis. The study identified alterations in ferroptosis markers, such as FPN1, SLC7A11, and GPX4, along with an upregulation of the LPO product MDA and the FA metabolism‐related enzyme ACSL4 [330]. To counteract LPS‐induced cystitis inflammation and ferroptosis, the bladder‐selective muscarinic receptor antagonist tolterodine was utilized. Mechanistically, the protective effect of tolterodine against ferroptosis is attributed to the modulation of the Nrf2–NF‐κB signaling pathway, which reduces Fe deposition and oxidative stress in human bladder epithelial cells. This study underscores the potential of ferroptosis as a significant area of focus in cystitis research [331], suggesting that therapeutic interventions targeting ferroptosis could be effectively developed for cystitis management.

##### 4.2.3.12 Prostate

Emerging evidence suggests that ferroptosis contributes to chronic prostatitis pathogenesis through Fe overload‐induced oxidative stress and inflammatory cascade amplification. Preclinical studies demonstrate that ferroptosis inhibitors like ferrostatin‐1 significantly attenuate prostate inflammation and fibrosis in experimental autoimmune prostatitis models, potentially via modulation of GPX4 and ACSL4 pathways [332]. However, the precise molecular mechanisms linking mitochondrial VDAC1 oligomerization to ferroptosis regulation in prostate epithelial cells remain poorly characterized, with current data primarily derived from cancer models [332, 333].

The therapeutic potential of Fe chelators and antioxidants in urological diseases warrants cautious interpretation. While DFO shows efficacy in reducing renal tubular ferroptosis in DN through HIF‐1α stabilization and Fe homeostasis restoration [334], direct evidence supporting its application in prostatic conditions remains sparse. Notably, DN studies reveal that ferroptosis contributes to renal damage through Nrf2/HO‐1 signaling dysregulation and endoplasmic reticulum stress‐mediated epithelial–mesenchymal transition (EMT) [335, 336]. These findings suggest potential mechanistic parallels between renal and prostatic pathologies that merit further investigation.

**FIGURE 8:**
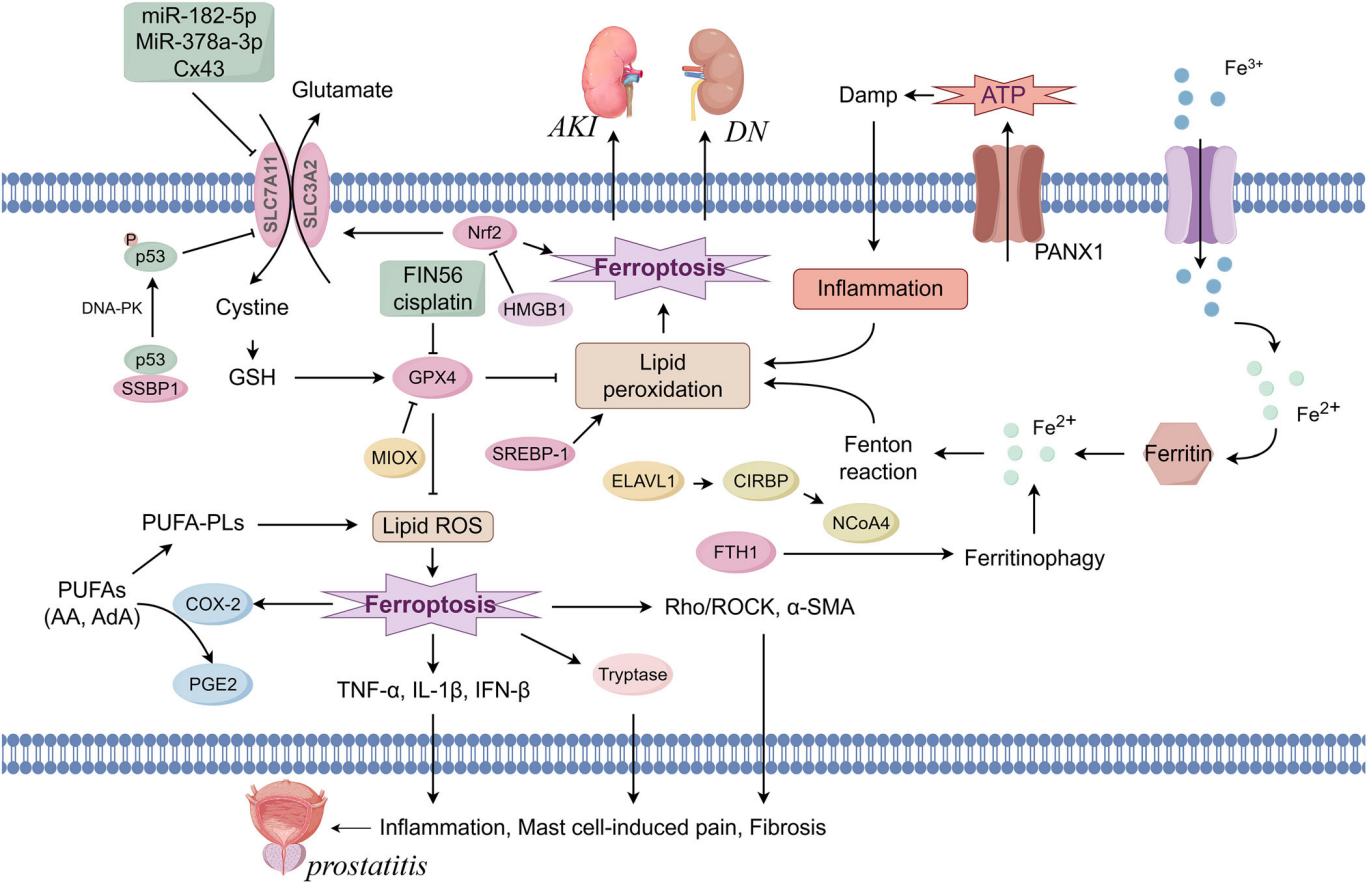
The regulatory roles of ferroptosis in AKI, DN, and prostatitis. Ferroptosis contributes to the pathogenesis of acute kidney injury (AKI), diabetic nephropathy (DN), and chronic prostatitis. In renal epithelial cells, glutamate export and cystine import via the cystine/glutamate antiporter system Xc^−^, composed of SLC7A11 and SLC3A2, are inhibited by tumor protein p53, DNA‐dependent protein kinase (DNA‐PK), miR‐182‐5p, miR‐378a‐3p, and connexin 43 (Cx43), leading to reduced glutathione (GSH) synthesis and impaired activity of glutathione peroxidase 4 (GPX4). GPX4 inhibition by FIN56 or cisplatin allows lipid hydroperoxides to accumulate, initiating ferroptosis. PUFA‐containing phospholipids are peroxidized into lipid ROS, a process amplified by cyclooxygenase‐2 (COX‐2)‐mediated prostaglandin E2 (PGE2) production. Intracellular Fe^2^⁺, released through NCOA4‐mediated ferritin degradation (ferritinophagy), undergoes Fenton reactions that exacerbate lipid peroxidation. Proteins including myo‐inositol oxygenase (MIOX), sterol regulatory element‐binding protein 1 (SREBP1), ELAV‐like protein 1 (ELAVL1), cold‐inducible RNA‐binding protein (CIRBP), and ferritin heavy chain 1 (FTH1) further modulate ferroptosis sensitivity. The transcription factor nuclear factor erythroid 2‐related factor 2 (Nrf2) counteracts ferroptosis by upregulating antioxidant defense. In the context of AKI and DN, ferroptosis leads to the release of high‐mobility group box 1 (HMGB1) and adenosine triphosphate (ATP), which exits via pannexin 1 (PANX1) channels and triggers inflammation. In prostate tissue, ferroptosis‐associated signals promote inflammation, pain, and fibrosis through the Rho‐associated protein kinase (ROCK) pathway, α‐smooth muscle actin (α‐SMA) expression, and mast cell‐derived tryptase.

Critical knowledge gaps persist in understanding cell‐type specific ferroptosis responses and microenvironmental influences. Recent work highlights tumor‐associate capacity of macrophage to modulate prostate cancer ferroptosis resistance through taurine metabolism [337], underscoring the need to explore stromal–epithelial crosstalk in benign prostatic diseases. The discovery of novel biomarkers like CSPP1, which regulates ferroptosis susceptibility across multiple cancers [338], opens new avenues for diagnostic and therapeutic development. Future research should prioritize human tissue validation of preclinical findings and address sexual dimorphism in ferroptosis regulation observed in metabolic disorders [339]. Figure 8 provides a comparative analysis of the specific roles of ferroptosis in AKI, DN, and prostatitis.

#### 4.2.4 Role of Ferroptosis in Inflammation and Tissue Damage in RA

Recent studies have revealed significant associations between characteristic molecular events of ferroptosis—including Fe homeostasis dysregulation, exacerbated LPO, GSH metabolism abnormalities, and GPX4 functional inactivation—and the pathological progression of RA [7, 340, 341]. Experimental evidence demonstrates that RA‐specific immune dysregulation and gut microbiota dysbiosis both interact with ferroptosis regulatory networks, indicating the crucial role of this cell death modality in RA pathogenesis. Notably, pharmacological modulation of ferroptosis exhibits therapeutic potential: FINs selectively eliminate hyperproliferative fibroblast‐like synoviocytes (FLSs), while ferroptosis inhibitors effectively alleviate joint inflammation and bone destruction. However, due to the complexity of ferroptosis molecular mechanisms and patient heterogeneity, precise therapeutic targeting of this pathway remains exploratory [341].

Mechanistically, groundbreaking research confirms that glycine significantly promotes RA‐FLSs ferroptosis through S‐adenosylmethionine‐dependent GPX4 promoter methylation pathways coupled with FTH1 downregulation, providing theoretical support for epigenetic‐based therapeutic strategies [341]. Another study reveals that bioactive peptide G1dP3 induces FLSs ferroptosis via the p53/SLC7A11 signaling axis, with its mechanism closely linked to somatic mutations in the p53 gene—a molecular basis for synovial hyperplasia and pannus formation in RA [342, 343, 344]. Advanced investigations demonstrate the dual regulatory role of p53 in ferroptosis: directly inducing ferroptosis by suppressing SLC7A11 activity, while indirectly influencing cell death through CDKN1A expression or DPP4 activity modulation [345, 346]. Intriguingly, the clinical drug sulfasalazine exhibits bidirectional effects: inhibiting ferroptosis via Xc^−^ system‐mediated GSH reduction while simultaneously promoting lipid ROS generation through Fe overload‐induced Fenton reactions, ultimately exacerbating ferroptosis [347].

Significant breakthroughs have emerged in antioxidant therapy research. Coenzyme Q10, functioning as a ROS scavenger, demonstrates remarkable anti‐inflammatory and antiferroptosis effects in collagen‐induced arthritis (CIA) models by suppressing Th17 cell differentiation and IL‐17 production through STAT3 signaling [348]. Plant‐derived polyphenols such as resveratrol and EGCG exert multiprotective effects in maintaining chondrocyte homeostasis through SIRT1/NF‐κB pathway activation or ERK/MAPK signaling cascade regulation [348, 349, 350]. Particularly noteworthy, the novel mechanism of icariin (a traditional Chinese medicine compound) in inhibiting synovial cell ferroptosis via the Xc^−^/GPX4 axis provides a paradigm for modernization research in herbal medicine [351].

In novel therapeutic target exploration, the regulatory role of TRPM7 ion channels has garnered substantial attention. Experimental confirmation shows abnormal overexpression of this channel in adjuvant‐induced arthritis model articular cartilage, with gene silencing significantly alleviating cartilage destruction and ferroptosis progression through PKCα–NOX4 signaling axis suppression [352, 353]. Furthermore, natural compounds like glycyrrhizin exhibit multitarget therapeutic effects via MAPK signaling inhibition and angiogenesis regulation, offering new perspectives for developing integrative RA treatment strategies [354]. These groundbreaking advances systematically elucidate the complex ferroptosis regulatory network in RA, laying a theoretical foundation for future precision medicine interventions.

### 4.3 Clinical Therapy of Targeting Ferroptosis in Inflammatory Diseases

Clinical therapeutic strategies targeting ferroptosis for inflammatory diseases are emerging as a research hotspot in translational medicine (Table 2). Ferroptosis exacerbates inflammatory damage through Fe‐dependent LPO and redox imbalance, a mechanism particularly prominent in IBD and RA [355]. For instance, Fe overload and GSH depletion in intestinal epithelial cells of IBD patients enhance ferroptosis susceptibility, while ferroptosis inhibition or Fe metabolism modulation can alleviate mucosal barrier disruption and aberrant immune responses [356]. Currently, Fe chelators such as DFO and epigallocatechin gallate have entered clinical trials, demonstrating oxidative stress and inflammation mitigation through intracellular free Fe reduction [357]. Additionally, antioxidants like Nrf2 activators exhibit protective effects against inflammatory neurodegenerative diseases in animal models by upregulating GSH synthesis and Fe homeostasis‐related genes [358].

**TABLE 2:** Advances in targeting ferroptosis for inflammatory disease treatment.

Category	Items	Diseases	Mechanism	References
Ferroptosis detection	Lipid droplet	Myocardial I/R injury	Lipid droplets (LDs), intracellular organelles for storing neutral lipids, influence cellular sensitivity to ferroptosis through the balance between LD degradation and storage. TPABTBP, an aggregation‐induced emission probe with high LD specificity and photostability, is suitable for imaging dynamic LD changes during ferroptosis.	[360]
	Mitochondrial viscosity	Myocardial I/R injury	Mitochondrial viscosity, affecting protein–protein interactions on mitochondrial membranes, is linked to multiple diseases and increases during mitoferroptosis. The fluorescent probe PPAC‐C4 enables ultraprecise quantification of mitochondrial viscosity by conjugating a mitochondria‐targeting cationic fragment to a vibration‐based fluorescent scaffold. Mito‐3, containing a cationic quinoline unit and a C12 chain, monitors intracellular mitochondrial viscosity changes via near‐infrared fluorescence. The probe CBS, based on electrostatic force and cytoskeletal protein molecular docking, enables stable and accurate detection of mitochondrial viscosity.	[361, 362]
	Hydrogen polysulfides (H₂Sn) and sulfur dioxide (SO₂)	Myocardial I/R injury	Metabolites of cysteine and glutathione, such as polysulfide (H₂S_n_) and sulfur dioxide (SO₂), are closely associated with ferroptosis. The nanosensor UCNP@mSiO₂@SP‐NP‐NAP, encapsulating a photoresponsive dye (SP‐NP‐NAP) on nanoparticles, detects H₂S_n_ and SO₂ in ferroptotic cells under visible and near‐infrared light excitation.	[363]
	Transferrin receptor 1 (TFR1)	Myocardial I/R injury	Transferrin receptor 1 (TFR1) on the cell membrane binds to serum transferrin to promote iron uptake, making it a promising biomarker for ferroptosis. The multimodal imaging platform based on superparamagnetic cubic iron oxide nanoparticles, using the probe SCIO–ICG–CRT–CPPs NPs, specifically detects intracellular TFR1 levels. CPPs facilitate tissue penetration, CRT binds to TFR1 via a nonclassical ligand‐guided mechanism, ICG enables optical imaging, and SCIO NPs allow MPI/MRI imaging.	[364]
Ferroptosis therapeutic targets	Transient receptor potential mucolipin 1 (TRPML1)	Cancer	In AKT‐hyperactivated cancer cells, AKT directly phosphorylates TRPML1 at Ser343, inhibits K552 ubiquitination‐mediated degradation of TRPML1, promotes ferroptosis, and enhances sensitivity to radiotherapy and immunotherapy.	[365]
	Fatty acid desaturases 1 and 2 (FADS1/2)	Triple‐negative breast cancer (TNBC)	FADS1/2 regulate lipid metabolism and ferroptosis sensitivity in triple‐negative breast cancer cells, serving as potential targets for cancer therapy.	[366]
	Migration inhibitory factor (MIF)	Cancer	MIF promotes DNA homologous recombination by activating breast cancer type 1 susceptibility protein, leading to cancer cell resistance to ferroptosis.	[367]
	Histone deacetylase 3 (HDAC3)	Liver injury	HDAC3 inhibition increases YAP nuclear translocation via the Hippo pathway and participates in iron overload‐induced ferroptosis in liver injury by altering hepcidin levels.	[368]
	Histone deacetylase 1/2 (HDAC1/2)	Intracerebral hemorrhage (ICH)	HDAC1/2 inhibition alleviates neuroferroptosis by modulating microglial heterogeneity, a mechanism linked to the Nrf2/HO‐1 pathway.	[369]
	Transient receptor potential melastatin 2 (TRPM2)	Liver I/R injury	TRPM2 induces mitochondrial lipid peroxidation via increased ALOX12 expression, contributing to hepatic ischemia/reperfusion (I/R) injury.	[370]
	Mg^2^⁺/Mn^2^⁺ dependent phosphatase 1K (PPM1K)	Cerebral I/R injury	Branched‐chain amino acids (BCAAs) induce significant changes in neuronal ferroptosis‐related genes and proteins and increase lipid ROS levels. PPM1K inhibits cellular ferroptosis by dephosphorylating the BCAAs dehydrogenase E1α subunit to promote BCAA degradation.	[371]
	Elabela–apelin receptor axis	Ischemic stroke	Upon binding to receptor APJ, elabela activates the Nrf2/ARE antioxidant signaling pathway, downregulates ACSL4 and ALOX15 expression, upregulates GPX4 and xCT expression, and reduces ferroptosis.	[372]
	lncRNA SEMA5A–IT1	Myocardial I/R injury	After being internalized by cardiomyocytes, lncRNA SEMA5A–IT1 in serum‐derived extracellular vesicles regulates BCL2 and SLC7A11 expression by sponging miR‐143‐3p, thereby inhibiting ferroptosis.	[373]
	LncRNA WAC–AS1	Renal I/R injury	Transcription factor BACH2 represses GPX4 and SLC7A11 transcription. Upon ferroptosis in renal tubular epithelial cells, lncRNA VEC–AS1 in exosomes secreted by damaged cells induces ferroptosis in neighboring cells by upregulating d‐fructose‐6‐phosphate amidotransferase 1 expression and inhibiting BACH2 ubiquitination‐mediated degradation. This process allows ferroptosis to spread widely in the renal tissue microenvironment as a “ferroptotic wave.”	[374]
Ferroptosis therapeutic strategies	GalNAc ligand‐coupled TMPRSS6–siRNA (SLN124)	β‐Thalassemia	Hepcidin is negatively regulated by TMPRSS6. Trimeric GalNAc‐conjugated TMPRSS6–siRNA (SLN124) enables effective hepatic‐targeted delivery of oligonucleotides, regulates hepcidin expression, and alleviates hepatic iron overload.	[375]
	Triphenylphosphine modified quercetin derived smart nanoparticle (TQCN)	Alzheimer's disease (AD)	TQCN effectively chelates iron through quercetin‐mediated spontaneous coordination and in situ self‐assembly of metal‐phenolic nanocomplexes, reducing iron overload and induced free radical bursts to ameliorate neuronal ferroptosis. Additionally, TQCN activates the Nrf2 endogenous defense system.	[376]
	TPA@Laponite hydrogel	Spinal cord injury (SCI)	The hydrogel TPA@Laponite exhibits shear‐thinning properties, transitioning from a gel to a flowable state under shear stress. With strong ROS‐scavenging capacity, TPA@Laponite inhibits ferroptosis by regulating iron metabolism. Introducing dental pulp stem cells into TPA@Laponite hydrogel alleviates muscle spasm and promotes spinal cord injury recovery by adjusting the ratio of excitatory and inhibitory synapses.	[377]
	mPEG–b‐Lys–BECI–TCO	Spinal cord injury (SCI)	Mesenchymal stem cells (MSCs) conjugated with fer‐1 to form nanoparticles mPEG–b‐Lys–BECI–TCO enable MSC‐mediated mitochondrial transfer to restore neuronal mitochondrial pools. MSC–Fer combination therapy inhibits ferroptosis and improves the inflammatory microenvironment after spinal cord injury.	[377]
	Idebenone micelles	Cerebral I/R injury	Idebenone, structurally similar to coenzyme Q10, exhibits potent antioxidant activity. CREKA peptide‐modified idebenone‐crosslinked micelles accumulate in ischemic brain tissue by binding to microthrombi in ipsilateral microvessels. Under ROS stimulation, diselenate in the micelles converts to hydrophilic selenite, achieving dual effects of ROS depletion and idebenone release to prevent neuronal ferroptosis.	[378]
	pH/GSH‐supported polyamino acid nanogel (NG/EDA)	Cerebral I/R injury	NG/EDA penetrates the blood–brain barrier and accumulates in rat brain ischemic injury sites. The acidic, high‐glutathione microenvironment activates NG/EDA, enabling selective and sustained release of edaravone to inhibit ferroptosis.	[379]
	Liproxstatin‐1@GluAC4A	Ischemic stroke	Conjugation of GluAC4A with liproxstatin‐1 (liproxstatin‐1@GluAC4A) significantly enhances liproxstatin‐1 solubility and release in hypoxic sites and reduces ferroptosis induced by rtPA treatment.	[380]
	M2pep–ADSC–Exos	Ischemic stroke	M2‐type microglia‐targeted stem cell‐derived adipocyte exosomes (M2pep–ADSC–Exos) contain Fxr2, which regulates ATF3 and SLC7A11 expression to reduce M2 microglia sensitivity to ferroptosis.	[381]
	N‐Cu5.4O@DFO NPs	Renal I/R injury	Neutrophil membrane‐coated copper‐based nanoparticles (N‐Cu5.4O@DFO NPs) exhibit high biocompatibility and stability, removing excess iron, reducing oxidative damage, and inhibiting ferroptosis.	[382]
	PEG–PDA@rutin NPs (PPR NPs)	Renal I/R injury	Rutin‐loaded polydopamine nanoparticles (PEG–PDA@rutin NPs, PPR NPs) accelerate ROS‐responsive rutin release and possess strong ROS‐scavenging ability, effectively entering cells to reduce Fe^2^⁺ deposition, lipid peroxidation, repair mitochondrial damage, and inhibit ferroptosis.	[383]
	PDN@AGL	Intestinal I/R injury	Amphiphilic molecules DTPA–N10‐10 and mPEG–TK–DA self‐assemble with apigenin‐7‐O‐glucoside (AGL) via hydrophilic interactions to form PDN@AGL, a multisite ROS‐scavenging nanoparticle. PDN@AGL inhibits ferroptosis by reducing ROS levels and lipid peroxidation and regulating the ATF3/SLC7A11 pathway.	[384]
	Polyphenol‐based nanomedics (ES NDs)	Osteoarthritis (OA)	Polyphenol‐based nanomedicines (ESND) synthesized via the Mannich condensation of selenomethionine and epigallocatechin gallate effectively reduce abnormal Fe^2^⁺ accumulation, GPX4 inactivation, and lipid peroxidation in chondrocytes, ameliorating ferroptosis‐induced metabolic disorders in chondrocytes.	[385]
	Cit–AuNRs@Anti‐TRPV1	Osteoarthritis (OA)	Citrate‐stabilized gold nanorods conjugated with TRPV1 monoclonal antibody (Cit–AuNRs@Anti‐TRPV1) act as photothermal switches to activate transient receptor potential vanilloid 1 (TRPV1) in chondrocytes under near‐infrared irradiation. Intracartilaginous injection of Cit–AuNRs@Anti‐TRPV1 upregulates GPX4 via TRPV1 activation to inhibit chondrocyte ferroptosis.	[386]
	tFNA–cur	Diabetic osteoporosis	Tetrahedral framework nucleic acid (tFNA)‐encapsulated curcumin (tFNA–cur) delivers curcumin to bone marrow, activates the Nrf2/GPX4 pathway, enhances mitochondrial function, and inhibits ferroptosis in bone marrow mesenchymal stem cells.	[387]

Exploration of combination therapies further accelerates clinical translation. For example, the synergistic use of Fe chelators with anti‐inflammatory agents concurrently suppresses ferroptosis and inflammatory signaling pathways, a strategy validated in Crohn's disease models [359]. Notably, while EGCG possesses dual Fe‐chelating and antioxidant properties, its low selectivity and pro‐oxidant risks at high concentrations necessitate mitigation through formulation optimization [359]. In current clinical practice, major investigational drugs include Fe chelators and antioxidants, detailed in Table 3.

**TABLE 3:** Clinical therapy of targeting ferroptosis in inflammatory diseases.

Category	Diseases/condition	Drug	NCT
Safety evaluation	Healthy volunteers	BPM31510 (a lipid–drug conjugate nanodispersion)	NCT03002935, NCT02486055
		Coenzyme Q10	NCT03429231, NCT05680857
		Deferiprone	NCT02465489, NCT02442310, NCT02189941, NCT01989455
		Deferasirox	NCT00419172, NCT00427505
		N‐acetylcysteine	NCT00552786, NCT01271088, NCT02723669, NCT02206178, NCT00434005
Metabolic disorders	Acute liver failure	N‐acetylcysteine	NCT00004467, NCT00248625, NCT03679442, NCT02182167, NCT03759158, NCT01394497
	End stage liver failure	N‐acetylcysteine	NCT00736541
	Acute lung injury	N‐acetylcysteine	NCT00655928
	Acute renal failure	N‐acetylcysteine	NCT01612013, NCT01907061, NCT00353340, NCT01467466, NCT00188630, NCT00187330, NCT01394419, NCT00736866, NCT02761577, NCT00356954, NCT00211653, NCT00122018
	Chronic kidney disease	N‐acetylcysteine	NCT00498342, NCT00506506, NCT05264584, NCT01232257, NCT00572663, NCT04916080, NCT03636932
		Coenzyme Q10	NCT03579693
	End‐stage renal disease	N‐acetylcysteine	NCT00440869, NCT00188630, NCT00187330
	Contrast‐induced nephropathy	N‐acetylcysteine	NCT01160627, NCT00497328, NCT00830193, NCT00492518, NCT00237614
		Deferiprone	NCT01146925
	Kidney transplantation	N‐acetylcysteine	NCT00851708
	Alcoholic hepatitis	N‐acetylcysteine	NCT03707951, NCT03216954, NCT03220776, NCT05840640, NCT00863785, NCT00962442, NCT00568087, NCT01214083
	Diabetes	Coenzyme Q10	NCT03111433, NCT02062034, NCT00703482
		N‐acetylcysteine	NCT00493727, NCT00463671, NCT00915200, NCT00556465, NCT01265563, NCT01082445, NCT00337038, NCT01386645, NCT00188773
	Hypercholesterolemia	Coenzyme Q10	NCT06391606
	Obesity	N‐acetylcysteine	NCT01550432, NCT02117700
	Chronic obstructive pulmonary disease	N‐acetylcysteine	NCT02818270, NCT01136239, NCT00969904, NCT03388853
Cardiovascular diseases	Cardiac arrest	Coenzyme Q10	NCT01319110
	Cardiac iron overload	Deferasirox	NCT01254227
		Deferoxamine	NCT01254227
	Diabetic cardiomyopathy	Coenzyme Q10	NCT02255682, NCT02115581
	Hypertrophic cardiomyopathy	N‐acetylcysteine	NCT01537926
	Myocardial infarction	N‐acetylcysteine	NCT01218178, NCT01501110
	Atherosclerosis	N‐acetylcysteine	NCT02422927
		Coenzyme Q10	NCT00908297
	Coronary artery disease	Coenzyme Q10	NCT01424761, NCT01163500, NCT00860847
		N‐acetylcysteine	NCT01021163
Central nervous system diseases	Parkinson's disease	Coenzyme Q10	NCT00076492, NCT01892176, NCT03061513, NCT00004731, NCT00180037
		Deferiprone	NCT02655315, NCT00943748, NCT02728843, NCT01539837 NCT02880033
		EPI‐743	NCT01923584
		N‐acetylcysteine	NCT01470027, NCT02212678, NCT02445651, NCT01427517
	Huntington's disease	Coenzyme Q10	NCT00980694, NCT00920699
	Autism spectrum disorder	N‐acetylcysteine	NCT03008889, NCT00889538, NCT00676195, NCT00627705, NCT00453180
	Depression	N‐acetylcysteine	NCT02269540
	Bipolar depression	Coenzyme Q10	NCT00720369, NCT01390389
		N‐acetylcysteine	NCT02294591, NCT02357290, NCT01797575, NCT05340504, NCT03730064
	Cannabis dependence	N‐acetylcysteine	NCT01005810, NCT00542750, NCT01675661, NCT03055377
	Cocaine abuse	N‐acetylcysteine	NCT02141620, NCT00218491, NCT02994875, NCT02124941, NCT00136825, NCT03556371
	Methamphetamine abuse	N‐acetylcysteine	NCT01063205, NCT00332605, NCT04405193
	Nicotine dependence	N‐acetylcysteine	NCT00751257, NCT02723162, NCT02737358,
	Obsessive‐compulsive disorder	N‐acetylcysteine	NCT01172275, NCT01555970
	Neuropathic pain	N‐acetylcysteine	NCT01840345, NCT03354572
		Coenzyme Q10	NCT00997269
	Traumatic brain injury	N‐acetylcysteine	NCT00724594, NCT01322009, NCT04291066, NCT01515839, NCT02791945
	Cognitive dysfunction	N‐acetylcysteine	NCT00611897
	Schizophrenia	N‐acetylcysteine	NCT01506765, NCT01232790, NCT01885338, NCT02505477, NCT01339858, NCT03510741, NCT01354132
	Dry eye syndrome	Coenzyme Q10	NCT03074344
		N‐acetylcysteine	NCT01747616, NCT01753752, NCT01278784, NCT01015209
Ischemia–reperfusion injury	Hepatectomy reperfusion injury	Deferasirox	NCT00432627
		N‐acetylcysteine	NCT01223326, NCT00564642
	Hypoxic–ischemic encephalopathy	N‐acetylcysteine	NCT04643821

*Data sources*: ClinicalTrials.gov.

Abbreviations: NCT: national clinical trial, HCC: hepatocellular carcinoma, AML: acute myeloid leukemia, CRC: colorectal cancer, NAC: N‐acetylcysteine, PDAC: pancreatic ductal adenocarcinoma, NSCLC: non‐small cell lung cancer, TNBC: triple‐negative breast cancer, CoQ10: coenzyme Q10.

#### 4.3.1 Targeting Ferroptosis to Enhance the Efficacy of Immunotherapy in the Treatment of Inflammatory Diseases

Recent studies continue to elucidate the dual regulatory role of ferroptosis (an Fe‐dependent cell death modality) in disease pathogenesis. Characterized by aberrant accumulation of LPO metabolites, this process can be effectively inhibited by Fe chelators, while exogenous Fe supplementation significantly potentiates the effects of inducers like erastin [388, 389]. In cardiovascular pathology models, ferroptosis has been implicated in myocardial IRI and chemotherapy‐associated cardiomyopathies: studies using ferritin‐deficient models demonstrate that doxorubicin‐induced myocardial injury involves Ripk3 (receptor‐interacting serine/threonine‐protein kinase 3)‐mediated necroptosis pathway activation, with ferroptosis inhibitor Fer‐1 intervention significantly improving survival outcomes [390, 391]. Mechanistically, experimental evidence showing ferroptotic cells promote neutrophil infiltration through the TLR4/IFN‐I signaling axis after cardiac transplantation further clarifies the molecular basis of its proinflammatory effects [392].

The regulatory network of Fe metabolism homeostasis also plays pivotal roles in organ fibrosis progression. SLC39A14 (solute carrier family 39 member 14) participates in ferroptosis‐driven hepatic fibrosis by modulating nontransferrin‐bound Fe levels [393], while Slc7a11 (solute carrier family 7 member 11) knockout models confirm that cystine/glutamate antiporter dysfunction under Fe overload specifically induces ferroptosis [394]. Notably, Slc7a11 exhibits unique biological functions in malaria parasite liver‐stage infection regulation: targeted inhibition of GPX4 or SLC7A11 significantly suppresses parasite proliferation through enhanced LPO, whereas NOX1 (NADPH oxidase 1, NADPH1) and TFR1 blockade produces opposing effects [395]. These findings suggest evolutionary significance of ferroptosis regulatory networks in host‐pathogen interactions.

## 5 Ferroptosis and Cancer

### 5.1 Ferroptosis in Tumor Biology: A Double‐Edged Sword

Small molecule‐induced ferroptosis represents an Fe‐dependent, LPO‐driven oxidative cell death modality mediated through transition metal Fe‐catalyzed redox reactions that generate ROS, demonstrating close associations with pathological processes in multiple diseases [396]. In neurological disorders, ferroptosis contributes to PD progression by causing mitochondrial dysfunction in dopaminergic neurons [397], while in oncology, this process significantly suppresses pancreatic cancer development through modulation of key proteins like GPX4 [398]. Research indicates that ferroptosis susceptibility is regulated by amino acid, Fe, PUFA, and GSH metabolism [32]. Interventions targeting these biological processes—such as copper ion modulation or Fe chelator application—can effectively alter tumor cell ferroptosis thresholds. Current targeted therapeutic strategies based on ferroptosis mechanisms have demonstrated promising antitumor growth potential in preclinical studies [399].

#### 5.1.1 Protumorigenic Roles

Ferroptosis, as an evolutionarily conserved cellular regulatory mechanism, demonstrates crucial biological functions in tumor biology. Multiple studies indicate its close association with tumor‐suppressive effects mediated by various factors. Taking the p53 protein family as an example, while its classical functions include cell cycle arrest and apoptosis induction, recent research has revealed novel tumor‐suppressive mechanisms through ferroptosis pathway regulation [400, 401, 402]. Notably, the posttranslational modification status of p53 significantly influences its proferroptotic activity. Studies demonstrate that p53 variants carrying triple lysine site mutations (3KR), despite losing traditional tumor‐suppressive functions, maintain ferroptosis‐mediated tumor suppression by activating the ALOX12 (arachidonate 12‐LOX) signaling pathway and significantly inhibiting SLC7A11 cystine transport system activity [401]. This functional specificity highlights the critical role of protein acetylation modifications in ferroptosis regulation. Research confirms that multiple tumor suppressors—including p53, BAP1 (BRCA1‐associated protein 1, fumarate hydratase (FH), Kelch‐like ECH‐associated protein 1 (KEAP1), and epigenetic regulator MLL4—participate in tumor suppression through ferroptosis pathway activation.

In the context of spatial heterogeneity, it is essential to note that ferroptosis may vary significantly between tumor subpopulations within the primary tumor and metastatic lesions. Studies using single‐cell RNA sequencing have indicated that ferroptosis‐related genes such as ALOX12 and SLC7A11 show differential expression patterns across distinct tumor regions, contributing to the heterogeneous response to FINs. This variation underscores the need for organ‐specific and spatially resolved analyses of ferroptosis to better understand its role in tumor progression and metastasis.

At the epigenetic regulation level, BAP1 as a histone deubiquitinase shows high‐frequency mutations in various malignancies [403]. This protein significantly suppresses target gene expression by regulating histone H2A deubiquitination at the SLC7A11 promoter region, thereby activating ferroptosis for tumor suppression [404]. BAP1 deficiency leads to mitochondrial morphological abnormalities and 4‐HNE protein downregulation [404], while exogenous BAP1 restoration inhibits tumor progression through liproxstatin‐1‐sensitive ferroptosis mechanisms, confirming the critical in vivo role of this pathway.

Spatially distinct tumor regions may exhibit differential levels of ferroptosis induction due to variations in oxygenation, nutrient availability, and metabolic stress. Recent studies have shown that the degree of ferroptosis in different regions of the tumor correlates with the availability of cystine and the activity of key metabolic pathways, such as FAO. This highlights the importance of understanding the spatial dynamics of ferroptosis to develop more effective therapeutic strategies.

Metabolic studies reveal that FH, a key TCA cycle enzyme, exhibits significant tumor‐suppressive functions in renal cell carcinoma (RCC) [405, 406]. FH mutations are detected not only in malignant lesions but also in precancerous conditions. Notably, FH‐deficient tumor cells demonstrate proliferation advantages under cystine deprivation that correlate with ferroptosis resistance, whereas wild‐type FH cells show marked growth inhibition under identical stress conditions, suggesting FH loss‐of‐function confers survival advantages in oxidative stress [407], a characteristic closely associated with ferroptosis‐resistant phenotypes.

Recent findings from single‐cell RNA sequencing and spatial transcriptomics have revealed that FH mutations are more prevalent in metastatic regions of RCC compared with primary tumors. This highlights the evolving role of ferroptosis in metastatic progression, where loss of FH activity could provide a survival advantage under the hypoxic and nutrient‐deprived conditions characteristic of secondary tumor sites.

KEAP1, as a critical component of the ubiquitin ligase complex, exerts tumor‐suppressive effects by promoting NRF2 protein degradation [408]. KEAP1 inactivation upregulates FSP1, conferring ferroptosis resistance [409, 410]. In glioma models, KEAP1 knockout significantly enhances tumor cell ferroptosis resistance through NRF2–SLC7A11 axis activation [411], molecularly elucidating the tumor‐suppressive mechanisms of KEAP1. Spatial variations in KEAP1 expression within tumors also contribute to differential ferroptosis sensitivity. In gliomas, for instance, KEAP1 expression may be reduced in the infiltrative tumor edge compared with the tumor core, influencing ferroptosis resistance and the effectiveness of ferroptosis‐targeted therapies in these regions.

Epigenetic studies demonstrate that histone methyltransferase MLL4 (mixed‐lineage leukemia protein 4) plays key preventive roles in skin cancer through ferroptosis‐related gene regulation [412]. This gene coordinates epidermal differentiation by activating critical gene clusters including ALOX12, thereby suppressing cutaneous squamous cell carcinoma development. Genetic analyses reveal that epidermal‐specific MLL4 knockout downregulates ALOXs family genes (including ALOX12, ALOX12B, and ALOXE3) while upregulating antiferroptosis genes like GPX4, ultimately causing epidermal differentiation defects and precancerous lesion formation [412]. These findings establish the central position of MLL4‐mediated ferroptosis pathways in maintaining skin homeostasis.

In gynecologic malignancies, ovarian cancer has garnered significant attention due to its high incidence and mortality rates [413]. Recent studies reveal that alantolactone regulates cellular redox homeostasis through dual mechanisms: dose‐dependently increasing intracellular ROS and ferrous ion concentrations while significantly suppressing key antioxidant proteins including SLC7A11 and GPX4 [414]. Notably, this compound markedly inhibits both translation and protein stability of SCD1 (sterol‐CoA desaturase 1), a lipid metabolism core enzyme, demonstrating significant tumor growth suppression in SKOV‐3 xenograft models [414, 415]. As a Δ9‐FA desaturase, SCD1 catalyzes monounsaturated FA biosynthesis, and its functional inhibition alters membrane lipid composition, ultimately triggering Fe‐dependent cell death [414].

In digestive system tumors, colorectal cancer (CRC) research has achieved substantial progress. RNA epigenetics studies demonstrate that chemical modifications at conserved sites critically regulate spatiotemporal gene expression, with methyltransferase‐like protein 17 (METTL17)‐mediated mitochondrial RNA methylation being closely associated with tumor metabolic reprogramming [416, 417, 418]. Clinical sample analyses show METTL17 overexpression in CRC tissues correlates positively with ferroptosis resistance. Mechanistically, this protein maintains mitochondrial‐encoded gene translation efficiency and energy metabolism homeostasis, effectively scavenging excess ROS and lipid peroxides [419]. Remarkably, Tagitinin C derived from Gentianaceae plants triggers characteristic LPO cascades by inducing oxidative stress microenvironments that activate the Nrf2/HO‐1 axis, causing labile Fe pool (LIP) expansion and GSH depletion [420]. The growth‐inhibitory effects of this natural compound on CRC cells provide experimental basis for novel ferroptosis‐targeted therapies.

For female reproductive system tumors, endometrial cancer research has achieved breakthroughs in ferroptosis regulation. Molecular evidence indicates sodium butyrate enhances endometrial cancer cell ferroptosis sensitivity by upregulating RNA‐binding protein RNA‐binding motif protein 3, which indirectly suppresses SLC7A11 transcriptional activity [421, 422, 423]. This discovery provides new theoretical support for epigenetic modulator applications in cancer therapy.

In hematologic malignancies, the metabolic regulatory mechanism of dihydroartemisinin on leukemia stem cells has been thoroughly dissected [424]. This compound activates the AMPK/mTOR/p70S6k signaling network, inducing ferritin autophagic degradation and cell cycle arrest to ultimately trigger Fe‐dependent cell death [425]. Notably, the functional status of Fe–S cluster assembly enzymes significantly influences tumor cell sensitivity to FINs, providing biomarker development directions for personalized treatment strategies.

During malignant tumor progression, metastatic dissemination represents the core biological feature underlying clinical treatment failure. Cross‐cancer studies reveal that cystatin SN (CST1, SN), a metastasis‐associated molecule, shows aberrant overexpression in colorectal, breast, and lung cancers [426]. Clinical cohort studies demonstrate significantly elevated CST1 levels in peripheral blood and ascites of gastric cancer patients with metastasis, with multivariate survival analyses confirming its role as an independent prognostic indicator [427]. Mechanistically, CST1 enhances cellular antioxidant capacity by stabilizing GPX4 protein while promoting EMT, thereby potentiating peritoneal, pulmonary, and hepatic metastasis of gastric cancer cells. Notably, ubiquitin‐specific protease 7 (USP7)‐mediated SCD1 deubiquitination has been identified as a crucial mechanism maintaining ferroptosis resistance in gastric cancer, with small‐molecule inhibitor DHPO effectively reversing this process through USP7 targeting, demonstrating significant metastasis suppression in animal models [428].

In breast cancer, a prevalent female malignancy, metastasis closely correlates with TME remodeling. Studies demonstrate that FIN erastin alters TAM phenotypes by increasing intracellular ferrous ion and ROS levels. Through exosome‐mediated intercellular communication, ferroptosis‐associated exosomes (Fe–Exos) significantly downregulate M2 macrophage markers Arg‐1 and CD206, consequently inhibiting tumor cell invasion and metastasis [429, 430]. In triple‐negative breast cancer (TNBC) research, Wilms tumor 1‐associated protein (WTAP)‐mediated N6‐methyladenosine (m6A) methylation has been shown to regulate Fe metabolic homeostasis via the nuclear protein 1/LCN2 axis. Epigenetic analyses reveal that WTAP deficiency causes mitochondrial Fe–S cluster biosynthesis defects, triggering LPO cascades and significantly suppressing metastatic potential [431, 432, 433].

Osteosarcoma metastasis research uncovers the critical role of the miR‐144‐3p/ZEB1 (zinc finger E‐box‐binding homeobox 1) regulatory axis in redox balance. This microRNA significantly reduces GSH/GSSG ratios and elevates ACSL4 levels through ZEB1 inhibition, altering membrane lipid composition and inducing Fe‐dependent death [434]. In RCC studies, KLF2 demonstrates transcriptional regulatory associations with GPX4. Clinicopathological analyses show shorter metastasis‐free survival in KLF2‐low patients, with mechanisms involving enhanced ferroptosis resistance‐mediated tumor cell invasiveness [435, 436].

Breakthroughs in ovarian cancer metastasis reveal transcription factor CEBPG (CCAAT/enhancer‐binding protein gamma) directly regulates SLC7A11 expression to modulate ferroptosis susceptibility. Proteomic analyses indicate CEBPG deficiency causes GSH metabolic pathway disruption, significantly inhibiting peritoneal metastatic seeding [437, 438]. In KRAS‐mutant CRC research, natural product ganoderic acid A exhibits unique autophagy‐dependent ferroptosis induction. This compound disrupts mitochondrial cristae structure and activates the ATF4–CHOP (C/EBP homologous protein) pathway, effectively suppressing splenic–hepatic metastasis in colon cancer models, providing novel therapeutic strategies for mutation‐driven tumors [439, 440].

Pancreatic ductal adenocarcinoma (PDAC) studies demonstrate mitochondrial calcium uniporter (MCU) regulates cystine metabolic reprogramming through the Keap1–Nrf2–SLC7A11 axis. Notably, MCU overexpression enhances metastatic potential while creating cystine addiction vulnerabilities, offering theoretical basis for synthetic lethal targeting strategies [441, 442]. In bladder cancer molecular subtyping, heat shock protein family A member 5 promotes tumor progression through dual mechanisms: regulating vascular endothelial growth factor A‐mediated angiogenesis and maintaining ferroptosis resistance via the p53/SLC7A11 pathway, highlighting its therapeutic potential [443, 444, 445].

Head and neck squamous cell carcinoma research reveals caveolin 1 modulates redox homeostasis through membrane lipid domain regulation. Its downregulation induces GPX4/FTH1 complex dissociation, effectively suppressing metastasis via an Fe overload‐LPO positive feedback loop [446, 447]. Melanoma studies identify prominin 2 (PROM2) as maintaining membrane stability through endocytic recycling regulation. Preclinical research demonstrates that PROM2 targeting significantly enhances ferroptosis sensitivity and blocks metastasis across multiple PDX models [448, 449].

#### 5.1.2 Tumor Suppressive Roles

Although Fe‐dependent cell death pathways play significant roles in tumor suppression, clinical observations reveal that most malignancies maintain progressive biological features, indicating tumor cells have evolved sophisticated defense systems to evade this PCD modality.

The tumor suppressor p53, as the most frequently dysregulated regulatory protein in human malignancies, exhibits complex interactions with ferroptosis regulatory networks [450, 451]. Beyond its classical roles in cell cycle arrest and apoptosis, p53 modulates ferroptosis through multidimensional mechanisms. Molecular studies demonstrate that p53 directly targets the SLC7A11 gene promoter or regulates USP7‐mediated histone H2B mono‐ubiquitination modifications, thereby enhancing tumor cell ferroptosis sensitivity via ALOX12‐dependent mechanisms [452, 453]. In addition to direct cystine metabolism regulation, p53 participates in ferroptosis control through LPO‐related metabolic pathways [454, 455]. Notably, specific p53 mutants retain tumor‐suppressive capacity via ferroptosis induction despite losing apoptosis or senescence‐inducing abilities [456], providing new perspectives for deciphering functional heterogeneity in p53 mutation spectra. However, under specific microenvironmental conditions, p53 may paradoxically exhibit ferroptosis‐suppressive functions [457], highlighting the spatiotemporal specificity of its regulatory networks.

BAP1, as a crucial epigenetic regulator, encodes a nuclear deubiquitinase involved in chromatin remodeling through histone H2A ubiquitination modifications [458, 459]. This protein exhibits high‐frequency mutations in various malignancies including uveal melanoma [460]. Functional studies reveal that BAP1 reduces H2Aub levels at the SLC7A11 promoter through its enzymatic activity, thereby inhibiting cystine uptake and inducing ferroptosis—a process demonstrating significant tumor suppression in animal models [460]. Clinically prevalent BAP1 loss‐of‐function mutations impair its ability to regulate SLC7A11 expression, consequently attenuating proferroptotic effects [460].

In oxidative stress regulation, the functional status of the KEAP1–NRF2 signaling axis critically determines ferroptosis susceptibility [461, 462]. KEAP1, as a negative regulator of NRF2, shows that its inactivation mutations enhance NRF2 protein stability, activating antioxidant genes including SLC7A11 [463, 464]. The alternative reading frame (ARF) tumor suppressor inhibits NRF2 transcriptional activity through a noncanonical mechanism independent of its known p53‐activating function [464]. In ARF‐deficient tumor models, aberrant NRF2 pathway activation significantly enhances ferroptosis resistance [465]. These findings suggest that KEAP1 and ARF cooperatively maintain redox homeostasis through NRF2 regulation, with their functional loss‐mediated NRF2 hyperactivation emerging as a key mechanism for tumor cell ferroptosis evasion.

### 5.2 Molecular Regulation of Ferroptosis in Cancer

#### 5.2.1 Cancer Cell‐Intrinsic Signaling Pathways

##### 5.2.1.1 Tumor Protein p53

One of the primary functions of p53 is to monitor DNA damage within cells and assist in repairing such damage or prompting damaged cells to undergo PCD, thereby preventing the further proliferation of aberrant cells [466]. It plays a critical role in cellular responses to various stresses [467], including p53‐mediated cell cycle arrest, senescence, and apoptosis, which are crucial barriers to cancer development [468].

Jiang et al. [466] revealed that p53 inhibits cystine uptake by suppressing the expression of the critical component of the cystine/glutamate antiporter, SLC7A11, thereby affecting GPX4 activity and sensitizing cells to ferroptosis [469]. They found that p53 directly targets response elements in the SLC7A11 promoter to inhibit its expression, triggering a cascade reaction. As extracellular cystine uptake decreases, GSH synthesis declines, GPX4 activity decreases, lipid peroxide levels increase, ultimately leading to ferroptosis. Additionally, p53 suppresses cystathionine beta‐synthase (CBS), inhibiting the transsulfuration pathway of serine, which limits GSH production and indirectly inhibits GPX4 enzyme activity, thereby promoting ferroptosis [470, 471].

The p53 mutant p533KR, with three lysine residues replaced by arginine residues at positions 117, 161, and 162, is highly effective in inhibiting SLC711A expression but does not affect the expression of other known p53 target genes involved in cell cycle regulation (such as CDKN1A/p21) or apoptosis [466]. In contrast, the p53^4KR98^ mutant (with an additional lysine residue replaced at position 98) fails to reduce SLC711A expression [472].

SAT1 (spermidine/spermine N1‐acetyltransferase 1) serves as a critical regulator of polyamine metabolism, acetylating spermidine and spermine using acetyl coenzyme A. Aberrant SAT1 expression has been implicated in various pathological conditions [473]. SAT1 activity is sensitive to multiple stresses and has recently been identified as a target of p53 [474]. SAT1 induces ferroptosis through the expression pathway of ALOX15 (arachidonate 15‐LOX) rather than affecting SLC7A11 and GPX4 [474]. Under normal conditions, SLC7A11 binds to its substrate PUFA (including those esterified in membranes) and sequesters ALOX12. Under oxidative stress, p53 enhances ALOX12 activity by downregulating SLC7A11 [469]. Upon release of ALOX12, oxidative membrane PUFAs produce lipid peroxides, triggering ferroptosis [475]. The mechanism by which ALOXE3 induces ferroptosis in GBM cells is similar to that of ALOX12 [476], and notably, both ALOX12 and ALOXE3 axes operate independently of GSH and GPX4.

Glutamine metabolism represents another target altered in ferroptosis, where the expression of GLS2 (glutaminase 2) is directly correlated with p53‐mediated oxygen consumption, mitochondrial respiration, and ATP production in cancer cells [477], serving as a direct transcriptional target of p53. Under conditions of cysteine deficiency or even partial or complete depletion of amino acids, glutamine can induce ferroptosis in a serum‐dependent manner [65]. Mechanistically, p53 binds to response elements in GLS2 and induces its transcription. Subsequently, GLS2 converts glutamine to glutamate, which is transformed into α‐ketoglutarate. A p53 variant (S47) exhibits impaired GLS2 transactivation ability, reducing GLS2 levels in S47 tumor cells and resisting ferroptosis.

##### 5.2.1.2 Nuclear Factor Erythroid 2–Related Factor 2

Nrf2 is a central regulatory factor in antioxidant responses, binding to the nucleus and coordinating the expression of numerous vital cellular antioxidant genes. Typically, Nrf2 exists in three complexes and maintains a basal low level in organisms; for instance, its protein‐bound form is associated with antioxidation, while the activity of Nrf2 is strictly regulated by Keap1 [478]. KEAP1 participates in intracellular redox balance and antioxidant responses.

Under normal conditions, Nrf2 binds to Keap1 and is inactivated via ubiquitination and degradation in the proteasome [479]. However, upon oxidative stress, Keap1 undergoes autophagic degradation, releasing Nrf2 [480]. Free Nrf2 rapidly translocates to the nucleus, where it binds to antioxidant response elements (AREs) in the promoter regions, driving the expression of antioxidant genes to balance intracellular oxidative stress and ultimately maintain cellular redox homeostasis [481]. Furthermore, Nrf2 can regulate numerous proteins and enzymes associated with ferroptosis. For example, subunits of system XC, GPX2, and GSH synthetase are all targets of Nrf2 [482]. Multiple studies have demonstrated that Nrf2 is a critical regulatory factor in cellular ferroptosis. Enhancing Nrf2 activity in renal tubules through upregulating gene expression or drug therapy can ameliorate AKI, and targeting the Keap1–Nrf2 system can prevent the progression of kidney diseases [483].

Recently, Koppula et al. [484] identified FSP1 as a transcriptional target of Nrf2. The CoQ–FSP1 axis was shown to mediate ferroptosis and radioresistance in KEAP1‐deficient lung cancer cells, further highlighting the close association between Nrf2 and cellular ferroptosis.

##### 5.2.1.3 PI3K–AKT–mTOR

The mTOR serves as a central hub for cellular metabolic regulation, integrating growth factor signaling and nutrient status to coordinate key biological processes including protein synthesis and lipid metabolism [485]. In tumor metabolic reprogramming research, the regulatory role of the PI3K–AKT–mTOR signaling axis in lipid biosynthesis has attracted considerable attention. This pathway promotes maturation and nuclear translocation of transcription factor SREBP1 (sterol regulatory element‐binding protein 1) through mTORC1 (mTOR complex 1) activation, thereby driving expression of lipid synthesis enzymes including SCD1 [486]. SCD1 catalyzes saturated FA conversion to monounsaturated FAs, crucial for maintaining membrane lipid composition and antioxidant stress capacity. Mechanistically, tumors with PIK3CA activating mutations or PTEN loss exhibit significantly enhanced ferroptosis resistance via the SREBP1/SCD1 axis [487]. Notably, mTORC1 regulates ferroptosis susceptibility through dual mechanisms: promoting GPX4 biosynthesis and enhancing GSH metabolic pathway activity [488]. Elucidation of this metabolic regulatory network provides theoretical foundation for combination therapies targeting PI3K–AKT–mTOR pathway inhibitors, with preclinical studies confirming their ability to significantly sensitize tumor cells to FINs.

In lung cancer molecular subtyping, the LKB1/KEAP1 double‐mutant subtype exhibits unique metabolic dependencies. Approximately 10% of lung adenocarcinoma patients harbor this genetic combination, demonstrating significantly worse prognosis than other subtypes [489]. Mechanistically, KEAP1 inactivation causes sustained NRF2 pathway activation, upregulating antioxidant genes including SCD1 [490]. Similar to PI3K–AKT–mTOR‐activated tumors, these lung cancer cells show high dependency on SCD1 activity, suggesting shared ferroptosis resistance mechanisms across genetically distinct tumors. From a translational perspective, combinatorial strategies targeting SCD1 (via direct inhibitors or upstream pathway modulators) with FINs may emerge as a universal therapeutic approach for multigenic mutant tumors.

##### 5.2.1.4 Hypoxia‐Inducible Factor

Hypoxia, a hallmark of solid TMEs, drives malignant progression and therapy resistance through activation of HIF signaling networks. The HIF heterodimer, composed of oxygen‐sensitive α subunits (HIF‐1α/endothelial PAS domain protein 1/HIF‐3α) and a constitutively stable β subunit (ARNT), demonstrates aberrant activation strongly associated with poor prognosis across multiple malignancies [491, 492]. Recent studies reveal that the HIF signaling axis regulates ferroptosis susceptibility through complex metabolic reprogramming mechanisms, exhibiting marked tumor type specificity.

In GBM models, HIF‐1α enhances SLC7A11 expression via PI3K/AKT‐dependent transcriptional activation, significantly reducing intracellular ROS levels and counteracting thioacetamide‐induced ferroptosis [493]. Notably, this regulatory mechanism persists in gastric cancer peritoneal metastases, with proteomic studies confirming that HIF‐1α establishes an SLC7A11‐mediated antioxidant defense system by inducing peritoneal metastasis‐associated protein (PMAN) expression, thereby maintaining lipid metabolic homeostasis in metastatic niches [494, 495]. Contrastingly, non‐small cell lung cancer (NSCLC) studies demonstrate that trabectedin activates the TFR1/HIF‐1/IRP1 axis, triggering Fe overload and ROS burst to induce ferroptosis [496]. This bidirectional regulation underscores the context‐dependent nature of HIF‐mediated ferroptosis control.

At the epigenetic level, functional loss of Fe–S cluster assembly protein ISCA2 disrupts HIF‐1/2α signaling, inducing LPO imbalance in pVHL‐deficient clear cell RCC [497]. This discovery provides novel therapeutic avenues targeting metabolic cofactors. Intriguingly, GBM multiforme research reveals that HIF activation enhances ferroptosis susceptibility in cancer stem cells, with this seemingly paradoxical regulatory effect potentially linked to cellular differentiation states [497], highlighting the profound impact of microenvironmental heterogeneity on therapeutic responses.

##### 5.2.1.5 Hippo‐YAP

The Hippo signaling pathway, a critical molecular system governing organ size control, maintains tissue homeostasis through precise regulation of cell proliferation and apoptosis. This pathway consists of highly conserved kinase cascade components, with its core function being suppression of proto‐oncogenic transcriptional coactivators YAP/TAZ (YAP/transcriptional coactivator with PDZ‐binding motif) activity [498]. In cellular density sensing, E‐cadherin‐mediated intercellular junctions not only serve as structural foundations for epithelial polarity but also act as key regulators of Hippo signaling transmission. Experimental evidence demonstrates a significant positive correlation between E‐cadherin expression levels and cell density, with E‐cadherin‐mediated adhesion complexes activating neurofibromin 2 (NF2) to modulate Hippo pathway activity and inhibit YAP nuclear translocation [499, 500].

Mechanistic studies reveal that the YAP/TAZ–TEAD4 (TEA domain transcription factor 4) complex directly binds promoter regions of ferroptosis‐associated genes. Specifically, this complex establishes an Fe metabolism‐LPO regulatory network by controlling transcriptional activity of TFRC and ACSL4. Elevated cell density significantly reduces TFRC and ACSL4 expression through YAP phosphorylation, generating ferroptosis‐resistant phenotypes [501]. Notably, genetic ablation of E‐cadherin or NF2 restores ferroptosis susceptibility by relieving YAP phosphorylation inhibition, unveiling the critical role of intercellular contact signaling in oxidative stress defense.

Recent breakthroughs elucidate cross‐talk mechanisms between Hippo and mTORC1 signaling networks. The large tumor suppressor kinases LATS1/2 (LATS1/2) inhibit mTORC1 complex activity through phosphorylation modification at Ser606 of Raptor protein [502]. This dual regulatory mechanism holds particular significance in metabolic reprogramming: the Hippo pathway modulates ferroptosis‐associated genes via YAP/TAZ while simultaneously influencing mTORC1‐mediated anabolic processes through LATS1/2, establishing a multilayered oxidative stress defense system. These findings provide novel perspectives for understanding tumor cell adaptive responses under density stress and suggest that combination therapies targeting intercellular signaling networks may potentiate ferroptosis‐inducing therapeutic efficacy.

#### 5.2.2 The TME and Ferroptosis

The TME is a critical regulatory system governing tumor initiation and progression. It consists of malignant cells, stromal supporting cells, heterogeneous immune cell subsets, vascular networks, and noncellular stromal elements [502]. Tumor cells establish functional networks with the surrounding microenvironment through bidirectional signaling, which significantly impacts tumor growth, immune escape, and therapeutic resistance. These interactions define how the TME influences not only tumor biology but also the susceptibility of the tumor to various forms of cell death, including ferroptosis.

Ferroptosis is intricately connected to the TME. It is not only the result of metabolic alterations in cancer cells but also involves the release of bioactive molecules that can activate or suppress immune responses. These molecules, such as lipid mediators and cytokines, affect both tumor cells and immune effector cells, thereby modulating antitumor immunity. Immune cells in the TME, through cytokine and ROS release, significantly alter the sensitivity of tumor cells to ferroptosis, thus shaping the overall immune response to the tumor.

A key aspect of ferroptosis in the TME is its influence on immune cells. CD8+ T lymphocytes, crucial for tumor immune surveillance, exhibit heightened sensitivity to FINs compared with tumor cells [503]. Ferroptotic interventions targeting both tumor cells and immune effector populations can profoundly affect tumor progression by altering immune cell survival and function. This dual‐action mechanism shapes the TME, influencing immune responses and the overall TME reaction to immunotherapy.

Studies have shown that GPX4 dysfunction in CD8+ T cells increases LPO, promoting ferroptosis and impairing T cell function [504]. Interestingly, pharmacological inhibition of GPX4 selectively induces ferroptosis in CD8+ T cells without significantly affecting tumor cell viability [505]. This introduces challenges in balancing T cell homeostasis within the TME, as unchecked ferroptosis could impair immune function.

Regulatory T cells (Tregs), which play a critical role in immune suppression, are also affected by ferroptosis. Research has demonstrated that combining FINs with PD‐L1 blockers can reduce Treg numbers in the TME, while enhancing CD8+ T cell activation and promoting antitumor immunity [506]. Additionally, Treg‐specific GPX4 knockout results in LPO accumulation, leading to IL‐1β secretion and the activation of TH17‐mediated antitumor responses [507].

TAMs, predominantly polarized to the protumorigenic M2 phenotype, are crucial regulators of ferroptosis in the TME. These M2‐polarized macrophages typically exhibit resistance to ferroptosis induced by GPX4 deficiency [508, 509]. However, recent studies have shown that FINs can reprogram macrophages from the M2 to an M1 phenotype. This reprogramming enhances proinflammatory cytokine secretion and antigen presentation, optimizing antitumor immune responses [510, 511].

This functional plasticity of TAMs underscores the importance of manipulating macrophage polarization in the context of ferroptosis. For example, Fe‐based nano‐delivery systems, designed to target macrophage polarization, have shown promising results in modulating macrophage phenotypes and boosting antitumor immunity [512].

Myeloid‐derived suppressor cells (MDSCs) are pivotal players in the immunosuppressive TME. Ferroptosis research has uncovered that inducing ferroptosis in polymorphonuclear MDSCs (PMN‐MDSCs) can reverse their immunosuppressive effects on T lymphocytes and NK cells, thus enhancing the efficacy of checkpoint inhibitors [513]. MDSCs exhibit complex lipid metabolic reprogramming as a resistance mechanism against ferroptosis. They suppress dendritic cell (DC) antigen presentation through myeloperoxidase‐mediated lipid transport and demonstrate selective enrichment of AA‐esterified triglycerides [514, 515].

Furthermore, MDSCs competitively deplete cystine in the TME via the Xc^−^ system, limiting the availability of essential amino acids for T cell activation [516]. Targeting these pathways could sensitize MDSCs to ferroptosis, providing an innovative strategy for improving immunotherapy outcomes.

Ferroptosis is also characterized by the release of lipid signaling molecules that regulate immune responses within the TME. These oxidized lipids, produced via LOX‐catalyzed pathways from esterified eicosanoids, function as “danger signals” to recruit antigen‐presenting cells to the tumor site [517]. Notably, oxidized PE species act as “phagocytic signals,” activating macrophages to clear ferroptotic cells [518].

The immunomodulatory functions of these lipid mediators are further evidenced by the regulation of proinflammatory mediators through NF‐κB signaling [517, 519]. However, some oxidized lipids, such as LTB4, have been shown to promote tumor progression through specific receptor signaling [520]. This dual role of lipids in both immune activation and tumor promotion adds complexity to the regulation of ferroptosis and its impact on tumor progression.

In summary, immune effector cells within the TME play a critical role in modulating ferroptosis sensitivity and regulation. Cytokines such as IFN‐γ and TGF‐β1 are key regulators in this process. IFN‐γ induces a ferroptosis‐prone state by suppressing the Xc^−^ system and increasing intracellular Fe storage [521], while TGF‐β1 enhances ferroptosis by downregulating the Xc^−^ system via SMAD signaling [522]. These bidirectional regulatory mechanisms between the immune system and ferroptosis lay the foundation for developing combined immunotherapeutic strategies aimed at modulating ferroptosis in tumor cells and immune cells.

### 5.3 Targeting Ferroptosis for Cancer Therapy

#### 5.3.1 Small Molecule Inducers and Inhibitors

A major challenge in current cancer therapy lies in the development of chemotherapeutic drug resistance in malignant cells, which has become a critical barrier limiting sustained treatment efficacy. Recent studies demonstrate that ferroptosis mechanisms play pivotal roles in regulating cell death and suppressing tumor growth across multiple malignancies [523, 524, 525]. Targeted intervention strategies focusing on ferroptosis regulatory pathways, when integrated with current innovative therapeutic modalities targeting molecular pathways, exhibit potential to overcome traditional drug resistance bottlenecks.

However, despite significant preclinical promise, the clinical translation of ferroptosis‐inducing therapies faces numerous hurdles. One of the key challenges lies in the toxicity of some FINs, such as ferrostatin‐1 and DFO, which have shown varying degrees of toxicity in both preclinical models and early‐phase clinical trials. Additionally, the specificity of these compounds in targeting tumor cells, without affecting normal cells, remains a critical issue. For instance, DFO, while effective at inducing ferroptosis, also binds to Fe in normal tissues, potentially leading to Fe overload and systemic side effects [526]. Thus, optimizing the therapeutic index by improving the specificity and selectivity of these drugs is a priority.

Combination therapies could offer a way to enhance the effectiveness of FINs while minimizing side effects. Ferrostatin‐1, for example, when used in combination with chemotherapeutic agents such as anthracyclines or platinum‐based drugs, could potentially enhance antitumor efficacy by sensitizing tumor cells to ferroptosis [527, 528]. However, the challenges associated with drug resistance and tumor heterogeneity complicate this approach, underscoring the need for precise molecular subtyping to tailor combination therapies for specific patient populations [526].

Developing novel anticancer drugs targeting ferroptosis remains a protracted process, though recent advances in ferroptosis‐inducing compounds have demonstrated therapeutic promise [526]. In Table 4, we summarize multiple therapeutic agents affecting newly identified ferroptosis mechanisms tested in vivo and/or in vitro [529].

**TABLE 4:** Classical pharmacological agents inducing ferroptosis for antitumor treatment.

Compounds	Target	Mechanism	Cancer type	References
Erastin	SLC7A11	GSH depletion by inhibiting SLC7A11 activity	HCC Melanoma NSCLC LUAD Ovarian cancer Anaplastic thyroid cancer	[93] [539] [540] [541] [542] [543] [544]
IKE	SLC7A11	GSH depletion by inhibiting SLC7A11 activity	DLBCL Sarcoma GBM NSCLC	[545] 532 546 547 548
Cystinase	Cystine	GSH depletion by degrading cysteine and cystine	Prostate cancer Breast cancer Lung cancer Pancreatic tumors	[549] [550] [94] [551]
RSL3	GPX4	GPX4 inactivation	Fibrosarcoma TNBC HCC Lung cancer Anaplastic thyroid cancer	[545] [552] [553] [554] [555] [544]
M162	GPX4	GPX4 inactivation	TNBC Anaplastic thyroid cancer	[553] [544]
ML210	GPX4	GPX4 inactivation	HNC TNBC NSCLC Anaplastic thyroid cancer	[556] [557] [558] [544]
FIN56	GPX4	GPX4 degradation	GBM Lung cancer	[559] [560]
FINO_2_	Iron and GPX4	Iron oxidation and GPX4 inactivation	Fibrosarcoma	[561]
Hemin	Iron	Iron loading	Lung cancer	[562]
icFSP1	FSP1	Phase separation of FSP1	Melanoma	[563]
iFSP1	FSP1	FSP1 inhibition	TNBC NSCLC	[564] [565]

In ferroptosis regulatory mechanisms, System Xc^−^ inhibitors trigger Fe‐dependent cell death by interfering with cystine transmembrane transport, leading to abnormal accumulation of unfolded proteins under endoplasmic reticulum stress. Studies confirm that erastin analogues significantly reduce intracellular GSH biosynthesis through targeted inhibition of System Xc^−^ transporter function [530]. Particularly in RAS‐mutant tumors, these compounds specifically activate RAF/MEK/ERK signaling cascades by targeting VDAC proteins on mitochondrial membranes. Gene silencing experiments reveal that loss of VDAC2/3 isoform expression confers marked resistance to erastin, providing a rationale for clinical combination therapy—these agents enhance cytotoxicity of anthracyclines and platinum‐based chemotherapeutics [527, 528]. Notably, pharmacokinetic optimization of erastin derivatives has achieved critical progress: piperazine derivatives maintain pharmacological activity while improving aqueous solubility [531], whereas keto group modifications simultaneously enhance compound stability and antitumor efficacy [532].

As a core regulatory component of ferroptosis, GPX4 exhibits dual biological significance. Given the pivotal role of GSH metabolism in tumor biology [533], GPX4 inhibition represents an emerging therapeutic strategy. Experimental data demonstrate that small‐molecule inhibitors like RSL3 directly target the GPX4 active site, inducing permanent enzyme inactivation via covalent modification of critical catalytic cysteine and selenocysteine residues [534]. This approach displays dual advantages in preclinical models: effective elimination of tumor stem cell populations and prevention of residual lesion regeneration posttreatment [535].

Crucially, tumor metabolic plasticity may influence therapeutic efficacy: certain malignancies bypass System Xc^−^ inhibition by converting methionine to cysteine via the transsulfuration pathway, emphasizing the necessity for precise molecular subtyping in ferroptosis‐related therapies [526].

Clinical research on pharmacological agents inducing ferroptosis as an antitumor therapeutic strategy is rapidly evolving. Multiple drug classes have entered clinical investigation, including system Xc^−^ inhibitors and GPX4 inhibitors, which promote LPO accumulation in tumor cells by blocking cystine uptake or inhibiting GPX activity [536]. For instance, sorafenib, an approved chemotherapeutic agent, enhances hepatocellular carcinoma cell sensitivity to ferroptosis through system Xc^−^ inhibition, demonstrating synergistic antitumor effects when combined with FINs in preclinical models [537]. Emerging agents targeting metabolic pathways, such as CPT1A (carnitine palmitoyltransferase 1A) inhibitors, have validated ferroptosis‐inducing capacity in NSCLC models by disrupting FAO to potentiate LPO [538].

Therapeutic strategies combining immunotherapy with FINs are gaining significant attention. Studies reveal that radiotherapy or PD‐1/PD‐L1 inhibitors can amplify antitumor immune responses through ferroptosis activation, with related combination regimens undergoing evaluation in early‐phase clinical trials [538]. Nevertheless, achieving tumor‐selective ferroptosis induction remains challenging. Current research prioritizes optimizing Fe metabolism regulation and nanoparticle‐based drug delivery systems as pivotal approaches for enhancing the therapeutic window (Table 5).

**TABLE 5:** Clinical trial drugs inducing ferroptosis for antitumor treatment.

Drugs	Target	Cancer type	Indication	NCT
Sorafenib	SLC7A11	HCC GC CCRC	HCC AML Neuroblastoma Lung cancer	NCT03794440 NCT03247088 NCT02559778 NCT00064350
Sulfasalazine	SLC7A11	Prostate cancer Lymphoma Lung cancer CRC HNC PDAC OCCC	GBM GBM Breast cancer Solid tumor	NCT04205357 NCT01577966 NCT03847311 NCT01198145
Lapatinib	Iron	Breast cancer	Breast cancer Breast cancer Breast cancer	NCT03085368 NCT00356811 NCT00667251
Neratinib	Iron	Breast cancer	Breast cancer CRC CRC	NCT04366713 NCT03377387 NCT03457896
Artesunate	Iron	NHL HCC	Breast cancer CRC	NCT00764036 NCT03093129
Cisplatin	GSH	GC HNC NSCLC	NSCLC Bladder cancer Cervical cancer Pancreas cancer	NCT01656551 NCT04574960 NCT01561586 NCT03649321
Gemcitabine	GPX4	PDAC LUAD	Pancreatic cancer BTC Solid tumor	NCT06015659 NCT05357196 NCT05147272
Withaferin A	GPX4	Neuroblastoma HCC	Ovarian cancer Advanced cancer Osteosarcoma	NCT05610735 NCT04092647 NCT00689195
Lovastatin	HMGCR	NSCLC	Prostate cancer Ovarian Cancer	NCT00580970 NCT00585052
Simvastatin	HMGCR	TNBC	Multiple myeloma	NCT00281476
Haloperidol	DRD2	GBM	Advanced cancer	NCT04833023 NCT03743649 NCT00124930
Zalcitabine	DNA stress	Pancreatic cancer	AIDS‐related Kaposi sarcoma	NCT00000954
β‐Elemene	TFEB	NSCLC	NSCLC GBM	NCT03123484 NCT02629757
BSO	GCL	TNBC	Neuroblastoma Neuroblastoma	NCT00005835 NCT00002730
Brequinar	DHODH	Cervical cancer Colon cancer Fibrosarcoma Lung cancer	AML	NCT03760666
Curcumenol	FTH1	Lung cancer	Lung cancer	NCT00475683

*Data sources*: ClinicalTrials.gov.

Abbreviations: SLC7A11: solute carrier family 7 member 11, GSH: glutathione, GPX4: glutathione peroxidase 4, HMGCR: 3‐hydroxy‐3‐methylglutaryl‐CoA reductase, GCL: glutamate cysteine ligase, FTH1: ferritin heavy chain 1, TFEB: transcription factor EB, DRD2: dopamine receptor D2, DHODH: dihydroorotate dehydrogenase.

#### 5.3.2 Nanotechnology‐Based Approaches

The dynamic remodeling of the tumor metabolic microenvironment offers unique therapeutic windows for targeting ferroptosis, with breakthroughs in nanomaterial engineering introducing novel possibilities. In tumor immune regulation, CD8+ T cell‐derived IFN‐γ exerts multidimensional control: it disrupts the oxidative defense barrier of tumor cells by suppressing the SLC7A11‐mediated cystine transport system, while simultaneously activating ACSL4 to catalyze biosynthesis of PUFA phospholipids such as AA. This dual mechanism significantly amplifies LPO [566, 567]. Neutrophils further establish a proferroptotic oxidative microenvironment through extracellular secretion of myeloperoxidase granules [568], providing a theoretical foundation for designing immune‐synergistic nanoformulations.

Nanomaterials exhibit unique advantages in ferroptosis modulation. Fe‐based nanomaterials initiate localized Fenton reactions via controlled Fe^2^⁺/Fe^3^⁺ ion release, efficiently generating ROS and triggering LPO cascades [569]. Non‐Fe‐based nanosystems achieve synergistic induction of metabolic reprogramming and ferroptosis through intelligent drug delivery of FINs or surface‐functionalized modulation of GSH metabolic pathways [570]. Novel biomimetic nanocarriers can precisely enrich PUFAs in tumor cell membranes by mimicking natural lipoprotein structures.

#### 5.3.3 Combination Therapies

Emerging evidence demonstrates that modulating tumor cell ferroptosis sensitivity significantly impacts immunotherapy responses, with ferroptosis resistance showing a significant association with immune checkpoint inhibitor (ICI) therapy resistance. Combining PD‐L1 immune blockade with cysteine metabolism modulation or AA pathway intervention effectively activates antitumor immunity, with combination regimens exhibiting superior tumor suppression compared with monotherapies [571, 572]. Molecular mechanistic studies confirm that SLC7A11‐deficient tumor models display heightened sensitivity to PD‐L1 monoclonal antibody therapy and its combination with radiotherapy [573]. Notably, periodic methionine‐restricted diets enhance cystine deprivation‐induced ferroptosis by activating the CHAC1 (GSH‐specific gamma‐glutamylcyclotransferase 1)‐mediated GSH degradation pathway, thereby improving tumor response rates to PD‐1 inhibitor therapy [574]. Further investigations reveal that combining methionine restriction with the SLC7A11‐specific inhibitor imidazole ketone erastin and PD‐1 blockade produces synergistic antitumor effects, significantly prolonging survival in experimental models [574].

At the molecular level, researchers have identified the pivotal role of the PKCβII–ACSL4 signaling axis in ferroptosis regulation. This pathway amplifies LPO through PKCβII‐mediated phosphorylation of ACSL4, thereby promoting ferroptosis execution. Experimental evidence shows that genetic knockout of PKCβII or ACSL4, or site‐specific mutation blocking ACSL4 phosphorylation, significantly suppresses ferroptosis and T cell‐mediated tumor immune killing, ultimately driving resistance to PD‐1 inhibitor therapy [575]. This discovery provides critical molecular insights into the synergy between ferroptosis and immunotherapy.

The association between ferroptotic cells and immune suppression in the TME exhibits multidimensional regulatory features. Recent studies suggest that DAMPs such as KRAS–G12D, HMGB1, and 8‐hydroxyguanosine (8‐OHG) may modulate immune cell functions via receptor‐mediated signaling networks. Pancreatic cancer cells have been experimentally confirmed to release KRAS–G12D through the autophagy‐lysosome pathway, a process involving dynamic fusion of autophagosome and multivesicular body membranes [576]. Upon binding to the AGER, KRAS–G12D significantly alters macrophage metabolic profiles, inducing arginine metabolic reprogramming and promoting secretion of immunosuppressive factors like IL‐10 and TGF‐β (transforming growth factor‐beta), thereby establishing an immune‐evasive microenvironment [576]. Similar mechanisms are observed in HMGB1‐mediated inflammatory responses, where this molecule enhances macrophage proinflammatory phenotypes via AGER‐dependent signaling [577]. Notably, 8‐OHG released under GPX4 deficiency activates the STING (stimulator of IFN genes) pathway, driving aberrant expression of inflammatory mediators such as IL‐6 and NOS2 (nitric oxide synthase 2), providing new insights into pancreatic cancer inflammatory microenvironment formation [578].

Research on ferroptosis‐immune interplay has expanded to prostaglandin metabolism. Experimental data indicate that upregulated PTGS2 expression with excessive PGE2 (prostaglandin E2) secretion may serve as a critical checkpoint for immunosuppressive microenvironment formation [579]. At threshold concentrations, PGE2 suppresses NK cell, DC, and cytotoxic T lymphocyte (CTL) antitumor activity by modulating MDSC and M2 macrophage functions [580]. Mechanistically, PGE2 induces DNMT3A (DNA methyltransferase 3 alpha) activation in myeloid cells, triggering epigenetic silencing of immune‐related genes [581]. In BrafV600E‐mutant melanoma models, PGE2 blocks DC‐mediated antigen presentation, impairing CD8+ T cell immunosurveillance [582]. Furthermore, PGE2 reduces intratumoral DC infiltration by downregulating chemokines CCL5 (C‐C motif chemokine ligand 5) and XCL1 (X‐C motif chemokine ligand 1) [583], while directly suppressing CTL cytotoxicity [584], collectively highlighting the pivotal role of ferroptosis‐related metabolites in immune evasion.

ROS metabolism demonstrates dual regulatory roles in tumor immunity. Studies reveal ROS concentration gradients induce T cell functional heterogeneity: high ROS levels inhibit immune responses by disrupting T cell receptor–major histocompatibility complex (MHC) interactions, whereas moderate levels enhance CTL cytotoxicity [585]. Preclinical data show chimeric antigen receptor T cell efficacy inversely correlates with mitochondrial oxidative stress, with catalase coadministration significantly improving therapeutic outcomes [586]. In ICI therapy, metformin reverses PD‐1/L1 antibody‐induced T cell exhaustion by modulating mitochondrial ROS metabolism [587]. Notably, ROS regulation of Tregs exhibits tissue specificity, with mitochondrial complex III activity determining their immunosuppressive potency in tumors [588]. Recent studies further demonstrate MDSCs suppress T cell proliferation via ROS‐mediated oxidative stress, a process reversible by antioxidants [589], providing mechanistic support for metabolic interventions.

Differential ferroptosis sensitivity among immune cells constitutes critical regulatory nodes in antitumor immunity. CD36‐mediated lipid uptake in CD8+ T cells exacerbates GPX4 inhibitor‐induced ferroptosis, fostering immunosuppression via reduced IFNγ secretion [590, 591]. Although NK cell ferroptosis mechanisms remain incompletely defined, LPO‐related oxidative stress impairs cytotoxicity by suppressing glucose metabolism [592]. DC dysfunction correlates with 12/15‐LO (12/15‐LOX)‐mediated oxidized lipid metabolism, which hinders antigen cross‐presentation via MHC I downregulation [593]. Intriguingly, NRF2 activation status determines DC functional fate under oxidative stress [593], suggesting therapeutic potential. These findings collectively underscore intricate crosstalk between ferroptosis‐related metabolic networks and immune cell functionality, with mechanistic elucidation poised to advance cancer immunotherapy strategies.

Innate lymphoid cell populations, including NK cells, mediate antitumor effects through perforin‐dependent cytotoxicity, TNF superfamily death receptors, and immune checkpoint molecules such as PD‐1 (PCD protein 1) [594]. Notably, PD‐1 shows constitutive absence in resting T lymphocytes but becomes markedly overexpressed in tumor‐infiltrating T cell subsets, suggesting tumor immune evasion through adaptive PD‐L1 expression [595, 596, 597]. Recent studies reveal the dual immunomodulatory nature of IFN signaling networks, which establish dynamic regulatory circuits between tumor and immune cells. These antagonistic molecular dialogues critically influence innate immune cytotoxicity. Clinical evidence demonstrates that PD‐1 pathway blockade effectively inhibits IFNγ signaling in breast cancer, melanoma, and CRC cells, thereby alleviating T cell exhaustion and enhancing CD8+ T lymphocyte‐mediated antitumor immunity [598]. However, the systemic response rates of immune checkpoint blockade therapy in most solid tumors remain suboptimal due to TME heterogeneity and host immune activation efficiency [599, 600].

Of particular interest, ferroptosis‐associated lipid peroxides serve as molecular signatures that enhance DC antigen capture and presentation, thereby activating CD8+ T cell‐mediated adaptive immunity. Tumor‐targeted delivery of RSL‐3 nanoparticles induces immunogenic cell death while stimulating CTL IFNγ secretion. This synergistic mechanism not only disrupts tumor lipid peroxide repair pathways but also achieves fourfold expansion of tumor‐infiltrating T cells following combined immune checkpoint intervention, significantly suppressing tumor progression and metastasis [601]. These findings highlight ferroptosis‐modulated combination strategies with immune checkpoint therapies as promising avenues for optimizing clinical translation in cancer immunotherapy.

Cancer immunotherapy achieves tumor intervention by modulating antitumor immune responses, with its core mechanism involving CD8+ T lymphocyte‐mediated tumor cell killing [602, 603]. Classical theory posits that activated CD8+ T cells primarily induce tumor cell death via the perforin‐granzyme system and Fas–FasL signaling pathway [604]. Notably, the JAK/STAT1 axis plays a pivotal role in IFNγ‐mediated immune regulation, participating not only in immune response modulation [605] but also in ferroptosis control through transcriptional regulation of solute carrier SLC7A11 Specifically, IFNγ secreted by CD8+ T cells enhances STAT1 binding to the SLC7A11 promoter, suppressing its transcription. Experimental evidence shows that STAT1 knockout in tumor cells blocks IFNγ‐induced SLC7A11 downregulation and significantly reduces RSL3‐triggered LPO and ferroptosis susceptibility [606]. Importantly, FINs like Erastin and RSL3 exhibit selective cytotoxicity toward tumor cells while sparing CD8+ T cells due to intrinsic resistance [607], suggesting novel opportunities for tumor‐specific therapies. Preclinical studies demonstrate synergistic enhancement of antitumor immunity and ferroptosis‐dependent tumor clearance through combined ICIs and FINs [606].

In radiotherapy, despite its long‐standing clinical utility [607, 608], therapeutic failure due to radioresistance remains a major challenge. Recent studies reveal close links between ferroptosis and radiosensitivity. Radiation induces KEAP1 mutations to upregulate SLC7A11 expression, conferring dual resistance to radiotherapy and ferroptosis [609]. Remarkably, radiation‐immunotherapy combinations enhance ferroptosis susceptibility, with cytoplasmic‐targeted radiotherapy showing synergy with FINs. This combinatorial approach achieves cell killing through glycolytic reprogramming and peroxide accumulation rather than conventional DNA damage mechanisms [610].

Mechanistic investigations demonstrate interconnected regulation between effector T cells and radiotherapy via ferroptosis pathways. Immunotherapy‐activated CD8+ T cells enhance tumor radiosensitivity by IFNγ‐driven LPO [611]. Radiation‐induced ataxia–telangiectasia mutated (ATM) activation inhibits SLC7A11 expression, causing impaired cystine uptake, reduced GSH synthesis, and elevated LPO that culminates in ferroptosis [612]. Additionally, the dynamic balance between radiation‐generated ROS and ferroptosis marker 4‐HNE proves critical, with SLC7A11‐ or GPX4‐targeting ferroptosis‐inducing agents (FINs) effectively reversing radioresistance [613]. Notably, ferroptosis regulatory mechanisms may influence radiotherapy‐associated complications like pulmonary fibrosis and hematopoietic damage [614, 615], providing crucial considerations for optimizing combination therapies.

The Warburg effect, a hallmark metabolic reprogramming phenomenon in tumor cells characterized by enhanced glucose uptake and aerobic glycolysis to sustain rapid proliferation, renders cancer cells particularly vulnerable to redox homeostasis disruption [616]. Crucially, the aberrant dependence on glucose and glutamine metabolism within the TME constitutes metabolic vulnerabilities, providing a theoretical foundation for selective targeting [617]. Studies demonstrate that mitochondrial metabolic stress significantly elevates ROS levels in T cells, with hypoxic conditions exacerbating T cell exhaustion through metabolic dysregulation. Modulating TME oxygenation and reducing intracellular ROS in T cells effectively preserves antitumor functionality, creating favorable conditions for combination immunotherapy [618].

Current therapeutic strategies exploiting these metabolic features focus on: (1) targeting SLC7A11 transporter activity to disrupt cystine metabolic homeostasis; (2) employing glucose metabolism inhibitors to selectively eliminate PPP‐dependent tumor cells; and (3) developing glutaminase inhibitors to block glutamine addiction. These interventions converge on disrupting tumor redox balance, ultimately inducing LPO cascades for ferroptosis‐specific elimination.

Recent studies have unveiled the unique role of ferroptosis in tumor immunomodulation, which not only exhibits direct immunogenic properties but also regulates neuroinflammatory responses to influence TME homeostasis [619]. TAMs, as key microenvironment modulators, exhibit high plasticity in polarization states: proinflammatory M1 phenotypes inhibit tumor growth and metastasis [620], while M2 phenotypes suppress antitumor immunity by secreting proangiogenic and immunosuppressive factors [621]. This phenotypic heterogeneity suggests that modulating macrophage polarization signaling to restore M1/M2 balance represents a novel therapeutic strategy.

Mechanistic investigations reveal that KRASG12D protein released by ferroptotic pancreatic cancer cells undergoes RAGE‐mediated macrophage uptake, activating STAT3‐dependent FAO pathways to promote M2 polarization [622]. Multiple technological platforms show potential for regulating this process: magnetic nanocarrier Fe_3_O_4_–SAS@PLT enables precise delivery of sulfasalazine to lesions [623]; combined regimens incorporating tumor‐derived microparticles, radiotherapy, PD‐1 antibodies, and TGF‐β inhibitors effectively reverse M2 polarization [624]; synergistic application of oxygen‐elevating photosensitizers and FINs significantly enhances LPO while promoting IFN‐γ secretion and immune microenvironment remodeling [625, 626]. Notably, M1 macrophages demonstrate superior IFN‐γ production capacity [627], and dual PD‐1/TGF‐β blockade not only strengthens M1 polarization but also induces tumor‐specific ferroptosis through Fenton reactions between elevated H2O2 levels in macrophages and metal ions released from nanoparticles [628]. These discoveries provide critical theoretical foundations for developing ferroptosis‐based combination therapies through macrophage phenotypic reprogramming.

Recent studies have elucidated the dual regulatory roles of ferroptosis in tumor immunomodulation, with its biological effects intricately linked to the dynamic release of DAMPs. When ferroptotic tumor cells release intracellular components, these normally sequestered molecules activate DC maturation and promote CD8+ T cell cross‐priming through PRR‐mediated mechanisms, accompanied by IFN‐γ secretion to initiate adaptive immunity [629, 630, 631]. Notably, the immunomodulatory effects of ferroptosis exhibit spatiotemporal specificity: in cardiac injury models, ferroptotic cells recruit neutrophils via the TLR4–TRIF signaling axis [632], demonstrating direct coupling between DAMP release and innate immune activation.

Within the TME, ferroptosis‐mediated DAMP release manifests multidimensional immunoregulatory properties. Phagocytic activity of bone marrow‐derived DCs is potently activated by key molecules such as calreticulin (CRT), HMGB1, and ATP released from ferroptotic cells [633]. Deeper mechanistic insights reveal that neutrophil tumor infiltration is dually regulated by ferroptosis‐modulated TLR4/TRIF/IFN‐I and Wnt signaling pathways, providing novel therapeutic avenues for targeting immunosuppressive microenvironments [634]. Particularly noteworthy, HMGB1—a critical chromatin‐binding protein involved in fundamental processes like DNA damage repair [635]—exhibits characteristic release patterns following FIN treatment, with its immune system interactions identified as pivotal determinants of therapeutic efficacy [636, 637].

Therapeutically, ferroptosis‐induced DAMP cascades present dual regulatory potential: excessive pathological DAMP accumulation may exacerbate tissue inflammation, while precisely controlled release kinetics can establish positive immunostimulatory feedback loops to suppress tumor growth [637]. This mechanistic duality provides a crucial framework for developing combination therapies leveraging immunogenic ferroptosis.

The differentiation plasticity of tumor cells exhibits significant correlation with therapeutic sensitivity, a biological characteristic that provides novel entry points for precision medicine. In melanoma, for instance, tumor heterogeneity manifests as differential ferroptosis responses among differentiation‐state subsets: TNFα‐ and IFNγ‐induced dedifferentiated subtypes demonstrate marked sensitivity to FINs, while melanocytic differentiation‐maintaining subtypes show substantial resistance [638]. This differentiation‐dependent susceptibility suggests that targeting cell fate determination pathways may enable precise therapeutic modulation.

Mechanistically, mesenchymal–phenotype tumor cells frequently undergo lipid metabolism reprogramming, with their therapeutic resistance correlating with upregulated lipid peroxidase activity and enhanced polyunsaturated lipid synthesis [639]. Two compound classes demonstrate selective lethality against these cells: FINs directly target LPO pathways, while statins impair the mevalonate pathway via HMG–CoA reductase inhibition, thereby depleting critical metabolites like coenzyme Q10. This metabolic vulnerability offers dual intervention strategies against mesenchymal‐state tumors.

Notably, the synergistic potential between Fe‐dependent cell death and other therapies is gaining attention. Although current evidence linking ferroptosis to immunotherapy predominantly originates from limited functional genomic studies [640, 641], and the pleiotropic functions of implicated genes may compromise data reliability, emerging research has uncovered its therapeutic potential. For example, FINs combined with radiotherapy enhance DNA damage effects [4], while differentiation state modulation synergizes with immune checkpoint blockade to overcome treatment resistance.

Integrating ferroptosis‐related strategies holds clinical significance based on tumor metabolic heterogeneity and differentiation plasticity. Key approaches include: spatiotemporal coordination of ferroptosis induction with immune checkpoint inhibition, differentiation state‐specific targeting of ferroptosis susceptibility, and combined metabolic stress with immune microenvironment remodeling. Establishing multidimensional intervention networks may overcome the limitations of monotherapies, opening new avenues for solid tumor treatment.

In the field of cancer therapeutics, radiotherapy has long been recognized as a cornerstone treatment modality [642, 643]. However, clinical observations reveal that therapeutic failure often correlates with tumor cell‐derived radioresistance, a phenomenon that not only diminishes treatment efficacy but also promotes distant metastasis. To address this clinical challenge, researchers have focused on exploring synergistic strategies combining radiotherapy with chemical sensitizers. Recent studies have uncovered intricate crosstalk between ferroptosis and ionizing radiation. Specifically, radiation‐induced KEAP1 mutations aberrantly activate the SLC7A11 signaling pathway, establishing dual resistance barriers against both ferroptosis and radiotherapy [560]. Notably, precise targeting of radiation fields to cytoplasmic regions rather than nuclei significantly enhances tumor cell susceptibility to FINs when combined with ICIs. This combinatorial approach operates through glycolytic reprogramming and activation of LPO cascades, fundamentally differing from traditional radiotherapy mechanisms dependent on DNA damage repair pathways [546].

Noncoding RNAs have emerged as master regulators of ferroptosis [644]. By targeting key ferroptosis drivers and suppressors, ncRNAs—miRNAs, lncRNAs, and circRNAs—dictate cellular susceptibility to LPO, offering novel therapeutic avenues.

miRNAs fine‐tune ferroptosis by silencing critical regulators. For instance, miR‐101‐3p and miR‐324‐3p directly suppress GPX4 [645, 646], the primary enzyme neutralizing lipid peroxides. Their downregulation in lung and breast cancer correlates with GPX4 upregulation, enabling ferroptosis evasion. Restoring miR‐101‐3p via nanoparticle delivery in lung cancer models reduces tumor growth by reactivating LPO. Conversely, miR‐4443 promotes cisplatin resistance in NSCLC by stabilizing FSP1 via METTL3‐mediated m6A modification, highlighting miRNAs as double‐edged swords in therapeutic contexts [647].

lncRNAs orchestrate ferroptosis through diverse mechanisms. The tumor‐suppressive lncRNA P53RRA (LINC00472) binds G3BP1, displacing p53 to the nucleus, where it represses SLC7A11 transcription. This depletes GSH, sensitizing lung cancer cells to erastin‐induced ferroptosis [648]. Conversely, oncogenic LINC00336 sponges miR‐6852 to stabilize CBS, enhancing GSH synthesis and ferroptosis resistance. MT1DP, a pseudogene‐derived lncRNA, destabilizes NRF2 by stabilizing miR‐365a‐3p, which targets NRF2 mRNA. In NSCLC, folate‐coated nanoparticles codelivering MT1DP and erastin amplify LPO, demonstrating lncRNA‐based therapeutic synergy [649].

circRNAs, with their covalently closed structure, evade degradation and serve as robust miRNA sponges. circKDM4C, downregulated in acute myeloid leukemia, sequesters let‐7b‐5p to upregulate p53 and ACSL4, driving Fe accumulation and LPO [650]. Conversely, circEPSTI1 promotes cervical cancer progression by sponging miR‐375, miR‐409‐3p, and miR‐515‐5p, which collectively target SLC7A11. Silencing circEPSTI1 restores SLC7A11 suppression, re‐sensitizing cells to ferroptosis [651].

ncRNA‐based therapies face hurdles in delivery and specificity but hold transformative potential. Lipid nanoparticles encapsulating miR‐101‐3p or MT1DP/erastin complexes improve bioavailability and tumor targeting in preclinical models. CircRNAs, owing to their stability, are ideal candidates for engineered sponges targeting oncogenic miRNAs. Combining ncRNA therapeutics with existing treatments—such as sorafenib, which upregulates proferroptotic circRNAs like cIARS in hepatocellular carcinoma (HCC)—may overcome chemoresistance [652].

Despite progress, challenges persist. Off‐target effects and immune recognition of synthetic ncRNAs necessitate advanced delivery systems, such as antibody‐conjugated nanoparticles or exosomes. Additionally, the interplay between ferroptosis and immune checkpoints remains underexplored, though lncRNA signatures in head and neck cancer suggest synergistic potential with immunotherapy [653].

ncRNAs sit at the nexus of ferroptosis regulation, offering precision tools to reinstate this death pathway in therapy‐resistant cancers. As delivery technologies and combinatorial strategies evolve, ncRNA‐based interventions may transform ferroptosis from a biological concept into a clinical cornerstone, unlocking new dimensions in cancer treatment.

The biological value of ferroptosis in cancer therapy has become increasingly prominent, with its unique cell death mechanism offering novel strategies to overcome tumor resistance. Recent studies have revealed metabolically dependent vulnerability windows in tumor cells toward ferroptosis [654], providing theoretical support for targeted therapies. As the primary site of intracellular ROS generation, mitochondria play dual roles in ferroptosis regulation: serving as both the origin of oxidative stress and key executors of PCD. This dual functionality positions mitochondrial ferroptosis pathways as highly promising therapeutic targets.

Mitochondrial ferroptosis regulation involves complex networks, primarily comprising GPX4‐dependent and non‐GPX4‐dependent pathways. Mitochondrial‐specific GPX4 (mGPX4) constitutes a critical antioxidant defense barrier, while the DHODH–COQH2 axis represents an independent regulatory route. Notably, drug‐resistant tumor cells exhibit heightened sensitivity to GPX4‐mediated ferroptosis [655, 656]. Although mGPX4‐specific inhibitors remain elusive, pan‐GPX4 inhibitors demonstrate significant efficacy in solid tumor models such as melanoma [657] and RCC [658], suggesting broad applicability of this pathway. Intriguingly, dynamic equilibrium exists between mGPX4 and DHODH pathways: tumor cells counteract LPO induced by DHODH inhibition through mGPX4 upregulation, whereas cytosolic GPX4 lacks such compensatory capacity [659]. This pathway complementarity supports combination therapies, particularly in GPX4‐low tumors where DHODH inhibitors effectively trigger ferroptosis.

Emerging evidence indicates that isolated DHODH targeting can induce ferroptosis under specific conditions. Zhang et al. [660] discovered that manganese ion exposure downregulates DHODH expression via IFN‐I signaling, leading to mitochondrial ROS accumulation and ferroptosis induction—a process independent of known death mechanisms and unaccompanied by alterations in GPX4 or other pathway components. Mechanistic analyses confirm the central role of DHODH, as dihydroorotate supplementation or IFN‐I blockade reverses this effect. Similarly, benzene‐induced myelotoxicity has been linked to DHODH‐mediated ferroptosis [661], expanding the pathophysiological relevance of the pathway.

CoQ, a pivotal carrier in mitochondrial oxidative phosphorylation, exhibits bidirectional regulatory effects in ferroptosis. While mitochondria serve as the primary site of CoQ biosynthesis [662], CoQ concurrently participates in both the DHODH axis and FSP1–CoQ10 pathway. Exogenous CoQ supplementation effectively suppresses LPO and enhances cellular survival [663], underscoring the need for spatiotemporal specificity when targeting CoQ metabolism. From an energy metabolism perspective, mitochondria regulate ferroptosis‐associated enzyme activity through ATP supply, a process disrupted by electron transport chain inhibitors [664], revealing intricate links between metabolic remodeling and ferroptosis. Importantly, the unique sensitivity of mitochondrial membrane structures to LPO [665] provides a structural basis for therapeutic targeting. Although current translational research remains exploratory, precision intervention strategies exploiting mitochondrial ferroptosis vulnerabilities undoubtedly open new frontiers in cancer therapy.

#### 5.3.4 Overcoming Drug Resistance

Recent studies reveal that ferroptosis regulation mechanisms offer novel strategies to overcome tumor chemotherapy resistance. Current interventions primarily focus on modulating GPX4 function, Fe homeostasis, and LPO pathways. As a pivotal antioxidant enzyme, GPX4 synergizes with GSH to eliminate hydrogen peroxide and organic hydroperoxides [666, 667]. Multiple studies confirm that targeting this enzymatic system significantly enhances chemosensitivity. In GBM models, curcumin analogues reverse temozolomide resistance via ubiquitination‐mediated degradation of the androgen receptor, subsequently inhibiting GPX4 activity and inducing ferroptosis [668]. CRC research demonstrates that the KIF20A/NUAK1/PP1β signaling axis mediates oxaliplatin resistance through GPX4 regulation, with pathway blockade restoring drug sensitivity [669].

Fe metabolism dysregulation critically contributes to chemotherapy resistance. Dihydroartemisinin treatment in PDAC induces LIP accumulation, triggering ferroptosis to overcome cisplatin resistance [670]. LCN2 and divalent metal transporter 1 (DMT1), key Fe homeostasis regulators, drive chemotherapy resistance in colon and breast cancers, respectively. Targeting these proteins effectively restores drug sensitivity [671, 672]. Notably, xCT system inhibitors in GSH synthesis pathways deplete cysteine reserves to induce ferroptosis in head/neck and gastric cancers, providing innovative solutions for cisplatin resistance [673, 674].

While ICIs revolutionized cancer therapy, drug resistance remains challenging. Ferroptosis demonstrates potential to overcome treatment resistance by remodeling TME immune components. Tumor‐intrinsic mechanisms like MAPK signaling abnormalities and PTEN loss impair immunogenicity. Emerging evidence shows ferroptosis releases DAMPs, activating DCs to promote antitumor immunity [675]. Strikingly, SAPE‐OOH molecules specifically expressed on ferroptotic cells enhance macrophage phagocytosis via TLR2 signaling [676], suggesting ferroptosis may exert immune adjuvant effects.

In TME modulation, Tregs and TAMs drive immunosuppression. Experimental data reveal GPX4 deficiency induces Treg ferroptosis while promoting Th17 differentiation, significantly enhancing antitumor immunity [677]. M2 macrophages exhibit higher ferroptosis susceptibility than M1 subtypes, with selective M2 depletion alleviating immunosuppression [678]. TYRO3 receptor inhibitors dually regulate tumor cell ferroptosis and macrophage polarization to reverse PD‐1/PD‐L1 resistance [679].

These findings highlight that precise modulation of ferroptosis‐related pathways can multidimensionally reshape tumor immune landscapes. This provides theoretical foundations for developing novel combination therapies, particularly in overcoming ICI resistance. Future research should elucidate spatiotemporal relationships between ferroptosis and immune regulation, optimizing intervention specificity to achieve precision therapeutic breakthroughs.

#### 5.3.5 Emerging Technologies

Gene editing technologies, particularly the clustered regularly interspaced short palindromic repeats/CRISPR‐associated protein 9 (CRISPR/Cas9) system, are reshaping the paradigm of ferroptosis research, offering novel perspectives for precise regulation of this Fe‐dependent cell death mechanism and its translational applications. By targeting ferroptosis‐related genes such as GPX4, SLC7A11, and ACSL4, CRISPR technology has not only elucidated molecular regulatory networks but also driven therapeutic innovation [680]. For instance, CRISPR‐mediated GPX4 knockout selectively eliminates chemotherapy‐resistant cells in TNBC models [681], while SLC7A11 editing enhances ferroptosis sensitivity by suppressing system Xc^−^ function and exacerbating GSH depletion [680]. These findings provide molecular foundations for overcoming tumor resistance, though potential risks such as prometastatic inflammatory factor release require systematic evaluation [681].

The integration of high‐throughput CRISPR screening has accelerated the discovery of ferroptosis regulators. A landmark study employing genome‐wide CRISPR–Cas9 screening identified protein arginine methyltransferase 1 (PRMT1) as a critical ferroptosis promoter [682]. PRMT1 enhances PUFA peroxidation through methylation‐mediated regulation of lipid metabolism proteins [682]. This mechanism, validated in PDAC, suggests PRMT1 targeting as a novel strategy to enhance chemosensitivity. However, broad biological functions necessitate meticulous analysis of tissue‐specific effects to avoid off‐target consequences [682].

Synergistic innovations in nanotechnology and CRISPR are overcoming gene delivery barriers. Copper‐based nanocarriers exemplify this progress: by codelivering CRISPR–Cas9 and cuprous ions, this system achieves dual mechanisms—ATP7A knockdown induces intracellular copper accumulation, triggering Fenton reactions and oxidative stress, while synchronized GPX4 inhibition amplifies LPO [683]. In pancreatic cancer mouse models, this strategy significantly suppresses tumor growth without systemic toxicity, demonstrating clinical potential [683]. Similarly, lipid‐encapsulated CRISPR systems enable efficient liver‐targeted editing, modulating hepcidin expression to alleviate Fe overload, offering new therapeutic approaches for ferroptosis‐driven nonalcoholic steatohepatitis [684].

The ferroptosis‐immunity interplay opens new dimensions for CRISPR‐based combination therapies. CRISPR‐mediated knockout of FSP1 in tumor cells not only enhances ferroptosis susceptibility but also activates DCs through DAMP release, improving anti‐PD‐1 immunotherapy response rates [681]. This “double‐edged sword” effect underscores the importance of spatiotemporal precision: premature ferroptosis induction may deplete immune cells, while timely triggering can remodel immunosuppressive microenvironments [681]. Thus, developing regulatable CRISPR systems represents a critical research frontier.

Despite promising prospects, CRISPR‐mediated ferroptosis regulation faces multiple challenges. Off‐target effects may disrupt Fe metabolism genes, interfering with physiological processes [683], while delivery limitations hinder effective editing in protected tissues like the blood–brain barrier [684]. Addressing these requires interdisciplinary collaboration: single‐cell multiomics can reveal heterogeneous ferroptosis regulatory networks [681], while novel delivery vehicles may enhance targeting specificity [683]. Furthermore, identifying ferroptosis‐specific biomarkers is crucial for patient stratification and treatment monitoring [684].

Looking ahead, gene editing technologies will accelerate the translation of ferroptosis research from mechanistic exploration to clinical application. Integrating spatial metabolomics, organoid models, and AI‐driven editing design may decode dynamic ferroptosis regulation maps, enabling personalized therapies based on molecular subtyping. Breakthroughs in this field promise not only to deepen understanding of cell death mechanisms but also to deliver transformative treatments for cancer, neurodegenerative diseases, and other intractable disorders.

## 6 Ferroptosis at the Nexus of Cancer and Inflammatory Diseases

### 6.1 Common Drivers and Regulatory Mechanisms

Ferroptosis has emerged as a crucial interface linking oncogenic signaling and inflammatory circuits, expanding its role beyond its traditional classification as a form of PCD. This Fe‐dependent necrotic cell death pathway, distinguished by its unique LPO features, creates novel therapeutic vulnerabilities in treatment‐resistant cancers and chronic inflammatory diseases. Increasing evidence positions ferroptosis as a central molecular mechanism that coordinates cellular transformation and remodeling of the inflammatory microenvironment—a duality that presents both significant challenges and exciting opportunities for therapeutic intervention.

The intersection between ferroptosis and inflammatory signaling is most prominently manifested in the generation of an eicosanoid storm. During ferroptotic membrane collapse, the released AA fuels the COX‐2/PTGS2 and 15‐LOX pathways [685], producing PGE2 and other proinflammatory lipid mediators that amplify NLRP3 inflammasome activation [686]. Paradoxically, this inflammatory feedback loop creates a protumorigenic microenvironment during early carcinogenesis [687], while simultaneously sensitizing established tumors to immunogenic cell death [688]. For example, IFNγ released by CD8+ T cells suppresses SLC7A11 expression in tumor cells [551], linking immune surveillance to ferroptotic clearance—a mechanism exploited in ICI therapies. In contrast, IL‐4‐polarized TAMs secrete lactoferrin to sequester Fe [689], thereby creating ferroptosis‐resistant niches that promote metastasis.

In oncogenic contexts, tumor suppressors like p53 and BAP1 exhibit dual regulation of ferroptosis. Cytoplasmic p53 variants promote the suppression of SLC7A11 through USP7‐mediated deubiquitination [690], while nuclear p53 transactivates SAT1 to upregulate ALOX15 [691], yielding cell state‐dependent ferroptotic outcomes. The chromatin remodeling activity of BAP1 directly represses SLC7A11 transcription by removing H2AK119ub1 [692], making BAP1‐mutant cancers hypersensitive to GPX4 inhibitors. Notably, these tumor‐suppressive mechanisms are intertwined with inflammatory signaling: p53 activation induces the senescence‐associated secretory phenotype to prime neighboring cells for ferroptosis [693], while BAP1 deficiency upregulates CXCL chemokines, recruiting immunosuppressive myeloid cells [692].

### 6.2 Inflammation and Ferroptosis in the TME

Ferroptosis in the TME can both initiate and exacerbate inflammatory responses, influencing tumor progression and immune modulation. Tumor cells undergoing ferroptosis release DAMPs, such as HMGB1 and ATP, which trigger immune activation through the cGAS–STING pathway [694]. This signaling cascade promotes DC maturation, macrophage phagocytosis, and CD8+ T cell infiltration into the tumor site, leading to tumor regression in preclinical models [695, 696, 697].

However, ferroptosis can also contribute to a protumorigenic inflammatory environment. In pancreatic cancer, ferroptotic cells release KRAS G12D, which polarizes macrophages to an M2 phenotype through STAT3 signaling, promoting immune suppression and tumor progression [698, 699, 700, 701]. This paradoxical role of ferroptosis highlights the complex relationship between ferroptosis‐induced inflammation and immune regulation within the TME.

While ferroptosis can trigger immune activation, it can also drive immunosuppressive inflammation. For example, ferroptotic cells release oncoproteins that activate STAT3 signaling in macrophages, promoting a protumor M2 polarization that suppresses CTLs and DCs. Additionally, ferroptosis in hepatocellular carcinoma leads to an imbalance in γδ T cells, further promoting tumor progression [702].

The release of inflammatory mediators like PGE2 during ferroptosis can further exacerbate immune suppression, promoting the accumulation of M2 macrophages and immunosuppressive TME formation [700, 701, 703]. These findings underscore the need for precise modulation of ferroptosis in therapeutic strategies, to avoid amplifying tumor‐promoting inflammation.

The temporal dynamics of DAMP release during ferroptosis are crucial in determining the inflammatory outcome. Early‐stage ferroptosis (1–3 h) is associated with the release of immunostimulatory molecules such as HMGB1 and ATP, which can induce DC maturation and generate antitumor immune memory [704]. However, late‐stage ferroptotic cells (after 24 h) lose this capacity, signaling the need for timely and controlled ferroptosis induction to optimize immunotherapy outcomes.

Ferroptosis‐associated DAMPs also play a role in the regulation of macrophage polarization. In pancreatic cancer models, ferroptotic cells release 8‐OHG, which promotes M2 macrophage polarization and contributes to an immunosuppressive TME via the STING pathway [698, 699]. Similarly, GPX4‐deficient mice with ferroptosis exhibit increased MDSC infiltration and PD‐L1 upregulation, forming an immune escape loop [705]. The temporal aspects of ferroptosis and DAMP release illustrate how the immune environment in the TME can shift, further complicating therapeutic interventions aimed at ferroptosis modulation.

### 6.3 Cross‐Over and Insights into Therapeutic Strategies

Pharmacological targeting of ferroptosis nodes demonstrates context‐dependent efficacy. System xCT inhibitors [706] synergize with anti‐PD1 therapy by reversing T cell exhaustion in “cold” tumors [707], whereas GPX4 degraders eliminate apoptosis‐resistant mesenchymal cancer stem cells [708]. Intriguingly, FINs exhibit paradoxical anti‐inflammatory effects in sterile inflammation models: liproxstatin‐1 attenuates IRI via 12/15‐LOX inhibition [709], while Fe chelators disrupt ASC oligomerization in NLRP3 inflammasomes [710]. Emerging nanoparticle platforms codeliver ferroptosis agonists (Fe^2^⁺, RSL3) and COX‐2 inhibitors to simultaneously reduce tumor burden and neutralize therapy‐induced inflammation [545].

Despite these advances, critical knowledge gaps remain in understanding the spatiotemporal regulation of ferroptosis within tumor immune ecosystems. The dual roles of LPO products—as DAMPs that stimulate antitumor immunity while also promoting prometastatic inflammation [711]—necessitate single‐cell resolution mapping across disease stages. Biomarker development is urgently needed: while ACSL4 expression [712] and 4‐HNE adducts show promise as diagnostic markers [713], their predictive value for therapeutic responses varies across cancer subtypes [714]. Moreover, tumor metabolic plasticity, through pathways like transsulfuration [715] or DHODH‐mediated ubiquinol synthesis [716], highlights the need for next‐generation FINs with orthogonal targeting mechanisms [717].

The complex interplay of ferroptosis, inflammation, and tumorigenesis calls for paradigm shifts in therapeutic approaches. As mechanistic studies continue to uncover how LPO signatures are interpreted by immune receptors and oncogenic transcription factors [718], future strategies must evolve from indiscriminate ROS generation to the precise modulation of membrane lipidomes and Fe chaperone networks [719]. Overcoming current challenges will require innovative advancements in chemical biology, spatial metabolomics, and immune microenvironment modeling. By bridging molecular mechanisms with system‐level pathophysiology, ferroptosis modulation has the potential to redefine therapeutic paradigms in cancer immunotherapy and the management of chronic inflammatory diseases.

## 7 Conclusion and Prospective

Ferroptosis, an Fe‐dependent form of RCD driven by LPO, has emerged as a pivotal mechanism linking cancer and inflammatory diseases [31]. This review has illuminated its dual roles—eliminating malignant cells while exacerbating tissue damage—revealing a therapeutic challenge that demands precision modulation. The path forward hinges on resolving fundamental dichotomies: how to harness the tumor‐suppressive potential of ferroptosis without triggering inflammatory collateral damage, how to overcome resistance mechanisms without disrupting systemic redox balance, and how to leverage immunomodulatory effects without perpetuating immunosuppression.

In cancer therapy, the appeal of ferroptosis induction lies in its ability to target resistant cell populations, yet its inflammatory consequences risk fueling protumorigenic microenvironments. However, its inflammatory consequences may paradoxically promote immunosuppressive microenvironments that facilitate metastasis, thereby undermining its therapeutic benefits [720]. Emerging strategies to achieve selectivity—such as targeted delivery systems or microenvironment‐sensitive inhibitors—may help minimize off‐target effects. However, these approaches must contend with the metabolic adaptability of tumors, where alternate pathways can compensate for blocked ferroptosis defenses, necessitating combinatorial approaches. Parallel challenges arise in inflammatory diseases, where ferroptosis inhibition could mitigate tissue damage but might inadvertently support abnormal cell survival. Parallel challenges arise in chronic inflammatory diseases, where ferroptosis‐induced immune cell death—especially of macrophages—can exacerbate tissue injury, while its inhibition may promote the survival of deleterious or premalignant cells [721]. Insights into cell‐type‐specific susceptibility thresholds offer a potential roadmap for tissue‐selective interventions.

The intersection of ferroptosis with immune regulation reveals further complexities. While certain immune signals promote tumor ferroptosis, they may simultaneously trigger resistant immune cell subsets that drive fibrosis or chronic inflammation. These contradictions reflect the context‐dependent outcomes of ferroptosis within diverse tissue environments and disease states [722]. This paradox underscores the need for precise timing in therapeutic interventions, where staged regimens could help separate beneficial effects from harmful cascades. Similarly, interactions between ferroptosis and immune checkpoint pathways suggest that combination therapies may require careful patient selection based on microenvironmental features.

To fully realize the therapeutic potential of ferroptosis while addressing these challenges, several key research directions must be pursued. First, precision medicine approaches are essential for identifying biomarkers that predict sensitivity or resistance to ferroptosis induction. By leveraging multiomics analyses and machine learning, researchers can stratify patients who would benefit most from ferroptosis‐targeted therapies, enabling tailored treatment strategies. This personalized approach is crucial given the context‐dependent nature of ferroptosis effects. Second, the integration of FINs with immunotherapy and other treatment modalities holds significant promise for overcoming therapy resistance. Understanding the synergistic effects of these combinations will be vital for optimizing treatment efficacy and improving patient outcomes. Third, continued exploration of novel ferroptosis modulators, particularly those derived from natural products, is necessary. These agents must be designed with improved specificity and reduced off‐target effects to maximize therapeutic benefit while minimizing toxicity. Fourth, advancing ferroptosis‐based therapies into clinical trials is a priority. This involves validating preclinical findings in human studies and addressing safety concerns, particularly the delicate balance between inducing ferroptosis in tumor cells and mitigating excessive inflammation. Finally, a comprehensive understanding of the regulatory networks involving ferroptosis, including its interactions with other cell death pathways, metabolism, and immune signaling, is needed. Multiomics approaches combined with computational modeling can provide an integrated view of ferroptosis regulation, facilitating the development of holistic therapeutic strategies.

Ultimately, the duality of ferroptosis reflects the interconnected biology of cancer and inflammation—mastering its therapeutic potential will require not just molecular precision but systems‐level understanding. By grounding discovery in clinically relevant models and addressing the dynamic interplay between ferroptosis and immunity, we can develop strategies that transform this double‐edged pathway into a targeted weapon against treatment‐resistant diseases.

## Author Contributions

G.S. and J.L. contributed equally to this work and share first authorship. G.S. and J.L. conceptualized and designed the study. Y.W. and F.D. conducted the literature review and contributed to manuscript drafting. J.L. and Z.D. critically revised the manuscript for intellectual content. G.S. and J.L. wrote the initial draft, while F.D. and Z.D. refined the manuscript and provided additional insights. Z.D. supervised the study and approved the final version for submission. All authors have read and approved the final manuscript.

## Ethics Statement

The authors have nothing to report.

## Conflicts of Interest

The authors declare no conflicts of interest.

## Data Availability

No new data were generated or analyzed in this study. Data sharing is not applicable to this article.

## References

[1] X. Jin , J. Tang , X. Qiu , et al., “Ferroptosis: Emerging mechanisms, biological function, and therapeutic potential in cancer and inflammation,” Cell Death Discov 10 (2024): 45. 38267442 10.1038/s41420-024-01825-7 PMC10808233

[2] S. J. Dixon , K. M. Lemberg , M. R. Lamprecht , et al., “Ferroptosis: An iron‐dependent form of nonapoptotic cell death,” Cell 149, no. 5 (2012): 1060‐1072. 22632970 10.1016/j.cell.2012.03.042 PMC3367386

[3] Q. Zhou , Y. Meng , D. Li , et al., “Ferroptosis in cancer: From molecular mechanisms to therapeutic strategies,” Signal Transduct Target Ther 9, no. 1 (2024): 55. 38453898 10.1038/s41392-024-01769-5 PMC10920854

[4] X. Chen , R. Kang , G. Kroemer , and D. Tang , “Ferroptosis in infection, inflammation, and immunity,” Journal of Experimental Medicine 218, no. 6 (2021): e20210518. 33978684 10.1084/jem.20210518 PMC8126980

[5] Q. Dang , Z. Sun , Y. Wang , L. Wang , Z. Liu , and X. Han , “Ferroptosis: A double‐edged sword mediating immune tolerance of cancer,” Cell death & disease 13, no. 11 (2022): 925. 36335094 10.1038/s41419-022-05384-6 PMC9637147

[6] H. Zhao , C. Tang , M. Wang , H. Zhao , and Y. Zhu , “Ferroptosis as an emerging target in rheumatoid arthritis,” Frontiers in immunology 14 (2023): 1260839. 37928554 10.3389/fimmu.2023.1260839 PMC10620966

[7] T. Zhao , Q. Yang , Y. Xi , et al., “Ferroptosis in Rheumatoid Arthritis: A Potential Therapeutic Strategy,” Frontiers in immunology 13 (2022): 779585. 35185879 10.3389/fimmu.2022.779585 PMC8847160

[8] X. Jiang , B. R. Stockwell , and M. Conrad , “Ferroptosis: Mechanisms, biology and role in disease,” Nature Reviews Molecular Cell Biology 22, no. 4 (2021): 266‐282. 33495651 10.1038/s41580-020-00324-8 PMC8142022

[9] A. M. Pisoschi and A. Pop , “The role of antioxidants in the chemistry of oxidative stress: A review,” European Journal of Medicinal Chemistry 97 (2015): 55‐74. 25942353 10.1016/j.ejmech.2015.04.040

[10] X. Chen , R. Kang , G. Kroemer , and D. Tang , “Broadening horizons: The role of ferroptosis in cancer,” Nature reviews Clinical oncology 18, no. 5 (2021): 280‐296. 10.1038/s41571-020-00462-0 33514910

[11] Y. Mou , J. Wang , J. Wu , et al., “Ferroptosis, a new form of cell death: Opportunities and challenges in cancer,” J Hematol OncolJ Hematol Oncol 12, no. 1 (2019): 34. 30925886 10.1186/s13045-019-0720-y PMC6441206

[12] M. P. Horowitz and J. T. Greenamyre , “Mitochondrial Iron Metabolism and Its Role in Neurodegeneration,” J Alzheimers Dis JAD 20, no. Suppl 2 (2010): S551‐S568. 20463401 10.3233/JAD-2010-100354 PMC3085540

[13] A. Pefanis , F. L. Ierino , J. M. Murphy , and P. J. Cowan , “Regulated necrosis in kidney ischemia‐reperfusion injury,” Kidney International 96, no. 2 (2019): 291‐301. 31005270 10.1016/j.kint.2019.02.009

[14] A. M. Pisoschi , A. Pop , F. Iordache , L. Stanca , G. Predoi , and A. I. Serban , “Oxidative stress mitigation by antioxidants—An overview on their chemistry and influences on health status,” European Journal of Medicinal Chemistry 209 (2021): 112891. 33032084 10.1016/j.ejmech.2020.112891

[15] B. R. Stockwell , J. P. F. Angeli , H. Bayir , et al., “Ferroptosis: A regulated cell death nexus linking metabolism, redox biology, and disease,” Cell 171, no. 2 (2017): 273‐285. 28985560 10.1016/j.cell.2017.09.021 PMC5685180

[16] W. Wang , M. Green , J. E. Choi , et al., “CD8+ T cells regulate tumour ferroptosis During cancer immunotherapy,” Nature 569, no. 7755 (2019): 270‐274. 31043744 10.1038/s41586-019-1170-y PMC6533917

[17] J. P. Friedmann Angeli , D. V. Krysko , and M. Conrad , “Ferroptosis at the crossroads of cancer‐acquired drug resistance and immune evasion,” Nature Reviews Cancer 19, no. 7 (2019): 405‐414. 31101865 10.1038/s41568-019-0149-1

[18] D. Liang , A. M. Minikes , and X. Jiang , “Ferroptosis at the intersection of lipid metabolism and cellular signaling,” Molecular Cell 82, no. 12 (2022): 2215‐2227. 35390277 10.1016/j.molcel.2022.03.022 PMC9233073

[19] H. Eagle , “Nutrition needs of mammalian cells in tissue culture,” Science 122, no. 3168 (1955): 501‐514. 13255879 10.1126/science.122.3168.501

[20] H. Eagle , “The Specific Amino Acid Requirements of a Human Carcinoma Cell (Strain Hela) in Tissue Culture,” Journal of Experimental Medicine 102, no. 1 (1955): 37‐48. 14392239 10.1084/jem.102.1.37 PMC2136494

[21] J. R. Mitchell , D. J. Jollow , W. Z. Potter , J. R. Gillette , and B. B. Brodie , “Acetaminophen‐induced hepatic necrosis. IV. Protective role of glutathione,” Journal of Pharmacology and Experimental Therapeutics 187, no. 1 (1973): 211‐217. 4746329

[22] S. Bannai , H. Tsukeda , and H. Okumura , “Effect of antioxidants on cultured human diploid fibroblasts exposed to cystine‐free medium,” Biochemical and Biophysical Research Communications 74, no. 4 (1977): 1582‐1588. 843380 10.1016/0006-291x(77)90623-4

[23] M. Yonezawa , S. A. Back , X. Gan , P. A. Rosenberg , and J. J. Volpe , “Cystine deprivation induces oligodendroglial death: Rescue by free radical scavengers and by a diffusible glial factor,” Journal of Neurochemistry 67, no. 2 (1996): 566‐573. 8764581 10.1046/j.1471-4159.1996.67020566.x

[24] S. Tan , D. Schubert , and P. Maher , “Oxytosis: A novel form of programmed cell death,” Current Topics in Medicinal Chemistry 1, no. 6 (2001): 497‐506. 11895126 10.2174/1568026013394741

[25] S. Mercille and B. Massie , “Induction of apoptosis in nutrient‐deprived cultures of hybridoma and myeloma cells,” Biotechnology and Bioengineering 44, no. 9 (1994): 1140‐1154. 18623032 10.1002/bit.260440916

[26] S. Christgen , R. E. Tweedell , and T. D. Kanneganti , “Programming inflammatory cell death for therapy,” Pharmacology & Therapeutics 232 (2022): 108010. 34619283 10.1016/j.pharmthera.2021.108010 PMC8930427

[27] L. Galluzzi , I. Vitale , S. A. Aaronson , et al., “Molecular mechanisms of cell death: Recommendations of the Nomenclature Committee on Cell Death 2018,” Cell Death and Differentiation 25, no. 3 (2018): 486‐541. 29362479 10.1038/s41418-017-0012-4 PMC5864239

[28] D. R. Green and B. Victor , “The pantheon of the fallen: Why are there so many forms of cell death?,” Trends in Cell Biology 22, no. 11 (2012): 555‐556. 22995729 10.1016/j.tcb.2012.08.008 PMC3568685

[29] H. O. Fearnhead , P. Vandenabeele , and T. Vanden Berghe , “How do we fit ferroptosis in the family of regulated cell death?,” Cell Death and Differentiation 24, no. 12 (2017): 1991‐1998. 28984871 10.1038/cdd.2017.149 PMC5686356

[30] S. J. Dixon , “Ferroptosis: Bug or feature?,” Immunological Reviews 277, no. 1 (2017): 150‐157. 28462529 10.1111/imr.12533

[31] S. J. Dixon and J. A. Olzmann , “The cell biology of ferroptosis,” Nature Reviews Molecular Cell Biology 25, no. 6 (2024): 424‐442. 38366038 10.1038/s41580-024-00703-5 PMC12187608

[32] D. Tang , X. Chen , R. Kang , and G. Kroemer , “Ferroptosis: Molecular mechanisms and health implications,” Cell Research 31, no. 2 (2021): 107‐125. 33268902 10.1038/s41422-020-00441-1 PMC8026611

[33] N. KC and F. Ka , “Apoptosis, Pyroptosis, and Necroptosis‐Oh My! The Many Ways a Cell Can Die,” Journal of Molecular Biology 434, no. 4 (2022): 167378. 34838807 10.1016/j.jmb.2021.167378

[34] M. S. D'Arcy , “Cell death: A review of the major forms of apoptosis, necrosis and autophagy,” Cell Biology International 43, no. 6 (2019): 582‐592. 30958602 10.1002/cbin.11137

[35] W. Gao , X. Wang , Y. Zhou , X. Wang , and Y. Yu , “Autophagy, ferroptosis, pyroptosis, and necroptosis in tumor immunotherapy,” Signal Transduct Target Ther 7, no. 1 (2022): 196. 35725836 10.1038/s41392-022-01046-3 PMC9208265

[36] J. Yan , P. Wan , S. Choksi , and Z. G. Liu , “Necroptosis and tumor progression,” Trends in cancer 8, no. 1 (2022): 21‐27. 34627742 10.1016/j.trecan.2021.09.003 PMC8702466

[37] D. Bertheloot , E. Latz , and B. S. Franklin , “Necroptosis, pyroptosis and apoptosis: An intricate game of cell death,” Cell Mol Immunol 18, no. 5 (2021): 1106‐1121. 33785842 10.1038/s41423-020-00630-3 PMC8008022

[38] H. Han , G. Zhang , X. Zhang , and Q. Zhao , “Nrf2‐mediated ferroptosis inhibition: A novel approach for managing inflammatory diseases,” Inflammopharmacology 32, no. 5 (2024): 2961‐2986. 39126567 10.1007/s10787-024-01519-7

[39] S. O. Vasudevan , B. Behl , and V. A. Rathinam , “Pyroptosis‐induced inflammation and tissue damage,” Seminars in Immunology 69 (2023): 101781. 37352727 10.1016/j.smim.2023.101781 PMC10598759

[40] P. Yu , X. Zhang , N. Liu , L. Tang , C. Peng , and X. Chen , “Pyroptosis: Mechanisms and diseases,” Signal Transduct Target Ther 6, no. 1 (2021): 128. 33776057 10.1038/s41392-021-00507-5 PMC8005494

[41] S. Liu , S. Yao , H. Yang , S. Liu , and Y. Wang , “Autophagy: Regulator of cell death,” Cell death & disease 14, no. 10 (2023): 648. 37794028 10.1038/s41419-023-06154-8 PMC10551038

[42] J. Debnath , N. Gammoh , and K. M. Ryan , “Autophagy and autophagy‐related pathways in cancer,” Nature Reviews Molecular Cell Biology 24, no. 8 (2023): 560‐575. 36864290 10.1038/s41580-023-00585-z PMC9980873

[43] N. Mizushima and M. Komatsu , “Autophagy: Renovation of cells and tissues,” Cell 147, no. 4 (2011): 728‐741. 22078875 10.1016/j.cell.2011.10.026

[44] D. Glick , S. Barth , and K. F. Macleod , “Autophagy: Cellular and molecular mechanisms,” Journal of Pathology 221, no. 1 (2010): 3‐12. 20225336 10.1002/path.2697 PMC2990190

[45] T. V. Berghe , A. Linkermann , S. Jouan‐Lanhouet , H. Walczak , and P. Vandenabeele , “Regulated necrosis: The expanding network of non‐apoptotic cell death pathways,” Nature Reviews Molecular Cell Biology 15, no. 2 (2014): 135‐147. 24452471 10.1038/nrm3737

[46] H. Wang , C. Liu , Y. Zhao , and G. Gao , “Mitochondria regulation in ferroptosis,” European Journal of Cell Biology 99, no. 1 (2020): 151058. 31810634 10.1016/j.ejcb.2019.151058

[47] J. P. F. Angeli , M. Schneider , B. Proneth , et al., “Inactivation of the ferroptosis regulator Gpx4 triggers acute renal failure in mice,” Nature Cell Biology 16, no. 12 (2014): 1180‐1191. 25402683 10.1038/ncb3064 PMC4894846

[48] L. J. Su , J. H. Zhang , H. Gomez , et al., “Reactive Oxygen Species‐Induced Lipid Peroxidation in Apoptosis, Autophagy, and Ferroptosis,” Oxid Med Cell Longev 2019 (2019): 5080843. 31737171 10.1155/2019/5080843 PMC6815535

[49] G. C. Forcina and S. J. Dixon , “GPX4 at the Crossroads of Lipid Homeostasis and Ferroptosis,” Proteomics 19, no. 18 (2019): e1800311. 30888116 10.1002/pmic.201800311

[50] M. Matsushita , S. Freigang , C. Schneider , M. Conrad , G. W. Bornkamm , and M. Kopf , “T cell lipid peroxidation induces ferroptosis and prevents immunity to infection,” Journal of Experimental Medicine 212, no. 4 (2015): 555‐568. 25824823 10.1084/jem.20140857 PMC4387287

[51] E. J. Jang , D. H. Kim , B. Lee , et al., “Activation of proinflammatory signaling by 4‐hydroxynonenal‐Src adducts in aged kidneys,” Oncotarget 7, no. 32 (2016): 50864‐50874. 27472463 10.18632/oncotarget.10854 PMC5239442

[52] R. Kang , R. Chen , Q. Zhang , et al., “HMGB1 in health and disease,” Molecular Aspects of Medicine 40 (2014): 1‐116. 25010388 10.1016/j.mam.2014.05.001 PMC4254084

[53] Q. Wen , J. Liu , R. Kang , B. Zhou , and D. Tang , “The release and activity of HMGB1 in ferroptosis,” Biochemical and Biophysical Research Communications 510, no. 2 (2019): 278‐283. 30686534 10.1016/j.bbrc.2019.01.090

[54] R. A. DeBose‐Boyd , “Significance and regulation of lipid metabolism,” Seminars in cell & developmental biology 81 (2018): 97. 29246858 10.1016/j.semcdb.2017.12.003

[55] W. S. Yang , K. J. Kim , M. M. Gaschler , M. Patel , M. S. Shchepinov , and B. R. Stockwell , “Peroxidation of polyunsaturated fatty acids by lipoxygenases drives ferroptosis,” PNAS 113, no. 34 (2016): E4966‐E4975. 27506793 10.1073/pnas.1603244113 PMC5003261

[56] S. Doll , B. Proneth , Y. Y. Tyurina , et al., “Acsl4 Dictates Ferroptosis Sensitivity by Shaping Cellular Lipid Composition,” Nature Chemical Biology 13, no. 1 (2017): 91‐98. 27842070 10.1038/nchembio.2239 PMC5610546

[57] V. E. Kagan , G. Mao , F. Qu , et al., “Oxidized Arachidonic/Adrenic Phosphatidylethanolamines Navigate Cells to Ferroptosis,” Nature Chemical Biology 13, no. 1 (2017): 81‐90. 27842066 10.1038/nchembio.2238 PMC5506843

[58] S. E. Wenzel , Y. Y. Tyurina , J. Zhao , et al., “PEBP1 Wardens Ferroptosis by Enabling Lipoxygenase Generation of Lipid Death Signals,” Cell 171, no. 3 (2017): 628‐641. e26. 29053969 10.1016/j.cell.2017.09.044 PMC5683852

[59] M. Conrad and D. A. Pratt , “The chemical basis of ferroptosis,” Nature Chemical Biology 15, no. 12 (2019): 1137‐1147. 31740834 10.1038/s41589-019-0408-1

[60] L. Magtanong , P. J. Ko , M. To , et al., “Exogenous Monounsaturated Fatty Acids Promote a Ferroptosis‐Resistant Cell State,” Cell Chem Biol 26, no. 3 (2019): 420‐432. e9. 30686757 10.1016/j.chembiol.2018.11.016 PMC6430697

[61] R. Shah , M. S. Shchepinov , and D. A. Pratt , “Resolving the Role of Lipoxygenases in the Initiation and Execution of Ferroptosis,” ACS Cent Sci 4, no. 3 (2018): 387‐396. 29632885 10.1021/acscentsci.7b00589 PMC5879472

[62] S. Khanna , S. Roy , H. Ryu , et al., “Molecular basis of vitamin E action: Tocotrienol modulates 12‐lipoxygenase, a key mediator of glutamate‐induced neurodegeneration,” Journal of Biological Chemistry 278, no. 44 (2003): 43508‐43515. 12917400 10.1074/jbc.M307075200 PMC1910692

[63] J. Liu , R. Kang , and D. Tang , “Signaling pathways and defense mechanisms of ferroptosis,” Febs Journal 289, no. 22 (2022): 7038‐7050. 34092035 10.1111/febs.16059

[64] R. P. L. van Swelm , J. F. M. Wetzels , and D. W. Swinkels , “The multifaceted role of iron in renal health and disease,” Nature Reviews Nephrology 16, no. 2 (2020): 77‐98. 31554933 10.1038/s41581-019-0197-5

[65] M. Gao , P. Monian , N. Quadri , R. Ramasamy , and X. Jiang , “Glutaminolysis and Transferrin Regulate Ferroptosis,” Molecular Cell 59, no. 2 (2015): 298‐308. 26166707 10.1016/j.molcel.2015.06.011 PMC4506736

[66] Z. Wang , Y. Ding , X. Wang , et al., “Pseudolaric acid B triggers ferroptosis in glioma cells via activation of Nox4 and inhibition of xCT,” Cancer Letters 428 (2018): 21‐33. 29702192 10.1016/j.canlet.2018.04.021

[67] W. Luo , J. Wang , W. Xu , et al., “LncRNA RP11‐89 facilitates tumorigenesis and ferroptosis resistance Through PROM2‐activated iron export by sponging miR‐129‐5p in bladder cancer,” Cell death & disease 12, no. 11 (2021): 1043. 34728613 10.1038/s41419-021-04296-1 PMC8563982

[68] D. L. Zhang , M. C. Ghosh , and T. A. Rouault , “The physiological functions of iron regulatory proteins in iron homeostasis—an update,” Frontiers in pharmacology 5 (2014): 124. 24982634 10.3389/fphar.2014.00124 PMC4056636

[69] H. Kawabata , “Transferrin and transferrin receptors update,” Free Radic Biol Med 133 (2019): 46‐54. 29969719 10.1016/j.freeradbiomed.2018.06.037

[70] W. Hou , Y. Xie , X. Song , et al., “Autophagy promotes ferroptosis by degradation of ferritin,” Autophagy 12, no. 8 (2016): 1425‐1428. 27245739 10.1080/15548627.2016.1187366 PMC4968231

[71] M. Gao , P. Monian , Q. Pan , W. Zhang , J. Xiang , and X. Jiang , “Ferroptosis is an autophagic cell death process,” Cell Research 26, no. 9 (2016): 1021‐1032. 27514700 10.1038/cr.2016.95 PMC5034113

[72] Y. Bai , L. Meng , L. Han , et al., “Lipid storage and lipophagy regulates ferroptosis,” Biochemical and Biophysical Research Communications 508, no. 4 (2019): 997‐1003. 30545638 10.1016/j.bbrc.2018.12.039

[73] B. Zhou , J. Liu , R. Kang , D. J. Klionsky , G. Kroemer , and D. Tang , “Ferroptosis is a type of autophagy‐dependent cell death,” Seminars in Cancer Biology 66 (2020): 89‐100. 30880243 10.1016/j.semcancer.2019.03.002

[74] J. D. Mancias , X. Wang , S. P. Gygi , J. W. Harper , and A. C. Kimmelman , “Quantitative proteomics identifies NCOA4 as the cargo receptor mediating ferritinophagy,” Nature 509, no. 7498 (2014): 105‐109. 24695223 10.1038/nature13148 PMC4180099

[75] M. Gao , J. Yi , J. Zhu , et al., “Role of Mitochondria in Ferroptosis,” Molecular Cell 73, no. 2 (2019): 354‐363. e3. 30581146 10.1016/j.molcel.2018.10.042 PMC6338496

[76] S. M. Parikh , Y. Yang , L. He , C. Tang , M. Zhan , and Z. Dong , “Mitochondrial Function and Disturbances in the Septic Kidney,” Seminars in Nephrology 35, no. 1 (2015): 108‐119. 25795504 10.1016/j.semnephrol.2015.01.011 PMC4465453

[77] P. J. Urrutia , N. P. Mena , and M. T. Núñez , “The interplay Between iron accumulation, mitochondrial dysfunction, and inflammation During the execution step of neurodegenerative disorders,” Frontiers in pharmacology 5 (2014): 38. 24653700 10.3389/fphar.2014.00038 PMC3948003

[78] A. Campanella , E. Rovelli , P. Santambrogio , A. Cozzi , F. Taroni , and S. Levi , “Mitochondrial ferritin limits oxidative damage regulating mitochondrial iron availability: Hypothesis for a protective role in Friedreich ataxia,” Human Molecular Genetics 18, no. 1 (2009): 1‐11. 18815198 10.1093/hmg/ddn308 PMC3298861

[79] F. Missirlis , S. Holmberg , T. Georgieva , B. C. Dunkov , T. A. Rouault , and J. H. Law , “Characterization of mitochondrial ferritin in Drosophila,” PNAS 103, no. 15 (2006): 5893‐5898. 16571656 10.1073/pnas.0601471103 PMC1458669

[80] G. Gao , N. Zhang , Y. Q. Wang , et al., “Mitochondrial Ferritin Protects Hydrogen Peroxide‐Induced Neuronal Cell Damage,” Aging Dis 8, no. 4 (2017): 458‐470. 28840060 10.14336/AD.2016.1108 PMC5524808

[81] A. A. Starkov , “The role of mitochondria in reactive oxygen species metabolism and signaling,” Ann N Y Acad Sci 1147 (2008): 37‐52. 19076429 10.1196/annals.1427.015 PMC2869479

[82] Y. Q. Wang , S. Y. Chang , Q. Wu , et al., “The Protective Role of Mitochondrial Ferritin on Erastin‐Induced Ferroptosis,” Frontiers in aging neuroscience 8 (2016): 308. 28066232 10.3389/fnagi.2016.00308 PMC5167726

[83] S. Toyokuni , F. Ito , K. Yamashita , Y. Okazaki , and S. Akatsuka , “Iron and thiol redox signaling in cancer: An exquisite balance to escape ferroptosis,” Free Radic Biol Med 108 (2017): 610‐626. 28433662 10.1016/j.freeradbiomed.2017.04.024

[84] S. Agrawal , J. Fox , B. Thyagarajan , and J. H. Fox , “Brain mitochondrial iron accumulates in Huntington's disease, mediates mitochondrial dysfunction, and can be removed pharmacologically,” Free Radic Biol Med 120 (2018): 317‐329. 29625173 10.1016/j.freeradbiomed.2018.04.002 PMC5940499

[85] O. Edenharter , J. Clement , S. Schneuwly , and J. A. Navarro , “Overexpression of Drosophila frataxin triggers cell death in an iron‐dependent manner,” Journal of Neurogenetics 31, no. 4 (2017): 189‐202. 28838288 10.1080/01677063.2017.1363200

[86] K. Chen , G. Lin , N. A. Haelterman , et al., “Loss of Frataxin induces iron toxicity, sphingolipid synthesis, and Pdk1/Mef2 activation, leading to neurodegeneration,” Elife 5 (2016): e16043. 27343351 10.7554/eLife.16043 PMC4956409

[87] S. J. Oh , M. Ikeda , T. Ide , K. Y. Hur , and M. S. Lee , “Mitochondrial event as an ultimate step in ferroptosis,” Cell Death Discov 8 (2022): 414. 36209144 10.1038/s41420-022-01199-8 PMC9547870

[88] K. G. Lyamzaev , A. A. Panteleeva , R. A. Simonyan , A. V. Avetisyan , and B. V. Chernyak , “Mitochondrial Lipid Peroxidation Is Responsible for Ferroptosis,” Cells 12, no. 4 (2023): 611. 36831278 10.3390/cells12040611 PMC9954536

[89] M. Colombini , “VDAC: The channel at the interface Between mitochondria and the cytosol,” Molecular and Cellular Biochemistry 256‐257 (2004): 107‐115. 1‐2. 10.1023/b:mcbi.0000009862.17396.8d 14977174

[90] N. Yagoda , M. von Rechenberg , E. Zaganjor , et al., “RAS–RAF–MEK‐dependent oxidative cell death involving voltage‐dependent anion channels,” Nature 447, no. 7146 (2007): 864‐868. 17568748 10.1038/nature05859 PMC3047570

[91] R. Guo , J. Duan , S. Pan , et al., “The Road From AKI to CKD: Molecular Mechanisms and Therapeutic Targets of Ferroptosis,” Cell death & disease 14, no. 7 (2023): 426. 37443140 10.1038/s41419-023-05969-9 PMC10344918

[92] Y. Chen , Y. Liu , T. Lan , et al., “Quantitative Profiling of Protein Carbonylations in Ferroptosis by an Aniline‐Derived Probe,” Journal of the American Chemical Society 140, no. 13 (2018): 4712‐4720. 29569437 10.1021/jacs.8b01462

[93] Y. Yang , M. Luo , K. Zhang , et al., “Nedd4 ubiquitylates VDAC2/3 to suppress erastin‐induced ferroptosis in melanoma,” Nature Communications 11, no. 1 (2020): 433. 10.1038/s41467-020-14324-x PMC6978386 31974380

[94] M. A. Badgley , D. M. Kremer , H. C. Maurer , et al., “Cysteine depletion induces pancreatic tumor ferroptosis in mice,” Science 368, no. 6486 (2020): 85‐89. 32241947 10.1126/science.aaw9872 PMC7681911

[95] X. Lang , M. D. Green , W. Wang , et al., “Radiotherapy and Immunotherapy Promote Tumoral Lipid Oxidation and Ferroptosis via Synergistic Repression of SLC7A11,” Cancer discovery 9, no. 12 (2019): 1673‐1685. 31554642 10.1158/2159-8290.CD-19-0338 PMC6891128

[96] T. Himi , M. Ikeda , T. Yasuhara , and S. I. Murota , “Oxidative neuronal death caused by glutamate uptake inhibition in cultured hippocampal neurons,” Journal of Neuroscience Research 71, no. 5 (2003): 679‐688. 12584726 10.1002/jnr.10510

[97] L. Wang , Y. Liu , T. Du , et al., “ATF3 promotes erastin‐induced ferroptosis by suppressing system Xc,” Cell Death and Differentiation 27, no. 2 (2020): 662‐675. 31273299 10.1038/s41418-019-0380-z PMC7206049

[98] Y. Xu , Y. Li , J. Li , and W. Chen , “Ethyl carbamate triggers ferroptosis in liver Through inhibiting GSH synthesis and suppressing Nrf2 activation,” Redox Biology 53 (2022): 102349. 35623314 10.1016/j.redox.2022.102349 PMC9142717

[99] J. Wang , Y. Liu , Y. Wang , and L. Sun , “The Cross‐Link Between Ferroptosis and Kidney Diseases,” Oxid Med Cell Longev 2021 (2021): 6654887. 34007403 10.1155/2021/6654887 PMC8110383

[100] F. Ursini , M. Maiorino , M. Valente , L. Ferri , and C. Gregolin , “Purification From pig liver of a protein which protects liposomes and biomembranes From peroxidative degradation and exhibits glutathione peroxidase activity on phosphatidylcholine hydroperoxides,” Biochimica Et Biophysica Acta 710, no. 2 (1982): 197‐211. 7066358 10.1016/0005-2760(82)90150-3

[101] V. E. Kagan , G. Mao , F. Qu , et al., “Oxidized arachidonic and adrenic PEs navigate cells to ferroptosis,” Nature Chemical Biology 13, no. 1 (2017): 81‐90. 27842066 10.1038/nchembio.2238 PMC5506843

[102] F. Ursini and M. Maiorino , “Lipid peroxidation and ferroptosis: The role of GSH and GPx4,” Free Radic Biol Med 152 (2020): 175‐185. 32165281 10.1016/j.freeradbiomed.2020.02.027

[103] K. Hosohata , T. Harnsirikarn , and S. Chokesuwattanaskul , “Ferroptosis: A Potential Therapeutic Target in Acute Kidney Injury,” International Journal of Molecular Sciences 23, no. 12 (2022): 6583. 35743026 10.3390/ijms23126583 PMC9223765

[104] T. M. Seibt , B. Proneth , and M. Conrad , “Role of GPX4 in ferroptosis and its pharmacological implication,” Free Radic Biol Med 133 (2019): 144‐152. 30219704 10.1016/j.freeradbiomed.2018.09.014

[105] C. Chen , D. Wang , Y. Yu , et al., “Legumain promotes tubular ferroptosis by facilitating chaperone‐mediated autophagy of GPX4 in AKI,” Cell death & disease 12, no. 1 (2021): 65. 33431801 10.1038/s41419-020-03362-4 PMC7801434

[106] K. Wu , M. Yan , T. Liu , et al., “Creatine kinase B suppresses ferroptosis by phosphorylating GPX4 Through a moonlighting function,” Nature Cell Biology 25, no. 5 (2023): 714‐725. 37156912 10.1038/s41556-023-01133-9

[107] Q. Xue , D. Yan , X. Chen , et al., “Copper‐dependent autophagic degradation of GPX4 drives ferroptosis,” Autophagy 19, no. 7 (2023): 1982‐1996. 36622894 10.1080/15548627.2023.2165323 PMC10283421

[108] D. M. Cheff , C. Huang , K. C. Scholzen , et al., “The ferroptosis inducing compounds RSL3 and ML162 are not direct inhibitors of GPX4 but of TXNRD1,” Redox Biology 62 (2023): 102703. 37087975 10.1016/j.redox.2023.102703 PMC10149367

[109] P. Sabatier , C. M. Beusch , R. Gencheva , Q. Cheng , R. Zubarev , and E. S. J. Arnér , “Comprehensive chemical proteomics analyses reveal that the new TRi‐1 and TRi‐2 compounds are more specific thioredoxin reductase 1 inhibitors Than auranofin,” Redox Biology 48 (2021): 102184. 34788728 10.1016/j.redox.2021.102184 PMC8591550

[110] W. Li , L. Liang , S. Liu , H. Yi , and Y. Zhou , “FSP1: A key regulator of ferroptosis,” Trends in Molecular Medicine 29, no. 9 (2023): 753‐764. 37357101 10.1016/j.molmed.2023.05.013

[111] F. Zeng , X. Chen , and G. Deng , “The anti‐ferroptotic role of FSP1: Current molecular mechanism and therapeutic approach,” Mol Biomed 3, no. 1 (2022): 37. 36445538 10.1186/s43556-022-00105-z PMC9708954

[112] K. Bersuker , J. M. Hendricks , Z. Li , et al., “The CoQ oxidoreductase FSP1 acts parallel to GPX4 to inhibit ferroptosis,” Nature 575, no. 7784 (2019): 688‐692. 31634900 10.1038/s41586-019-1705-2 PMC6883167

[113] K. Shimada , R. Skouta , A. Kaplan , et al., “Global survey of cell death mechanisms reveals metabolic regulation of ferroptosis,” Nature Chemical Biology 12, no. 7 (2016): 497‐503. 27159577 10.1038/nchembio.2079 PMC4920070

[114] P. Mladěnka , K. Macáková , L. Kujovská Krčmová , et al., “Vitamin K—sources, physiological role, kinetics, deficiency, detection, therapeutic use, and toxicity,” Nutrition Reviews 80, no. 4 (2022): 677‐698. 34472618 10.1093/nutrit/nuab061 PMC8907489

[115] X. Lin , Q. Zhang , Q. Li , et al., “Upregulation of CoQ shifts ferroptosis dependence From GPX4 to FSP1 in acquired radioresistance,” Drug Resist Updat Rev Comment Antimicrob Anticancer Chemother 73 (2023): 101032. 10.1016/j.drup.2023.101032 38198846

[116] Y. Liu , D. Cheng , Y. Wang , et al., “UHRF1‐mediated ferroptosis promotes pulmonary fibrosis via epigenetic repression of GPX4 and FSP1 genes,” Cell death & disease 13, no. 12 (2022): 1070. 36566325 10.1038/s41419-022-05515-z PMC9789966

[117] J. Zheng and M. Conrad , “The Metabolic Underpinnings of Ferroptosis,” Cell metabolism 32, no. 6 (2020): 920‐937. 33217331 10.1016/j.cmet.2020.10.011

[118] H. Yuan , X. Li , X. Zhang , R. Kang , and D. Tang , “Identification of ACSL4 as a biomarker and contributor of ferroptosis,” Biochemical and Biophysical Research Communications 478, no. 3 (2016): 1338‐1343. 27565726 10.1016/j.bbrc.2016.08.124

[119] I. Ingold , C. Berndt , S. Schmitt , et al., “Selenium Utilization by GPX4 Is Required to Prevent Hydroperoxide‐Induced Ferroptosis,” Cell 172, no. 3 (2018): 409‐422. e21. 29290465 10.1016/j.cell.2017.11.048

[120] H. L. Zhang , B. X. Hu , Z. L. Li , et al., “PKCβII phosphorylates ACSL4 to amplify lipid peroxidation to induce ferroptosis,” Nature Cell Biology 24, no. 1 (2022): 88‐98. 35027735 10.1038/s41556-021-00818-3

[121] P. Liao , W. Wang , and W. Wang , “CD8+ T cells and fatty acids orchestrate tumor ferroptosis and immunity via ACSL4,” Cancer Cell 40, no. 4 (2022): 365‐378. e6. 35216678 10.1016/j.ccell.2022.02.003 PMC9007863

[122] Y. Yang , T. Zhu , X. Wang , et al., “ACSL3 and ACSL4, Distinct Roles in Ferroptosis and Cancers,” Cancers 14, no. 23 (2022): 5896. 36497375 10.3390/cancers14235896 PMC9739553

[123] M. Li , Z. Meng , S. Yu , et al., “Baicalein ameliorates cerebral ischemia‐reperfusion injury by inhibiting ferroptosis via regulating GPX4/ACSL4/ACSL3 axis,” Chemico‐Biological Interactions 366 (2022): 110137. 36055377 10.1016/j.cbi.2022.110137

[124] C. Louandre , I. Marcq , H. Bouhlal , et al., “The retinoblastoma (Rb) protein regulates ferroptosis induced by sorafenib in human hepatocellular carcinoma cells,” Cancer Letters 356, no. 2 Pt B (2015): 971‐977. 25444922 10.1016/j.canlet.2014.11.014

[125] S. M. Wilhelm , L. Adnane , P. Newell , A. Villanueva , J. M. Llovet , and M. Lynch , “Preclinical overview of sorafenib, a multikinase inhibitor that targets both Raf and VEGF and PDGF receptor tyrosine kinase signaling,” Molecular Cancer Therapeutics 7, no. 10 (2008): 3129‐3140. 18852116 10.1158/1535-7163.MCT-08-0013 PMC12261297

[126] G. M. Keating and A. Santoro , “Sorafenib: A review of its use in advanced hepatocellular carcinoma,” Drugs 69, no. 2 (2009): 223‐240. 19228077 10.2165/00003495-200969020-00006

[127] Y. Zhang , D. Xue , X. Wang , M. Lu , B. Gao , and X. Qiao , “Screening of kinase inhibitors targeting BRAF for regulating autophagy based on kinase pathways,” Mol Med Rep 9, no. 1 (2014): 83‐90. 24213221 10.3892/mmr.2013.1781

[128] S. Dolma , S. L. Lessnick , W. C. Hahn , and B. R. Stockwell , “Identification of genotype‐selective antitumor agents using synthetic lethal chemical screening in engineered human tumor cells,” Cancer Cell 3, no. 3 (2003): 285‐296. 12676586 10.1016/s1535-6108(03)00050-3

[129] W. S. Yang , R. SriRamaratnam , M. E. Welsch , et al., “Regulation of ferroptotic cancer cell death by GPX4,” Cell 156, no. 1‐2 (2014): 317‐331. 24439385 10.1016/j.cell.2013.12.010 PMC4076414

[130] D. Moosmayer , A. Hilpmann , J. Hoffmann , et al., “Crystal structures of the selenoprotein glutathione peroxidase 4 in its apo form and in complex With the covalently bound inhibitor ML162,” Acta Crystallogr Sect Struct Biol 77, no. Pt 2 (2021): 237‐248. 10.1107/S2059798320016125 PMC7869902 33559612

[131] Y. Wu , S. Zhang , X. Gong , et al., “The epigenetic regulators and metabolic changes in ferroptosis‐associated cancer progression,” Molecular cancer 19 (2020): 39. 32103754 10.1186/s12943-020-01157-x PMC7045519

[132] H. Yamaguchi , J. L. Hsu , C. T. Chen , et al., “Caspase‐independent cell death is involved in the negative effect of EGF receptor inhibitors on cisplatin in non‐small cell lung cancer cells,” Clin Cancer Res Off J Am Assoc Cancer Res 19, no. 4 (2013): 845‐854. 10.1158/1078-0432.CCR-12-2621 PMC3703145 23344263

[133] M. Y. Kwon , E. Park , S. J. Lee , and S. W. Chung , “Heme oxygenase‐1 accelerates erastin‐induced ferroptotic cell death,” Oncotarget 6, no. 27 (2015): 24393‐24403. 26405158 10.18632/oncotarget.5162 PMC4695193

[134] P. W. Gout , A. R. Buckley , C. R. Simms , and N. Bruchovsky , “Sulfasalazine, a potent suppressor of lymphoma growth by inhibition of the x(c)‐ cystine transporter: A new action for an old drug,” Leukemia 15, no. 10 (2001): 1633‐1640. 11587223 10.1038/sj.leu.2402238

[135] R. Skouta , S. J. Dixon , J. Wang , et al., “Ferrostatins inhibit oxidative lipid damage and cell death in diverse disease models,” Journal of the American Chemical Society 136, no. 12 (2014): 4551‐4556. 24592866 10.1021/ja411006a PMC3985476

[136] A. Hinman , C. R. Holst , J. C. Latham , et al., “Vitamin E hydroquinone is an endogenous regulator of ferroptosis via redox control of 15‐lipoxygenase,” PLoS ONE 13, no. 8 (2018): e0201369. 30110365 10.1371/journal.pone.0201369 PMC6093661

[137] R. Kang , L. Zeng , S. Zhu , et al., “Lipid Peroxidation Drives Gasdermin D‐Mediated Pyroptosis in Lethal Polymicrobial Sepsis,” Cell Host & Microbe 24, no. 1 (2018): 97‐108. e4. 29937272 10.1016/j.chom.2018.05.009 PMC6043361

[138] D. Wallach , T. B. Kang , and A. Kovalenko , “Concepts of tissue injury and cell death in inflammation: A historical perspective,” Nature Reviews Immunology 14, no. 1 (2014): 51‐59. 10.1038/nri3561 24336099

[139] B. Proneth and M. Conrad , “Ferroptosis and necroinflammation, a yet poorly explored link,” Cell Death and Differentiation 26, no. 1 (2019): 14‐24. 30082768 10.1038/s41418-018-0173-9 PMC6294786

[140] Y. Qiu , Y. Cao , W. Cao , Y. Jia , and N. Lu , “The Application of Ferroptosis in Diseases,” Pharmacological Research 159 (2020): 104919. 32464324 10.1016/j.phrs.2020.104919

[141] Y. Sun , P. Chen , B. Zhai , et al., “The emerging role of ferroptosis in inflammation,” Biomedicine & Pharmacotherapy 127 (2020): 110108. 32234642 10.1016/j.biopha.2020.110108

[142] H. Mao , Y. Zhao , H. Li , and L. Lei , “Ferroptosis as an emerging target in inflammatory diseases,” Progress in Biophysics and Molecular Biology 155 (2020): 20‐28. 32311424 10.1016/j.pbiomolbio.2020.04.001

[143] W. S. Yang , K. J. Kim , M. M. Gaschler , M. Patel , M. S. Shchepinov , and B. R. Stockwell , “Peroxidation of polyunsaturated fatty acids by lipoxygenases drives ferroptosis,” PNAS 113, no. 34 (2016): E4966‐E4975. 27506793 10.1073/pnas.1603244113 PMC5003261

[144] J. Y. Lee , W. K. Kim , K. H. Bae , S. C. Lee , and E. W. Lee , “Lipid Metabolism and Ferroptosis,” Biology 10, no. 3 (2021): 184. 33801564 10.3390/biology10030184 PMC8000263

[145] H. Mao , Y. Zhao , H. Li , and L. Lei , “Ferroptosis as an emerging target in inflammatory diseases,” Progress in Biophysics and Molecular Biology 155 (2020): 20‐28. 32311424 10.1016/j.pbiomolbio.2020.04.001

[146] Y. Sun , P. Chen , B. Zhai , et al., “The emerging role of ferroptosis in inflammation,” Biomedicine & Pharmacotherapy 127 (2020): 110108. 32234642 10.1016/j.biopha.2020.110108

[147] J. Loscalzo , “Membrane Redox State and Apoptosis: Death by Peroxide,” Cell metabolism 8, no. 3 (2008): 182‐183. 18762018 10.1016/j.cmet.2008.08.004

[148] G. Folco and R. C. Murphy , “Eicosanoid transcellular biosynthesis: From cell‐cell interactions to in vivo tissue responses,” Pharmacological Reviews 58, no. 3 (2006): 375‐388. 16968946 10.1124/pr.58.3.8

[149] B. Proneth and M. Conrad , “Ferroptosis and necroinflammation, a yet poorly explored link,” Cell Death and Differentiation 26, no. 1 (2019): 14‐24. 30082768 10.1038/s41418-018-0173-9 PMC6294786

[150] H. H. Dar , Y. Y. Tyurina , K. Mikulska‐Ruminska , et al., “ *Pseudomonas aeruginosa* utilizes host polyunsaturated phosphatidylethanolamines to trigger theft‐ferroptosis in bronchial epithelium,” Journal of Clinical Investigation 128, no. 10 (2019): 4639‐4653. 10.1172/JCI99490 PMC6159971 30198910

[151] I. Splichal , S. M. Donovan , V. Jenistova , et al., “High Mobility Group Box 1 and TLR4 Signaling Pathway in Gnotobiotic Piglets Colonized/Infected With L. amylovorus, L. mucosae, E. coli Nissle 1917 and S. Typhimurium,” International Journal of Molecular Sciences 20, no. 24 (2019): 6294. 31847111 10.3390/ijms20246294 PMC6940798

[152] Q. Wen , J. Liu , R. Kang , B. Zhou , and D. Tang , “The release and activity of HMGB1 in ferroptosis,” Biochemical and Biophysical Research Communications 510, no. 2 (2019): 278‐283. 30686534 10.1016/j.bbrc.2019.01.090

[153] Y. Sun , P. Chen , B. Zhai , et al., “The emerging role of ferroptosis in inflammation,” Biomedicine & Pharmacotherapy 127 (2020): 110108. 32234642 10.1016/j.biopha.2020.110108

[154] S. J. Dixon , K. M. Lemberg , M. R. Lamprecht , et al., “Ferroptosis: An iron‐dependent form of nonapoptotic cell death,” Cell 149, no. 5 (2012): 1060‐1072. 22632970 10.1016/j.cell.2012.03.042 PMC3367386

[155] E. M. Kenny , E. Fidan , Q. Yang , et al., “Ferroptosis Contributes to Neuronal Death and Functional Outcome After Traumatic Brain Injury∗,” Critical Care Medicine 47, no. 3 (2019): 410‐418. 30531185 10.1097/CCM.0000000000003555 PMC6449247

[156] Z. Zhang , Y. Wu , S. Yuan , et al., “Glutathione peroxidase 4 participates in secondary brain injury Through mediating ferroptosis in a rat model of intracerebral hemorrhage,” Brain Research 1701 (2018): 112‐125. 30205109 10.1016/j.brainres.2018.09.012

[157] D. Martin‐Sanchez , O. Ruiz‐Andres , J. Poveda , et al., “Ferroptosis, but not necroptosis, is important in nephrotoxic folic acid‐induced AKI,” Journal of the American Society of Nephrology 28, no. 1 (2017): 218‐229. 27352622 10.1681/ASN.2015121376 PMC5198282

[158] S. Masaldan , A. A. Belaidi , S. Ayton , and A. I. Bush , “Cellular senescence and iron dyshomeostasis in alzheimer's disease,” Pharmaceuticals 12, no. 2 (2019): 93. 31248150 10.3390/ph12020093 PMC6630536

[159] M. Sarhan , W. G. Land , W. Tonnus , C. P. Hugo , and A. Linkermann , “Origin and Consequences of Necroinflammation,” Physiological Reviews 98, no. 2 (2018): 727‐780. 29465288 10.1152/physrev.00041.2016

[160] A. Linkermann , “Death and fire‐the concept of necroinflammation,” Cell Death and Differentiation 26, no. 1 (2019): 1‐3. 30470796 10.1038/s41418-018-0218-0 PMC6294805

[161] T. Gong , L. Liu , W. Jiang , and R. Zhou , “DAMP‐sensing receptors in sterile inflammation and inflammatory diseases,” Nature Reviews Immunology 20, no. 2 (2020): 95‐112. 10.1038/s41577-019-0215-7 31558839

[162] F. Pandolfi , S. Altamura , S. Frosali , and P. Conti , “Key Role of DAMP in Inflammation, Cancer, and Tissue Repair,” Clinical Therapeutics 38, no. 5 (2016): 1017‐1028. 27021609 10.1016/j.clinthera.2016.02.028

[163] S. Reuter , S. C. Gupta , M. M. Chaturvedi , and B. B. Aggarwal , “Oxidative stress, inflammation, and cancer: How are they linked?,” Free Radic Biol Med 49, no. 11 (2010): 1603‐1616. 20840865 10.1016/j.freeradbiomed.2010.09.006 PMC2990475

[164] T. W. Mak , M. Grusdat , G. S. Duncan , et al., “Glutathione Primes T Cell Metabolism for Inflammation,” Immunity 46, no. 4 (2017): 675‐689. 28423341 10.1016/j.immuni.2017.03.019

[165] C. J. Chen , H. S. Huang , and W. C. Chang , “Depletion of phospholipid hydroperoxide glutathione peroxidase up‐regulates arachidonate metabolism by 12S‐lipoxygenase and cyclooxygenase 1 in human epidermoid carcinoma A431 cells,” FASEB J Off Publ Fed Am Soc Exp Biol 17, no. 12 (2003): 1694‐1696. 10.1096/fj.02-0847fje 12958179

[166] H. Sakamoto , H. Imai , and Y. Nakagawa , “Involvement of phospholipid hydroperoxide glutathione peroxidase in the modulation of prostaglandin D2 synthesis,” Journal of Biological Chemistry 275, no. 51 (2000): 40028‐40035. 11010961 10.1074/jbc.M003191200

[167] T. W. Mak , M. Grusdat , G. S. Duncan , et al., “Glutathione Primes T Cell Metabolism for Inflammation,” Immunity 46, no. 4 (2017): 675‐689. 28423341 10.1016/j.immuni.2017.03.019

[168] C. Li , X. Deng , X. Xie , Y. Liu , J. P. Friedmann Angeli , and L. Lai , “Activation of Glutathione Peroxidase 4 as a Novel Anti‐inflammatory Strategy,” Frontiers in pharmacology 9 (2018): 1120. 30337875 10.3389/fphar.2018.01120 PMC6178849

[169] H. Suzuki , Y. Kayama , M. Sakamoto , et al., “Arachidonate 12/15‐lipoxygenase‐induced inflammation and oxidative stress are involved in the development of diabetic cardiomyopathy,” Diabetes 64, no. 2 (2015): 618‐630. 25187369 10.2337/db13-1896

[170] Y. Zhou , K. T. Que , Z. Zhang , et al., “Iron overloaded polarizes macrophage to proinflammation phenotype Through ROS/acetyl‐p53 pathway,” Cancer medicine 7, no. 8 (2018): 4012‐4022. 29989329 10.1002/cam4.1670 PMC6089144

[171] P. Handa , S. Thomas , V. Morgan‐Stevenson , et al., “Iron alters macrophage polarization status and leads to steatohepatitis and fibrogenesis,” J Leukoc Biol 105, no. 5 (2019): 1015‐1026. 30835899 10.1002/JLB.3A0318-108R

[172] S. Islam , S. Jarosch , J. Zhou , et al., “Anti‐inflammatory and anti‐bacterial effects of iron chelation in experimental sepsis,” Journal of Surgical Research 200, no. 1 (2016): 266‐273. 26235905 10.1016/j.jss.2015.07.001

[173] T. Thorburn , M. Aali , L. Kostek , et al., “Anti‐inflammatory effects of a novel iron chelator, DIBI, in experimental sepsis,” Clinical Hemorheology and Microcirculation 67, no. 3‐4 (2017): 241‐250. 28869457 10.3233/CH-179205

[174] X. Hu , J. Li , M. Fu , X. Zhao , and W. Wang , “The JAK/STAT signaling pathway: From bench to clinic,” Signal Transduct Target Ther 6, no. 1 (2021): 402. 34824210 10.1038/s41392-021-00791-1 PMC8617206

[175] Y. Luo , M. Alexander , M. Gadina , J. J. O'Shea , F. Meylan , and D. M. Schwartz , “JAK‐STAT signaling in human disease: From genetic syndromes to clinical inhibition,” Journal of Allergy and Clinical Immunology 148, no. 4 (2021): 911‐925. 34625141 10.1016/j.jaci.2021.08.004 PMC8514054

[176] N. Awasthi , C. Liongue , and A. C. Ward , “STAT proteins: A kaleidoscope of canonical and non‐canonical functions in immunity and cancer,” J Hematol OncolJ Hematol Oncol 14, no. 1 (2021): 198. 34809691 10.1186/s13045-021-01214-y PMC8607625

[177] P. Xin , X. Xu , C. Deng , et al., “The role of JAK/STAT signaling pathway and its inhibitors in diseases,” International Immunopharmacology 80 (2020): 106210. 31972425 10.1016/j.intimp.2020.106210

[178] R. Kong , N. Wang , W. Han , W. Bao , and J. Lu , “IFNγ‐mediated repression of system xc‐ drives vulnerability to induced ferroptosis in hepatocellular carcinoma cells,” J Leukoc Biol 110, no. 2 (2021): 301‐314. 34318944 10.1002/JLB.3MA1220-815RRR

[179] W. Wang , M. Green , J. E. Choi , et al., “CD8+ T cells regulate tumour ferroptosis During cancer immunotherapy,” Nature 569, no. 7755 (2019): 270‐274. 31043744 10.1038/s41586-019-1170-y PMC6533917

[180] Y. Zhang , Y. Zuo , B. Li , et al., “Propofol prevents oxidative stress and apoptosis by regulating iron homeostasis and targeting JAK/STAT3 signaling in SH‐SY5Y cells,” Brain Research Bulletin 153 (2019): 191‐201. 31472185 10.1016/j.brainresbull.2019.08.018

[181] Y. P. Liu , Z. Z. Qiu , X. H. Li , and E. Y. Li , “Propofol induces ferroptosis and inhibits malignant phenotypes of gastric cancer cells by regulating miR‐125b‐5p/STAT3 axis,” World journal of gastrointestinal oncology 13, no. 12 (2021): 2114‐2128. 35070046 10.4251/wjgo.v13.i12.2114 PMC8713308

[182] M. Y. Hsu , E. Mina , A. Roetto , and P. E. Porporato , “Iron: An Essential Element of Cancer Metabolism,” Cells 9, no. 12 (2020): 2591. 33287315 10.3390/cells9122591 PMC7761773

[183] Y. Chen , F. Wang , P. Wu , et al., “Artesunate induces apoptosis, autophagy and ferroptosis in diffuse large B cell lymphoma cells by impairing STAT3 signaling,” Cell. Signalling 88 (2021): 110167. 34628002 10.1016/j.cellsig.2021.110167

[184] K. V. Kowdley , E. M. Gochanour , V. Sundaram , R. A. Shah , and P. Handa , “Hepcidin Signaling in Health and Disease: Ironing Out the Details,” Hepatol Commun 5, no. 5 (2021): 723‐735. 34027264 10.1002/hep4.1717 PMC8122377

[185] L. Yang , H. Wang , X. Yang , et al., “Auranofin mitigates systemic iron overload and induces ferroptosis via distinct mechanisms,” Signal Transduct Target Ther 5, no. 1 (2020): 138. 32732975 10.1038/s41392-020-00253-0 PMC7393508

[186] E. Saint‐Germain , L. Mignacca , M. Vernier , D. Bobbala , S. Ilangumaran , and G. Ferbeyre , “SOCS1 regulates senescence and ferroptosis by modulating the expression of p53 target genes,” Aging 9, no. 10 (2017): 2137‐2162. 29081404 10.18632/aging.101306 PMC5680560

[187] L. Opazo‐Ríos , Y. Sanchez Matus , R. R. Rodrigues‐Díez , et al., “Anti‐inflammatory, antioxidant and renoprotective effects of SOCS1 mimetic peptide in the BTBR ob/ob mouse model of type 2 diabetes,” BMJ Open Diabetes Res Care 8, no. 1 (2020): e001242. 10.1136/bmjdrc-2020-001242 PMC7478022 32900697

[188] A. Tefferi , “Primary myelofibrosis: 2021 update on diagnosis, risk‐stratification and management,” American Journal of Hematology 96, no. 1 (2021): 145‐162. 33197049 10.1002/ajh.26050

[189] Q. Zhang , M. J. Lenardo , and D. Baltimore , “30 Years of NF‐κB: A Blossoming of Relevance to Human Pathobiology,” Cell 168, no. 1‐2 (2017): 37‐57. 28086098 10.1016/j.cell.2016.12.012 PMC5268070

[190] J. P. Mitchell and R. J. Carmody , “NF‐κB and the Transcriptional Control of Inflammation,” Int Rev Cell Mol Biol 335 (2018): 41‐84. 29305014 10.1016/bs.ircmb.2017.07.007

[191] H. Yu , L. Lin , Z. Zhang , H. Zhang , and H. Hu , “Targeting NF‐κB pathway for the therapy of diseases: Mechanism and clinical study,” Signal Transduct Target Ther 5, no. 1 (2020): 209. 32958760 10.1038/s41392-020-00312-6 PMC7506548

[192] X. Zhong , Z. Zhang , H. Shen , et al., “Hepatic NF‐κB‐Inducing Kinase and Inhibitor of NF‐κB Kinase Subunit α Promote Liver Oxidative Stress, Ferroptosis, and Liver Injury,” Hepatol Commun 5, no. 10 (2021): 1704‐1720. 34558831 10.1002/hep4.1757 PMC8485893

[193] F. Yao , Y. Deng , Y. Zhao , et al., “A targetable LIFR‐NF‐κB‐LCN2 axis controls liver tumorigenesis and vulnerability to ferroptosis,” Nature Communications 12, no. 1 (2021): 7333. 10.1038/s41467-021-27452-9 PMC8683481 34921145

[194] N. Yan , Z. Xu , C. Qu , and J. Zhang , “Dimethyl fumarate improves cognitive deficits in chronic cerebral hypoperfusion rats by alleviating inflammation, oxidative stress, and ferroptosis via NRF2/ARE/NF‐κB signal pathway,” International Immunopharmacology 98 (2021): 107844. 34153667 10.1016/j.intimp.2021.107844

[195] S. Li , Y. He , K. Chen , et al., “RSL3 Drives Ferroptosis Through NF‐κB Pathway Activation and GPX4 Depletion in Glioblastoma,” Oxid Med Cell Longev 2021 (2021): 2915019. 34987700 10.1155/2021/2915019 PMC8720588

[196] B. M. Oh , S. J. Lee , G. L. Park , et al., “Erastin Inhibits Septic Shock and Inflammatory Gene Expression via Suppression of the NF‐κB Pathway,” Journal of Clinical Medicine 8, no. 12 (2019): 2210. 31847346 10.3390/jcm8122210 PMC6947339

[197] K. H. Pulkkinen , S. Ylä‐Herttuala , and A. L. Levonen , “Heme oxygenase 1 is induced by miR‐155 via reduced BACH1 translation in endothelial cells,” Free Radic Biol Med 51, no. 11 (2011): 2124‐2131. 21982894 10.1016/j.freeradbiomed.2011.09.014

[198] M. P. Seldon , G. Silva , N. Pejanovic , et al., “Heme oxygenase‐1 inhibits the expression of adhesion molecules associated With endothelial cell activation via inhibition of NF‐kappaB RelA phosphorylation at serine 276,” J Immunol Baltim Md 1950 179, no. 11 (2007): 7840‐7851. 10.4049/jimmunol.179.11.7840 18025230

[199] Y. Guan , X. Zhao , W. Liu , and Y. Wang , “Galuteolin suppresses proliferation and inflammation in TNF‐α‐induced RA‐FLS cells by activating HMOX1 to regulate IKKβ/NF‐κB pathway,” J Orthop Surg 15, no. 1 (2020): 484. 10.1186/s13018-020-02004-x PMC7579913 33087158

[200] X. Kou , Y. Jing , W. Deng , et al., “Tumor necrosis factor‐α attenuates starvation‐induced apoptosis Through upregulation of ferritin heavy chain in hepatocellular carcinoma cells,” BMC cancer 13 (2013): 438. 24066693 10.1186/1471-2407-13-438 PMC3849379

[201] A. Zarjou , L. M. Black , K. R. McCullough , et al., “Ferritin Light Chain Confers Protection Against Sepsis‐Induced Inflammation and Organ Injury,” Frontiers in immunology 10 (2019): 131. 30804939 10.3389/fimmu.2019.00131 PMC6371952

[202] V. A. K. Rathinam and K. A. Fitzgerald , “Inflammasome Complexes: Emerging Mechanisms and Effector Functions,” Cell 165, no. 4 (2016): 792‐800. 27153493 10.1016/j.cell.2016.03.046 PMC5503689

[203] O. Schnappauf , J. J. Chae , D. L. Kastner , and I. Aksentijevich , “The Pyrin Inflammasome in Health and Disease,” Frontiers in immunology 10 (2019): 1745. 31456795 10.3389/fimmu.2019.01745 PMC6698799

[204] D. Chauhan , L. Vande Walle , and M. Lamkanfi , “Therapeutic modulation of inflammasome pathways,” Immunological Reviews 297, no. 1 (2020): 123‐138. 32770571 10.1111/imr.12908 PMC7497261

[205] U. Gupta , S. Ghosh , C. T. Wallace , et al., “Increased LCN2 (lipocalin 2) in the RPE decreases autophagy and activates inflammasome‐ferroptosis processes in a mouse model of dry AMD,” Autophagy 19, no. 1 (2023): 92‐111. 35473441 10.1080/15548627.2022.2062887 PMC9809950

[206] S. S. Xie , Y. Deng , S. L. Guo , et al., “Endothelial cell ferroptosis mediates monocrotaline‐induced pulmonary hypertension in rats by modulating NLRP3 inflammasome activation,” Scientific Reports 12, no. 1 (2022): 3056. 35197507 10.1038/s41598-022-06848-7 PMC8866506

[207] L. Meihe , G. Shan , K. Minchao , et al., “The Ferroptosis‐NLRP1 Inflammasome: The Vicious Cycle of an Adverse Pregnancy,” Frontiers in Cell and Developmental Biology 9 (2021): 707959. 34490257 10.3389/fcell.2021.707959 PMC8417576

[208] M. Orecchioni , K. Kobiyama , H. Winkels , et al., “Olfactory receptor 2 in vascular macrophages drives atherosclerosis by NLRP3‐dependent IL‐1 production,” Science 375, no. 6577 (2022): 214‐221. 35025664 10.1126/science.abg3067 PMC9744443

[209] S. Chokchaiwong , Y. T. Kuo , S. H. Lin , et al., “Coenzyme Q10 serves to couple mitochondrial oxidative phosphorylation and fatty acid β‐oxidation, and attenuates NLRP3 inflammasome activation,” Free Radic Res 52, no. 11‐12 (2018): 1445‐1455. 30003820 10.1080/10715762.2018.1500695

[210] C. G. Hsu , C. L. Chávez , C. Zhang , M. Sowden , C. Yan , and B. C. Berk , “The lipid peroxidation product 4‐hydroxynonenal inhibits NLRP3 inflammasome activation and macrophage pyroptosis,” Cell Death and Differentiation 29, no. 9 (2022): 1790‐1803. 35264781 10.1038/s41418-022-00966-5 PMC9433404

[211] Y. Zhou , H. Zhou , L. Hua , et al., “Verification of ferroptosis and pyroptosis and identification of PTGS2 as the hub gene in human coronary artery atherosclerosis,” Free Radic Biol Med 171 (2021): 55‐68. 33974977 10.1016/j.freeradbiomed.2021.05.009

[212] R. Kang , L. Zeng , S. Zhu , et al., “Lipid Peroxidation Drives Gasdermin D‐Mediated Pyroptosis in Lethal Polymicrobial Sepsis,” Cell Host & Microbe 24, no. 1 (2018): 97‐108. 29937272 10.1016/j.chom.2018.05.009 PMC6043361

[213] R. Fan , J. Sui , X. Dong , B. Jing , and Z. Gao , “Wedelolactone alleviates acute pancreatitis and associated lung injury via GPX4 mediated suppression of pyroptosis and ferroptosis,” Free Radic Biol Med 173 (2021): 29‐40. 34246777 10.1016/j.freeradbiomed.2021.07.009

[214] J. S. C. Arthur and S. C. Ley , “Mitogen‐activated protein kinases in innate immunity,” Nature Reviews Immunology 13, no. 9 (2013): 679‐692. 10.1038/nri3495 23954936

[215] M. Gaestel , “MAPK‐Activated Protein Kinases (MKs): Novel Insights and Challenges,” Frontiers in Cell and Developmental Biology 3 (2015): 88. 26779481 10.3389/fcell.2015.00088 PMC4705221

[216] A. Martínez‐Limón , M. Joaquin , M. Caballero , F. Posas , and E. de Nadal , “The p38 Pathway: From Biology to Cancer Therapy,” International Journal of Molecular Sciences 21, no. 6 (2020): 1913. 32168915 10.3390/ijms21061913 PMC7139330

[217] K. Zhu , X. Zhu , S. Sun , et al., “Inhibition of TLR4 prevents hippocampal hypoxic‐ischemic injury by regulating ferroptosis in neonatal rats,” Experimental Neurology 345 (2021): 113828. 34343528 10.1016/j.expneurol.2021.113828

[218] K. Zhu , X. Zhu , S. Sun , et al., “Inhibition of TLR4 prevents hippocampal hypoxic‐ischemic injury by regulating ferroptosis in neonatal rats,” Experimental Neurology 345 (2021): 113828. 34343528 10.1016/j.expneurol.2021.113828

[219] D. U. Kim , D. G. Kim , J. W. Choi , et al., “Loganin Attenuates the Severity of Acute Kidney Injury Induced by Cisplatin Through the Inhibition of ERK Activation in Mice,” International Journal of Molecular Sciences 22, no. 3 (2021): 1421. 33572597 10.3390/ijms22031421 PMC7866969

[220] Y. Ikeda , A. Satoh , Y. Horinouchi , et al., “Iron accumulation causes impaired myogenesis correlated With MAPK signaling pathway inhibition by oxidative stress,” FASEB J Off Publ Fed Am Soc Exp Biol 33, no. 8 (2019): 9551‐9564. 10.1096/fj.201802724RR 31145863

[221] S. A. Salama and A. M. Kabel , “Taxifolin ameliorates iron overload‐induced hepatocellular injury: Modulating PI3K/AKT and p38 MAPK signaling, inflammatory response, and hepatocellular regeneration,” Chemico‐Biological Interactions 330 (2020): 109230. 32828744 10.1016/j.cbi.2020.109230

[222] Z. Cavdar , M. A. Oktan , C. Ural , et al., “Renoprotective Effects of Alpha Lipoic Acid on Iron Overload‐Induced Kidney Injury in Rats by Suppressing NADPH Oxidase 4 and p38 MAPK Signaling,” Biological Trace Element Research 193, no. 2 (2020): 483‐493. 31025242 10.1007/s12011-019-01733-3

[223] S. Fu , R. Lv , L. Wang , H. Hou , H. Liu , and S. Shao , “Resveratrol, an antioxidant, protects spinal cord injury in rats by suppressing MAPK pathway,” Saudi J Biol Sci 25, no. 2 (2018): 259‐266. 29472775 10.1016/j.sjbs.2016.10.019 PMC5815991

[224] S. Doll , F. P. Freitas , R. Shah , et al., “FSP1 is a glutathione‐independent ferroptosis suppressor,” Nature 575, no. 7784 (2019): 693‐698. 31634899 10.1038/s41586-019-1707-0

[225] E. H. Sidhom , C. Kim , M. Kost‐Alimova , et al., “Targeting a Braf/Mapk pathway rescues podocyte lipid peroxidation in CoQ‐deficiency kidney disease,” Journal of Clinical Investigation 131, no. 5 (2021): e141380. 141380. 33444290 10.1172/JCI141380 PMC7919729

[226] F. Huang , S. Zhang , X. Li , Y. Huang , S. He , and L. Luo , “STAT3‐mediated ferroptosis is involved in ulcerative colitis,” Free Radic Biol Med 188 (2022): 375‐385. 35779691 10.1016/j.freeradbiomed.2022.06.242

[227] Y. Chen , P. Zhang , W. Chen , and G. Chen , “Ferroptosis mediated DSS‐induced ulcerative colitis associated With Nrf2/HO‐1 signaling pathway,” Immunology Letters 225 (2020): 9‐15. 32540488 10.1016/j.imlet.2020.06.005

[228] M. Xu , J. Tao , Y. Yang , et al., “Ferroptosis involves in intestinal epithelial cell death in ulcerative colitis,” Cell death & disease 11, no. 2 (2020): 86. 32015337 10.1038/s41419-020-2299-1 PMC6997394

[229] J. V. Patankar and C. Becker , “Cell death in the gut epithelium and implications for chronic inflammation,” Nature reviews Gastroenterology & hepatology 17, no. 9 (2020): 543‐556. 32651553 10.1038/s41575-020-0326-4

[230] J. Huang , J. Zhang , J. Ma , et al., “Inhibiting Ferroptosis: A Novel Approach for Ulcerative Colitis Therapeutics,” Oxid Med Cell Longev 2022 (2022): 9678625. 35378823 10.1155/2022/9678625 PMC8976662

[231] Y. Chen , P. Zhang , W. Chen , and G. Chen , “Ferroptosis mediated DSS‐induced ulcerative colitis associated With Nrf2/HO‐1 signaling pathway,” Immunology Letters 225 (2020): 9‐15. 32540488 10.1016/j.imlet.2020.06.005

[232] L. Luo , S. Zhang , N. Guo , H. Li , and S. He , “ACSF2‐mediated ferroptosis is involved in ulcerative colitis,” Life Sciences 313 (2023): 121272. 36509196 10.1016/j.lfs.2022.121272

[233] D. J. Cui , C. Chen , W. Q. Yuan , Y. H. Yang , and L. Han , “Integrative analysis of ferroptosis‐related genes in ulcerative colitis,” Journal of International Medical Research 49, no. 9 (2021): 3000605211042975. 34510961 10.1177/03000605211042975 PMC8442491

[234] Y. Chen , P. Zhang , W. Chen , and G. Chen , “Ferroptosis mediated DSS‐induced ulcerative colitis associated With Nrf2/HO‐1 signaling pathway,” Immunology Letters 225 (2020): 9‐15. 32540488 10.1016/j.imlet.2020.06.005

[235] H. Ma , Q. Shu , D. Li , et al., “Accumulation of Intracellular Ferrous Iron in Inflammatory‐Activated Macrophages,” Biological Trace Element Research 201, no. 5 (2023): 2303‐2310. 35852674 10.1007/s12011-022-03362-9

[236] M. Gao , P. Monian , Q. Pan , W. Zhang , J. Xiang , and X. Jiang , “Ferroptosis is an autophagic cell death process,” Cell Research 26, no. 9 (2016): 1021‐1032. 27514700 10.1038/cr.2016.95 PMC5034113

[237] M. Xu , J. Tao , Y. Yang , et al., “Ferroptosis involves in intestinal epithelial cell death in ulcerative colitis,” Cell death & disease 11, no. 2 (2020): 86. 32015337 10.1038/s41419-020-2299-1 PMC6997394

[238] S. K. Panda , V. Peng , R. Sudan , et al., “Repression of the aryl‐hydrocarbon receptor prevents oxidative stress and ferroptosis of intestinal intraepithelial lymphocytes,” Immunity 56, no. 4 (2023): 797‐812. 36801011 10.1016/j.immuni.2023.01.023 PMC10101911

[239] M. Xu , J. Tao , Y. Yang , et al., “Ferroptosis involves in intestinal epithelial cell death in ulcerative colitis,” Cell death & disease 11, no. 2 (2020): 86. 32015337 10.1038/s41419-020-2299-1 PMC6997394

[240] J. H. Lin , P. Walter , and T. S. B. Yen , “Endoplasmic reticulum stress in disease pathogenesis,” Annu Rev Pathol 3 (2008): 399‐425. 18039139 10.1146/annurev.pathmechdis.3.121806.151434 PMC3653419

[241] Y. Hu , S. Sun , and H. Li . Identification and functional exploration of Ferroptosis and Immune Related Long Non‐Coding RNA in Inflammatory bowel diseas. Published online 2022. 10.21203/rs.3.rs-1857506/v1

[242] J. M. O'Brien , N. A. Ali , S. K. Aberegg , and E. Abraham , “Sepsis,” American Journal of Medicine 120, no. 12 (2007): 1012‐1022. 18060918 10.1016/j.amjmed.2007.01.035

[243] D. Ma , P. Jiang , Y. Jiang , H. Li , and D. Zhang , “Effects of Lipid Peroxidation‐Mediated Ferroptosis on Severe Acute Pancreatitis‐Induced Intestinal Barrier Injury and Bacterial Translocation,” Oxid Med Cell Longev 2021 (2021): 6644576. 34257815 10.1155/2021/6644576 PMC8245223

[244] R. Ma , L. Fang , L. Chen , X. Wang , J. Jiang , and L. Gao , “Ferroptotic stress promotes macrophages Against intracellular bacteria,” Theranostics 12, no. 5 (2022): 2266‐2289. 35265210 10.7150/thno.66663 PMC8899587

[245] M. Matsushita , S. Freigang , C. Schneider , M. Conrad , G. W. Bornkamm , and M. Kopf , “T cell lipid peroxidation induces ferroptosis and prevents immunity to infection,” Journal of Experimental Medicine 212, no. 4 (2015): 555‐568. 25824823 10.1084/jem.20140857 PMC4387287

[246] N. Li , W. Wang , H. Zhou , et al., “Ferritinophagy‐mediated ferroptosis is involved in sepsis‐induced cardiac injury,” Free Radic Biol Med 160 (2020): 303‐318. 32846217 10.1016/j.freeradbiomed.2020.08.009

[247] C. Wang , W. Yuan , A. Hu , et al., “Dexmedetomidine alleviated sepsis‑induced myocardial ferroptosis and septic heart injury,” Mol Med Rep 22, no. 1 (2020): 175‐184. 32377745 10.3892/mmr.2020.11114 PMC7248514

[248] J. Fang , B. Kong , W. Shuai , et al., “Ferroportin‐mediated ferroptosis involved in new‐onset atrial fibrillation With LPS‐induced endotoxemia,” European Journal of Pharmacology 913 (2021): 174622. 34748769 10.1016/j.ejphar.2021.174622

[249] Y. Zhang , L. Zheng , H. Deng , et al., “Electroacupuncture Alleviates LPS‐Induced ARDS Through α7 Nicotinic Acetylcholine Receptor‐Mediated Inhibition of Ferroptosis,” Frontiers in immunology 13 (2022): 832432. 35222419 10.3389/fimmu.2022.832432 PMC8866566

[250] N. N. Liang , Y. Zhao , Y. Y. Guo , et al., “Mitochondria‐derived reactive oxygen species are involved in renal cell ferroptosis During lipopolysaccharide‐induced acute kidney injury,” International Immunopharmacology 107 (2022): 108687. 35279512 10.1016/j.intimp.2022.108687

[251] Z. Xie , M. Xu , J. Xie , et al., “Inhibition of Ferroptosis Attenuates Glutamate Excitotoxicity and Nuclear Autophagy in a CLP Septic Mouse Model,” Shock Augusta Ga 57, no. 5 (2022): 694‐702. 35066511 10.1097/SHK.0000000000001893

[252] J. Xiao , Q. Yang , Y. Zhang , et al., “Maresin conjugates in tissue regeneration‐1 suppresses ferroptosis in septic acute kidney injury,” Cell Biosci 11, no. 1 (2021): 221. 34961563 10.1186/s13578-021-00734-x PMC8711186

[253] Z. Xie , M. Xu , J. Xie , et al., “Inhibition of Ferroptosis Attenuates Glutamate Excitotoxicity and Nuclear Autophagy in a CLP Septic Mouse Model,” Shock Augusta Ga 57, no. 5 (2022): 694‐702. 35066511 10.1097/SHK.0000000000001893

[254] S. Wei , J. Bi , L. Yang , et al., “Serum irisin levels are decreased in patients With sepsis, and exogenous irisin suppresses ferroptosis in the liver of septic mice,” Clinical and translational medicine 10, no. 5 (2020): e173. 32997405 10.1002/ctm2.173 PMC7522760

[255] X. B. Wei , W. Q. Jiang , J. H. Zeng , et al., “Exosome‐Derived lncRNA NEAT1 Exacerbates Sepsis‐Associated Encephalopathy by Promoting Ferroptosis Through Regulating miR‐9‐5p/TFRC and GOT1 Axis,” Molecular Neurobiology 59, no. 3 (2022): 1954‐1969. 35038133 10.1007/s12035-022-02738-1 PMC8882117

[256] J. A. Kellum , P. Romagnani , G. Ashuntantang , C. Ronco , A. Zarbock , and H. J. Anders , “Acute kidney injury,” Nat Rev Dis Primer 7, no. 1 (2021): 1‐17. 10.1038/s41572-021-00284-z 34267223

[257] K. Lee , H. R. Jang , J. Jeon , et al., “Repair phase modeling of ischemic acute kidney injury: Recovery vs. transition to chronic kidney disease,” American journal of translational research 14, no. 1 (2022): 554. 35173874 PMC8829619

[258] Q. Wei , H. Sun , S. Song , et al., “MicroRNA‐668 represses MTP18 to preserve mitochondrial dynamics in ischemic acute kidney injury,” Journal of Clinical Investigation 128, no. 12: 5448‐5464. 10.1172/JCI121859 PMC6264638 30325740

[259] S. R. Mulay , M. M. Honarpisheh , O. Foresto‐Neto , et al., “Mitochondria Permeability Transition versus Necroptosis in Oxalate‐Induced AKI,” J Am Soc Nephrol JASN 30, no. 10 (2019): 1857‐1869. 31296606 10.1681/ASN.2018121218 PMC6779355

[260] G. P. Kaushal , “Autophagy protects proximal tubular cells From injury and apoptosis,” Kidney International 82, no. 12 (2012): 1250‐1253. 23203020 10.1038/ki.2012.337 PMC4068008

[261] A. B. Sanz , M. D. Sanchez‐Niño , A. M. Ramos , and A. Ortiz , “Regulated cell death pathways in kidney disease,” Nature Reviews Nephrology 19, no. 5 (2023): 281‐299. 36959481 10.1038/s41581-023-00694-0 PMC10035496

[262] J. Guerrero‐Mauvecin , N. Villar‐Gómez , S. Rayego‐Mateos , et al., “Regulated necrosis role in inflammation and repair in acute kidney injury,” Frontiers in immunology 14 (2023): 1324996. 38077379 10.3389/fimmu.2023.1324996 PMC10704359

[263] D. Martin‐Sanchez , M. Fontecha‐Barriuso , J. M. Martinez‐Moreno , et al., “Ferroptosis and kidney disease,” Nefrologia: Publicacion Oficial De La Sociedad Espanola Nefrologia 40, no. 4 (2020): 384‐394. 32624210 10.1016/j.nefro.2020.03.005

[264] D. Martin‐Sanchez , O. Ruiz‐Andres , J. Poveda , et al., “Ferroptosis, but Not Necroptosis, Is Important in Nephrotoxic Folic Acid‐Induced AKI,” J Am Soc Nephrol JASN 28, no. 1 (2017): 218‐229. 27352622 10.1681/ASN.2015121376 PMC5198282

[265] Z. Hu , H. Zhang , S. K. Yang , et al., “Emerging Role of Ferroptosis in Acute Kidney Injury,” Oxid Med Cell Longev 2019 (2019): 8010614. 31781351 10.1155/2019/8010614 PMC6875218

[266] J. Li , S. Zheng , Y. Fan , and K. Tan , “Emerging significance and therapeutic targets of ferroptosis: A potential avenue for human kidney diseases,” Cell death & disease 14, no. 9 (2023): 628. 37739961 10.1038/s41419-023-06144-w PMC10516929

[267] Y. Wang , M. Zhang , R. Bi , et al., “ACSL4 deficiency confers protection Against ferroptosis‐mediated acute kidney injury,” Redox Biology 51 (2022): 102262. 35180475 10.1016/j.redox.2022.102262 PMC8857079

[268] W. H. Tao , X. S. Shan , J. X. Zhang , et al., “Dexmedetomidine Attenuates Ferroptosis‐Mediated Renal Ischemia/Reperfusion Injury and Inflammation by Inhibiting ACSL4 via α2‐AR,” Frontiers in pharmacology 13 (2022): 782466. 35873574 10.3389/fphar.2022.782466 PMC9307125

[269] Z. Sun , J. Wu , Q. Bi , and W. Wang , “Exosomal lncRNA TUG1 derived From human urine‐derived stem cells attenuates renal ischemia/reperfusion injury by interacting With SRSF1 to regulate ASCL4‐mediated ferroptosis,” Stem Cell Res Ther 13, no. 1 (2022): 297. 35841017 10.1186/s13287-022-02986-x PMC9284726

[270] L. Huang , L. Zhang , Z. Zhang , et al., “Loss of nephric augmenter of liver regeneration facilitates acute kidney injury via ACSL4‐mediated ferroptosis,” Journal of Cellular and Molecular Medicine (2023), 10.1111/jcmm.18076. Published online December 13. PMC10844764 38088220

[271] L. L. Huang , R. T. Long , G. P. Jiang , et al., “Augmenter of liver regeneration promotes mitochondrial biogenesis in renal ischemia‐reperfusion injury,” Apoptosis Int J Program Cell Death 23, no. 11‐12 (2018): 695‐706. 10.1007/s10495-018-1487-2 30259216

[272] D. J. Zhu , X. H. Liao , W. Q. Huang , H. Sun , L. Zhang , and Q. Liu , “Augmenter of Liver Regeneration Protects Renal Tubular Epithelial Cells From Ischemia‐Reperfusion Injury by Promoting PINK1/Parkin‐Mediated Mitophagy,” Front Physiol 11 (2020): 178. 32231587 10.3389/fphys.2020.00178 PMC7082309

[273] L. L. Huang , X. H. Liao , H. Sun , X. Jiang , Q. Liu , and L. Zhang , “Augmenter of liver regeneration protects the kidney From ischaemia‐reperfusion injury in ferroptosis,” Journal of Cellular and Molecular Medicine 23, no. 6 (2019): 4153‐4164. 30993878 10.1111/jcmm.14302 PMC6533476

[274] X. Sun , N. Huang , P. Li , et al., “TRIM21 ubiquitylates GPX4 and promotes ferroptosis to aggravate ischemia/reperfusion‐induced acute kidney injury,” Life Sciences 321 (2023): 121608. 36958437 10.1016/j.lfs.2023.121608 PMC11483487

[275] R. Feng , Y. Xiong , Y. Lei , et al., “Lysine‐specific demethylase 1 aggravated oxidative stress and ferroptosis induced by renal ischemia and reperfusion injury Through activation of TLR4/NOX4 pathway in mice,” Journal of Cellular and Molecular Medicine 26, no. 15 (2022): 4254‐4267. 35775122 10.1111/jcmm.17444 PMC9344828

[276] A. Ozkok and C. L. Edelstein , “Pathophysiology of cisplatin‐induced acute kidney injury,” BioMed research international 2014 (2014): 967826. 25165721 10.1155/2014/967826 PMC4140112

[277] F. Deng , I. Sharma , Y. Dai , M. Yang , and Y. S. Kanwar , “Myo‐inositol oxygenase expression profile modulates pathogenic ferroptosis in the renal proximal tubule,” Journal of Clinical Investigation 129, no. 11 (2019): 5033‐5049. 31437128 10.1172/JCI129903 PMC6819105

[278] J. H. Baek , A. Yalamanoglu , R. P. Brown , D. M. Saylor , R. A. Malinauskas , and P. W. Buehler , “Renal Toxicodynamic Effects of Extracellular Hemoglobin After Acute Exposure,” Toxicol Sci Off J Soc Toxicol 166, no. 1 (2018): 180‐191. 10.1093/toxsci/kfy193 30085279

[279] L. Zhou , P. Yu , T. T. Wang , et al., “Polydatin Attenuates Cisplatin‐Induced Acute Kidney Injury by Inhibiting Ferroptosis,” Oxid Med Cell Longev 2022 (2022): 9947191. 35075382 10.1155/2022/9947191 PMC8783728

[280] Y. Ma , L. Huang , Z. Zhang , et al., “CD36 promotes tubular ferroptosis by regulating the ubiquitination of FSP1 in acute kidney injury,” Genes Dis 11, no. 1 (2023): 449‐463. 37588197 10.1016/j.gendis.2022.12.003 PMC10425750

[281] L. J. Yan , “Folic acid‐induced animal model of kidney disease,” Anim Models Exp Med 4, no. 4 (2021): 329‐342. 10.1002/ame2.12194 PMC8690981 34977484

[282] R. Kandel and K. P. Singh , “Higher Concentrations of Folic Acid Cause Oxidative Stress, Acute Cytotoxicity, and Long‐Term Fibrogenic Changes in Kidney Epithelial Cells,” Chem. Res. Toxicol. 35, no. 11 (2022): 2168‐2179. 36354958 10.1021/acs.chemrestox.2c00258 PMC10314330

[283] D. Li , B. Liu , Y. Fan , et al., “Nuciferine protects Against folic acid‐induced acute kidney injury by inhibiting ferroptosis,” British Journal of Pharmacology 178, no. 5 (2021): 1182‐1199. 33450067 10.1111/bph.15364

[284] X. Yang , S. Dong , Y. Fan , et al., “Krüppel‐Like Factor 15 Suppresses Ferroptosis by Activating an NRF2/GPX4 Signal to Protect Against Folic Acid‐Induced Acute Kidney Injury,” International Journal of Molecular Sciences 24, no. 19 (2023): 14530. 37833977 10.3390/ijms241914530 PMC10572468

[285] L. Zhang , F. Chen , J. Dong , et al., “HDAC3 aberration‐incurred GPX4 suppression drives renal ferroptosis and AKI‐CKD progression,” Redox Biology 68 (2023): 102939. 37890360 10.1016/j.redox.2023.102939 PMC10638610

[286] R. Vanholder , M. Sükrü Sever , and N. Lameire , “Kidney problems in disaster situations,” Nephrol Ther 17S (2021): S27‐S36. 33910695 10.1016/j.nephro.2020.02.009

[287] G. A. Herrera , “Myoglobin and the kidney: An overview,” Ultrastructural Pathology 18, no. 1‐2 (1994): 113‐117. 8191616 10.3109/01913129409016280

[288] R. A. Zager and K. Burkhart , “Myoglobin toxicity in proximal human kidney cells: Roles of Fe, Ca2+, H2O2, and terminal mitochondrial electron transport,” Kidney International 51, no. 3 (1997): 728‐738. 9067905 10.1038/ki.1997.104

[289] X. Bosch , E. Poch , and J. M. Grau , “Rhabdomyolysis and acute kidney injury,” New England Journal of Medicine 361, no. 1 (2009): 62‐72. 19571284 10.1056/NEJMra0801327

[290] H. Zhu , J. Cen , C. Hong , et al., “Targeting Labile Iron‐Mediated Ferroptosis Provides a Potential Therapeutic Strategy for Rhabdomyolysis‐Induced Acute Kidney Injury,” Acs Chemical Biology 18, no. 6 (2023): 1294‐1304. 37172039 10.1021/acschembio.2c00914

[291] S. Zhao , X. Wang , X. Zheng , et al., “Iron deficiency exacerbates cisplatin‐ or rhabdomyolysis‐induced acute kidney injury Through promoting iron‐catalyzed oxidative damage,” Free Radic Biol Med 173 (2021): 81‐96. 34298093 10.1016/j.freeradbiomed.2021.07.025 PMC9482792

[292] J. M. O'Brien , N. A. Ali , S. K. Aberegg , and E. Abraham , “Sepsis,” American Journal of Medicine 120, no. 12 (2007): 1012‐1022. 18060918 10.1016/j.amjmed.2007.01.035

[293] S. Peerapornratana , C. L. Manrique‐Caballero , H. Gómez , and J. A. Kellum , “Acute kidney injury From sepsis: Current concepts, epidemiology, pathophysiology, prevention and treatment,” Kidney International 96, no. 5 (2019): 1083‐1099. 31443997 10.1016/j.kint.2019.05.026 PMC6920048

[294] J. T. Poston and J. L. Koyner , “Sepsis associated acute kidney injury,” The BMJ 364 (2019): k4891. 30626586 10.1136/bmj.k4891 PMC6890472

[295] S. Buchanan , E. Combet , P. Stenvinkel , and P. G. Shiels , “Klotho, Aging, and the Failing Kidney,” Front Endocrinol 11 (2020): 560. 10.3389/fendo.2020.00560 PMC7481361 32982966

[296] T. Landry , D. Shookster , and H. Huang , “Circulating α‐klotho regulates metabolism via distinct central and peripheral mechanisms,” Metabolism 121 (2021): 154819. 34153302 10.1016/j.metabol.2021.154819 PMC8277751

[297] P. Zhou , C. Zhao , Y. Chen , X. Liu , C. Wu , and Z. Hu , “Klotho activation of Nrf2 inhibits the ferroptosis signaling pathway to ameliorate sepsis‐associated acute kidney injury,” Transl Androl Urol 12, no. 12 (2023): 1871‐1884. 38196698 10.21037/tau-23-573 PMC10772648

[298] W. Q. Zhuo , Y. Wen , H. J. Luo , Z. L. Luo , and L. Wang , “Mechanisms of ferroptosis in chronic kidney disease,” Frontiers in Molecular Biosciences 9 (2022): 975582. 36090053 10.3389/fmolb.2022.975582 PMC9448928

[299] B. J. Nankivell , R. A. Boadle , and D. C. Harris , “Iron accumulation in human chronic renal disease,” Am J Kidney Dis Off J Natl Kidney Found 20, no. 6 (1992): 580‐584. 10.1016/s0272-6386(12)70222-6 1462986

[300] Y. Liu and J. Wang , “Ferroptosis, a Rising Force Against Renal Fibrosis,” Oxid Med Cell Longev 2022 (2022): 7686956. 36275899 10.1155/2022/7686956 PMC9581688

[301] M. Maus , V. López‐Polo , L. Mateo , et al., “Iron accumulation drives fibrosis, senescence and the senescence‐associated secretory phenotype,” Nat Metab 5, no. 12 (2023): 2111‐2130. 38097808 10.1038/s42255-023-00928-2 PMC10730403

[302] Y. Sato and M. Yanagita , “Immune cells and inflammation in AKI to CKD progression,” American Journal of Physiology. Renal Physiology 315, no. 6 (2018): F1501‐F1512. 30156114 10.1152/ajprenal.00195.2018

[303] K. T. K. Giuliani , A. Grivei , P. Nag , et al., “Hypoxic human proximal tubular epithelial cells undergo ferroptosis and elicit an NLRP3 inflammasome response in CD1c+ dendritic cells,” Cell death & disease 13, no. 8 (2022): 739. 36030251 10.1038/s41419-022-05191-z PMC9420140

[304] P. Bhargava and R. G. Schnellmann , “Mitochondrial energetics in the kidney,” Nature Reviews Nephrology 13, no. 10 (2017): 629‐646. 28804120 10.1038/nrneph.2017.107 PMC5965678

[305] J. Wang , Y. Wang , Y. Liu , et al., “Ferroptosis, a new target for treatment of renal injury and fibrosis in a 5/6 nephrectomy‐induced CKD rat model,” Cell Death Discov 8, no. 1 (2022): 127. 35318301 10.1038/s41420-022-00931-8 PMC8941123

[306] J. Zhou , Y. Tan , R. Wang , and X. Li , “Role of Ferroptosis in Fibrotic Diseases,” J Inflamm Res 15 (2022): 3689‐3708. 35783244 10.2147/JIR.S358470 PMC9248952

[307] L. Li , H. Fu , and Y. Liu , “The fibrogenic niche in kidney fibrosis: Components and mechanisms,” Nature Reviews Nephrology 18, no. 9 (2022): 545‐557. 35788561 10.1038/s41581-022-00590-z

[308] Y. Zhang , Y. Mou , J. Zhang , et al., “Therapeutic Implications of Ferroptosis in Renal Fibrosis,” Frontiers in Molecular Biosciences 9 (2022): 890766. 35655759 10.3389/fmolb.2022.890766 PMC9152458

[309] L. Zhou , X. Xue , Q. Hou , and C. Dai , “Targeting Ferroptosis Attenuates Interstitial Inflammation and Kidney Fibrosis,” Kidney Dis Basel Switz 8, no. 1 (2022): 57‐71. 10.1159/000517723 PMC8820137 35224007

[310] B. Zhang , X. Chen , F. Ru , et al., “Liproxstatin‐1 attenuates unilateral ureteral obstruction‐induced renal fibrosis by inhibiting renal tubular epithelial cells ferroptosis,” Cell death & disease 12, no. 9 (2021): 843. 34511597 10.1038/s41419-021-04137-1 PMC8435531

[311] Y. Dai , Y. Chen , D. Mo , et al., “Inhibition of ACSL4 ameliorates tubular ferroptotic cell death and protects Against fibrotic kidney disease,” Communications Biology 6, no. 1 (2023): 907. 37670055 10.1038/s42003-023-05272-5 PMC10480178

[312] W. Lai , R. Huang , B. Wang , et al., “Novel aspect of neprilysin in kidney fibrosis via ACSL4‐mediated ferroptosis of tubular epithelial cells,” MedComm 4, no. 4 (2023): e330. 37457659 10.1002/mco2.330 PMC10349188

[313] B. Wang , L. N. Yang , L. T. Yang , et al., “Fisetin ameliorates fibrotic kidney disease in mice via inhibiting ACSL4‐mediated tubular ferroptosis,” Acta Pharmacologica Sinica (2023), 10.1038/s41401-023-01156-w. Published online September 11. PMC10770410 37696989

[314] M. S. Balzer , T. Doke , Y. W. Yang , et al., “Single‐cell analysis highlights differences in druggable pathways underlying adaptive or fibrotic kidney regeneration,” Nature Communications 13 (2022): 4018. 10.1038/s41467-022-31772-9 PMC9276703 35821371

[315] A. Perrone , A. Giovino , J. Benny , and F. Martinelli , “Advanced Glycation End Products (AGEs): Biochemistry, Signaling, Analytical Methods, and Epigenetic Effects,” Oxid Med Cell Longev 2020 (2020): 3818196. 32256950 10.1155/2020/3818196 PMC7104326

[316] Y. Wang , R. Bi , F. Quan , et al., “Ferroptosis involves in renal tubular cell death in diabetic nephropathy,” European Journal of Pharmacology 888 (2020): 173574. 32976829 10.1016/j.ejphar.2020.173574

[317] S. Kim , S. W. Kang , J. Joo , et al., “Characterization of ferroptosis in kidney tubular cell death Under diabetic conditions,” Cell death & disease 12, no. 2 (2021): 160. 33558472 10.1038/s41419-021-03452-x PMC7870666

[318] S. Li , L. Zheng , J. Zhang , X. Liu , and Z. Wu , “Inhibition of ferroptosis by up‐regulating Nrf2 delayed the progression of diabetic nephropathy,” Free Radic Biol Med 162 (2021): 435‐449. 33152439 10.1016/j.freeradbiomed.2020.10.323

[319] X. Feng , S. Wang , Z. Sun , et al., “Ferroptosis Enhanced Diabetic Renal Tubular Injury via HIF‐1α/HO‐1 Pathway in db/db Mice,” Front Endocrinol 12 (2021): 626390. 10.3389/fendo.2021.626390 PMC7930496 33679620

[320] T. Robert , L. Berthelot , A. Cambier , E. Rondeau , and R. C. Monteiro , “Molecular Insights Into the Pathogenesis of IgA Nephropathy,” Trends in Molecular Medicine 21, no. 12 (2015): 762‐775. 26614735 10.1016/j.molmed.2015.10.003

[321] H. Wang , K. Nishiya , H. Ito , T. Hosokawa , K. Hashimoto , and T. Moriki , “Iron deposition in renal biopsy specimens From patients With kidney diseases,” Am J Kidney Dis Off J Natl Kidney Found 38, no. 5 (2001): 1038‐1044. 10.1053/ajkd.2001.28593 11684557

[322] Z. Y. Tian , Z. Li , L. Chu , et al., “Iron metabolism and chronic inflammation in IgA nephropathy,” Renal Failure 45, no. 1 (2023): 2195012. 37013479 10.1080/0886022X.2023.2195012 PMC10075521

[323] H. Lu , X. Sun , M. Jia , et al., “Rosiglitazone Suppresses Renal Crystal Deposition by Ameliorating Tubular Injury Resulted From Oxidative Stress and Inflammatory Response via Promoting the Nrf2/HO‐1 Pathway and Shifting Macrophage Polarization,” Oxid Med Cell Longev 2021 (2021): 5527137. 34691355 10.1155/2021/5527137 PMC8531781

[324] Z. Ye , Y. Xia , L. Li , et al., “p53 deacetylation alleviates calcium oxalate deposition‐induced renal fibrosis by inhibiting ferroptosis,” Biomed Pharmacother Biomedecine Pharmacother 164 (2023): 114925. 10.1016/j.biopha.2023.114925 37236026

[325] M. Tsujihata , “Mechanism of calcium oxalate renal stone formation and renal tubular cell injury,” Int J Urol Off J Jpn Urol Assoc 15, no. 2 (2008): 115‐120. 10.1111/j.1442-2042.2007.01953.x 18269444

[326] S. R. Khan , “Reactive oxygen species as the molecular modulators of calcium oxalate kidney stone formation: Evidence From clinical and experimental investigations,” Journal of Urology 189, no. 3 (2013): 803‐811. 23022011 10.1016/j.juro.2012.05.078 PMC5683176

[327] J. Zhou , L. Meng , Z. He , et al., “Melatonin exerts a protective effect in ameliorating nephrolithiasis via targeting AMPK/PINK1‐Parkin mediated mitophagy and inhibiting ferroptosis in vivo and in vitro,” International Immunopharmacology 124 (2023): 110801. 37651854 10.1016/j.intimp.2023.110801

[328] Z. He , W. Liao , Q. Song , et al., “Role of ferroptosis induced by a high concentration of calcium oxalate in the formation and development of urolithiasis,” International Journal of Molecular Medicine 47, no. 1 (2021): 289‐301. 33416117 10.3892/ijmm.2020.4770 PMC7723503

[329] L. Li , Z. Ye , Y. Xia , et al., “YAP/ACSL4 Pathway‐Mediated Ferroptosis Promotes Renal Fibrosis in the Presence of Kidney Stones,” Biomedicines 11, no. 10 (2023): 2692. 37893066 10.3390/biomedicines11102692 PMC10603838

[330] Z. Mao , K. Zhong , X. Liu , and X. Zeng , “Ferroptosis contributes to cyclophosphamide‐induced hemorrhagic cystitis,” Chemico‐Biological Interactions 384 (2023): 110701. 37690746 10.1016/j.cbi.2023.110701

[331] X. Wang , T. Ji , Z. Jiang , J. Wang , X. Su , and L. Shan , “Tolterodine ameliorates inflammatory response and ferroptosis Against LPS in human bladder epithelial cells,” Journal of Biochemical and Molecular Toxicology 38, no. 1 (2024): e23517. 37702107 10.1002/jbt.23517

[332] D. Lin , M. Zhang , C. Luo , P. Wei , K. Cui , and Z. Chen , “Targeting Ferroptosis Attenuates Inflammation, Fibrosis, and Mast Cell Activation in Chronic Prostatitis,” Journal of Immunology Research 2022 (2022): 6833867. 35755168 10.1155/2022/6833867 PMC9232311

[333] A. Ghoochani , E. C. Hsu , M. Aslan , et al., “Ferroptosis Inducers Are a Novel Therapeutic Approach for Advanced Prostate Cancer,” Cancer Research 81, no. 6 (2021): 1583‐1594. 33483372 10.1158/0008-5472.CAN-20-3477 PMC7969452

[334] J. Wang , L. Zeng , N. Wu , et al., “Inhibition of phosphoglycerate dehydrogenase induces ferroptosis and overcomes enzalutamide resistance in castration‐resistant prostate cancer cells,” Drug Resist Updat Rev Comment Antimicrob Anticancer Chemother 70 (2023): 100985. 10.1016/j.drup.2023.100985 37423117

[335] H. Wang , X. Yu , D. Liu , et al., “VDR Activation Attenuates Renal Tubular Epithelial Cell Ferroptosis by Regulating Nrf2/HO‐1 Signaling Pathway in Diabetic Nephropathy,” Adv Sci Weinh Baden‐Wurtt Ger 11, no. 10 (2024): e2305563. 10.1002/advs.202305563 PMC10933633 38145959

[336] Z. Liu , P. Nan , Y. Gong , L. Tian , Y. Zheng , and Z. Wu , “Endoplasmic reticulum stress‐triggered ferroptosis via the XBP1‐Hrd1‐Nrf2 pathway induces EMT progression in diabetic nephropathy,” Biomed Pharmacother Biomedecine Pharmacother 164 (2023): 114897. 10.1016/j.biopha.2023.114897 37224754

[337] H. Xiao , X. Du , Z. Tao , et al., “Taurine Inhibits Ferroptosis Mediated by the Crosstalk Between Tumor Cells and Tumor‐Associated Macrophages in Prostate Cancer,” Adv Sci Weinh Baden‐Wurtt Ger 11, no. 3 (2024): e2303894. 10.1002/advs.202303894 PMC10797466 38031260

[338] W. Wang , J. Zhang , Y. Wang , Y. Xu , and S. Zhang , “Identifies microtubule‐binding protein CSPP1 as a novel cancer biomarker associated With ferroptosis and tumor microenvironment,” Computational and Structural Biotechnology Journal 20 (2022): 3322‐3335. 35832625 10.1016/j.csbj.2022.06.046 PMC9253833

[339] D. Liang , Y. Feng , F. Zandkarimi , et al., “Ferroptosis surveillance independent of GPX4 and differentially regulated by sex hormones,” Cell 186, no. 13 (2023): 2748‐2764. e22. 37267948 10.1016/j.cell.2023.05.003 PMC10330611

[340] S. Chang , M. Tang , B. Zhang , D. Xiang , and F. Li , “Ferroptosis in inflammatory arthritis: A promising future,” Frontiers in immunology 13 (2022): 955069. 35958605 10.3389/fimmu.2022.955069 PMC9361863

[341] B. Lai , C. H. Wu , C. Y. Wu , S. F. Luo , and J. H. Lai , “Ferroptosis and Autoimmune Diseases,” Frontiers in immunology 13 (2022): 916664. 35720308 10.3389/fimmu.2022.916664 PMC9203688

[342] J. Hu , R. Zhang , Q. Chang , et al., “p53: A Regulator of Ferroptosis Induced by Galectin‐1 Derived Peptide 3 in MH7A Cells,” Frontiers in Genetics 13 (2022): 920273. 35860469 10.3389/fgene.2022.920273 PMC9289366

[343] G. Nygaard and G. S. Firestein , “Restoring synovial homeostasis in rheumatoid arthritis by targeting fibroblast‐Like synoviocytes,” Nat Rev Rheumatol 16, no. 6 (2020): 316‐333. 32393826 10.1038/s41584-020-0413-5 PMC7987137

[344] M. Taghadosi , M. Adib , A. Jamshidi , M. Mahmoudi , and E. Farhadi , “The p53 status in rheumatoid arthritis With focus on fibroblast‐Like synoviocytes,” Immunologic Research 69, no. 3 (2021): 225‐238. 33983569 10.1007/s12026-021-09202-7

[345] L. Jiang , J. H. Hickman , S. J. Wang , and W. Gu , “Dynamic roles of p53‐mediated metabolic activities in ROS‐induced stress responses,” Cell Cycle Georget Tex 14, no. 18 (2015): 2881‐2885. 10.1080/15384101.2015.1068479 PMC4825584 26218928

[346] Y. Xie , S. Zhu , X. Song , et al., “The Tumor Suppressor p53 Limits Ferroptosis by Blocking DPP4 Activity,” Cell reports 20, no. 7 (2017): 1692‐1704. 28813679 10.1016/j.celrep.2017.07.055

[347] Y. Su , B. Zhao , L. Zhou , et al., “Ferroptosis, a novel pharmacological mechanism of anti‐cancer drugs,” Cancer Letters 483 (2020): 127‐136. 32067993 10.1016/j.canlet.2020.02.015

[348] J. Jhun , J. Moon , J. Ryu , et al., “Liposome/gold hybrid nanoparticle encoded With CoQ10 (LGNP‐CoQ10) suppressed rheumatoid arthritis via STAT3/Th17 targeting,” PLoS ONE 15, no. 11 (2020): e0241080. 33156836 10.1371/journal.pone.0241080 PMC7647073

[349] S. Ravalli , M. A. Szychlinska , R. M. Leonardi , and G. Musumeci , “Recently highlighted nutraceuticals for preventive management of osteoarthritis,” World J Orthop 9, no. 11 (2018): 255‐261. 30479972 10.5312/wjo.v9.i11.255 PMC6242728

[350] D. Jean‐Gilles , L. Li , V. G. Vaidyanathan , et al., “Inhibitory effects of polyphenol punicalagin on type‐II collagen degradation in vitro and inflammation in vivo,” Chemico‐Biological Interactions 205, no. 2 (2013): 90‐99. 23830812 10.1016/j.cbi.2013.06.018

[351] H. Luo and R. Zhang , “Icariin enhances cell survival in lipopolysaccharide‐induced synoviocytes by suppressing ferroptosis via the Xc‐/GPX4 axis,” Exp Ther Med 21, no. 1 (2021): 72. 33365072 10.3892/etm.2020.9504 PMC7716635

[352] R. Zhou , Y. Chen , S. Li , et al., “TRPM7 channel inhibition attenuates rheumatoid arthritis articular chondrocyte ferroptosis by suppression of the PKCα‐NOX4 axis,” Redox Biology 55 (2022): 102411. 35917680 10.1016/j.redox.2022.102411 PMC9344030

[353] G. Ma , Y. Yang , Y. Chen , et al., “Blockade of TRPM7 Alleviates Chondrocyte Apoptosis and Articular Cartilage Damage in the Adjuvant Arthritis Rat Model Through Regulation of the Indian Hedgehog Signaling Pathway,” Frontiers in pharmacology 12 (2021): 655551. 33927631 10.3389/fphar.2021.655551 PMC8076952

[354] K. F. Zhai , H. Duan , C. Y. Cui , et al., “Liquiritin From Glycyrrhiza uralensis Attenuating Rheumatoid Arthritis via Reducing Inflammation, Suppressing Angiogenesis, and Inhibiting MAPK Signaling Pathway,” Journal of Agricultural and Food Chemistry 67, no. 10 (2019): 2856‐2864. 30785275 10.1021/acs.jafc.9b00185

[355] H. Liu , H. Xue , Q. Guo , et al., “Ferroptosis meets inflammation: A new frontier in cancer therapy,” Cancer Letters 620 (2025): 217696. 40189012 10.1016/j.canlet.2025.217696

[356] Y. Dong , M. Zheng , W. Ding , H. Guan , J. Xiao , and F. Li , “Nrf2 activators for the treatment of rare iron overload diseases: From bench to bedside,” Redox Biology 81 (2025): 103551. 39965404 10.1016/j.redox.2025.103551 PMC11876910

[357] M. T. Nuñez , and P. Chana‐Cuevas , “New Perspectives in Iron Chelation Therapy for the Treatment of Neurodegenerative Diseases,” Pharmaceuticals 11, no. 4 (2018): 109. 30347635 10.3390/ph11040109 PMC6316457

[358] Y. Dong , M. Zheng , W. Ding , H. Guan , J. Xiao , and F. Li , “Nrf2 activators for the treatment of rare iron overload diseases: From bench to bedside,” Redox Biology 81 (2025): 103551. 39965404 10.1016/j.redox.2025.103551 PMC11876910

[359] T. Ramadoss , D. S. Weimer , and H. N. Mayrovitz , “Topical Iron Chelator Therapy: Current Status and Future Prospects,” Cureus 15, no. 10: e47720. 38022031 10.7759/cureus.47720 PMC10675985

[360] Y. Wang , Y. Song , L. Xu , et al., “A Membrane‐Targeting Aggregation‐Induced Emission Probe for Monitoring Lipid Droplet Dynamics in Ischemia/Reperfusion‐Induced Cardiomyocyte Ferroptosis,” Adv Sci Weinh Baden‐Wurtt Ger 11, no. 26 (2024): e2309907. 10.1002/advs.202309907 PMC11234465 38696589

[361] M. Tan , W. Li , H. He , et al., “Targeted mitochondrial fluorescence probe With large stokes shift for detecting viscosity changes in vivo and in ferroptosis process,” Spectrochimica Acta. Part A, Molecular and Biomolecular Spectroscopy 315 (2024): 124246. 38593540 10.1016/j.saa.2024.124246

[362] J. Yin , X. Zheng , Y. Zhao , et al., “Investigating the Therapeutic Effects of Ferroptosis on Myocardial Ischemia‐Reperfusion Injury Using a Dual‐Locking Mitochondrial Targeting Strategy,” Angewandte Chemie (International ed in English) 63, no. 21 (2024): e202402537. 38509827 10.1002/anie.202402537

[363] X. Luo , C. Zhang , C. Yue , Y. Jiang , F. Yang , and Y. Xian , “A near‐infrared light‐activated nanoprobe for simultaneous detection of hydrogen polysulfide and sulfur dioxide in myocardial ischemia‐reperfusion injury,” Chemical Science 14, no. 48 (2023): 14290‐14301. 38098706 10.1039/d3sc04937j PMC10718178

[364] W. Yang , Y. Wang , C. Fu , et al., “Quantitative visualization of myocardial ischemia‐reperfusion‐induced cardiac lesions via ferroptosis magnetic particle imaging,” Theranostics 14, no. 3 (2024): 1081‐1097. 38250046 10.7150/thno.89190 PMC10797296

[365] H. L. Zhang , B. X. Hu , Z. P. Ye , et al., “TRPML1 triggers ferroptosis defense and is a potential therapeutic target in AKT‐hyperactivated cancer,” Science Translational Medicine 16, no. 753 (2024): eadk0330. 38924427 10.1126/scitranslmed.adk0330

[366] N. Lorito , A. Subbiani , A. Smiriglia , et al., “FADS1/2 control lipid metabolism and ferroptosis susceptibility in triple‐negative breast cancer,” EMBO Molecular Medicine 16, no. 7 (2024): 1533‐1559. 38926633 10.1038/s44321-024-00090-6 PMC11251055

[367] D. Chen , C. Zhao , J. Zhang , et al., “Small Molecule MIF Modulation Enhances Ferroptosis by Impairing DNA Repair Mechanisms,” Adv Sci Weinh Baden‐Wurtt Ger 11, no. 32 (2024): e2403963. 10.1002/advs.202403963 PMC11348242 38924362

[368] H. Meng , Y. Yu , E. Xie , et al., “Hepatic HDAC3 Regulates Systemic Iron Homeostasis and Ferroptosis via the Hippo Signaling Pathway,” Res Wash DC 6 (2023): 0281. 10.34133/research.0281 PMC10687581 38034086

[369] Z. Jiang , H. Yang , W. Ni , et al., “Attenuation of neuronal ferroptosis in intracerebral hemorrhage by inhibiting HDAC1/2: Microglial heterogenization via the Nrf2/HO1 pathway,” CNS neuroscience & therapeutics 30, no. 3 (2024): e14646. 38523117 10.1111/cns.14646 PMC10961428

[370] C. Zhong , J. Yang , Y. Zhang , et al., “TRPM2 Mediates Hepatic Ischemia‐Reperfusion Injury via Ca2+‐Induced Mitochondrial Lipid Peroxidation Through Increasing ALOX12 Expression,” Res Wash DC 6 (2023): 0159. 10.34133/research.0159 PMC10232356 37275121

[371] T. Li , L. Zhao , Y. Li , et al., “PPM1K mediates metabolic disorder of branched‐chain amino acid and regulates cerebral ischemia‐reperfusion injury by activating ferroptosis in neurons,” Cell death & disease 14, no. 9 (2023): 634. 37752100 10.1038/s41419-023-06135-x PMC10522625

[372] P. Xu , L. Kong , C. Tao , et al., “Elabela‐APJ axis attenuates cerebral ischemia/reperfusion injury by inhibiting neuronal ferroptosis,” Free Radic Biol Med 196 (2023): 171‐186. 36681202 10.1016/j.freeradbiomed.2023.01.008

[373] T. Wu , G. Shi , Z. Ji , S. Wang , L. Geng , and Z. Guo , “Circulating small extracellular vesicle‐encapsulated SEMA5A‐IT1 attenuates myocardial ischemia‐reperfusion injury After cardiac surgery With cardiopulmonary bypass,” Cellular & Molecular Biology Letters 27, no. 1 (2022): 95. 36284269 10.1186/s11658-022-00395-9 PMC9594885

[374] X. Li , X. Peng , X. Zhou , et al., “Small extracellular vesicles delivering lncRNA WAC‐AS1 aggravate renal allograft ischemia‒reperfusion injury by inducing ferroptosis propagation,” Cell Death and Differentiation 30, no. 9 (2023): 2167‐2186. 37532764 10.1038/s41418-023-01198-x PMC10482833

[375] J. Vadolas , G. Z. Ng , K. Kysenius , et al., “SLN124, a GalNac‐siRNA targeting transmembrane serine protease 6, in combination With deferiprone therapy reduces ineffective erythropoiesis and hepatic iron‐overload in a mouse model of β‐thalassaemia,” British Journal of Haematology 194, no. 1 (2021): 200‐210. 33942901 10.1111/bjh.17428 PMC8359948

[376] Y. Liu , D. Zhao , F. Yang , et al., “Situ Self‐Assembled Phytopolyphenol‐Coordinated Intelligent Nanotherapeutics for Multipronged Management of Ferroptosis‐Driven Alzheimer's Disease,” ACS Nano 18, no. 11 (2024): 7890‐7906. 38445977 10.1021/acsnano.3c09286

[377] Y. Ying , Z. Huang , Y. Tu , et al., “A shear‐thinning, ROS‐scavenging hydrogel combined With dental pulp stem cells promotes spinal cord repair by inhibiting ferroptosis,” Bioact Mater 22 (2023): 274‐290. 36263097 10.1016/j.bioactmat.2022.09.019 PMC9556860

[378] C. Li , Y. Wu , Q. Chen , et al., “Pleiotropic Microenvironment Remodeling Micelles for Cerebral Ischemia‐Reperfusion Injury Therapy by Inhibiting Neuronal Ferroptosis and Glial Overactivation,” ACS Nano 17, no. 18 (2023): 18164‐18177. 37703316 10.1021/acsnano.3c05038

[379] Y. Zhang , Z. Zou , S. Liu , et al., “Edaravone‐loaded poly(amino acid) nanogel inhibits ferroptosis for neuroprotection in cerebral ischemia injury,” Asian Journal of Pharmaceutical Sciences 19, no. 2 (2024): 100886. 38590795 10.1016/j.ajps.2024.100886 PMC10999513

[380] Y. Q. Geng , L. N. Qiu , Y. Q. Cheng , et al., “Alleviating Recombinant Tissue Plasminogen Activator‐induced Hemorrhagic Transformation in Ischemic Stroke via Targeted Delivery of a Ferroptosis Inhibitor,” Adv Sci Weinh Baden‐Wurtt Ger 11, no. 24 (2024): e2309517. 10.1002/advs.202309517 PMC11199968 38647405

[381] Y. Wang , Z. Liu , L. Li , et al., “Anti‐ferroptosis exosomes engineered for targeting M2 microglia to improve neurological function in ischemic stroke,” J Nanobiotechnology 22, no. 1 (2024): 291. 38802919 10.1186/s12951-024-02560-y PMC11129432

[382] C. Ding , B. Wang , J. Zheng , et al., “Neutrophil Membrane‐Inspired Nanorobots Act as Antioxidants Ameliorate Ischemia Reperfusion‐Induced Acute Kidney Injury,” ACS Appl Mater Interfaces 15, no. 34 (2023): 40292‐40303. 37603686 10.1021/acsami.3c08573

[383] W. Feng , N. Zhu , Y. Xia , et al., “Melanin‐Like nanoparticles alleviate ischemia‐reperfusion injury in the kidney by scavenging reactive oxygen species and inhibiting ferroptosis,” Iscience 27, no. 4 (2024): 109504. 38632989 10.1016/j.isci.2024.109504 PMC11022057

[384] X. Zhao , Z. Wang , G. Wu , et al., “Apigenin‐7‐glucoside‐loaded nanoparticle alleviates intestinal ischemia‐reperfusion by ATF3/SLC7A11‐mediated ferroptosis,” J Control Release Off J Control Release Soc 366 (2024): 182‐193. 10.1016/j.jconrel.2023.12.038 38145659

[385] H. Yu , Z. Song , J. Yu , et al., “Supramolecular self‐assembly of EGCG‐selenomethionine nanodrug for treating osteoarthritis,” Bioact Mater 32 (2024): 164‐176. 37822916 10.1016/j.bioactmat.2023.09.020 PMC10563013

[386] W. Li , Z. Lv , P. Wang , et al., “Near Infrared Responsive Gold Nanorods Attenuate Osteoarthritis Progression by Targeting TRPV1,” Adv Sci Weinh Baden‐Wurtt Ger 11, no. 16 (2024): e2307683. 10.1002/advs.202307683 PMC11040380 38358041

[387] Y. Li , Z. Cai , W. Ma , L. Bai , E. Luo , and Y. Lin , “A DNA tetrahedron‐based ferroptosis‐suppressing nanoparticle: Superior delivery of curcumin and alleviation of diabetic osteoporosis,” Bone Res 12, no. 1 (2024): 14. 38424439 10.1038/s41413-024-00319-7 PMC10904802

[388] C. Wu and K. Shemisa , “Sorafenib‐Associated Heart Failure Complicated by Cardiogenic Shock After Treatment of Advanced Stage Hepatocellular Carcinoma: A Clinical Case Discussion,” Case Rep Cardiol 2017 (2017): 7065759. 28536660 10.1155/2017/7065759 PMC5425844

[389] Y. Xie , W. Hou , X. Song , et al., “Ferroptosis: Process and function,” Cell Death and Differentiation 23, no. 3 (2016): 369‐379. 26794443 10.1038/cdd.2015.158 PMC5072448

[390] X. Fang , Z. Cai , H. Wang , et al., “Loss of Cardiac Ferritin H Facilitates Cardiomyopathy via Slc7a11‐Mediated Ferroptosis,” Circulation Research 127, no. 4 (2020): 486‐501. 32349646 10.1161/CIRCRESAHA.120.316509

[391] X. Fang , H. Wang , D. Han , et al., “Ferroptosis as a target for protection Against cardiomyopathy,” Pnas 116, no. 7 (2019): 2672‐2680. 30692261 10.1073/pnas.1821022116 PMC6377499

[392] W. Li , G. Feng , J. M. Gauthier , et al., “Ferroptotic cell death and TLR4/Trif signaling initiate neutrophil recruitment After heart transplantation,” Journal of Clinical Investigation 129, no. 6 (2019): 2293‐2304. 30830879 10.1172/JCI126428 PMC6546457

[393] Y. Yu , L. Jiang , H. Wang , et al., “Hepatic transferrin plays a role in systemic iron homeostasis and liver ferroptosis,” Blood 136, no. 6 (2020): 726‐739. 32374849 10.1182/blood.2019002907 PMC7414596

[394] H. Wang , P. An , E. Xie , et al., “Characterization of ferroptosis in murine models of hemochromatosis,” Hepatology 66, no. 2 (2017): 449. 28195347 10.1002/hep.29117 PMC5573904

[395] H. S. Kain , E. K. K. Glennon , K. Vijayan , et al., “Liver stage malaria infection is controlled by host regulators of lipid peroxidation,” Cell Death and Differentiation 27, no. 1 (2020): 44‐54. 31065106 10.1038/s41418-019-0338-1 PMC7206113

[396] E. Dai , W. Zhang , D. Cong , R. Kang , J. Wang , and D. Tang , “AIFM2 blocks ferroptosis independent of ubiquinol metabolism,” Biochemical and Biophysical Research Communications 523, no. 4 (2020): 966‐971. 31964528 10.1016/j.bbrc.2020.01.066

[397] S. Y. Xu , S. S. Yin , L. Wang , H. Zhong , H. Wang , and H. Y. Yu , “Insights Into emerging mechanisms of ferroptosis: New regulators for cancer therapeutics,” Cell Biology and Toxicology 41, no. 1 (2025): 63. 40131564 10.1007/s10565-025-10010-0 PMC11937073

[398] D. Jiao , Y. Yang , K. Wang , and Y. Wang , “Ferroptosis: A novel pathogenesis and therapeutic strategies for Parkinson disease: A review,” Medicine 104, no. 3 (2025): e41218. 39833092 10.1097/MD.0000000000041218 PMC11749581

[399] F. Alves , D. Lane , T. P. M. Nguyen , A. I. Bush , and S. Ayton , “In defence of ferroptosis,” Signal Transduct Target Ther 10, no. 1 (2025): 1‐29. 39746918 10.1038/s41392-024-02088-5 PMC11696223

[400] Y. Wang , L. Yang , X. Zhang , et al., “Epigenetic regulation of ferroptosis by H2B monoubiquitination and p53,” Embo Reports 20, no. 7 (2019): e47563. 31267712 10.15252/embr.201847563 PMC6607012

[401] L. Jiang , N. Kon , T. Li , et al., “Ferroptosis as a p53‐mediated activity During tumour suppression,” Nature 520, no. 7545 (2015): 57‐62. 25799988 10.1038/nature14344 PMC4455927

[402] B. Chu , N. Kon , D. Chen , et al., “ALOX12 is required for p53‐mediated tumour suppression Through a distinct ferroptosis pathway,” Nature Cell Biology 21, no. 5 (2019): 579‐591. 30962574 10.1038/s41556-019-0305-6 PMC6624840

[403] D. E. Jensen , M. Proctor , S. T. Marquis , et al., “BAP1: A novel ubiquitin hydrolase which binds to the BRCA1 RING finger and enhances BRCA1‐mediated cell growth suppression,” Oncogene 16, no. 9 (1998): 1097‐1112. 9528852 10.1038/sj.onc.1201861

[404] Y. Zhang , J. Shi , X. Liu , et al., “BAP1 links metabolic regulation of ferroptosis to tumour suppression,” Nature Cell Biology 20, no. 10 (2018): 1181‐1192. 30202049 10.1038/s41556-018-0178-0 PMC6170713

[405] N. A. Alam , S. Olpin , A. Rowan , et al., “Missense mutations in fumarate hydratase in multiple cutaneous and uterine leiomyomatosis and renal cell cancer,” J Mol Diagn JMD 7, no. 4 (2005): 437‐443. 16237213 10.1016/S1525-1578(10)60574-0 PMC1888487

[406] G. S. Chuang , A. Martinez‐Mir , A. Geyer , et al., “Germline fumarate hydratase mutations and evidence for a founder mutation underlying multiple cutaneous and uterine leiomyomata,” Journal of the American Academy of Dermatology 52 (2005): 410‐416. 3 Pt 1. 15761418 10.1016/j.jaad.2004.08.051

[407] M. Gao , J. Yi , J. Zhu , et al., “Role of Mitochondria in Ferroptosis,” Molecular Cell 73, no. 2 (2019): 354‐363. e3. 30581146 10.1016/j.molcel.2018.10.042 PMC6338496

[408] P. S. Hammerman , M. S. Lawrence , D. Voet , et al., “Comprehensive genomic characterization of squamous cell lung cancers,” Nature 489, no. 7417 (2012): 519‐525. 22960745 10.1038/nature11404 PMC3466113

[409] S. Scalera , M. Mazzotta , C. Cortile , et al., “KEAP1‐Mutant NSCLC: The Catastrophic Failure of a Cell‐Protecting Hub,” Journal of thoracic oncology 17, no. 6 (2022): 751‐757. 35351670 10.1016/j.jtho.2022.03.011

[410] P. Koppula , G. Lei , Y. Zhang , et al., “A targetable CoQ‐FSP1 axis drives ferroptosis‐ and radiation‐resistance in KEAP1 inactive lung cancers,” Nature Communications 13, no. 1 (2022): 2206. 10.1038/s41467-022-29905-1 PMC9033817 35459868

[411] Z. Fan , A. K. Wirth , D. Chen , et al., “Nrf2‐Keap1 pathway promotes cell proliferation and diminishes ferroptosis,” Oncogenesis 6, no. 8 (2017): e371‐e371. 28805788 10.1038/oncsis.2017.65 PMC5608917

[412] S. Egolf , J. Zou , A. Anderson , et al., “MLL4 mediates differentiation and tumor suppression Through ferroptosis,” Science Advances 7, no. 50 (2021): eabj9141. 34890228 10.1126/sciadv.abj9141 PMC8664260

[413] L. Kuroki and S. R. Guntupalli , “Treatment of epithelial ovarian cancer,” Bmj 371 (2020): m3773. 33168565 10.1136/bmj.m3773

[414] Y. Liu , X. Liu , H. Wang , P. Ding , and C. Wang , “Agrimonolide inhibits cancer progression and induces ferroptosis and apoptosis by targeting SCD1 in ovarian cancer cells,” Phytomedicine Int J Phytother Phytopharm 101 (2022): 154102. 10.1016/j.phymed.2022.154102 35526323

[415] H. Wang , M. G. Klein , H. Zou , et al., “Crystal structure of human stearoyl‐coenzyme A desaturase in complex With substrate,” Nature structural & molecular biology 22, no. 7 (2015): 581‐585. 10.1038/nsmb.3049 26098317

[416] M. T. Bohnsack and K. E. Sloan , “The mitochondrial epitranscriptome: The roles of RNA modifications in mitochondrial translation and human disease,” Cellular and molecular life sciences CMLS 75, no. 2 (2018): 241‐260. 28752201 10.1007/s00018-017-2598-6 PMC5756263

[417] S. Delaunay and M. Frye , “RNA modifications regulating cell fate in cancer,” Nature Cell Biology 21, no. 5 (2019): 552‐559. 31048770 10.1038/s41556-019-0319-0

[418] Z. Shi , S. Xu , S. Xing , et al., “Mettl17, a regulator of mitochondrial ribosomal RNA modifications, is required for the translation of mitochondrial coding genes,” FASEB J Off Publ Fed Am Soc Exp Biol 33, no. 11 (2019): 13040‐13050. 10.1096/fj.201901331R 31487196

[419] H. Li , K. Yu , H. Hu , et al., “METTL17 coordinates ferroptosis and tumorigenesis by regulating mitochondrial translation in colorectal cancer,” Redox Biology 71 (2024): 103087. 38377789 10.1016/j.redox.2024.103087 PMC10884776

[420] R. Wei , Y. Zhao , J. Wang , et al., “Tagitinin C induces ferroptosis Through PERK‐Nrf2‐HO‐1 signaling pathway in colorectal cancer cells,” Int J Biol Sci 17, no. 11 (2021): 2703‐2717. 34345202 10.7150/ijbs.59404 PMC8326123

[421] X. Miao and N. Zhang , “Role of RBM3 in the regulation of cell proliferation in hepatocellular carcinoma,” Experimental and Molecular Pathology 117 (2020): 104546. 32976820 10.1016/j.yexmp.2020.104546

[422] N. Melling , K. Bachmann , B. Hofmann , et al., “Prevalence and clinical significance of RBM3 immunostaining in non‐small cell lung cancers,” Journal of Cancer Research and Clinical Oncology 145, no. 4 (2019): 873‐879. 30758670 10.1007/s00432-019-02850-1 PMC11810404

[423] Z. Wang , W. Shu , R. Zhao , Y. Liu , and H. Wang , “Sodium butyrate induces ferroptosis in endometrial cancer cells via the RBM3/SLC7A11 axis,” Apoptosis Int J Program Cell Death 28, no. 7‐8 (2023): 1168‐1183. 10.1007/s10495-023-01850-4 37170022

[424] A. E. Whiteley , T. T. Price , G. Cantelli , and D. A. Sipkins , “Leukaemia: A model metastatic disease,” Nature Reviews Cancer 21, no. 7 (2021): 461‐475. 33953370 10.1038/s41568-021-00355-z PMC8722462

[425] J. Du , T. Wang , Y. Li , et al., “DHA inhibits proliferation and induces ferroptosis of leukemia cells Through autophagy dependent degradation of ferritin,” Free Radic Biol Med 131 (2019): 356‐369. 30557609 10.1016/j.freeradbiomed.2018.12.011

[426] J. Jiang , H. L. Liu , L. Tao , et al., “Let‑7d inhibits colorectal cancer cell proliferation Through the CST1/p65 pathway,” International Journal of Oncology 53, no. 2 (2018): 781‐790. 29845224 10.3892/ijo.2018.4419

[427] D. Li , Y. Wang , C. Dong , et al., “CST1 inhibits ferroptosis and promotes gastric cancer metastasis by regulating GPX4 protein stability via OTUB1,” Oncogene 42, no. 2 (2023): 83‐98. 36369321 10.1038/s41388-022-02537-x PMC9816059

[428] X. Guan , Y. Wang , W. Yu , et al., “Blocking Ubiquitin‐Specific Protease 7 Induces Ferroptosis in Gastric Cancer via Targeting Stearoyl‐CoA Desaturase,” Adv Sci Weinh Baden‐Wurtt Ger 11, no. 18 (2024): e2307899. 10.1002/advs.202307899 PMC11095140 38460164

[429] J. Gu , R. Sun , D. Tang , F. Liu , X. Chang , and Q. Wang , “Astragalus mongholicus Bunge‐Curcuma aromatica Salisb. suppresses growth and metastasis of colorectal cancer cells by inhibiting M2 macrophage polarization via a Sp1/ZFAS1/miR‐153‐3p/CCR5 regulatory axis,” Cell Biology and Toxicology 38, no. 4 (2022): 679‐697. 35072892 10.1007/s10565-021-09679-w

[430] C. Yi , S. Wu , Q. Duan , et al., “Ferroptosis‐dependent breast cancer cell‐derived exosomes inhibit migration and invasion of breast cancer cells by suppressing M2 macrophage polarization,” PeerJ 11 (2023): e15060. 36949762 10.7717/peerj.15060 PMC10026718

[431] C. Q. Wang , C. H. Tang , Y. Wang , et al., “Upregulated WTAP expression appears to both promote breast cancer growth and inhibit lymph node metastasis,” Scientific Reports 12, no. 1 (2022): 1023. 35046505 10.1038/s41598-022-05035-y PMC8770795

[432] J. Liu , X. Song , F. Kuang , et al., “NUPR1 is a critical repressor of ferroptosis,” Nature Communications 12, no. 1 (2021): 647. 10.1038/s41467-021-20904-2 PMC7843652 33510144

[433] M. Tan , Y. He , J. Yi , et al., “WTAP Mediates NUPR1 Regulation of LCN2 Through m6A Modification to Influence Ferroptosis, Thereby Promoting Breast Cancer Proliferation, Migration and Invasion,” Biochemical Genetics 62, no. 2 (2024): 876‐891. 37477758 10.1007/s10528-023-10423-8

[434] M. Jiang , Y. Jike , K. Liu , et al., “Exosome‐mediated miR‐144‐3p promotes ferroptosis to inhibit osteosarcoma proliferation, migration, and invasion Through regulating ZEB1,” Molecular cancer 22, no. 1 (2023): 113. 37461104 10.1186/s12943-023-01804-z PMC10351131

[435] M. J. Rane , Y. Zhao , and L. Cai , “Krϋppel‐Like factors (KLFs) in renal physiology and disease,” EBioMedicine 40 (2019): 743‐750. 30662001 10.1016/j.ebiom.2019.01.021 PMC6414320

[436] Y. Lu , H. Qin , B. Jiang , et al., “KLF2 inhibits cancer cell migration and invasion by regulating ferroptosis Through GPX4 in clear cell renal cell carcinoma,” Cancer Letters 522 (2021): 1‐13. 34520818 10.1016/j.canlet.2021.09.014

[437] R. L. Siegel , K. D. Miller , H. E. Fuchs , and A. Jemal , “Cancer Statistics, 2021,” CA: A Cancer Journal for Clinicians 71, no. 1 (2021): 7‐33. 33433946 10.3322/caac.21654

[438] X. Zhang , X. Zheng , X. Ying , W. Xie , Y. Yin , and X. Wang , “CEBPG suppresses ferroptosis Through transcriptional control of SLC7A11 in ovarian cancer,” Journal of translational medicine 21, no. 1 (2023): 334. 37210575 10.1186/s12967-023-04136-0 PMC10199564

[439] D. P. Modest , I. Ricard , and V. Heinemann , “Outcome according to KRAS‐, NRAS‐ and BRAF‐mutation as well as KRAS mutation variants: Pooled analysis of five randomized trials in metastatic colorectal cancer by the AIO colorectal cancer study group,” Ann Oncol Off J Eur Soc Med Oncol 27, no. 9 (2016): 1746‐1753. 10.1093/annonc/mdw261 PMC4999563 27358379

[440] Q. Miao , W. Q. Deng , W. Y. Lyu , et al., “Erianin inhibits the growth and metastasis Through autophagy‐dependent ferroptosis in KRASG13D colorectal cancer,” Free Radic Biol Med 204 (2023): 301‐312. 37217090 10.1016/j.freeradbiomed.2023.05.008

[441] J. M. Baughman , F. Perocchi , H. S. Girgis , et al., “Integrative genomics identifies MCU as an essential component of the mitochondrial calcium uniporter,” Nature 476, no. 7360 (2011): 341‐345. 21685886 10.1038/nature10234 PMC3486726

[442] X. Wang , Y. Li , Z. Li , et al., “Mitochondrial Calcium Uniporter Drives Metastasis and Confers a Targetable Cystine Dependency in Pancreatic Cancer,” Cancer Research 82, no. 12 (2022): 2254‐2268. 35413105 10.1158/0008-5472.CAN-21-3230 PMC9203979

[443] T. W. Lager , C. Conner , C. R. Keating , J. N. Warshaw , and A. D. Panopoulos , “Cell surface GRP78 and Dermcidin cooperate to regulate breast cancer cell migration Through Wnt signaling,” Oncogene 40, no. 23 (2021): 4050‐4059. 33981001 10.1038/s41388-021-01821-6 PMC8197743

[444] S. Samanta , S. Yang , B. Debnath , et al., “The Hydroxyquinoline Analogue YUM70 Inhibits GRP78 to Induce ER Stress‐Mediated Apoptosis in Pancreatic Cancer,” Cancer Research 81, no. 7 (2021): 1883‐1895. 33531374 10.1158/0008-5472.CAN-20-1540 PMC8137563

[445] Q. Wang , S. Ke , Z. Liu , H. Shao , M. He , and J. Guo , “HSPA5 Promotes the Proliferation, Metastasis and Regulates Ferroptosis of Bladder Cancer,” International Journal of Molecular Sciences 24, no. 6 (2023): 5144. 36982218 10.3390/ijms24065144 PMC10048805

[446] J. Ketteler and D. Klein , “Caveolin‐1, cancer and therapy resistance,” International Journal of Cancer 143, no. 9 (2018): 2092‐2104. 29524224 10.1002/ijc.31369

[447] T. Lu , Z. Zhang , X. Pan , et al., “Caveolin‐1 promotes cancer progression via inhibiting ferroptosis in head and neck squamous cell carcinoma,” J Oral Pathol Med Off Publ Int Assoc Oral Pathol Am Acad Oral Pathol 51, no. 1 (2022): 52‐62. 10.1111/jop.13267 PMC9300096 34874578

[448] J. Paris , C. Wilhelm , C. Lebbé , et al., “PROM2 overexpression induces metastatic potential Through epithelial‐to‐mesenchymal transition and ferroptosis resistance in human cancers,” Clinical and translational medicine 14, no. 3 (2024): e1632. 38515278 10.1002/ctm2.1632 PMC10958126

[449] S. N. Dowland , R. J. Madawala , C. E. Poon , L. A. Lindsay , and C. R. Murphy , “Prominin‐2 Prevents the Formation of Caveolae in Normal and Ovarian Hyperstimulated Pregnancy,” Reprod Sci Thousand Oaks Calif 25, no. 8 (2018): 1231‐1242. 10.1177/1933719117737842 29113580

[450] V. J. N. Bykov , S. E. Eriksson , J. Bianchi , and K. G. Wiman , “Targeting mutant p53 for efficient cancer therapy,” Nature Reviews Cancer 18, no. 2 (2018): 89‐102. 29242642 10.1038/nrc.2017.109

[451] K. T. Bieging , S. S. Mello , and L. D. Attardi , “Unravelling mechanisms of p53‐mediated tumour suppression,” Nature Reviews Cancer 14, no. 5 (2014): 359‐370. 10.1038/nrc3711 PMC4049238 24739573

[452] G. Lei , Y. Zhang , T. Hong , et al., “Ferroptosis as a mechanism to mediate p53 function in tumor radiosensitivity,” Oncogene 40, no. 20 (2021): 3533‐3547. 33927351 10.1038/s41388-021-01790-w PMC8141034

[453] Y. Wang , L. Yang , X. Zhang , et al., “Epigenetic regulation of ferroptosis by H2B monoubiquitination and p53,” Embo Reports 20, no. 7 (2019): e47563. 31267712 10.15252/embr.201847563 PMC6607012

[454] Y. Ou , S. J. Wang , D. Li , B. Chu , and W. Gu , “Activation of SAT1 engages polyamine metabolism With p53‐mediated ferroptotic responses,” PNAS 113, no. 44 (2016): E6806‐E6812. 27698118 10.1073/pnas.1607152113 PMC5098629

[455] M. Jennis , C. P. Kung , S. Basu , et al., “An African‐specific polymorphism in the TP53 gene impairs p53 tumor suppressor function in a mouse model,” Genes & development 30, no. 8 (2016): 918‐930. 27034505 10.1101/gad.275891.115 PMC4840298

[456] L. Jiang , N. Kon , T. Li , et al., “Ferroptosis as a p53‐mediated activity During tumour suppression,” Nature 520, no. 7545 (2015): 57‐62. 25799988 10.1038/nature14344 PMC4455927

[457] A. Tarangelo , L. Magtanong , K. T. Bieging‐Rolett , et al., “p53 Suppresses Metabolic Stress‐Induced Ferroptosis in Cancer Cells,” Cell reports 22, no. 3 (2018): 569‐575. 29346757 10.1016/j.celrep.2017.12.077 PMC5791910

[458] M. Carbone , H. Yang , H. I. Pass , T. Krausz , J. R. Testa , and G. Gaudino , “BAP1 and cancer,” Nature Reviews Cancer 13, no. 3 (2013): 153‐159. 10.1038/nrc3459 PMC3792854 23550303

[459] K. H. Ventii , N. S. Devi , K. L. Friedrich , et al., “BRCA1‐associated protein‐1 is a tumor suppressor that requires deubiquitinating activity and nuclear localization,” Cancer Research 68, no. 17 (2008): 6953‐6962. 18757409 10.1158/0008-5472.CAN-08-0365 PMC2736608

[460] M. Carbone , J. W. Harbour , J. Brugarolas , et al., “Biological Mechanisms and Clinical Significance of BAP1 Mutations in Human Cancer,” Cancer discovery 10, no. 8 (2020): 1103‐1120. 32690542 10.1158/2159-8290.CD-19-1220 PMC8006752

[461] Cancer Genome Atlas Research Network . Comprehensive genomic characterization of squamous cell lung cancers. Nature 2012;489(7417):519‐525. 22960745 10.1038/nature11404 PMC3466113

[462] Cancer Genome Atlas Research Network . Comprehensive molecular profiling of lung adenocarcinoma. Nature 2014;511(7511):543‐550. 25079552 10.1038/nature13385 PMC4231481

[463] L. Baird and M. Yamamoto , “The Molecular Mechanisms Regulating the KEAP1‐NRF2 Pathway,” Molecular and Cellular Biology 40, no. 13 (2020): e00099‐20. 32284348 10.1128/MCB.00099-20 PMC7296212

[464] T. W. Kensler , N. Wakabayashi , and S. Biswal , “Cell survival responses to environmental stresses via the Keap1‐Nrf2‐ARE pathway,” Annual Review of Pharmacology and Toxicology 47 (2007): 89‐116. 10.1146/annurev.pharmtox.46.120604.141046 16968214

[465] D. Chen , O. Tavana , B. Chu , et al., “NRF2 Is a Major Target of ARF in p53‐Independent Tumor Suppression,” Molecular Cell 68, no. 1 (2017): 224‐232. e4. 28985506 10.1016/j.molcel.2017.09.009 PMC5683418

[466] L. Jiang , N. Kon , T. Li , et al., “Ferroptosis as a p53‐mediated activity During tumour suppression,” Nature 520, no. 7545 (2015): 57‐62. 25799988 10.1038/nature14344 PMC4455927

[467] E. Kastenhuber and S. Lowe , “Putting p53 in context,” Cell 170, no. 6 (2017): 1062‐1078. 28886379 10.1016/j.cell.2017.08.028 PMC5743327

[468] T. Li , N. Kon , L. Jiang , et al., “Tumor suppression in the absence of p53‐mediated cell cycle arrest, apoptosis, and senescence,” Cell 149, no. 6 (2012): 1269‐1283. 22682249 10.1016/j.cell.2012.04.026 PMC3688046

[469] Y. Liu and W. Gu , “p53 in ferroptosis regulation: The new weapon for the old guardian,” Cell Death and Differentiation 29, no. 5 (2022): 895‐910. 35087226 10.1038/s41418-022-00943-y PMC9091200

[470] Y. Ou , S. J. Wang , L. Jiang , B. Zheng , and W. Gu , “p53 Protein‐mediated Regulation of Phosphoglycerate Dehydrogenase (PHGDH) Is Crucial for the Apoptotic Response upon Serine Starvation,” Journal of Biological Chemistry 290, no. 1 (2015): 457‐466. 25404730 10.1074/jbc.M114.616359 PMC4281747

[471] M. Wang , C. Mao , L. Ouyang , et al., “Long noncoding RNA LINC00336 inhibits ferroptosis in lung cancer by functioning as a competing endogenous RNA,” Cell Death and Differentiation 26, no. 11 (2019): 2329‐2343. 30787392 10.1038/s41418-019-0304-y PMC6889193

[472] S. J. Wang , D. Li , Y. Ou , et al., “Acetylation Is Crucial for p53‐mediated Ferroptosis and Tumor Suppression,” Cell reports 17, no. 2 (2016): 366‐373. 27705786 10.1016/j.celrep.2016.09.022 PMC5227654

[473] T. Thomas and T. J. Thomas , “Polyamine metabolism and cancer,” Journal of Cellular and Molecular Medicine 7, no. 2 (2003): 113‐126. 12927050 10.1111/j.1582-4934.2003.tb00210.x PMC6740079

[474] Y. Ou , S. J. Wang , D. Li , B. Chu , and W. Gu , “Activation of SAT1 engages polyamine metabolism With p53‐mediated ferroptotic responses,” PNAS 113, no. 44 (2016): E6806‐E6812. 27698118 10.1073/pnas.1607152113 PMC5098629

[475] B. Chu , N. Kon , D. Chen , et al., “ALOX12 is required for p53‐mediated tumor suppression Through a distinct ferroptosis pathway,” Nature Cell Biology 21, no. 5 (2019): 579‐591. 30962574 10.1038/s41556-019-0305-6 PMC6624840

[476] X. Yang , J. Liu , C. Wang , et al., “miR‐18a promotes glioblastoma development by Down‐regulating ALOXE3‐mediated ferroptotic and anti‐migration activities,” Oncogenesis 10, no. 2 (2021): 15. 33579899 10.1038/s41389-021-00304-3 PMC7881152

[477] W. Hu , C. Zhang , R. Wu , Y. Sun , A. Levine , and Z. Feng , “Glutaminase 2, a novel p53 target gene regulating energy metabolism and antioxidant function,” PNAS 107, no. 16 (2010): 7455‐7460. 20378837 10.1073/pnas.1001006107 PMC2867677

[478] M. Kobayashi and M. Yamamoto , “Nrf2‐Keap1 regulation of cellular defense mechanisms Against electrophiles and reactive oxygen species,” Advances in Enzyme Regulation 46 (2006): 113‐140. 16887173 10.1016/j.advenzreg.2006.01.007

[479] D. D. Zhang , S. C. Lo , J. V. Cross , D. J. Templeton , and M. Hannink , “Keap1 is a redox‐regulated substrate adaptor protein for a Cul3‐dependent ubiquitin ligase complex,” Molecular and Cellular Biology 24, no. 24 (2004): 10941‐10953. 15572695 10.1128/MCB.24.24.10941-10953.2004 PMC533977

[480] J. W. Kaspar , S. K. Niture , and A. K. Jaiswal , “Nrf2:INrf2 (Keap1) signaling in oxidative stress,” Free Radic Biol Med 47, no. 9 (2009): 1304‐1309. 19666107 10.1016/j.freeradbiomed.2009.07.035 PMC2763938

[481] M. K. Kwak , J. M. Cho , B. Huang , S. Shin , and T. W. Kensler , “Role of increased expression of the proteasome in the protective effects of sulforaphane Against hydrogen peroxide‐mediated cytotoxicity in murine neuroblastoma cells,” Free Radic Biol Med 43, no. 5 (2007): 809‐817. 17664144 10.1016/j.freeradbiomed.2007.05.029

[482] C. Tonelli , I. I. C. Chio , and D. A. Tuveson , “Transcriptional Regulation by Nrf2,” Antioxid Redox Signaling 29, no. 17 (2018): 1727‐1745. 10.1089/ars.2017.7342 PMC6208165 28899199

[483] M. Nezu , N. Suzuki , and M. Yamamoto , “Targeting the KEAP1‐NRF2 System to Prevent Kidney Disease Progression,” American Journal of Nephrology 45, no. 6 (2017): 473‐483. 28502971 10.1159/000475890

[484] P. Koppula , G. Lei , Y. Zhang , et al., “A targetable CoQ‐FSP1 axis drives ferroptosis‐ and radiation‐resistance in KEAP1 inactive lung cancers,” Nature Communications 13, no. 1 (2022): 2206. 10.1038/s41467-022-29905-1 PMC9033817 35459868

[485] D. A. Guertin and D. M. Sabatini , “Defining the role of mTOR in cancer,” Cancer Cell 12, no. 1 (2007): 9‐22. 17613433 10.1016/j.ccr.2007.05.008

[486] T. Porstmann , C. R. Santos , B. Griffiths , et al., “SREBP activity is regulated by mTORC1 and contributes to Akt‐dependent cell growth,” Cell metabolism 8, no. 3 (2008): 224‐236. 18762023 10.1016/j.cmet.2008.07.007 PMC2593919

[487] J. Yi , J. Zhu , J. Wu , C. B. Thompson , and X. Jiang , “Oncogenic activation of PI3K‐AKT‐mTOR signaling suppresses ferroptosis via SREBP‐mediated lipogenesis,” PNAS 117, no. 49 (2020): 31189‐31197. 33229547 10.1073/pnas.2017152117 PMC7733797

[488] Y. Zhang , R. V. Swanda , L. Nie , et al., “mTORC1 couples cyst(e)ine availability With GPX4 protein synthesis and ferroptosis regulation,” Nature Communications 12, no. 1 (2021): 1589. 10.1038/s41467-021-21841-w PMC7952727 33707434

[489] R. Shen , A. Martin , A. Ni , et al., “Harnessing Clinical Sequencing Data for Survival Stratification of Patients With Metastatic Lung Adenocarcinomas,” JCO Precis Oncol 3 (2019): PO.18.00307. 10.1200/PO.18.00307 PMC6474404 31008437

[490] C. A. Wohlhieter , A. L. Richards , F. Uddin , et al., “Concurrent Mutations in STK11 and KEAP1 Promote Ferroptosis Protection and SCD1 Dependence in Lung Cancer,” Cell reports 33, no. 9 (2020): 108444. 33264619 10.1016/j.celrep.2020.108444 PMC7722473

[491] B. Keith , R. S. Johnson , and M. C. Simon , “HIF1α and HIF2α: Sibling rivalry in hypoxic tumour growth and progression,” Nature Reviews Cancer 12, no. 1 (2012): 9‐22. 10.1038/nrc3183 PMC3401912 22169972

[492] V. Infantino , A. Santarsiero , P. Convertini , S. Todisco , and V. Iacobazzi , “Cancer Cell Metabolism in Hypoxia: Role of HIF‐1 as Key Regulator and Therapeutic Target,” International Journal of Molecular Sciences 22, no. 11 (2021): 5703. 34071836 10.3390/ijms22115703 PMC8199012

[493] S. Sun , C. Guo , T. Gao , et al., “Hypoxia Enhances Glioma Resistance to Sulfasalazine‐Induced Ferroptosis by Upregulating SLC7A11 via PI3K/AKT/HIF‐1 α Axis,” Oxid Med Cell Longev 2022 (2022): 7862430. 36439690 10.1155/2022/7862430 PMC9699746

[494] Z. Lin , J. Song , Y. Gao , et al., “Hypoxia‐induced HIF‐1α/lncRNA‐PMAN inhibits ferroptosis by promoting the cytoplasmic translocation of ELAVL1 in peritoneal dissemination From gastric cancer,” Redox Biology 52 (2022): 102312. 35447413 10.1016/j.redox.2022.102312 PMC9043498

[495] H. Choudhry and A. L. Harris , “Advances in Hypoxia‐Inducible Factor Biology,” Cell metabolism 27, no. 2 (2018): 281‐298. 29129785 10.1016/j.cmet.2017.10.005

[496] S. Cai , Z. Ding , X. Liu , and J. Zeng , “Trabectedin induces ferroptosis via regulation of HIF‐1α/IRP1/TFR1 and Keap1/Nrf2/GPX4 axis in non‐small cell lung cancer cells,” Chemico‐Biological Interactions 369 (2023): 110262. 36396105 10.1016/j.cbi.2022.110262

[497] Y. S. Green , M. C. Ferreira dos Santos , D. G. Fuja , et al., “ISCA2 inhibition decreases HIF and induces ferroptosis in clear cell renal carcinoma,” Oncogene 41, no. 42 (2022): 4709‐4723. 36097192 10.1038/s41388-022-02460-1 PMC9568429

[498] B. Zhao , Q. Y. Lei , and K. L. Guan , “The Hippo‐YAP pathway: New connections Between regulation of organ size and cancer,” Current Opinion in Cell Biology 20, no. 6 (2008): 638‐646. 18955139 10.1016/j.ceb.2008.10.001 PMC3296452

[499] F. van Roy and G. Berx , “The cell‐cell adhesion molecule E‐cadherin,” Cellular and molecular life sciences CMLS 65, no. 23 (2008): 3756‐3788. 18726070 10.1007/s00018-008-8281-1 PMC11131785

[500] N. G. Kim , E. Koh , X. Chen , and B. M. Gumbiner , “E‐cadherin mediates contact inhibition of proliferation Through Hippo signaling‐pathway components,” PNAS 108, no. 29 (2011): 11930‐11935. 21730131 10.1073/pnas.1103345108 PMC3141988

[501] J. Wu , A. M. Minikes , M. Gao , et al., “Intercellular interaction dictates cancer cell ferroptosis via NF2‐YAP signalling,” Nature 572, no. 7769 (2019): 402‐406. 31341276 10.1038/s41586-019-1426-6 PMC6697195

[502] W. Gan , X. Dai , X. Dai , et al., “LATS suppresses mTORC1 activity to directly coordinate Hippo and mTORC1 pathways in growth control,” Nature Cell Biology 22, no. 2 (2020): 246‐256. 32015438 10.1038/s41556-020-0463-6 PMC7076906

[503] J. M. Drijvers , J. E. Gillis , T. Muijlwijk , et al., “Pharmacologic Screening Identifies Metabolic Vulnerabilities of CD8+ T Cells,” Cancer immunology research 9, no. 2 (2021): 184‐199. 33277233 10.1158/2326-6066.CIR-20-0384 PMC7864883

[504] M. Matsushita , S. Freigang , C. Schneider , M. Conrad , G. W. Bornkamm , and M. Kopf , “T cell lipid peroxidation induces ferroptosis and prevents immunity to infection,” Journal of Experimental Medicine 212, no. 4 (2015): 555‐568. 25824823 10.1084/jem.20140857 PMC4387287

[505] J. M. Drijvers , J. E. Gillis , T. Muijlwijk , et al., “Pharmacologic screening identifies metabolic vulnerabilities of CD8+ T cells,” Cancer immunology research 9, no. 2 (2021): 184‐199. 33277233 10.1158/2326-6066.CIR-20-0384 PMC7864883

[506] S. D. Jeong , B. K. Jung , D. Lee , et al., “Enhanced Immunogenic Cell Death by Apoptosis/Ferroptosis Hybrid Pathway Potentiates PD‐L1 Blockade Cancer Immunotherapy,” ACS Biomater Sci Eng 8, no. 12 (2022): 5188‐5198. 36449494 10.1021/acsbiomaterials.2c00950

[507] C. Xu , S. Sun , T. Johnson , et al., “The glutathione peroxidase Gpx4 prevents lipid peroxidation and ferroptosis to sustain Treg cell activation and suppression of antitumor immunity,” Cell reports 35, no. 11 (2021): 109235. 34133924 10.1016/j.celrep.2021.109235

[508] A. A. Kapralov , Q. Yang , H. H. Dar , et al., “Redox lipid reprogramming commands susceptibility of macrophages and microglia to ferroptotic death,” Nature Chemical Biology 16, no. 3 (2020): 278‐290. 32080625 10.1038/s41589-019-0462-8 PMC7233108

[509] X. Luo , H. B. Gong , H. Y. Gao , et al., “Oxygenated phosphatidylethanolamine navigates phagocytosis of ferroptotic cells by interacting With TLR2,” Cell Death and Differentiation 28, no. 6 (2021): 1971‐1989. 33432112 10.1038/s41418-020-00719-2 PMC8185102

[510] Z. Shi , J. Zheng , W. Tang , et al., “Multifunctional Nanomaterials for Ferroptotic Cancer Therapy,” Front Chem 10 (2022): 868630. 35402376 10.3389/fchem.2022.868630 PMC8987283

[511] Z. Gu , T. Liu , C. Liu , et al., “Ferroptosis‐Strengthened Metabolic and Inflammatory Regulation of Tumor‐Associated Macrophages Provokes Potent Tumoricidal Activities,” Nano Letters 21, no. 15 (2021): 6471‐6479. 34292757 10.1021/acs.nanolett.1c01401

[512] K. Chen , H. Li , A. Zhou , et al., “Cell Membrane Camouflaged Metal Oxide–Black Phosphorus Biomimetic Nanocomplex Enhances Photo‐chemo‐dynamic Ferroptosis,” ACS Appl Mater Interfaces 14, no. 23 (2022): 26557‐26570. 35658416 10.1021/acsami.2c08413

[513] R. Kim , A. Hashimoto , N. Markosyan , et al., “Ferroptosis of tumour neutrophils causes immune suppression in cancer,” Nature 612, no. 7939 (2022): 338‐346. 36385526 10.1038/s41586-022-05443-0 PMC9875862

[514] F. Veglia , V. A. Tyurin , M. Blasi , et al., “Fatty acid transport protein 2 reprograms neutrophils in cancer,” Nature 569, no. 7754 (2019): 73‐78. 30996346 10.1038/s41586-019-1118-2 PMC6557120

[515] A. Ugolini , V. A. Tyurin , Y. Y. Tyurina , et al., “Polymorphonuclear myeloid‐derived suppressor cells limit antigen cross‐presentation by dendritic cells in cancer,” JCI Insight 5, no. 15 (2020): e138581. 138581. 32584791 10.1172/jci.insight.138581 PMC7455061

[516] M. K. Srivastava , P. Sinha , V. K. Clements , P. Rodriguez , and S. Ostrand‐Rosenberg , “Myeloid‐derived suppressor cells inhibit T‐cell activation by depleting cystine and cysteine,” Cancer Research 70, no. 1 (2010): 68‐77. 20028852 10.1158/0008-5472.CAN-09-2587 PMC2805057

[517] J. P. Friedmann Angeli , D. V. Krysko , and M. Conrad , “Ferroptosis at the crossroads of cancer‐acquired drug resistance and immune evasion,” Nature Reviews Cancer 19, no. 7 (2019): 405‐414. 31101865 10.1038/s41568-019-0149-1

[518] X. Luo , H. B. Gong , H. Y. Gao , et al., “Oxygenated phosphatidylethanolamine navigates phagocytosis of ferroptotic cells by interacting With TLR2,” Cell Death and Differentiation 28, no. 6 (2021): 1971‐1989. 33432112 10.1038/s41418-020-00719-2 PMC8185102

[519] J. P. Friedmann Angeli , M. Schneider , B. Proneth , et al., “Inactivation of the ferroptosis regulator Gpx4 triggers acute renal failure in mice,” Nature Cell Biology 16, no. 12 (2014): 1180‐1191. 25402683 10.1038/ncb3064 PMC4894846

[520] D. Wang and R. N. Dubois , “Eicosanoids and cancer,” Nature Reviews Cancer 10, no. 3 (2010): 181‐193. 20168319 10.1038/nrc2809 PMC2898136

[521] W. Wang , M. Green , J. E. Choi , et al., “CD8+ T cells regulate tumour ferroptosis During cancer immunotherapy,” Nature 569, no. 7755 (2019): 270‐274. 31043744 10.1038/s41586-019-1170-y PMC6533917

[522] D. H. Kim , W. D. Kim , S. K. Kim , D. H. Moon , and S. J. Lee , “TGF‐β1‐mediated repression of SLC7A11 drives vulnerability to GPX4 inhibition in hepatocellular carcinoma cells,” Cell death & disease 11, no. 5 (2020): 406. 32471991 10.1038/s41419-020-2618-6 PMC7260246

[523] J. Guo , B. Xu , Q. Han , et al., “Ferroptosis: A Novel Anti‐tumor Action for Cisplatin,” Cancer research and treatment: official journal of Korean Cancer Association 50, no. 2 (2018): 445‐460. 10.4143/crt.2016.572 PMC5912137 28494534

[524] S. Ma , E. S. Henson , Y. Chen , and S. B. Gibson , “Ferroptosis is induced following siramesine and lapatinib treatment of breast cancer cells,” Cell death & disease 7, no. 7 (2016): e2307. 27441659 10.1038/cddis.2016.208 PMC4973350

[525] Y. Yamaguchi , T. Kasukabe , and S. Kumakura , “Piperlongumine rapidly induces the death of human pancreatic cancer cells mainly Through the induction of ferroptosis,” International Journal of Oncology 52, no. 3 (2018): 1011‐1022. 29393418 10.3892/ijo.2018.4259

[526] C. Liang , X. Zhang , M. Yang , and X. Dong , “Recent Progress in Ferroptosis Inducers for Cancer Therapy,” Adv Mater Deerfield Beach Fla 31, no. 51 (2019): e1904197. 10.1002/adma.201904197 31595562

[527] H. Yamaguchi , J. L. Hsu , C. T. Chen , et al., “Caspase‐independent cell death is involved in the negative effect of EGF receptor inhibitors on cisplatin in non‐small cell lung cancer cells,” Clin Cancer Res Off J Am Assoc Cancer Res 19, no. 4 (2013): 845‐854. 10.1158/1078-0432.CCR-12-2621 PMC3703145 23344263

[528] L. Chen , X. Li , L. Liu , B. Yu , Y. Xue , and Y. Liu , “Erastin sensitizes glioblastoma cells to temozolomide by restraining xCT and cystathionine‐γ‐lyase function,” Oncology Reports 33, no. 3 (2015): 1465‐1474. 25585997 10.3892/or.2015.3712

[529] X. Tong , R. Tang , M. Xiao , et al., “Targeting cell death pathways for cancer therapy: Recent developments in necroptosis, pyroptosis, ferroptosis, and cuproptosis research,” J Hematol OncolJ Hematol Oncol 15 (2022): 174. 36482419 10.1186/s13045-022-01392-3 PMC9733270

[530] S. J. Dixon , D. N. Patel , M. Welsch , et al., “Pharmacological inhibition of cystine‐glutamate exchange induces endoplasmic reticulum stress and ferroptosis,” Elife 3 (2014): e02523. 24844246 10.7554/eLife.02523 PMC4054777

[531] W. S. Yang , R. SriRamaratnam , M. E. Welsch , et al., “Regulation of ferroptotic cancer cell death by GPX4,” Cell 156, no. 1‐2 (2014): 317‐331. 24439385 10.1016/j.cell.2013.12.010 PMC4076414

[532] Y. Zhang , H. Tan , J. D. Daniels , et al., “Imidazole Ketone Erastin Induces Ferroptosis and Slows Tumor Growth in a Mouse Lymphoma Model,” Cell Chem Biol 26, no. 5 (2019): 623‐633. e9. 30799221 10.1016/j.chembiol.2019.01.008 PMC6525071

[533] G. K. Balendiran , R. Dabur , and D. Fraser , “The role of glutathione in cancer,” Cell Biochemistry and Function 22, no. 6 (2004): 343‐352. 15386533 10.1002/cbf.1149

[534] C. Liang , X. Zhang , M. Yang , and X. Dong , “Recent Progress in Ferroptosis Inducers for Cancer Therapy,” Adv Mater Deerfield Beach Fla 31, no. 51 (2019): e1904197. 10.1002/adma.201904197 31595562

[535] A. Recasens and L. Munoz , “Targeting Cancer Cell Dormancy,” Trends in Pharmacological Sciences 40, no. 2 (2019): 128‐141. 30612715 10.1016/j.tips.2018.12.004

[536] H. L. Zhang , B. X. Hu , Z. P. Ye , et al., “TRPML1 triggers ferroptosis defense and is a potential therapeutic target in AKT‐hyperactivated cancer,” Science Translational Medicine 16, no. 753 (2024): eadk0330. 38924427 10.1126/scitranslmed.adk0330

[537] Z. Li , Z. M. Xu , W. P. Chen , et al., “Tumor‐repopulating cells evade ferroptosis via PCK2‐dependent phospholipid remodeling,” Nature Chemical Biology 20, no. 10 (2024): 1341‐1352. 38720107 10.1038/s41589-024-01612-6 PMC11427348

[538] L. Ma , C. Chen , C. Zhao , et al., “Targeting carnitine palmitoyl transferase 1A (CPT1A) induces ferroptosis and synergizes With immunotherapy in lung cancer,” Signal Transduct Target Ther 9, no. 1 (2024): 64. 38453925 10.1038/s41392-024-01772-w PMC10920667

[539] W. Wang , K. Lu , X. Jiang , et al., “Ferroptosis inducers enhanced cuproptosis induced by copper ionophores in primary liver cancer,” J Exp Clin Cancer Res CR 42, no. 1 (2023): 142. 37277863 10.1186/s13046-023-02720-2 PMC10242978

[540] Y. Li , H. Yan , X. Xu , H. Liu , C. Wu , and L. Zhao , “Erastin/sorafenib induces cisplatin‐resistant non‐small cell lung cancer cell ferroptosis Through inhibition of the Nrf2/xCT pathway,” Oncology letters 19, no. 1 (2020): 323‐333. 31897145 10.3892/ol.2019.11066 PMC6923844

[541] Q. Cheng , L. Bao , M. Li , K. Chang , and X. Yi , “Erastin synergizes With cisplatin via ferroptosis to inhibit ovarian cancer growth in vitro and in vivo,” Journal of Obstetrics and Gynaecology Research 47, no. 7 (2021): 2481‐2491. 33882617 10.1111/jog.14779

[542] Y. Shibata , H. Yasui , K. Higashikawa , N. Miyamoto , and Y. Kuge , “Erastin, a ferroptosis‐inducing agent, sensitized cancer cells to X‐ray irradiation via glutathione starvation in vitro and in vivo,” PLoS ONE 14, no. 12 (2019): e0225931. 31800616 10.1371/journal.pone.0225931 PMC6892486

[543] B. Tang , J. Zhu , Y. Wang , et al., “Targeted xCT‐mediated Ferroptosis and Protumoral Polarization of Macrophages Is Effective Against HCC and Enhances the Efficacy of the Anti‐PD‐1/L1 Response,” Adv Sci Weinh Baden‐Wurtt Ger 10, no. 2 (2023): e2203973. 10.1002/advs.202203973 PMC9839855 36442849

[544] Z. Yang , R. Huang , Y. Wang , et al., “SIRT6 drives sensitivity to ferroptosis in anaplastic thyroid cancer Through NCOA4‐dependent autophagy,” Am J Cancer Res 13, no. 2 (2023): 464‐474. 36895980 PMC9989618

[545] W. S. Yang , R. SriRamaratnam , M. E. Welsch , et al., “Regulation of ferroptotic cancer cell death by GPX4,” Cell 156, no. 1‐2 (2014): 317‐331. 24439385 10.1016/j.cell.2013.12.010 PMC4076414

[546] L. F. Ye , K. R. Chaudhary , F. Zandkarimi , et al., “Radiation‐Induced Lipid Peroxidation Triggers Ferroptosis and Synergizes With Ferroptosis Inducers,” Acs Chemical Biology 15, no. 2 (2020): 469‐484. 31899616 10.1021/acschembio.9b00939 PMC7180072

[547] L. Ferrada , M. J. Barahona , M. Vera , B. R. Stockwell , and F. Nualart , “Dehydroascorbic acid sensitizes cancer cells to system xc‐ inhibition‐induced ferroptosis by promoting lipid droplet peroxidation,” Cell death & disease 14, no. 9 (2023): 637. 37752118 10.1038/s41419-023-06153-9 PMC10522586

[548] G. Shan , G. Bi , G. Zhao , et al., “Inhibition of PKA/CREB1 pathway confers sensitivity to ferroptosis in non‐small cell lung cancer,” Respiratory Research 24, no. 1 (2023): 277. 37957645 10.1186/s12931-023-02567-3 PMC10644539

[549] S. L. Cramer , A. Saha , J. Liu , et al., “Systemic depletion of L‐cyst(e)ine With cyst(e)inase increases reactive oxygen species and suppresses tumor growth,” Nature Medicine 23, no. 1 (2017): 120‐127. 10.1038/nm.4232 PMC5218918 27869804

[550] S. W. Alvarez , V. O. Sviderskiy , E. M. Terzi , et al., “NFS1 undergoes positive selection in lung tumours and protects cells From ferroptosis,” Nature 551, no. 7682 (2017): 639‐643. 29168506 10.1038/nature24637 PMC5808442

[551] W. Wang , M. Green , J. E. Choi , et al., “CD8+ T cells regulate tumour ferroptosis During cancer immunotherapy,” Nature 569, no. 7755 (2019): 270‐274. 31043744 10.1038/s41586-019-1170-y PMC6533917

[552] Y. Bai , L. Meng , L. Han , et al., “Lipid storage and lipophagy regulates ferroptosis,” Biochemical and Biophysical Research Communications 508, no. 4 (2019): 997‐1003. 30545638 10.1016/j.bbrc.2018.12.039

[553] F. Yang , Y. Xiao , J. H. Ding , et al., “Ferroptosis heterogeneity in triple‐negative breast cancer reveals an innovative immunotherapy combination strategy,” Cell metabolism 35, no. 1 (2023): 84‐100. e8. 36257316 10.1016/j.cmet.2022.09.021

[554] S. Y. Park , K. J. Jeong , A. Poire , et al., “Irreversible HER2 inhibitors overcome resistance to the RSL3 ferroptosis inducer in non‐HER2 amplified luminal breast cancer,” Cell death & disease 14, no. 8 (2023): 532. 37596261 10.1038/s41419-023-06042-1 PMC10439209

[555] X. Zhang , S. Sui , L. Wang , et al., “Inhibition of tumor propellant glutathione peroxidase 4 induces ferroptosis in cancer cells and enhances anticancer effect of cisplatin,” Journal of Cellular Physiology 235, no. 4 (2020): 3425‐3437. 31556117 10.1002/jcp.29232 PMC13482695

[556] J. H. You , J. Lee , and J. L. Roh , “Mitochondrial pyruvate carrier 1 regulates ferroptosis in drug‐tolerant persister head and neck cancer cells via epithelial‐mesenchymal transition,” Cancer Letters 507 (2021): 40‐54. 33741422 10.1016/j.canlet.2021.03.013

[557] L. Zhou , J. Chen , R. Li , et al., “Metal‐Polyphenol‐Network Coated Prussian Blue Nanoparticles for Synergistic Ferroptosis and Apoptosis via Triggered GPX4 Inhibition and Concurrent In Situ Bleomycin Toxification,” Small Weinh Bergstr Ger 17, no. 47 (2021): e2103919. 10.1002/smll.202103919 34623753

[558] Q. Hu , W. Zhu , J. Du , et al., “A GPX4‐targeted photosensitizer to reverse hypoxia‐induced inhibition of ferroptosis for non‐small cell lung cancer therapy,” Chemical Science 14, no. 34 (2023): 9095‐9100. 37655031 10.1039/d3sc01597a PMC10466276

[559] X. Zhang , Y. Guo , H. Li , and L. Han , “FIN56, a novel ferroptosis inducer, triggers lysosomal membrane permeabilization in a TFEB‐dependent manner in glioblastoma,” Journal of Cancer 12, no. 22 (2021): 6610‐6619. 34659551 10.7150/jca.58500 PMC8517990

[560] G. Lei , Y. Zhang , P. Koppula , et al., “The role of ferroptosis in ionizing radiation‐induced cell death and tumor suppression,” Cell Research 30, no. 2 (2020): 146‐162. 31949285 10.1038/s41422-019-0263-3 PMC7015061

[561] M. M. Gaschler , A. A. Andia , H. Liu , et al., “FINO2 initiates ferroptosis Through GPX4 inactivation and iron oxidation,” Nature Chemical Biology 14, no. 5 (2018): 507‐515. 29610484 10.1038/s41589-018-0031-6 PMC5899674

[562] Z. He , H. Zhou , Y. Zhang , et al., “Oxygen‐boosted biomimetic nanoplatform for synergetic phototherapy/ferroptosis activation and reversal of immune‐suppressed tumor microenvironment,” Biomaterials 290 (2022): 121832. 36228518 10.1016/j.biomaterials.2022.121832

[563] T. Nakamura , C. Hipp , A. Santos Dias Mourão , et al., “Phase separation of FSP1 promotes ferroptosis,” Nature 619, no. 7969 (2023): 371‐377. 37380771 10.1038/s41586-023-06255-6 PMC10338336

[564] Y. Zhou , K. Chen , W. K. Lin , et al., “Photo‐Enhanced Synergistic Induction of Ferroptosis for Anti‐Cancer Immunotherapy,” Adv Healthc Mater 12, no. 27 (2023): e2300994. 37432874 10.1002/adhm.202300994 PMC11468986

[565] J. W. S. Cheu , D. Lee , Q. Li , et al., “Ferroptosis Suppressor Protein 1 Inhibition Promotes Tumor Ferroptosis and Anti‐tumor Immune Responses in Liver Cancer,” Cell Mol Gastroenterol Hepatol 16, no. 1 (2023): 133‐159. 36893885 10.1016/j.jcmgh.2023.03.001 PMC10230009

[566] M. Mu , Y. Wang , S. Zhao , et al., “Engineering a pH/Glutathione‐Responsive Tea Polyphenol Nanodevice as an Apoptosis/Ferroptosis‐Inducing Agent,” ACS Appl Bio Mater 3, no. 7 (2020): 4128‐4138. 10.1021/acsabm.0c00225 35025415

[567] X. Wang , M. Wu , X. Zhang , et al., “Hypoxia‐responsive nanoreactors based on self‐enhanced photodynamic sensitization and triggered ferroptosis for cancer synergistic therapy,” J Nanobiotechnology 19, no. 1 (2021): 204. 34238297 10.1186/s12951-021-00952-y PMC8265128

[568] C. Zhang , Z. Liu , Y. Zhang , L. Ma , E. Song , and Y. Song , “Iron free‘’ zinc oxide nanoparticles With ion‐leaking properties disrupt intracellular ROS and iron homeostasis to induce ferroptosis,” Cell death & disease 11, no. 3 (2020): 183. 32170066 10.1038/s41419-020-2384-5 PMC7070056

[569] Z. Shen , J. Song , B. C. Yung , Z. Zhou , A. Wu , and X. Chen , “Emerging Strategies of Cancer Therapy Based on Ferroptosis,” Adv Mater Deerfield Beach Fla 30, no. 12 (2018): e1704007. 10.1002/adma.201704007 PMC6377162 29356212

[570] M. Gao , J. Deng , F. Liu , et al., “Triggered ferroptotic polymer micelles for reversing multidrug resistance to chemotherapy,” Biomaterials 223 (2019): 119486. 31520887 10.1016/j.biomaterials.2019.119486

[571] W. Wang , M. Green , J. E. Choi , et al., “CD8+ T cells regulate tumour ferroptosis During cancer immunotherapy,” Nature 569, no. 7755 (2019): 270‐274. 31043744 10.1038/s41586-019-1170-y PMC6533917

[572] P. Liao , W. Wang , W. Wang , et al., “CD8+ T cells and fatty acids orchestrate tumor ferroptosis and immunity via ACSL4,” Cancer Cell 40, no. 4 (2022): 365‐378. e6. 35216678 10.1016/j.ccell.2022.02.003 PMC9007863

[573] X. Lang , M. D. Green , W. Wang , et al., “Radiotherapy and Immunotherapy Promote Tumoral Lipid Oxidation and Ferroptosis via Synergistic Repression of SLC7A11,” Cancer discovery 9, no. 12 (2019): 1673‐1685. 31554642 10.1158/2159-8290.CD-19-0338 PMC6891128

[574] Y. Xue , F. Lu , Z. Chang , et al., “Intermittent dietary methionine deprivation facilitates tumoral ferroptosis and synergizes With checkpoint blockade,” Nature Communications 14, no. 1 (2023): 4758. 10.1038/s41467-023-40518-0 PMC10409767 37553341

[575] H. L. Zhang , B. X. Hu , Z. L. Li , et al., “PKCβII phosphorylates ACSL4 to amplify lipid peroxidation to induce ferroptosis,” Nature Cell Biology 24, no. 1 (2022): 88‐98. 35027735 10.1038/s41556-021-00818-3

[576] E. Dai , L. Han , J. Liu , et al., “Autophagy‐dependent ferroptosis drives tumor‐associated macrophage polarization via release and uptake of oncogenic KRAS protein,” Autophagy 16, no. 11 (2020): 2069‐2083. 31920150 10.1080/15548627.2020.1714209 PMC7595620

[577] Q. Wen , J. Liu , R. Kang , B. Zhou , and D. Tang , “The release and activity of HMGB1 in ferroptosis,” Biochemical and Biophysical Research Communications 510, no. 2 (2019): 278‐283. 30686534 10.1016/j.bbrc.2019.01.090

[578] E. Dai , L. Han , J. Liu , et al., “Ferroptotic damage promotes pancreatic tumorigenesis Through a TMEM173/STING‐dependent DNA sensor pathway,” Nature Communications 11, no. 1 (2020): 6339. 10.1038/s41467-020-20154-8 PMC7732843 33311482

[579] J. P. Friedmann Angeli , D. V. Krysko , and M. Conrad , “Ferroptosis at the crossroads of cancer‐acquired drug resistance and immune evasion,” Nature Reviews Cancer 19, no. 7 (2019): 405‐414. 31101865 10.1038/s41568-019-0149-1

[580] D. Wang and R. N. DuBois , “The Role of Prostaglandin E(2) in Tumor‐Associated Immunosuppression,” Trends in Molecular Medicine 22, no. 1 (2016): 1‐3. 26711015 10.1016/j.molmed.2015.11.003 PMC4762482

[581] A. M. Johnson , E. K. Kleczko , and R. A. Nemenoff , “Eicosanoids in Cancer: New Roles in Immunoregulation,” Frontiers in pharmacology 11 (2020): 595498. 33364964 10.3389/fphar.2020.595498 PMC7751756

[582] S. Zelenay , A. G. van der Veen , J. P. Böttcher , et al., “Cyclooxygenase‐Dependent Tumor Growth Through Evasion of Immunity,” Cell 162, no. 6 (2015): 1257‐1270. 26343581 10.1016/j.cell.2015.08.015 PMC4597191

[583] J. P. Böttcher , E. Bonavita , P. Chakravarty , et al., “NK Cells Stimulate Recruitment of cDC1 Into the Tumor Microenvironment Promoting Cancer Immune Control,” Cell 172, no. 5 (2018): 1022‐1037. e14. 29429633 10.1016/j.cell.2018.01.004 PMC5847168

[584] D. Wang and R. N. DuBois , “Immunosuppression associated With chronic inflammation in the tumor microenvironment,” Carcinogenesis 36, no. 10 (2015): 1085‐1093. 26354776 10.1093/carcin/bgv123 PMC5006153

[585] S. E. Weinberg , L. A. Sena , and N. S. Chandel , “Mitochondria in the regulation of innate and adaptive immunity,” Immunity 42, no. 3 (2015): 406‐417. 25786173 10.1016/j.immuni.2015.02.002 PMC4365295

[586] M. A. Ligtenberg , D. Mougiakakos , M. Mukhopadhyay , et al., “Coexpressed Catalase Protects Chimeric Antigen Receptor‐Redirected T Cells as well as Bystander Cells From Oxidative Stress‐Induced Loss of Antitumor Activity,” J Immunol Baltim Md 1950 196, no. 2 (2016): 759‐766. 10.4049/jimmunol.1401710 PMC4705591 26673145

[587] N. E. Scharping , A. V. Menk , R. D. Whetstone , X. Zeng , and G. M. Delgoffe , “Efficacy of PD‐1 Blockade Is Potentiated by Metformin‐Induced Reduction of Tumor Hypoxia,” Cancer immunology research 5, no. 1 (2017): 9‐16. 27941003 10.1158/2326-6066.CIR-16-0103 PMC5340074

[588] S. E. Weinberg , B. D. Singer , E. M. Steinert , et al., “Mitochondrial complex III is essential for suppressive function of regulatory T cells,” Nature 565, no. 7740 (2019): 495‐499. 30626970 10.1038/s41586-018-0846-z PMC6345596

[589] J. Wei , M. Zhang , and J. Zhou , “Myeloid‐derived suppressor cells in major depression patients suppress T‐cell responses Through the production of reactive oxygen species,” Psychiatry Research 228, no. 3 (2015): 695‐701. 26165964 10.1016/j.psychres.2015.06.002

[590] X. Ma , L. Xiao , L. Liu , et al., “CD36‐mediated ferroptosis dampens intratumoral CD8+ T cell effector function and impairs their antitumor ability,” Cell metabolism 33, no. 5 (2021): 1001‐1012. e5. 33691090 10.1016/j.cmet.2021.02.015 PMC8102368

[591] S. Xu , O. Chaudhary , P. Rodríguez‐Morales , et al., “Uptake of oxidized lipids by the scavenger receptor CD36 promotes lipid peroxidation and dysfunction in CD8+ T cells in tumors,” Immunity 54, no. 7 (2021): 1561‐1577. e7. 34102100 10.1016/j.immuni.2021.05.003 PMC9273026

[592] S. M. Poznanski , K. Singh , T. M. Ritchie , et al., “Metabolic flexibility determines human NK cell functional fate in the tumor microenvironment,” Cell metabolism 33, no. 6 (2021): 1205‐1220. e5. 33852875 10.1016/j.cmet.2021.03.023

[593] T. Rothe , F. Gruber , S. Uderhardt , et al., “12/15‐Lipoxygenase‐mediated enzymatic lipid oxidation regulates DC maturation and function,” Journal of Clinical Investigation 125, no. 5 (2015): 1944‐1954. 25844901 10.1172/JCI78490 PMC4463199

[594] Y. Gao , F. Souza‐Fonseca‐Guimaraes , T. Bald , et al., “Tumor immunoevasion by the conversion of effector NK cells Into type 1 innate lymphoid cells,” Nature Immunology 18, no. 9 (2017): 1004‐1015. 28759001 10.1038/ni.3800

[595] D. N. Khalil , E. L. Smith , R. J. Brentjens , and J. D. Wolchok , “The future of cancer treatment: Immunomodulation, CARs and combination immunotherapy,” Nature reviews Clinical oncology 13, no. 6 (2016): 394. 10.1038/nrclinonc.2016.65 PMC5558237 27118494

[596] W. Zou , J. D. Wolchok , and L. Chen , “PD‐L1 (B7‐H1) and PD‐1 pathway blockade for cancer therapy: Mechanisms, response biomarkers, and combinations,” Science Translational Medicine 8, no. 328 (2016): 328rv4. 10.1126/scitranslmed.aad7118 PMC4859220 26936508

[597] P. Jiang , S. Gu , D. Pan , et al., “Signatures of T cell dysfunction and exclusion predict cancer immunotherapy response,” Nature Medicine 24, no. 10 (2018): 1550‐1558. 10.1038/s41591-018-0136-1 PMC6487502 30127393

[598] J. L. Benci , L. R. Johnson , R. Choa , et al., “Opposing Functions of Interferon Coordinate Adaptive and Innate Immune Responses to Cancer Immune Checkpoint Blockade,” Cell 178, no. 4 (2019): 933‐948. e14. 31398344 10.1016/j.cell.2019.07.019 PMC6830508

[599] M. B. Dong , G. Wang , R. D. Chow , et al., “Systematic Immunotherapy Target Discovery Using Genome‐Scale In Vivo CRISPR Screens in CD8 T Cells,” Cell 178, no. 5 (2019): 1189‐1204. e23. 31442407 10.1016/j.cell.2019.07.044 PMC6719679

[600] Q. Jiang , K. Wang , X. Zhang , et al., “Platelet Membrane‐Camouflaged Magnetic Nanoparticles for Ferroptosis‐Enhanced Cancer Immunotherapy,” Small Weinh Bergstr Ger 16, no. 22 (2020): e2001704. 10.1002/smll.202001704 32338436

[601] R. Song , T. Li , J. Ye , et al., “Acidity‐Activatable Dynamic Nanoparticles Boosting Ferroptotic Cell Death for Immunotherapy of Cancer,” Adv Mater Deerfield Beach Fla 33, no. 31 (2021): e2101155. 10.1002/adma.202101155 34170581

[602] W. B. Coley , “The Treatment of Inoperable Sarcoma by Bacterial Toxins (the Mixed Toxins of the Streptococcus erysipelas and the Bacillus prodigiosus),” Proceedings of the Royal Society of Medicine 3 (1910): 1‐48. Surg Sect. 10.1177/003591571000301601 PMC1961042 19974799

[603] L. T. Nguyen and P. S. Ohashi , “Clinical blockade of PD1 and LAG3–potential mechanisms of action,” Nature Reviews Immunology 15, no. 1 (2015): 45‐56. 10.1038/nri3790 25534622

[604] L. T. Nguyen and P. S. Ohashi , “Clinical blockade of PD1 and LAG3–potential mechanisms of action,” Nature Reviews Immunology 15, no. 1 (2015): 45‐56. 10.1038/nri3790 25534622

[605] W. Wang , I. Kryczek , L. Dostál , et al., “Effector T Cells Abrogate Stroma‐Mediated Chemoresistance in Ovarian Cancer,” Cell 165, no. 5 (2016): 1092‐1105. 27133165 10.1016/j.cell.2016.04.009 PMC4874853

[606] W. Wang , M. Green , J. E. Choi , et al., “CD8+ T cells regulate tumour ferroptosis During cancer immunotherapy,” Nature 569, no. 7755 (2019): 270‐274. 31043744 10.1038/s41586-019-1170-y PMC6533917

[607] G. Delaney , S. Jacob , C. Featherstone , and M. Barton , “The role of radiotherapy in cancer treatment: Estimating optimal utilization From a review of evidence‐based clinical guidelines,” Cancer 104, no. 6 (2005): 1129‐1137. 16080176 10.1002/cncr.21324

[608] K. E. Baidoo , K. Yong , and M. W. Brechbiel , “Molecular pathways: Targeted α‐particle radiation therapy,” Clin Cancer Res Off J Am Assoc Cancer Res 19, no. 3 (2013): 530‐537. 10.1158/1078-0432.CCR-12-0298 PMC3563752 23230321

[609] G. Lei , Y. Zhang , P. Koppula , et al., “The role of ferroptosis in ionizing radiation‐induced cell death and tumor suppression,” Cell Research 30, no. 2 (2020): 146‐162. 31949285 10.1038/s41422-019-0263-3 PMC7015061

[610] L. F. Ye , K. R. Chaudhary , F. Zandkarimi , et al., “Radiation‐Induced Lipid Peroxidation Triggers Ferroptosis and Synergizes With Ferroptosis Inducers,” Acs Chemical Biology 15, no. 2 (2020): 469‐484. 31899616 10.1021/acschembio.9b00939 PMC7180072

[611] X. Lang , M. D. Green , W. Wang , et al., “Radiotherapy and Immunotherapy Promote Tumoral Lipid Oxidation and Ferroptosis via Synergistic Repression of SLC7A11,” Cancer discovery 9, no. 12 (2019): 1673‐1685. 31554642 10.1158/2159-8290.CD-19-0338 PMC6891128

[612] X. Lang , M. D. Green , W. Wang , et al., “Radiotherapy and Immunotherapy Promote Tumoral Lipid Oxidation and Ferroptosis via Synergistic Repression of SLC7A11,” Cancer discovery 9, no. 12 (2019): 1673‐1685. 31554642 10.1158/2159-8290.CD-19-0338 PMC6891128

[613] G. Lei , Y. Zhang , P. Koppula , et al., “The role of ferroptosis in ionizing radiation‐induced cell death and tumor suppression,” Cell Research 30, no. 2 (2020): 146‐162. 31949285 10.1038/s41422-019-0263-3 PMC7015061

[614] X. Li , L. Duan , S. Yuan , X. Zhuang , T. Qiao , and J. He , “Ferroptosis inhibitor alleviates Radiation‐induced lung fibrosis (RILF) via Down‐regulation of TGF‐β1,” J Inflamm Lond Engl 16 (2019): 11. 10.1186/s12950-019-0216-0 PMC6542066 31160885

[615] X. Zhang , X. Xing , H. Liu , et al., “Ionizing radiation induces ferroptosis in granulocyte‐macrophage hematopoietic progenitor cells of murine bone marrow,” International Journal of Radiation Biology 96, no. 5 (2020): 584‐595. 31906761 10.1080/09553002.2020.1708993

[616] O. Warburg and S. Minami , “Versuche an Überlebendem Carcinom‐gewebe,” Klinische Wochenschrift 2, no. 17 (1923): 776‐777.

[617] M. V. Gwangwa , A. M. Joubert , and M. H. Visagie , “Crosstalk Between the Warburg effect, redox regulation and autophagy induction in tumourigenesis,” Cellular & Molecular Biology Letters 23 (2018): 20. 29760743 10.1186/s11658-018-0088-y PMC5935986

[618] N. E. Scharping , D. B. Rivadeneira , A. V. Menk , et al., “Mitochondrial stress induced by continuous stimulation Under hypoxia rapidly drives T cell exhaustion,” Nature Immunology 22, no. 2 (2021): 205‐215. 33398183 10.1038/s41590-020-00834-9 PMC7971090

[619] C. M. Bebber , F. Müller , L. Prieto Clemente , J. Weber , and S. von Karstedt , “Ferroptosis in Cancer Cell Biology,” Cancers 12, no. 1 (2020): 164. 31936571 10.3390/cancers12010164 PMC7016816

[620] S. K. Watkins , N. K. Egilmez , J. Suttles , and R. D. Stout , “IL‐12 rapidly alters the functional profile of tumor‐associated and tumor‐infiltrating macrophages in vitro and in vivo,” J Immunol Baltim Md 1950 178, no. 3 (2007): 1357‐1362. 10.4049/jimmunol.178.3.1357 17237382

[621] S. Nucera , D. Biziato , and M. De Palma , “The interplay Between macrophages and angiogenesis in development, tissue injury and regeneration,” International Journal of Developmental Biology 55, no. 4‐5 (2011): 495‐503. 21732273 10.1387/ijdb.103227sn

[622] E. Dai , L. Han , J. Liu , et al., “Autophagy‐dependent ferroptosis drives tumor‐associated macrophage polarization via release and uptake of oncogenic KRAS protein,” Autophagy 16, no. 11 (2020): 2069‐2083. 31920150 10.1080/15548627.2020.1714209 PMC7595620

[623] Q. Jiang , K. Wang , X. Zhang , et al., “Platelet Membrane‐Camouflaged Magnetic Nanoparticles for Ferroptosis‐Enhanced Cancer Immunotherapy,” Small Weinh Bergstr Ger 16, no. 22 (2020): e2001704. 10.1002/smll.202001704 32338436

[624] C. Wan , Y. Sun , Y. Tian , et al., “Irradiated tumor cell‐derived microparticles mediate tumor eradication via cell killing and immune reprogramming,” Science Advances 6, no. 13 (2020): eaay9789. 32232155 10.1126/sciadv.aay9789 PMC7096163

[625] T. Xu , Y. Ma , Q. Yuan , et al., “Enhanced Ferroptosis by Oxygen‐Boosted Phototherapy Based on a 2‐in‐1 Nanoplatform of Ferrous Hemoglobin for Tumor Synergistic Therapy,” ACS Nano 14, no. 3 (2020): 3414‐3425. 32155051 10.1021/acsnano.9b09426

[626] V. D. Turubanova , I. V. Balalaeva , T. A. Mishchenko , et al., “Immunogenic cell death induced by a new photodynamic therapy based on photosens and photodithazine,” Journal for ImmunoTherapy of Cancer 7, no. 1 (2019): 350. 31842994 10.1186/s40425-019-0826-3 PMC6916435

[627] C. D. Mills , K. Kincaid , J. M. Alt , M. J. Heilman , and A. M. Hill , “M‐1/M‐2 macrophages and the Th1/Th2 paradigm,” J Immunol Baltim Md 1950 164, no. 12 (2000): 6166‐6173. 10.4049/jimmunol.164.12.6166 10843666

[628] F. Zhang , F. Li , G. H. Lu , et al., “Engineering Magnetosomes for Ferroptosis/Immunomodulation Synergism in Cancer,” ACS Nano 13, no. 5 (2019): 5662‐5673. 31046234 10.1021/acsnano.9b00892

[629] A. Linkermann , R. Skouta , N. Himmerkus , et al., “Synchronized renal tubular cell death involves ferroptosis,” PNAS 111, no. 47 (2014): 16836‐16841. 25385600 10.1073/pnas.1415518111 PMC4250130

[630] R. Tang , J. Xu , B. Zhang , et al., “Ferroptosis, necroptosis, and pyroptosis in anticancer immunity,” J Hematol OncolJ Hematol Oncol 13, no. 1 (2020): 110. 32778143 10.1186/s13045-020-00946-7 PMC7418434

[631] T. L. Aaes , A. Kaczmarek , T. Delvaeye , et al., “Vaccination With Necroptotic Cancer Cells Induces Efficient Anti‐tumor Immunity,” Cell reports 15, no. 2 (2016): 274‐287. 27050509 10.1016/j.celrep.2016.03.037

[632] W. Li , G. Feng , J. M. Gauthier , et al., “Ferroptotic cell death and TLR4/Trif signaling initiate neutrophil recruitment After heart transplantation,” Journal of Clinical Investigation 129, no. 6 (2019): 2293‐2304. 30830879 10.1172/JCI126428 PMC6546457

[633] V. D. Turubanova , I. V. Balalaeva , T. A. Mishchenko , et al., “Immunogenic cell death induced by a new photodynamic therapy based on photosens and photodithazine,” Journal for ImmunoTherapy of Cancer 7, no. 1 (2019): 350. 31842994 10.1186/s40425-019-0826-3 PMC6916435

[634] B. Proneth and M. Conrad , “Ferroptosis and necroinflammation, a yet poorly explored link,” Cell Death and Differentiation 26, no. 1 (2019): 14‐24. 30082768 10.1038/s41418-018-0173-9 PMC6294786

[635] H. Wang , O. Bloom , M. Zhang , et al., “HMG‐1 as a late mediator of endotoxin lethality in mice,” Science 285, no. 5425 (1999): 248‐251. 10398600 10.1126/science.285.5425.248

[636] Q. Wen , J. Liu , R. Kang , B. Zhou , and D. Tang , “The release and activity of HMGB1 in ferroptosis,” Biochemical and Biophysical Research Communications 510, no. 2 (2019): 278‐283. 30686534 10.1016/j.bbrc.2019.01.090

[637] A. D. Garg , D. V. Krysko , P. Vandenabeele , and P. Agostinis , “Hypericin‐based photodynamic therapy induces surface exposure of damage‐associated molecular patterns Like HSP70 and calreticulin,” Cancer Immunol Immunother CII 61, no. 2 (2012): 215‐221. 22193987 10.1007/s00262-011-1184-2 PMC11029694

[638] J. Tsoi , L. Robert , K. Paraiso , et al., “Multi‐stage differentiation defines melanoma subtypes With differential vulnerability to drug‐induced iron‐dependent oxidative stress,” Cancer Cell 33, no. 5 (2018): 890‐904. e5. 29657129 10.1016/j.ccell.2018.03.017 PMC5953834

[639] V. S. Viswanathan , M. J. Ryan , H. D. Dhruv , et al., “Dependency of a therapy‐resistant state of cancer cells on a lipid peroxidase pathway,” Nature 547, no. 7664 (2017): 453‐457. 28678785 10.1038/nature23007 PMC5667900

[640] X. Chen , R. Kang , G. Kroemer , and D. Tang , “Broadening horizons: The role of ferroptosis in cancer,” Nature reviews Clinical oncology 18, no. 5 (2021): 280‐296. 10.1038/s41571-020-00462-0 33514910

[641] H. Wang , Y. Cheng , C. Mao , et al., “Emerging mechanisms and targeted therapy of ferroptosis in cancer,” Mol Ther J Am Soc Gene Ther 29, no. 7 (2021): 2185‐2208. 10.1016/j.ymthe.2021.03.022 PMC8261167 33794363

[642] G. Delaney , S. Jacob , C. Featherstone , and M. Barton , “The role of radiotherapy in cancer treatment: Estimating optimal utilization From a review of evidence‐based clinical guidelines,” Cancer 104, no. 6 (2005): 1129‐1137. 16080176 10.1002/cncr.21324

[643] K. E. Baidoo , K. Yong , and M. W. Brechbiel , “Molecular pathways: Targeted α‐particle radiation therapy,” Clin Cancer Res Off J Am Assoc Cancer Res 19, no. 3 (2013): 530‐537. 10.1158/1078-0432.CCR-12-0298 PMC3563752 23230321

[644] A. Balihodzic , F. Prinz , M. A. Dengler , G. A. Calin , P. J. Jost , and M. Pichler , “Non‐coding RNAs and ferroptosis: Potential implications for cancer therapy,” Cell Death and Differentiation 29, no. 6 (2022): 1094‐1106. 35422492 10.1038/s41418-022-00998-x PMC9177660

[645] Y. Luo , G. Niu , H. Yi , et al., “Nanomedicine promotes ferroptosis to inhibit tumour proliferation in vivo,” Redox Biology 42 (2021): 101908. 33674250 10.1016/j.redox.2021.101908 PMC8113035

[646] S. H. Deng , D. M. Wu , L. Li , et al., “miR‐324‐3p reverses cisplatin resistance by inducing GPX4‐mediated ferroptosis in lung adenocarcinoma cell line A549,” Biochemical and Biophysical Research Communications 549 (2021): 54‐60. 33662669 10.1016/j.bbrc.2021.02.077

[647] Z. Song , G. Jia , P. Ma , and S. Cang , “Exosomal miR‐4443 promotes cisplatin resistance in non‐small cell lung carcinoma by regulating FSP1 m6A modification‐mediated ferroptosis,” Life Sciences 276 (2021): 119399. 33781830 10.1016/j.lfs.2021.119399

[648] X. Deng , W. Xiong , X. Jiang , et al., “LncRNA LINC00472 regulates cell stiffness and inhibits the migration and invasion of lung adenocarcinoma by binding to YBX1,” Cell death & disease 11, no. 11 (2020): 945. 33144579 10.1038/s41419-020-03147-9 PMC7609609

[649] C. Gai , C. Liu , X. Wu , et al., “MT1DP loaded by folate‐modified liposomes sensitizes erastin‐induced ferroptosis via regulating miR‐365a‐3p/NRF2 axis in non‐small cell lung cancer cells,” Cell death & disease 11, no. 9 (2020): 751. 32929075 10.1038/s41419-020-02939-3 PMC7490417

[650] L. H. Dong , J. J. Huang , P. Zu , et al., “CircKDM4C upregulates P53 by sponging hsa‐let‐7b‐5p to induce ferroptosis in acute myeloid leukemia,” Environmental Toxicology 36, no. 7 (2021): 1288‐1302. 33733556 10.1002/tox.23126

[651] P. Wu , C. Li , D. M. Ye , et al., “Circular RNA circEPSTI1 accelerates cervical cancer progression via miR‐375/409‐3P/515‐5p‐SLC7A11 axis,” Aging 13, no. 3 (2021): 4663‐4673. 33534779 10.18632/aging.202518 PMC7906137

[652] Z. Liu , Q. Wang , X. Wang , Z. Xu , X. Wei , and J. Li , “Circular RNA cIARS regulates ferroptosis in HCC cells Through interacting With RNA binding protein ALKBH5,” Cell Death Discov 6 (2020): 72. 32802409 10.1038/s41420-020-00306-x PMC7414223

[653] Y. Tang , C. Li , Y. J. Zhang , and Z. H. Wu , “Ferroptosis‐Related Long Non‐Coding RNA signature predicts the prognosis of Head and neck squamous cell carcinoma,” Int J Biol Sci 17, no. 3 (2021): 702‐711. 33767582 10.7150/ijbs.55552 PMC7975700

[654] G. Lei , L. Zhuang , and B. Gan , “Targeting ferroptosis as a vulnerability in cancer,” Nature Reviews Cancer 22, no. 7 (2022): 381‐396. 35338310 10.1038/s41568-022-00459-0 PMC10243716

[655] M. J. Hangauer , V. S. Viswanathan , M. J. Ryan , et al., “Drug‐tolerant persister cancer cells are vulnerable to GPX4 inhibition,” Nature 551, no. 7679 (2017): 247‐250. 29088702 10.1038/nature24297 PMC5933935

[656] V. S. Viswanathan , M. J. Ryan , H. D. Dhruv , et al., “Dependency of a therapy‐resistant state of cancer cells on a lipid peroxidase pathway,” Nature 547, no. 7664 (2017): 453‐457. 28678785 10.1038/nature23007 PMC5667900

[657] J. Tsoi , L. Robert , K. Paraiso , et al., “Multi‐stage Differentiation Defines Melanoma Subtypes With Differential Vulnerability to Drug‐Induced Iron‐Dependent Oxidative Stress,” Cancer Cell 33, no. 5 (2018): 890‐904. e5. 29657129 10.1016/j.ccell.2018.03.017 PMC5953834

[658] Y. Zou , W. S. Henry , E. L. Ricq , et al., “Plasticity of ether lipids promotes ferroptosis susceptibility and evasion,” Nature 585, no. 7826 (2020): 603‐608. 32939090 10.1038/s41586-020-2732-8 PMC8051864

[659] C. Mao , X. Liu , Y. Zhang , et al., “DHODH‐mediated ferroptosis defence is a targetable vulnerability in cancer,” Nature 593, no. 7860 (2021): 586‐590. 33981038 10.1038/s41586-021-03539-7 PMC8895686

[660] S. Zhang , L. Kang , X. Dai , et al., “Manganese induces tumor cell ferroptosis Through type‐I IFN dependent inhibition of mitochondrial dihydroorotate dehydrogenase,” Free Radic Biol Med 193 (2022): 202‐212. Pt 1. 36228830 10.1016/j.freeradbiomed.2022.10.004

[661] W. Zhang , J. Wang , Z. Liu , et al., “Iron‐dependent ferroptosis participated in benzene‐induced anemia of inflammation Through IRP1‐DHODH‐ALOX12 axis,” Free Radic Biol Med 193 (2022): 122‐133. Pt 1. 36244588 10.1016/j.freeradbiomed.2022.10.273

[662] J. A. Stefely and D. J. Pagliarini , “Biochemistry of Mitochondrial Coenzyme Q Biosynthesis,” Trends in Biochemical Sciences 42, no. 10 (2017): 824‐843. 28927698 10.1016/j.tibs.2017.06.008 PMC5731490

[663] N. Rizzardi , I. Liparulo , G. Antonelli , et al., “Coenzyme Q10 Phytosome Formulation Improves CoQ10 Bioavailability and Mitochondrial Functionality in Cultured Cells,” Antioxid Basel Switz 10, no. 6 (2021): 927. 10.3390/antiox10060927 PMC8226950 34200321

[664] H. Lee , F. Zandkarimi , Y. Zhang , et al., “Energy‐stress‐mediated AMPK activation inhibits ferroptosis,” Nature Cell Biology 22, no. 2 (2020): 225‐234. 32029897 10.1038/s41556-020-0461-8 PMC7008777

[665] B. Gan , “Mitochondrial regulation of ferroptosis,” Journal of Cell Biology 220, no. 9 (2021): e202105043. 34328510 10.1083/jcb.202105043 PMC8329737

[666] W. S. Yang , R. SriRamaratnam , M. E. Welsch , et al., “Regulation of ferroptotic cancer cell death by GPX4,” Cell 156, no. 1‐2 (2014): 317‐331. 24439385 10.1016/j.cell.2013.12.010 PMC4076414

[667] R. Brigelius‐Flohé , and M. Maiorino , “Glutathione peroxidases,” Biochimica Et Biophysica Acta 1830, no. 5 (2013): 3289‐3303. 23201771 10.1016/j.bbagen.2012.11.020

[668] T. C. Chen , J. Y. Chuang , C. Y. Ko , et al., “AR ubiquitination induced by the curcumin analog suppresses growth of temozolomide‐resistant glioblastoma Through disrupting GPX4‐Mediated redox homeostasis,” Redox Biology 30 (2020): 101413. 31896509 10.1016/j.redox.2019.101413 PMC6940696

[669] C. Yang , Y. Zhang , S. Lin , Y. Liu , and W. Li , “Suppressing the KIF20A/NUAK1/Nrf2/GPX4 signaling pathway induces ferroptosis and enhances the sensitivity of colorectal cancer to oxaliplatin,” Aging 13, no. 10 (2021): 13515‐13534. 33819186 10.18632/aging.202774 PMC8202845

[670] J. Du , X. Wang , Y. Li , et al., “DHA exhibits synergistic therapeutic efficacy With cisplatin to induce ferroptosis in pancreatic ductal adenocarcinoma via modulation of iron metabolism,” Cell death & disease 12, no. 7 (2021): 705. 34262021 10.1038/s41419-021-03996-y PMC8280115

[671] N. Chaudhary , B. S. Choudhary , S. G. Shah , et al., “Lipocalin 2 expression promotes tumor progression and therapy resistance by inhibiting ferroptosis in colorectal cancer,” International Journal of Cancer 149, no. 7 (2021): 1495‐1511. 34146401 10.1002/ijc.33711

[672] A. L. Turcu , A. Versini , N. Khene , et al., “DMT1 Inhibitors Kill Cancer Stem Cells by Blocking Lysosomal Iron Translocation,” Chem – Eur J 26, no. 33 (2020): 7369‐7373. 32083771 10.1002/chem.202000159

[673] J. L. Roh , E. H. Kim , H. J. Jang , J. Y. Park , and D. Shin , “Induction of ferroptotic cell death for overcoming cisplatin resistance of head and neck cancer,” Cancer Letters 381, no. 1 (2016): 96‐103. 27477897 10.1016/j.canlet.2016.07.035

[674] D. Fu , C. Wang , L. Yu , and R. Yu , “Induction of ferroptosis by ATF3 elevation alleviates cisplatin resistance in gastric cancer by restraining Nrf2/Keap1/xCT signaling,” Cellular & Molecular Biology Letters 26, no. 1 (2021): 26. 34098867 10.1186/s11658-021-00271-y PMC8186082

[675] D. Tang , O. Kepp , and G. Kroemer , “Ferroptosis becomes immunogenic: Implications for anticancer treatments,” Oncoimmunology 10, no. 1 (2020): 1862949. 33457081 10.1080/2162402X.2020.1862949 PMC7781761

[676] X. Luo , H. B. Gong , H. Y. Gao , et al., “Oxygenated phosphatidylethanolamine navigates phagocytosis of ferroptotic cells by interacting With TLR2,” Cell Death and Differentiation 28, no. 6 (2021): 1971‐1989. 33432112 10.1038/s41418-020-00719-2 PMC8185102

[677] C. Xu , S. Sun , T. Johnson , et al., “The glutathione peroxidase Gpx4 prevents lipid peroxidation and ferroptosis to sustain Treg cell activation and suppression of antitumor immunity,” Cell reports 35, no. 11 (2021): 109235. 34133924 10.1016/j.celrep.2021.109235

[678] A. A. Kapralov , Q. Yang , H. H. Dar , et al., “Redox lipid reprogramming commands susceptibility of macrophages and microglia to ferroptotic death,” Nature Chemical Biology 16, no. 3 (2020): 278‐290. 32080625 10.1038/s41589-019-0462-8 PMC7233108

[679] Z. Jiang , S. O. Lim , M. Yan , et al., “TYRO3 induces anti‐PD‐1/PD‐L1 therapy resistance by limiting innate immunity and tumoral ferroptosis,” Journal of Clinical Investigation 131, no. 8 (2021): e139434. 139434. 33855973 10.1172/JCI139434 PMC8262501

[680] G. Lyu , H. Liao , and R. Li , “Ferroptosis and renal fibrosis: Mechanistic insights and emerging therapeutic targets,” Renal Failure 47, no. 1 (2025): 2498629. 40329437 10.1080/0886022X.2025.2498629 PMC12057793

[681] M. Zhang , C. Liu , J. Tu , et al., “Advances in cancer immunotherapy: Historical perspectives, current developments, and future directions,” Molecular cancer 24, no. 1 (2025): 136. 40336045 10.1186/s12943-025-02305-x PMC12057291

[682] X. Zhang , Y. Duan , S. Li , et al., “CRISPR screening identifies PRMT1 as a key pro‐ferroptotic gene via a two‐layer regulatory mechanism,” Cell reports 43, no. 9 (2024): 114662. 39178116 10.1016/j.celrep.2024.114662

[683] X. Wu , Z. Bai , H. Wang , et al., “CRISPR‐Cas9 gene editing strengthens cuproptosis/chemodynamic/ferroptosis synergistic cancer therapy,” Acta Pharm Sin B 14, no. 9 (2024): 4059‐4072. 39309486 10.1016/j.apsb.2024.05.029 PMC11413702

[684] Y. Xiao , M. He , X. Zhang , et al., “Research progress on the mechanism of tumor cell ferroptosis regulation by epigenetics,” Epigenetics 20, no. 1 (2025): 2500949. 40327848 10.1080/15592294.2025.2500949 PMC12064064

[685] W. S. Yang , R. SriRamaratnam , M. E. Welsch , et al., “Regulation of ferroptotic cancer cell death by GPX4,” Cell 156, no. 1‐2 (2014): 317‐331. 24439385 10.1016/j.cell.2013.12.010 PMC4076414

[686] A. C. Araújo , C. E. Wheelock , and J. Z. Haeggström , “The Eicosanoids, Redox‐Regulated Lipid Mediators in Immunometabolic Disorders,” Antioxid Redox Signaling 29, no. 3 (2018): 275‐296. 10.1089/ars.2017.7332 28978222

[687] C. Li , X. Deng , X. Xie , Y. Liu , J. P. Friedmann Angeli , and L. Lai , “Activation of Glutathione Peroxidase 4 as a Novel Anti‐inflammatory Strategy,” Frontiers in pharmacology 9 (2018): 1120. 30337875 10.3389/fphar.2018.01120 PMC6178849

[688] A. J. Marrogi , W. D. Travis , J. A. Welsh , et al., “Nitric oxide synthase, cyclooxygenase 2, and vascular endothelial growth factor in the angiogenesis of non‐small cell lung carcinoma,” Clin Cancer Res Off J Am Assoc Cancer Res 6, no. 12 (2000): 4739‐4744. 11156228

[689] X. Sun , Z. Ou , M. Xie , et al., “HSPB1 as a novel regulator of ferroptotic cancer cell death,” Oncogene 34, no. 45 (2015): 5617‐5625. 25728673 10.1038/onc.2015.32 PMC4640181

[690] L. Jiang , N. Kon , T. Li , et al., “Ferroptosis as a p53‐mediated activity During tumour suppression,” Nature 520, no. 7545 (2015): 57‐62. 25799988 10.1038/nature14344 PMC4455927

[691] Y. Ou , S. J. Wang , D. Li , B. Chu , and W. Gu , “Activation of SAT1 engages polyamine metabolism With p53‐mediated ferroptotic responses,” PNAS 113, no. 44 (2016): E6806‐E6812. 27698118 10.1073/pnas.1607152113 PMC5098629

[692] Y. Zhang , J. Shi , X. Liu , et al., “BAP1 links metabolic regulation of ferroptosis to tumour suppression,” Nature Cell Biology 20, no. 10 (2018): 1181‐1192. 30202049 10.1038/s41556-018-0178-0 PMC6170713

[693] S. J. Wang , D. Li , Y. Ou , et al., “Acetylation Is Crucial for p53‐Mediated Ferroptosis and Tumor Suppression,” Cell reports 17, no. 2 (2016): 366‐373. 27705786 10.1016/j.celrep.2016.09.022 PMC5227654

[694] G. Kroemer , C. Galassi , L. Zitvogel , and L. Galluzzi , “Immunogenic cell stress and death,” Nature Immunology 23, no. 4 (2022): 487‐500. 35145297 10.1038/s41590-022-01132-2

[695] D. Shen , J. Luo , L. Chen , et al., “PARPi treatment enhances radiotherapy‐induced ferroptosis and antitumor immune responses via the cGAS signaling pathway in colorectal cancer,” Cancer Letters 550 (2022): 215919. 36116741 10.1016/j.canlet.2022.215919

[696] J. L. Liang , X. K. Jin , S. M. Zhang , et al., “Specific activation of cGAS‐STING pathway by nanotherapeutics‐mediated ferroptosis evoked endogenous signaling for boosting systemic tumor immunotherapy,” Sci Bull 68, no. 6 (2023): 622‐636. 10.1016/j.scib.2023.02.027 36914548

[697] Q. Ding , W. Tang , X. Li , et al., “Mitochondrial‐targeted brequinar liposome boosted mitochondrial‐related ferroptosis for promoting checkpoint blockade immunotherapy in bladder cancer,” Journal of Controlled Release 363 (2023): 221‐234. 37717657 10.1016/j.jconrel.2023.09.024

[698] E. Dai , L. Han , J. Liu , et al., “Ferroptotic damage promotes pancreatic tumorigenesis Through a TMEM173/STING‐dependent DNA sensor pathway,” Nature Communications 11, no. 1 (2020): 6339. 10.1038/s41467-020-20154-8 PMC7732843 33311482

[699] C. Fang , F. Mo , L. Liu , et al., “Oxidized mitochondrial DNA sensing by STING signaling promotes the antitumor effect of an irradiated immunogenic cancer cell vaccine,” Cell Mol Immunol 18, no. 9 (2021): 2211‐2223. 32398808 10.1038/s41423-020-0456-1 PMC8429462

[700] A. M. Johnson , E. K. Kleczko , and R. A. Nemenoff , “Eicosanoids in Cancer: New Roles in Immunoregulation,” Frontiers in pharmacology 11 (2020): 595498. 33364964 10.3389/fphar.2020.595498 PMC7751756

[701] P. Ni , J. Xie , C. Chen , Y. Jiang , Y. Lu , and X. Hu , “Fluorometric determination of the activity of alkaline phosphatase and its inhibitors based on ascorbic acid‐induced aggregation of carbon dots,” Microchimica Acta 186, no. 3 (2019): 202. 30796533 10.1007/s00604-019-3303-2

[702] Y. Hu , D. Chen , M. Hong , et al., “Apoptosis, Pyroptosis, and Ferroptosis Conspiringly Induce Immunosuppressive Hepatocellular Carcinoma Microenvironment and γδ T‐Cell Imbalance,” Frontiers in immunology 13 (2022): 845974. 35444645 10.3389/fimmu.2022.845974 PMC9013882

[703] E. Dai , L. Han , J. Liu , et al., “Ferroptotic damage promotes pancreatic tumorigenesis Through a TMEM173/STING‐dependent DNA sensor pathway,” Nature Communications 11, no. 1 (2020): 6339. 10.1038/s41467-020-20154-8 PMC7732843 33311482

[704] I. Efimova , E. Catanzaro , L. Van der Meeren , et al., “Vaccination With early ferroptotic cancer cells induces efficient antitumor immunity,” Journal for ImmunoTherapy of Cancer 8, no. 2 (2020): e001369. 33188036 10.1136/jitc-2020-001369 PMC7668384

[705] C. Conche , F. Finkelmeier , M. Pešić , et al., “Combining ferroptosis induction With MDSC blockade renders primary tumours and metastases in liver sensitive to immune checkpoint blockade,” Gut 72, no. 9 (2023): 1774‐1782. 36707233 10.1136/gutjnl-2022-327909 PMC10423492

[706] N. Chande , D. J. Tsoulis , and J. K. MacDonald , “Azathioprine or 6‐mercaptopurine for induction of remission in Crohn's disease,” Cochrane Database of Systematic Reviews (Online), no. 4 (2013): CD000545, 10.1002/14651858.CD000545.pub4. 23633304

[707] W. Wang , M. Green , J. E. Choi , et al., “CD8+ T cells regulate tumour ferroptosis During cancer immunotherapy,” Nature 569, no. 7755 (2019): 270‐274. 31043744 10.1038/s41586-019-1170-y PMC6533917

[708] V. S. Viswanathan , M. J. Ryan , H. D. Dhruv , et al., “Dependency of a therapy‐resistant state of cancer cells on a lipid peroxidase pathway,” Nature 547, no. 7664 (2017): 453‐457. 28678785 10.1038/nature23007 PMC5667900

[709] A. C. Araújo , C. E. Wheelock , and J. Z. Haeggström , “The Eicosanoids, Redox‐Regulated Lipid Mediators in Immunometabolic Disorders,” Antioxid Redox Signaling 29, no. 3 (2018): 275‐296. 10.1089/ars.2017.7332 28978222

[710] X. Liu , T. Wang , W. Wang , et al., “Emerging Potential Therapeutic Targets of Ferroptosis in Skeletal Diseases,” Oxid Med Cell Longev 2022 (2022): 3112388. 35941905 10.1155/2022/3112388 PMC9356861

[711] M. J. Hangauer , V. S. Viswanathan , M. J. Ryan , et al., “Drug‐tolerant persister cancer cells are vulnerable to GPX4 inhibition,” Nature 551, no. 7679 (2017): 247‐250. 29088702 10.1038/nature24297 PMC5933935

[712] X. Chen , R. Kang , G. Kroemer , and D. Tang , “Broadening horizons: The role of ferroptosis in cancer,” Nature reviews Clinical oncology 18, no. 5 (2021): 280‐296. 10.1038/s41571-020-00462-0 33514910

[713] T. Liu , W. Liu , M. Zhang , et al., “Ferrous‐Supply‐Regeneration Nanoengineering for Cancer‐Cell‐Specific Ferroptosis in Combination With Imaging‐Guided Photodynamic Therapy,” ACS Nano 12, no. 12 (2018): 12181‐12192. 30458111 10.1021/acsnano.8b05860

[714] D. Shin , E. H. Kim , J. Lee , and J. L. Roh , “Nrf2 inhibition reverses resistance to GPX4 inhibitor‐induced ferroptosis in head and neck cancer,” Free Radic Biol Med 129 (2018): 454‐462. 30339884 10.1016/j.freeradbiomed.2018.10.426

[715] F. Ursini , M. Maiorino , M. Valente , L. Ferri , and C. Gregolin , “Purification From pig liver of a protein which protects liposomes and biomembranes From peroxidative degradation and exhibits glutathione peroxidase activity on phosphatidylcholine hydroperoxides,” Biochimica Et Biophysica Acta 710, no. 2 (1982): 197‐211. 7066358 10.1016/0005-2760(82)90150-3

[716] K. Shimada , R. Skouta , A. Kaplan , et al., “Global survey of cell death mechanisms reveals metabolic regulation of ferroptosis,” Nature Chemical Biology 12, no. 7 (2016): 497‐503. 27159577 10.1038/nchembio.2079 PMC4920070

[717] H. Fan , G. Yan , Z. Zhao , et al., “A Smart Photosensitizer‐Manganese Dioxide Nanosystem for Enhanced Photodynamic Therapy by Reducing Glutathione Levels in Cancer Cells,” Angewandte Chemie (International ed in English) 55, no. 18 (2016): 5477‐5482. 27010667 10.1002/anie.201510748 PMC4971833

[718] Z. Cong , F. Yuan , H. Wang , et al., “BTB domain and CNC homolog 1 promotes glioma invasion mainly Through regulating extracellular matrix and increases ferroptosis sensitivity,” Biochim Biophys Acta Mol Basis Dis 1868, no. 12 (2022): 166554. 36181980 10.1016/j.bbadis.2022.166554

[719] E. Dierge , E. Debock , C. Guilbaud , et al., “Peroxidation of n‐3 and n‐6 polyunsaturated fatty acids in the acidic tumor environment leads to ferroptosis‐mediated anticancer effects,” Cell metabolism 33, no. 8 (2021): 1701‐1715. e5. 34118189 10.1016/j.cmet.2021.05.016

[720] G. Lei , L. Zhuang , and B. Gan , “The roles of ferroptosis in cancer: Tumor suppression, tumor microenvironment, and therapeutic interventions,” Cancer Cell 42, no. 4 (2024): 513‐534. 38593779 10.1016/j.ccell.2024.03.011

[721] X. Chen , R. Kang , G. Kroemer , and D. Tang , “Broadening horizons: The role of ferroptosis in cancer,” Nature reviews Clinical oncology 18, no. 5 (2021): 280‐296. 10.1038/s41571-020-00462-0 33514910

[722] N. Ebrahimi , S. Adelian , S. Shakerian , et al., “Crosstalk Between ferroptosis and the epithelial‐mesenchymal transition: Implications for inflammation and cancer therapy,” Cytokine & Growth Factor Reviews 64 (2022): 33‐45. 35219587 10.1016/j.cytogfr.2022.01.006

